# Proceedings of the Fifth Biennial Conference of the Society for Implementation Research Collaboration (SIRC) 2019: where the rubber meets the road: the intersection of research, policy, and practice - part 2: Seattle, WA, USA. 12-14 September 2019

**DOI:** 10.1186/s13012-020-01033-8

**Published:** 2020-09-30

**About this supplement**

This article has been published as part of Implementation Science Volume 15 Supplement 2, 2020: Proceedings of the Fifth Biennial Conference of the Society for Implementation Research Collaboration (SIRC) 2019: Where the rubber meets the road: The intersection of research, policy, and practice - Part 2. The first part of this supplement is available online at https://implementationscience.biomedcentral.com/articles/supplements/volume-15-supplement-3. Please note that this is part 2 of 2.

## A1 Public opinion as an outer-contextual factor in health policy D&I research and practice: evidence that the public cares about evidence

### Correspondence: Jonathan Purtle (jpp46@drexel.edu)

#### Dornsife School of Public Health, Drexel University, Philadelphia, PA, USA

**Background**

Barriers to evidence-informed health policymaking are well-established [1]. Although many barriers are technical in nature (e.g., poor communication of research findings) [2], a major impediment stems from the political nature of policymaking [3-4]. Public opinion is a key aspect of politics; and one that is relevant to efforts to promote evidence-informed policymaking because public opinion influences policymakers’ behaviors [5-6]. Thus, if policymakers learn that the public wants their decisions to be supported by evidence, this information could spur policymakers to make more evidence-informed health policy decisions and demonstrate evidence use to their constituents. However, no prior research has examined public opinion about evidence-informed policymaking. This study sought to characterize public opinion about the influence that evidence should, and does, have on health policy development in U.S. Congress relative to other factors and examine differences in opinion by political party affiliation.

**Materials and Methods**

A public opinion survey was conducted in 2018 using the SSRS Probability Panel (N=532), a nationally representative internet panel. Respondents separately rated the extent to which six factors (e.g., evidence, budget impact, industry interests) “should have” and “currently have” influence on U.S. congresspersons’ health policy decisions.

**Results**

Evidence (59%) was the most frequently identified factor that should have “a lot of influence” on health policy development, but only 11% of respondents thought that evidence currently has “a lot of influence” (p<.001). Opinions about evidence did not vary significantly by respondent political party affiliation.

**Conclusions**

There is strong bi-partisan public support for evidence to have much more influence on health policymaking in U.S. Congress. This finding is promising in a time of elevated political polarization in the United States. The survey results have implications for interventions that aim to promote evidence-informed health policymaking. As findings suggest public demand for evidence-informed health policymaking, and prior research demonstrating that public opinion often influences elected policymakers’ behaviors [5-6], interventions that systematically document the extent to which elected policymakers’ actions (e.g., public statements, content of bills introduced, tweets) are evidence-supported and disseminate this information to the public could encourage policymakers to make more evidence-informed health policy decisions.

**References**

1. National Research Council. Using science as evidence in public policy. Schwandt TA, Straf ML, editors. Washington, DC: The National Academies Press; 2012. doi:https://doi.org/10.17226/13460.

2. Oliver K, Innvar S, Lorenc T, Woodman J, Thomas J. A systematic review of barriers to and facilitators of the use of evidence by policymakers. BMC Health Serv Res. 2014;14(1):2.

3. Liverani M, Hawkins B, Parkhurst JO. Political and institutional influences on the use of evidence in public health policy: a systematic review. PloS One. 2013;8(10):e77404.

4. Oliver TR. The politics of public health policy. Annu Rev Public Health. 2006;27:195-233.

5. Burstein P. The impact of public opinion on public policy: a review and an agenda. Polit Research Q. 2003;56(1):29-40.

6. Butler DM, Nickerson DW. Can learning constituency opinion affect how legislators vote? Results from a field experiment. Q J Polit Sci. 2011;6(1):55-83.

## A2 Federal mental health legislation: what becomes law and why? Results from a 30-year review

### Correspondence: Max Crowley (dmc397@psu.edu)

#### College of Health and Human Development, Penn State University, University Park, PA, USA

**Background**

Mental health problems affect millions of individuals and cost over $240 billion annually in increased healthcare, criminal justice, child welfare, education, and labor costs [1]. Ongoing efforts to take evidence-based mental health strategies to scale have encountered a number of barriers to successful uptake and sustained use [2-4]. To overcome these barriers, researchers and advocates often seek to influence public policy to facilitate increased investment in mental health [5-7]. Yet, little work has systematically sought to understand the specific content of past mental health legislation and how the content of the legislation is related to whether a bill becomes law.

**Materials and Methods**

In order to answer these pressing questions about mental health policy, we conducted a mixed methods review of all federal bills introduced to Congress over the last three decades (1989-2019; N=171,861). This includes systematic coding of mental health’s inclusion in federal legislation, quantitative analyses of that inclusion’s relationship with bills becoming law, and qualitative analyses of how mental health policy may be used to improve population health.

**Results**

In the 101st Congress (January 3, 1989, to January 3, 1991), only 14 mental health bills were introduced, comprising only .001% of all bills introduced. By the 115th Congress (January 3, 2017, to January 3, 2019), over 4% of all bills of any type included mental health provisions. In addition to increases in mental health policy across time, we will also present results of analyses that identify characteristics of bills that are more likely to be successfully enacted into law. Finally, results from qualitative analyses will be used to illustrate how elements of mental health policies can facilitate or restrict high-quality implementation.

**Conclusions**

We will discuss implications of these findings through the lens of the outer context of the implementation ecology; specifically, by identifying supports and barriers from federal legislation and policy that may be most likely to promote successful implementation of evidence-based treatments. We will also discuss implications for how best to engage directly with policymakers to support increased availability and effectiveness of mental health services.

**References**

1. O’Connell ME, Boat TF, Warner KE. Preventing mental, emotional, and behavioral disorders among young people: progress and possibilities. Washington, D.C.: National Academies Press; 2009.

2. CDC. The Power of Prevention. Atlanta, GA: Centers for Disease Control and Prevention; 2009.

3. Haskins R, Margolis G. Show me the evidence: Obama’s fight for rigor and evidence in social policy. Washington, DC: Brookings Institution Press; 2015.

4. Baron J, Haskins R. The Obama administration’s evidence-based social policy initiatives: An overview. 2011. http://www.brookings.edu/opinions/2011/1102_budget_deficit_haskins.aspx.

5. Lynch JP, Sabol WJ. Assessing the effects of mass incarceration on informal social control in communities. Criminol Public Pol. 2004;3(2):267-294. doi:10.1111/j.1745-9133.2004.tb00042.x

6. Ratzliff A, Phillips KE, Sugarman JR, Unützer J, Wagner EH. Practical approaches for achieving integrated behavioral health care in primary care settings. Am J Med Qual. 2017;32(2):117-121. doi:10.1177/1062860615618783

7. Crowley M, Scott T, Fishbein DH. Translating prevention research for evidence-based policymaking: Results from the Research-to-Policy Collaboration pilot. Prev Sci. 2018;19(2):260-270.

## A3 Measurement infrastructure for influencing the outer context: Integrating evidence-based practice reporting and client surveys to guide decision-making in a learning health care system

### Noah R. Gubner^1,2^, Felix I. Rodriguez^3^, Rose Krebill-Prather^4^, Kristen Petersen^4^, Sarah Cusworth Walker^1,2^

#### ^1^Department of Psychiatry and Behavioral Sciences, University of Washington, Seattle, WA, USA; ^2^Washington State Evidence-Based Practice Institute, Seattle, WA, USA; ^3^Washington State Health Care Authority, Olympia, WA, USA; ^4^Social and Economic Sciences Research Center, Washington State University, Pullman, WA, USA

##### **Correspondence:** Sarah Cusworth Walker (secwalkr@uw.edu)

**Background**

Legislation in Washington State (HB2536), passed in 2012, mandated the reporting of evidence-based practices (EBPs) for all children-serving systems in Washington State. In response, the Division of Behavioral Health and Recovery and University of Washington’s Evidence-Based Practice Institute developed a measurement method to track use of EBPs in routine services statewide. This method provides a cost effective and adaptable surveillance tool to monitor evidence-based practices (EBPs) and can also be merged with other data sources to monitor disparities in utilization and effectiveness. In this paper we will present this as an example of “outer setting” influences on EBP use as well as proof of concept for this integrated data infrastructure as a means to guide decision-making at the policy level.

**Materials and Methods**

We analyzed a sample of youth (<21 years old) from Washington State who had received at least one hour of publicly funded outpatient mental health (MH) service. Current procedural terminology (CPT) billing codes were used to identify if clients in the sample received any valid EBP psychotherapy sessions during the observation period (May – Oct 2015). Billing data were then tied to self-reported perceptions of outcomes of service from the Child-Family Mental Health Consumer Survey. These outcome data were compared between youth who did and did not receive a valid EBP session during the observation period

**Results**

Among this sample of 1,580 youth, 19.7% (n=312) received at least one valid EBP psychotherapy session. Youth from rural (21.4%) versus urban (16.2%) providers were more likely to have received an EBP session (χ2=6.02, p=0.014). There were significant differences by race/ethnicity (χ2=14.71, p=0.04). Non-Hispanic Whites (20.2%) and American Indians (33.3%) were more likely, while African Americans (12.3%) and Hispanics (15.7%) were less likely to have received a valid EBP session. There was a trend for an interaction between race/ethnicity and receipt of an EBP session on self-reported positive services outcomes, with receipt of an EBP session potentially being associated with more positive services outcomes among youth who were non-White versus White.

**Conclusions**

As a proof of concept, we demonstrate that billing data provides a cost-effective tool to monitor the receipt of EBP MH sessions and can be tied to other data sources to examine outcomes across a health network.

## A4 Giving the outer setting its due: adapting the stages of implementation completion to policy and system-level change efforts

### Eric J. Bruns^1^, Jonathan R. Olson^1^, Philip H. Benjamin^1^, Lisa Saldana^2^

#### ^1^Department of Psychiatry & Behavioral Sciences, University of Washington, Seattle, WA, USA; ^2^Oregon Social Learning Center, Eugene, OR, USA

##### **Correspondence:** Eric J. Bruns (ebruns@uw.edu)

**Background**

Successful implementation of Wraparound care coordination for youth with complex behavioral health needs requires hospitable policy and financing conditions [1]. Thus, when the “rubber meets the road,” implementation support for Wraparound requires attention to the “outer setting” of the implementation ecology [2], including cross-agency coordination, Medicaid payment reform, and cross-sector information systems. Unfortunately, existing implementation measures are scarce at the outer context [3], as is research on relevant outer setting strategies [4]. This presentation describes efforts to track Wraparound implementation at system and organizational levels using an adaptation of the Stages of Implementation Completion (SIC) [5].

**Materials and Methods**

The SIC assesses implementation across eight stages and three phases: pre-implementation, implementation, and sustainment. We adapted and added SIC items to align with multilevel Wraparound implementation support as enacted by the National Wraparound Implementation Center (NWIC), including an array of state systems (outer setting) variables such as state leadership engagement, cross-system communication and collaboration, financing strategies, and contract requirements. We have collected data for two pilot states and are completing collation for eight additional states.

**Results**

Adaptation of the SIC entailed adding 12 items and removing 4. Furthermore, we operationalized each SIC variable in terms of measurable Wraparound processes and activities. Preliminary results from two pilot states indicate high completion rates across stages for both states (completion percentages from 60% to 100%). However, states differed significantly in their time to completion, with State 1 averaging 3.75 months for completion of stages and state 2 averaging 26.38 months. Item- and stage-level analyses revealed that State 2 struggled to engage state leadership in implementation. State 1 adopted a new approach to building Wraparound implementation infrastructure – investing in Care Management Entities (CMEs) [6], while State 2 relied on Community Mental Health Centers.

**Conclusions**

Findings provide proof of concept for incorporating outer setting items into an established implementation measure and underscore the influence of outer context in Wraparound implementation. The presentation will show how NWIC is using this measure to support states to build systems that facilitate better implementation and outcomes, while also promoting new and needed research for mental health and implementation science.

**References**

1. Bruns EJ, Sather A, Pullmann MD, Stambaugh LF. National trends in implementing wraparound: results from the state wraparound survey. J Child Fam Stud. 2011;20(6):726-735.

2. Damschroder LJ, Aron DC, Keith RE, Kirsh SR, Alexander JA, Lowery JC. Fostering implementation of health services research findings into practice: a consolidated framework for advancing implementation science. Implement Sci. 2009; 4:50. doi:10.1186/1748-5908-4-50.

3. Lewis CC, Stanick CF, Martinez RG, Weiner BJ, Kim M, Barwick M, Comtois KA. The Society for Implementation Research Collaboration Instrument Review Project: a methodology to promote rigorous evaluation. Implement Sci. 2015; 10:2.

4. Purtle J, Peters R, Brownson RC. A review of policy dissemination and implementation research funded by the National Institutes of Health, 2007–2014. Implement Sci. 2016;11:1.

5. Saldana L, Chamberlain P, Wang W, Brown CH. Predicting program start-up using the stages of implementation measure. Adm Policy Ment Health. 2012:39(6):419-25.

6. Center for Health Care Strategies. Care management entities: a primer. 2011. https://www.chcs.org/resource/care-management-entities-a-primer/. Accessed 27 March 2019.

## A5 Cross-collaborations among researchers, community, government agencies, and a federal funding agency to support implementation of evidence-based cardiovascular disease prevention in primary care: the EvidenceNOW Initiative

### Donna Shelley^1^, Michael Parchman^2^, Robert McNellis^3^

#### ^1^School of Medicine, New York University, New York, NY, USA; ^2^Kaiser Permanente Washington Health Research Institute, Seattle, WA, USA; ^3^Agency for Healthcare Research and Quality, Rockville, MD, USA

##### **Correspondence:** Donna Shelley (donna.shelley@nyulangone.org)

**Background**

EvidenceNOW is an Agency for Healthcare Research and Quality (AHRQ)-funded research initiative focused on helping primary care practices improve the delivery of the “ABCS” of cardiovascular disease (CVD) prevention: Aspirin in high-risk individuals, Blood pressure control, Cholesterol management, and Smoking cessation. Seven cooperatives used practice facilitation (PF) as a unifying strategy to support the implementation and dissemination of evidence-based care for CVD risk factors. Each cooperative enrolled over 200 practices in their region. Cooperatives created an infrastructure to engage stakeholder organizations in their region to facilitate the initiative. AHRQ created infrastructure that included a national evaluator (ESCALATES) and committees and meetings that fostered cross-cooperative collaboration and dissemination.

**Materials and Methods**

This panel includes an AHRQ program officer who will discuss the infrastructure created and lessons learned for guiding the design of future funding opportunities [1] and case studies demonstrating how two cooperatives, HealthyHearts New York City [2] and Healthy Hearts Northwest [3], partnered with community and government agencies in their region to accomplish EvidenceNOW goals. The discussant is the ESCALATES Principal Investigator.

**Results**

The aligned efforts of Cooperatives, AHRQ, and ESCALATES facilitated the collective expansion of capacity for practice transformation and rigorous evaluation. We found that: (a) large-scale, federally-funded research initiatives were strengthened through infrastructure that fostered collaboration across grantees (demonstrated by 12 cross-cooperative publications to date); (b) an external evaluator added tremendous value in supporting cross-cooperative collaboration and amplified opportunities for dissemination; (c) engaging with partner organizations early helped assess fit with organizational strategic plans, capacity, readiness for change, and data collection systems; (d) applying an implementation science framework [4] was necessary to guide intervention development and assessment; and (e) stakeholder organizations valued being included in research and funder meetings and dissemination activities.

**Conclusions**

For cooperatives, aligning goals, and attending to opportunities to grow research and quality improvement capacity among partnering agencies facilitated strong, enduring partnerships for practice transformation. Funders have a role to play in facilitating collaborations among cooperatives and evaluation. The findings that emerge through the efforts of multi-level partners are greater than the sum of the parts and further the field of dissemination and implementation science.

**References**

1. Cohen DJ, Balasubramanian BA, Gordon L, Marino M, Ono S, Solberg LI, Crabtree BF, Strange KC, Davis M, Miller WL, Damschroder LJ, McConnell KJ, Creswell J. A national evaluation of a dissemination and implementation initiative to enhance primary care practice capacity and improve cardiovascular disease care: the ESCALATES study protocol. Implement Sci. 2016;11(86):1-13. doi:10.1186/s13012-016-0449-8

2. Shelley DR, Ogedegbe G, Anane S, Wu WY, Goldfield K, Gold HT, Kaplan S, Berry C. Testing the use of practice facilitation in a cluster randomized stepped-wedge design trial to improve adherence to cardiovascular disease prevention guidelines: HealthyHearts NYC. Implement Sci. 2015;11(1):88. doi:10.1186/s13012-016-0450-2

3. Parchman ML, Fagnan LJ, Dorr DA, Evans P, Cook AJ, Penfold RB, Hsu C, Cheadle A, Baldwin LM, Tuzzio L. Study protocol for “Healthy Hearts Northwest”: a 2 × 2 randomized factorial trial to build quality improvement capacity in primary care. Implement Sci. 2016;11(138):1-9. doi:10.1186/s13012-016-0502-7

4. Damschroder LJ, Aron DC, Keith RE, Kirsh SR, Alexander JA, Lowery JC. Fostering implementation of health services research findings into practice: a consolidated framework for advancing implementation science. Implement Sci. 2009;4(1):50. doi:10.1186/1748-5908-4-50

## A6 A collaboration between practitioners, intermediaries, and researchers to increase access to evidence-based chronic pain care

### Jessica Chen^1,2^, Lisa Glynn^2^, Timothy Dawson^1,2^, Hannah Gelman^3^, Steven Zeliadt^1,3^

#### ^1^Department of Psychology, University of Washington, Seattle, WA, USA; ^2^VA Puget Sound, Seattle, WA, USA; ^3^Seattle-Denver Center of Innovation, VA HSR&D, Seattle, WA, USA

##### **Correspondence:** Jessica Chen (jessica.chen663@va.gov)

**Background**

This presentation describes initial lessons learned from a collaboration between front-line providers, intermediaries, and researchers to implement a telehealth model of chronic pain care to rural VA clinics.

As opioid-related overdoses have increased exponentially in the U.S. over the last two decades, there has been greater emphasis on increasing access to non-opioid and non-pharmacological pain management, particularly to rural and under-served areas and in primary care settings.

**Materials and methods**

To address these priorities, front-line clinicians and clinical administrators at one Veterans Health Administration (VA) facility initiated a collaboration with VA implementation researchers to implement a novel hub-and-spoke telehealth model for chronic pain management (TelePain), which will deliver patient education, evidence-based psychotherapies, movement therapies (e.g., yoga for lower back pain), and non-opioid pharmacotherapies from a central specialty pain “hub” to rural VA primary care clinic “spokes.” The goal of the collaboration between practitioners, intermediaries, and researchers was to obtain grant funding to support external facilitation and evaluation activities.

**Results**

There were several lessons learned from this collaborative process regarding health care system changes that may better foster collaboration between research, policy, and practice. One was that the unpredictable and cyclical nature of grant funding can interfere with delivering implementation support when it is needed, namely at the time of clinical services rollout. Therefore, when clinical programs are being designed, they should consider building in funding for implementation support and evaluation from the outset. A second lesson learned was that implementation intermediaries, individuals who have experience with both care delivery and evaluative sciences, may be particularly important for bridging the cultural divide between researchers and practitioners.

**Conclusions**

Embedding intermediaries as fully-funded staff in the TelePain clinical program has helped to align research aims with clinical priorities and may support the long-term sustainability of implementation efforts.

## A7 Untangling trauma-related knowledge and practice changes among brokers in a community-based learning collaborative: role of interprofessional collaboration

### Funlola Are^1^, Rochelle Hanson^1^, Samuel Peer^2^, Ben Saunders^1^

#### ^1^Department of Psychiatry and Behavioral Sciences, Medical University of South Carolina, Charleston, SC, USA; ^2^Psychology Department, Idaho State University, Pocatello, ID, USA

##### **Correspondence:** Funlola Are *(*aref@musc.edu*)*

**Background**

While evidence-based treatments (EBTs) exist to ameliorate trauma-related mental health problems, many children do not receive them [1]. Possible reasons to account for this include limited availability of EBTs and poor collaboration amongst professionals involved in youth service provision [2]. Brokers, often child welfare workers, serve an important intermediary role in improving service access for youth [3], but they are often trained separately from clinical providers, precluding the opportunity to promote cross-discipline collaboration. Community Based Learning Collaboratives (CBLC) use specific training/ implementation strategies involving multidisciplinary stakeholders to foster collaboration and build community capacity for trauma-focused EBTs [4]. The broker curriculum includes information about trauma impact, trauma-focused EBTs, family engagement strategies, and trauma-focused treatment planning, while also providing opportunities for cross-discipline training.

**Materials and Methods**

This presentation examines changes in trauma-related knowledge, practices and interprofessional collaboration among n = 33 brokers participating in CBLCs conducted as part of a statewide dissemination initiative. Brokers completed self-report measures examining knowledge of trauma-related topics taught as part of the CBLC curriculum, organizational climate, interprofessional collaboration, and trauma-related practices (e.g., assessment, psychoeducation) pre- and post-CBLC. Bivariate correlations were computed in SPSS, and interaction effects were probed using the PROCESS macro in SPSS.

**Results**

Analyses revealed that increases in knowledge about the trauma-related curriculum topics were significantly associated with positive changes in broker practices following CBLC participation. However, neither changes related to interprofessional collaboration nor organizational climate were significantly related to changes in broker practices. Moderation analyses revealed a significant interaction effect between interprofessional collaboration and knowledge of evidence-based treatment planning pre- and post-CBLC [F (1, 29) = 5.14; p = .03; R2 = .25]. Those participants who reported the greatest pre- to post-CBLC change in interprofessional collaboration also reported a significant increase in skills related to evidence-based treatment planning (t = 2.62, p = .03, 95% CI = .04, 0.84).

**Conclusions**

Study findings suggest that brokers play an important role in building community capacity for EBT access and that cross-discipline strategies help to foster collaborative relationships among youth service providers. Implications for future research, policy and practice will be addressed.

**References**

1. U.S. Department of Health & Human Services, Administration for Children and Families, Administration on Children, Youth and Families, Children’s Bureau. Child maltreatment 2016. 2018. https://www.acf.hhs.gov/cb/research-data-technology/statistics-research/child-maltreatment

2. Fitzgerald MM, Torres MM, Shipman K, Gorrono J, Kerns SEU, Dorsey S. Child welfare caseworkers as brokers of mental health services: a pilot evaluation of Project Focus Colorado. Child Maltreat. 2015; 20(1);37-49. https://doi.org/10.1177/1077559514562448

3. Stiffman AR, Pescosolido B, Cabassa LJ. Building a model to understand youth service access: the gateway provider model. Ment Health Serv Res. 2004;6:189. https://doi.org/10.1023/B:MHSR. 0000044745.09952.33

4. Hanson RF, Saunders BE, Ralston E, Moreland AD, Peer SO, Fitzgerald MM. Statewide implementation of trauma-specific treatment services using the Community-Based Learning Collaborative Model. Psychol Serv. 2019;16:171-181.

## A8 Change in patient outcomes after augmenting a low-level implementation strategy in community practices that are slow to adopt a collaborative chronic care model: a cluster randomized implementation trial

### Shawna Smith^1^, Daniel Almirall^1^, Katherine Prenovost^1^, Mark Bauer^2^, Celeste Liebrecht^1^, Daniel Eisenberg^1^, Amy Kilbourne^1^

#### ^1^Institute for Healthcare Policy and Innovation University of Michigan, Ann Arbor, MI, USA; ^2^Department of Psychiatry, Harvard Medical School-VA Boston, Boston, MA, USA

##### **Correspondence:** Shawna Smith (shawnana@umich.edu)

**Backgr*ound***

Implementation strategies are essential for promoting uptake of evidence-based practices and for patients to receive optimal care [1]. Yet strategies differ substantially in their intensity and feasibility. Lower-intensity strategies (e.g., training, technical support) are commonly used, but may be insufficient for all clinics. Limited research has examined the comparative effectiveness of augmentations to low-level implementation strategies for non-responding clinics [2-3].

**Materials and methods**

In this Hybrid Type III implementation-effectiveness study [4], we compare the effectiveness of two augmentation strategies, External Facilitation (EF) vs. External + Internal Facilitation (EF/IF)[5] for improving uptake of an evidence-based collaborative care model (CCM) on 18-month mental health outcomes for patients with depression at community-based clinics initially non-responsive to lower-level implementation support.

**Results**

Providers initially received support using a low-level implementation strategy, Replicating Effective Programs (REP). After 6 months, non-responsive clinics that had failed to deliver a clinically significant dose of the CCM to >10 patients were randomized to augment REP with either EF or EF/IF. Mixed effects models evaluated the comparative effectiveness of the two augmentations on patient outcomes at 18 months. The primary outcome was patient SF-12 mental health score; secondary outcomes were PHQ-9 depression score and self-reported receipt of the CCM during months 6 through 18.

27 clinics were non-responsive after 6 months of REP. 13 clinics (N=77 patients) were randomized to REP+EF and 14 (N=92) to REP+EF/IF. At 18 months, patients in the REP+EF/IF arm had worse SF-12 (diff=8.38; 95%CI=3.59, 13.18) and PHQ-9 scores (diff=1.82; 95%CI=-0.14, 3.79), and lower odds of CCM receipt (OR=0.67, 95% CI=0.30,1.49) than REP+EF patients.

**Conclusion**

Patients at initially non-responsive community-based clinics that were randomized to receive the more intensive EF/IF augmentation saw less improvement in mood symptoms at 18 months than those the received the EF augmentation, and were also no more likely to receive the CCM. While EF generally appeared to help clinics, a number of EF/IF clinics experienced barriers in implementing the IF strategy with fidelity, including failing to identify an IF. For large-scale implementation in community-based clinics, augmenting REP with EF for sites that need additional support may be more feasible, and ultimately more effective, than a more intensive EF/IF augmentation.

**Trial Registration:** ClinicalTrials.gov NCT02151331

**References**

1. Proctor EK, Powell BJ, McMillen JC. Implementation strategies: recommendations for specifying and reporting. Implement Sci. 2013;8:139. doi:10.1186/1748-5908-8-139

2. Bonham AC, Solomon MZ. Moving comparative effectiveness research into practice: implementation science and the role of academic medicine. Health Aff. 2010;29.10:1901-1905.

3. Atkins D, Kupersmith J. Implementation research: A critical component of realizing the benefits of comparative effectiveness research. Am J Med. 2010;123(12):e38-e45.

4. Curran GM, Bauer M, Mittman B, Pyne JM, Stetler C. Effectiveness-implementation hybrid designs: combining elements of clinical effectiveness and implementation research to enhance public health impact. Med Care. 2012; 50(3):217-26.

5. Kilbourne, AM, Almirall, D, Eisenberg, D, Waxmonsky, J, Goodrich, DE, Fortney, JC, Kyle, J. Protocol: Adaptive Implementation of Effective Programs Trial (ADEPT): Cluster randomized SMART trial comparing a standard versus enhanced implementation strategy to improve outcomes of a mood disorders program. Implement Sci. 2010;9(1):132.

## A9 A community-based implementation roadmap to inform scalability, sustainability, and spread of evidence-based collaborative care interventions

### Amy Rusch, Shawna Smith, Lindsay Decamp, Celeste Liebrecht, Gregory Dalack, Amy Kilbourne

#### Institute for Healthcare Policy and Innovation University of Michigan, Ann Arbor, MI, USA

##### **Correspondence:** Amy Rusch (amyrusch@med.umich.edu)

**Background**

Evidence-based collaborative care interventions (CCIs) can improve health by mitigating the access gap in mental health care treatment. However, CCIs can be difficult to implement and efforts to scale up CCIs are often stymied by a lack of practitioner knowledge for identifying and addressing barriers to implementation [1]. Implementation roadmaps provide a playbook outlining critical steps practitioners should follow in scaling up CCIs to new settings that overcome implementation barriers, measure implementation success, and garnering leadership support for longer-term CCI sustainability [2]. We present an Implementation Roadmap [3] that guides community-based CCI implementation based on the experiences of stakeholders successfully implementing a broad spectrum of CCIs through the Michigan Mental Health Integration Partnership (MIP) [4]. The goal of MIP is to support the scale up and spread of CCIs that enhance access to care for Medicaid-eligible consumers with behavioral healthcare needs, while also providing an in-situ implementation laboratory for informing sustained uptake of CCIs across a variety of community-based settings.

**Materials and Methods**

Semi-structured interviews were carried out with stakeholders from successfully adopted MIP CCI projects to define common barriers, challenges, and implementation strategies deployed by the project teams. Interviews were transcribed and analyzed for common themes that identified a series of critical steps scaffolding the CCI implementation process and accompanying metrics for evaluating implementation progress. 25 interviews of key stakeholders were conducted across 7 successful MIP implementation teams, including 11 providers at implementation sites and 14 researchers/project managers.

**Results**

Stakeholders commonly identified specific steps that overcame barriers to CCI implementation, including deployment of web-based tools for facilitating implementation, embedding key metrics of implementation success, garnering upper level administration buy-in upfront, and specifying a process for tailoring implementation strategy deployment to specific site needs. These findings informed our resulting Implementation Roadmap, which includes eleven critical implementation steps and evaluative metrics for investigators implementing CCIs to consider across pre-implementation, implementation, and sustainability phases.

**Conclusions**

Maximal CCI public health impact requires improved reach. Our Implementation Roadmap provides a clear and practical guide for early stage community CCI implementation efforts, and ensure practitioners collect key metrics and systematically address barriers in ways that are foundational for larger scale, sustainable implementation efforts.

**References**

1. Beidas RS, Stewart RE, Adams DR, Fernandez T, Lustbader S, Powell BJ, Aarons GA, Hoagwood KE, Evans AC, Hurford MO, Rubin R, Hadley T, Mandell DS, Barg FK. A multi-level examination of stakeholder perspectives of implementation of evidence-based practices in a large urban publicly-funded mental health system. Adm Policy Ment Health. 2016;43(6):893-908.

2. Proctor E, Luke D, Calhoun A, McMillen C, Brownson R, McCrary S, Padek M. Sustainability of evidence-based healthcare: research agenda, methodological advances, and infrastructure support. Implement Sci. 2015;10(88):1-13.

3. Bolboli AS, Reiche M. A model for sustainable business excellence: implementation and the roadmap. TQM J. 2013;25(4), 331-346.

4. Heller D J, Hoffman C, Bindman AB. Supporting the needs of state health policy makers through university partnerships. J Health Polit Policy Law. 2014;39(3):667-677.

## A10 The collaborative chronic care model for mental health conditions: from partnered implementation trial to scale-up and spread

### Mark Bauer^1,2^, Kendra Weaver^3^, Bo Kim^1,2^, Christopher Miller^1,2^, Robert Lew^1,4^, Kelly Stolzmann^1^, Jennifer Sullivan^1,4^, Rachel Riendeau^1,5^, Samantha Connolly^1,2^, Jeffery Pitcock^6^, Stig Ludvigsen^1^, A. Rani Elwy^1,7^

#### ^1^Veterans Health Administration, Boston, MA, USA; ^2^Department of Psychiatry, Harvard Medical School Boston, MA, USA; ^3^VA Office of Mental Health and Suicide Prevention, Washington, DC, USA; ^4^School of Public Health, Boston University; ^5^Department of Anthropology, University of Iowa, Iowa City, IA, USA; ^6^Central Arkansas Veterans Healthcare System, Little Rock, AR, USA; ^7^Department of Psychiatry and Human Behavior, Brown University Warren Alpert School of Medicine, Providence, RI, USA

##### **Correspondence:** Mark Bauer (mark.bauer@va.gov)

**Background**

Collaborative Chronic Care Models (CCMs) have extensive controlled trial evidence for effectiveness in serious mental illnesses [1-2], but there is little evidence regarding feasibility or impact in typical practice conditions. In partnership with the VA Office of Mental Health and Suicide Prevention (OMHSP) we conducted a randomized, stepped wedge implementation trial using blended internal-external facilitation [3] to implement CCMs in Behavioral Health Interdisciplinary Program (BHIP) teams in the general mental health clinics of nine VA medical centers [4-5]. Based on experience in this trial, OMHSP launched an initiative to scale-up and spread the implementation effort more broadly.

**Materials and Methods**

Our research team and OMHSP engaged with Transformational Coaches (T-Coaches) from the VA Office of Veterans Access to Care to serve as external facilitators to engage additional VA medical centers across the country. T-Coaches are senior facilitators with skills in team-building and process redesign from diverse professional disciplines. Trial external facilitators and OMHSP leadership trained 17 T-Coaches in methods used in the trial. Sites were recruited by OMHSP. Blended facilitation was conducted for 12 months as in the implementation trial. Each of the T-Coaches partnered with a BHIP-CCM subject matter expert for the effort, and they conferred on a regular basis throughout the year.

**Results**

Thirty-nine sites were approached; of these 35 (89.7%) signed a letter of agreement. Of these, 28 facilities (80.0%) completed a site visit and entered the ongoing virtual facilitation process. Of these, 21 facilities (75.0%) completed the one-year facilitation and submitted CCM-concordance process summaries. The proportion of CCM-concordant processes ranged widely across facilities, with the more concordant sites equaling rates seen in the implementation trial and a broader low-end distribution (trial: 44-89, T-Coach scale-up: 13-93%).

**Conclusions**

In summary there was, not surprisingly, a broader range of CCM-concordance among these scale-up sites compared to the implementation trial. Nonetheless, taken together, the two BHIP-CCM implementation efforts reached 30 VA medical centers, of which 17 (56.7%) aligned over half of designated care processes with the evidence-based CCM. With strong operational partnerships and support, implementation trial efforts can be scaled up and spread to achieve broader healthcare system impact.

**Trial Registration:** ClinicalTrials.gov NCT02543840

**References**

1. Woltmann E, Grogan-Kaylor A, Perron B, Georges H, Kilbourne AM, Bauer MS. Comparative effectiveness of collaborative chronic care models for mental health conditions across primary, specialty, and behavioral health care settings: systematic review and meta-analysis. Am J Psychiatry. 2012; 169:790-804.

2. Miller CJ, Grogan-Kaylor A, Perron BE, Kilbourne AM, Woltmann E, Bauer MS. Collaborative chronic care models for mental health conditions: cumulative meta-analysis and metaregression to guide future research and implementation. Med Care. 2013;51:922-930.

3. Kirchner JE, Ritchie MJ, Pitcock JA, Parker LE, Curran GM, Fortney JC. Outcomes of a partnered facilitation strategy to implement primary care-mental health. J Gen Intern Med. 2014;29 Suppl 4:904-912.

4. Bauer MS, Miller C, Kim B, Lew R, Weaver K, Coldwell C, Henderson K, Holmes S, Seibert MN, Stolzmann K, Elwy AR, Kirchner J. Partnering with health system operations leadership to develop a controlled implementation trial. Implement Sci. 2016;11:22.

5. Bauer MS, Miller C, Kim B, Lew R, Stolzman K, Sullivan J, Reindeau R, Pitcock J, Williamson A, Connolly S, Elwy AR, Weaver K. Effectiveness of implementing a Collaborative Chronic Care Model for clinician teams on patient outcomes and health status in mental health: a randomized clinical trial. JAMA Netw Open. 2019;2:e190230.

## A11 A randomized stepped wedge hybrid-II trial to implement the collaborative chronic care model in VA general mental health clinics

### Christopher Miller^1^, Bo Kim^1^, Robert Lew^1^, Kelly Stolzmann^1^, Jennifer Sullivan^1^, Rachel Riendeau^2^, Jeffery Pitcock^3^, Alicia Williamson^4^, Samantha Connolly^1^, A. Rani Elwy^5^, Kendra Weaver^6^, Mark Bauer^1^

#### ^1^VA Boston Healthcare System, Boston, MA, USA; ^2^Department of Anthropology, University of Iowa, Iowa City, IA, USA; ^3^Central Arkansas Veterans Healthcare System, Little Rock, AR, USA; ^4^School of Information, University of Michigan, Ann Arbor, MI, USA; ^5^Brown University Warren Alpert School of Medicine, Providence, RI, USA; ^6^VA Office of Mental Health and Suicide Prevention, Washington, DC, USA

##### **Correspondence:** Christopher Miller (christopher.miller8@va.gov)

**Background**

Collaborative Chronic Care Models (CCMs) have extensive controlled trial evidence for effectiveness in serious mental illnesses [1], but there is little evidence regarding feasibility or impact in typical practice conditions. We determined the effectiveness of implementation facilitation on establishing the CCM in mental health teams, and its impact on health outcomes of team-treated individuals.

**Materials and Methods**

We used a randomized stepped wedge trial in Behavioral Health Interdisciplinary Program (BHIP) teams in outpatient general mental health clinics of nine VA facilities, using blended internal-external facilitation. Facilitation combined a study-funded external facilitator with a facility-funded internal facilitator working with a designated team for one year. We hypothesized that facilitation would be associated with improvements in both implementation and intervention outcomes (hybrid-II trial) [2]. Implementation outcomes included the clinician Team Development Measure (TDM) and proportion of CCM-concordant team care processes. The study was powered for the primary health outcome, VR-12 Mental Component Score (MCS). All Veterans treated by designated teams were included for hospitalization analyses, based on administrative data; a randomly selected sample was identified for health status interview. Individuals with dementia were excluded. For implementation outcomes, 62 clinicians were surveyed; site process summaries were rated for CCM concordance.

**Results**

The population (n=5,596) included 881 (15%) women, average age 52.2+14.5. The interviewed sample (n=1,050) was similar, but oversampled for women (n=210, 20.0%). Facilitation was associated with improvements in TDM subscales for role clarity and team primacy. Percentage of CCM-concordant processes achieved varied (44-89%). No improvement in veteran self-ratings, including the primary outcome, was seen. However, in post-hoc analyses MCS improved in veterans with >3 treated mental health diagnoses versus others. Mental health hospitalization rate demonstrated a robust drop during facilitation; this finding withstood four internal validity tests [3].

**Conclusions**

Working solely at the clinician level with minimal study-funded support, CCM implementation yielded provider and Veteran benefits. Although impact on self-reported overall population health status was negligible, health status improved for complex individuals, and hospitalization rate declined. Facilitating CCM implementation provides a potential model for realigning VA outpatient general mental health care with an evidence-based model that improves provider team function and Veteran outcomes.

**Trial Registration:** ClinicalTrials.gov NCT02543840

**References**

1. Miller CJ, Grogan-Kaylor A, Perron BE, Kilbourne AM, Woltmann E, Bauer MS. Collaborative chronic care models for mental health conditions: cumulative meta-analysis and meta-regression to guide future research and implementation. Med Care. 2013;51:922-930.

2. Bauer MS, Miller CJ, Kim B, Lew R, Weaver K, Coldwell C, Henderson K, Holmes SK, Nealon-Seibert M, Stolzmann K, Elwy AR, Kirchner J. Partnering with health system operations leadership to develop a controlled implementation trial. Implement Sci. 2016;11(22):1-11.

3. Bauer MS, Miller CJ, Kim B, Lew R, Stolzmann K, Sullivan J, Riendeau R, Pitcock J, Williamson A, Connolly S, Elwy AR, Weaver K. Effectiveness of implementing a collaborative chronic care model for clinician teams on patient outcomes and health status in mental health: a randomized clinical trial. JAMA Netw Open. 2019;2(3):e190230. doi:10.1001/jamanetworkopen.2019.0230.

## A12 Development, adaptation, and preliminary evaluation of the Leadership and Organizational Change for Implementation strategy

### Mark G. Ehrhart^1^, Marisa Sklar^2,3^, Kristine Carandang^2,3^, Melissa R. Hatch^2,3^, Joanna C. Moullin^4^, Gregory A. Aarons^2,3^

#### ^1^Psychology Department, University of Central Florida, Orlando, FL, USA; ^2^Department of Psychiatry, University of California San Diego, San Diego, CA, USA; ^3^Child and Adolescent Services Research Center, University of California San Diego, San Diego, CA, USA; ^4^Faculty of Health Sciences, Curtin University, Perth, Australia

##### **Correspondence:** Gregory A. Aarons (gaarons@ucsd.edu)

**Background**

This presentation describes the development of the Leadership and Organizational Change for Implementation (LOCI) strategy [1], subsequent adaptation, and preliminary data for implementing Motivational Interviewing (MI) in substance abuse treatment settings [2]. LOCI is an implementation strategy developed to align higher level organizational strategies with first-level leader development to create a strategic organizational climate to support implementation and sustainment of evidence-based practices (EBPs) [3]. Adaptations to the general design of LOCI that have occurred over time, as well as adaptations built into LOCI to tailor the strategy to particular settings will be described. LOCI provides a general structure, curricula, and process for leading implementation, allowing for flexibility so that leaders across levels can prioritize issues most relevant at a given time for a given context. We also describe mechanisms of change central to LOCI.

**Materials and Methods**

The current study involves 3 cohorts of 20 clinics in which clinics are being randomized to LOCI vs. webinar control conditions. For this presentation we utilize data from the first cohort 19 clinics and quantitatively examine differences in implementation leadership and climate by condition. Qualitative data were collected from and analyzed to identify factors influencing EBP implementation. Fourteen leaders across intervention and control conditions responded to an online survey and provided rankings (order of importance) and ratings of factors affecting EBP implementation. These data were supplemented with qualitative interviews.

**Results**

Preliminary results from the first cohort demonstrated that LOCI, compared to the control condition, showed significant improvements in implementation leadership and implementation climate. Qualitative analyses showed that compared to control leaders, LOCI leaders were more focused on staff competency and overcoming resistance. Time, turnover, and the influence of external contracts emerged as key themes.

**Conclusions**

The LOCI implementation strategy was designed to improve general and implementation leadership, subsequent implementation climate, and provider implementation behaviors including the adoption and use of EBP with fidelity. The ultimate goal is to improve client engagement in services and patient outcomes. Preliminary results suggest that LOCI can improve the context for implementation or EBPs, and that LOCI can help to focus leaders’ attention on improving implementation.

**Trial Registration:** ClinicalTrials.gov NCT03042832

**References**

1. Aarons GA, Ehrhart MG, Farahnak LR, Hurlburt MS. Leadership and organizational change for implementation (LOCI): a randomized mixed method pilot study of a leadership and organization development intervention for evidence-based practice implementation. Implement Sci. 2015;10:11

2. Aarons GA, Ehrhart MG, Moullin JC, Torres EM, Green AE. Testing the Leadership and Organizational Change for Implementation (LOCI) intervention in substance abuse treatment: A cluster randomized trial study protocol. Implement Sci. 2017;12:29.

3. Aarons GA, Ehrhart MG, Farahnak LR, Sklar M. Aligning leadership across systems and organizations to develop a strategic climate for evidence-based practice implementation. Annu Rev Public Health. 2014; 35:255-274.

## A13 Testing a multi-level implementation strategy for two evidence-based autism interventions

### Lauren Brookman-Frazee^1,2^, Aubyn Stahmer^3^, Allison Jobin^1,2^, Kristine Carandang^1,2^

#### ^1^Department of Psychiatry, University of California San Diego, San Diego, CA, USA; ^2^Child and Adolescent Services Research Center, University of California San Diego, San Diego, CA, USA; ^3^MIND Institute, University of California Davis, Davis, CA, USA

##### **Correspondence:** Lauren Brookman-Frazee (lbrookman@ucsd.edu)

**Background**

Children with ASD are a high priority population served in multiple public service systems. Evidence-based behavioral interventions are available [1-2], however, they are not routinely delivered in community care [3-6]. In response, our research groups used community-partnered approaches to adapt and test ASD interventions for routine delivery - “AIM HI” in children’s mental health [7] and “CPRT” in education [8]. We identified implementation leadership and climate as key implementation mechanisms in recent community effectiveness trials. We are conducting two, coordinated studies testing the effectiveness of an adapted version of the Leadership and Organizational Change for implementation (LOCI) strategy [9] as part of an implementation package [10]. This presentation describes the application of LOCI in two ASD services contexts for two EBIs.

**Materials and Methods**

The TEAMS project includes two linked randomized Hybrid Type 3 implementation trials to test two implementation strategies when paired with AIM HI or CPRT and examine mechanisms of these strategies, including implementation leadership and climate. The TEAMS Leadership Institute (TLI) applies LOCI as follows: (1) uses the LOCI components linked to mechanisms identified in the AIM HI and CPRT community effectiveness trials (i.e. implementation leadership and climate modules); (2) targets executive and mid-level leaders required to coordinate implementation; and (3) targets implementation of specific ASD interventions. We present process data on TLI implementation and initial themes from qualitative interviews to examine leader perceptions of the utility of TLI components and their impact on EBI implementation.

**Results**

To date, TLI has been conducted in 18 programs/districts in three California counties including 18 workshops and 152 coaching calls. Preliminary themes from interviews with 6 leaders who completed TLI indicate that TLI is feasible and useful to (1) convey to staff the importance of systematically planning EBI implementation, and (2) to maintain leader motivation and focus on executing strategic initiatives around AIM HI and CPRT amidst competing demands.

**Conclusions**

Preliminary data indicate the TEAMS application of LOCI is feasible and perceived as effective in facilitating the implementation of two ASD interventions. Future analyses will examine the impact of LOCI on targeted mechanisms – implementation leadership and climate.

**Trial Registration:** ClinicalTrials.gov NCT03380078

**References**

1. National Autism Center. National Standards Project, Phase 2. 2015. http://www.nationalautismcenter.org/national-standards-project/phase-2/.

2. Wong C, Odom SL, Hume KA, Cox AW, Fettig A, Kucharczyk S, Brock ME, Plavnick JB, Fleury VP, Schultz TR. Evidence-based practices for children, youth, and young adults with autism spectrum disorder: a comprehensive review. J Autism Dev Disord. 2015;45(7):1951–1966.

3. Brookman-Frazee L, Baker-Ericzén M, Stadnick N, Taylor R. Parent perspectives on community mental health services for children with autism spectrum disorders. J Child Fam Stud. 2012;21(4):533–544.

4. Brookman-Frazee L, Drahota A, Stadnick N, Palinkas LA. Therapist perspectives on community mental health services for children with autism spectrum disorders. Adm Policy Ment Health. 2012;39(5):365-373.

5. Brookman-Frazee L, Taylor R, Garland AF. Characterizing community-based mental health services for children with autism spectrum disorders and disruptive behavior problems. J Autism Dev Disord. 2010; 40(10):1188–201.

6. Stahmer AC, Collings NM, Palinkas LA. Early intervention practices for children with autism: descriptions from community providers. Focus Autism Other Dev Disabil, 2005;20(2):66-79.

7. Brookman-Frazee L, Roesch S, Chelbowski C, Baker-Ericzen, Ganger W. Effectiveness of training therapists to deliver an individualized mental health intervention for children with ASD in publicly funded mental health services: a cluster randomized clinical trial. JAMA Psychiatry. 2019;76(6):574-583.

8. Suhrheinrich J, Rieth SR, Dickson KS, Roesch S, Stahmer AC. Classroom pivotal response teaching: Teacher training outcomes of a community efficacy trial. Teach Educ Spec Educ. 2019. https://doi.org/10.1177/0888406419850876

9. Aarons GA, Ehrhart MG, Moullin JC, Torres EM, Green AE. Testing the Leadership and Organizational Change for Implementation (LOCI) Intervention in substance abuse treatment: a cluster randomized trial study protocol. Implement Sci. 2017;12(1):29.

10. Brookman-Frazee L. Stahmer AC. Effectiveness of a multi-level implementation strategy for ASD interventions: study protocol for two linked cluster randomized trials. Implement Sci. 2018;13(66).

## A14 Translation and adaptation of LOCI for implementation of evidence-based treatment for PTSD in Norwegian child and adult mental health care services

### Erlend Høen Laukvik, Ane-Marthe Solheim Skar, Karina M. Egeland

#### Norwegian Centre for Violence and Traumatic Stress Studies, Oslo, Norway

##### **Correspondence:** Erlend Høen Laukvik (e.h.laukvik@nkvts.no)

**Background**

The Norwegian Centre for Violence and Traumatic Stress Studies (NKVTS) is commissioned by the Ministry of Health and Care Services to implement evidence-based treatment for post-traumatic stress disorder (PTSD) in both child and adult mental health care services. As part of this effort, the Leadership and Organizational Change for Implementation (LOCI) [1] was translated and adapted for the implementation of trauma treatment in Norwegian health trusts. The aim of the project is to evaluate the effectiveness of LOCI in supporting the implementation of evidence-based treatment for PTSD in Norwegian specialized mental health clinics [2]. The presentation will identify the implementation determinants, targets, and mechanisms being examined.

**Materials and Methods**

The study is a Type III scale-out project [3]. Several a-priori adaptations were made, including translation of the LOCI materials into Norwegian, and tailoring of the LOCI fidelity tool. The study design is a stepped wedge cluster randomized trial with random and sequential enrollment of clinics into three cohorts, with crossover of clusters from control conditions to active intervention conditions based on time intervals. Executives, clinic leaders, and therapists complete surveys assessing leadership and implementation climate at baseline, 4, 8, 12, 16, and 20 months. At baseline, all therapists at the participating clinics were trained in trauma screening and a sub sample in the treatment models for PTSD (TF-CBT, EMDR, CT-PTSD), and units were randomly assigned to one of three cohorts. In addition, the strategy uses the 360 degrees assessments to inform subsequent work on tailored leadership and climate development plans to enhance implementation. Therapy sessions are audio or video recorded and scored for fidelity. Patients complete surveys assessing symptom development during the therapy process.

**Results**

Consistent with the LOCI theoretical model, assessment of mechanisms will examine the effects of leadership on EBP fidelity through its effect on implementation climate.

**Conclusions**

This study will provide knowledge about the effect of the LOCI program within a Norwegian context. As such, the results might inform evidence-supported implementation strategies that could help sustain national-wide implementation of evidence-based trauma treatment and increase the quality and effectiveness of Norwegian health services.

**References**

1. Aarons GA, Ehrhart MG, Moullin JC, Torres EM, Green AE. Testing the Leadership and Organizational Change for Implementation (LOCI) Intervention in substance abuse treatment: a cluster randomized trial study protocol. Implement Sci. 2017;12(1):29.

2. Egeland KM, Skar AMS, Endsjø M, Laukvik EH, Bækkelund H, Babaii A, Granly LB, Husebø GK, Borge RH, Ehrhart MG, Sklar M. Testing the leadership and organizational change for implementation (LOCI) intervention in Norwegian mental health clinics: a stepped-wedge cluster randomized design study protocol. Implement Sci. 2019;14(1):28.

3. Aarons GA, Sklar M, Mustanski B, Benbow N, Brown CH. “Scaling-out” evidence-based interventions to new populations or new health care delivery systems. Implement Sci. 2017;12(1):111.

## A15 Making sense of context: a systematic review

### Lisa Rogers, Aoife DeBrún, Eilish McAuliffe

#### School of Nursing, Midwifery and Health Systems, University College Dublin, Dublin, Ireland

##### **Correspondence:** Lisa Rogers (lisa.rogers@ucdconnect.ie)

**Background**

The uptake of evidence-based healthcare interventions is challenging, with, on average, a 17-year time gap between the generation of evidence and implementation of interventions into routine practice [1]. Although contextual factors such as culture are strong influences for successful implementation [2], context remains a poorly understood construct, with a lack of consensus regarding how it should be defined and accounted for within research [3]. A systematic review was conducted to address this issue by providing an insight into how context is defined and assessed within healthcare implementation science literature and develops a definition to better enable effective measurement of context.

**Materials and Methods**

The databases of PubMed, PsycInfo, CINAHL, and EMBASE were searched. English language empirical studies published in the previous 10 years were included if context was treated as a key component in implementing a healthcare initiative. Articles also needed to provide a definition and measure of context in order to be included. Results were synthesised using a narrative approach and supported using PRISMA guidelines for the conduct and reporting of systematic reviews.

**Results**

The searches yielded 3,021 records of which 64 met the eligibility criteria and were included. Studies used a variety of definitions. Some listed contextual factors (n=19) while others documented sub-elements of a framework that included context (n=19). Remaining articles provided a rich definition of an aspect of context (n=14) or context generally (n=12). Quantitative studies mostly employed the Alberta Context Tool while qualitative papers used a variety of frameworks with Promoting Action on Research Implementation in Health Services framework the most highly cited. Mixed methods studies used diverse approaches to assess context. Some used frameworks to inform the methods chosen while others used quantitative measures to inform qualitative data collection. Most papers (n=50) applied the chosen measure to all aspects of study design with a majority analysing context at an individual or level (n=51).

**Conclusions**

This review highlighted inconsistencies in defining and measuring context which supported the development of an enhanced understanding for this construct. By providing this consensus, improvements in implementation processes may result as greater understanding will help researchers appropriately account for context in research.

**References**

1. Bauer MS, Damschroder L, Hagedorn H, Smith J, Kilbourne AM. An introduction to implementation science for the non-specialist. BMC Psychol. 2015;3(1). doi:10.1186/s40359-015-0089-9

2. Proctor EK, Powell BJ, Baumann AA, Hamilton AM, Santens RL. Writing implementation research grant proposals: ten key ingredients. Implement Sci. 2012;7(96). http://www.implementationscience.com/content/7/1/96. Accessed May 15, 2018.

3. Nilsen P. Making sense of implementation theories, models, and frameworks. Implement Sci. 2015;10(1):53. doi:10.1186/s13012-015-0242-0

## A16 Implementing mental health assessment in a juvenile detention behavioral health unit: lessons learned from a community academic partnership

### Brittany Rudd^1^, Jacquelyn George^2^, Lauren Cliggitt^3^, Sean Snyder^4^, Mynesha Whyte^4^, Rinad S. Beidas^1^

#### ^1^Department of Psychiatry, University of Pennsylvania, Philadelphia, PA, USA; ^2^College of Public Health, Temple University, Philadelphia, PA, USA; ^3^Community Behavioral Health, Philadelphia, PA, USA; ^4^Hall Mercer Community Mental Health Center, University of Pennsylvania, Philadelphia, PA, USA

##### **Correspondence:** Brittany Rudd (ruddb@upenn.edu)

**Background**

The dual objectives of juvenile justice are to assure youth safety while in custody and to facilitate rehabilitation. Suicide is the second leading cause of death among 10-25 year olds [1], and is four times more likely among youth who enter juvenile justice (JJ) settings [2]. As youth in juvenile detention are at risk for engaging in suicidal behaviors, it is critical that behavioral health clinicians in juvenile detention settings conduct systematic evidence-based suicide risk, as well as general mental health, assessment. Recommendations for assessment in juvenile detention exist [3], but there is little guidance regarding how to implement them among behavioral health clinicians. The current presentation will describe a community academic (CAP) partnership, and the process that the partners underwent to implement a systemic protocol for assessing youth in a juvenile detention behavioral health unit.

**Materials and Methods**

A CAP was developed and a quality improvement procedure was utilized to develop and implement the assessment protocol.

**Results**

The CAP team included the service clinicians (Snyder and Whyte), and clinical supervisor (Cliggitt) in a behavioral health unit housed in a large, juvenile detention center in an urban city in Pennsylvania, as well as researchers from the University of Pennsylvania (Rudd, George, and Beidas). The development of the assessment protocol was an iterative process that occurred over eight months. The process started with a comprehensive review of current workflow and workflow infrastructure, including how youth were referred to the behavioral health unit and the information behavioral health unit staff had about youth prior to their intake. Iterative changes to workflow procedures were needed, including developing infrastructure to support assessment (e.g., developing report templates) during the behavioral health intake appointment. Finally, several assessment measures were piloted to determine fit.

**Conclusions**

The creation of a CAP was key to developing and implementing a comprehensive and feasible mental health and suicide assessment protocol. Lessons learned from the application of implementation science to the juvenile detention context from the joint perspectives of researcher (Rudd) and clinician (Synder) stakeholder perspectives will be presented.

**References**

1. Heron MP. Deaths: Leading causes for 2016. National Vital statistics reports. 2018;67(6).

2. Wasserman GA, McReynolds LS. Suicide risk at juvenile justice intake. Suicide Life Threat Behav. 2006;36(2):239-249.

3. Grisso T, Underwood LA. Screening and assessing mental health and substance use disorders among youth in the juvenile justice system. a resource guide for practitioners. US Department of Justice. 2004. https://www.ncjrs.gov/pdffiles1 /ojjdp/204956.pdf.

## A17 DIY implementation: lessons from a practitioner-led implementation of an evidence-based practice

### Sean Wright^1^, Sonia Combs^2^

#### ^1^Lutheran Community Services Northwest, Spokane, WA, USA; ^2^Cor Counseling and Wellness, Spokane, WA, USA

##### **Correspondence:** Sean Wright (swright@lcsnw.org)

**Background**

Reports of implementation efforts initiated at the practitioner level are uncommon. To address this gap, we describe the results of and lessons from an ongoing practitioner-led implementation of Acceptance and Commitment Therapy (ACT), an evidence-based practice, in a community mental health center team.

**Materials and Methods**

We used a variety of implementation strategies (mostly training) during an ongoing implementation of ACT. Initially, we conducted a mixed methods study of the facilitators and barriers to implementation, collecting qualitative and quantitative survey data anonymously at two time points, sampled from all clinical staff (N=39) at our agency. The survey measured attitudes, knowledge, experience, and acceptability of the EBP. We assessed the significance of changes in Likert ratings using the sign test [1]. We used thematic analysis to code qualitative data. Recently, penetration was measured by relative use of ACT in a one-month sample of progress notes and by the relative percentage of team members using ACT. Implementation strategies used were identified by retrospective review and coded in accordance with the Expert Recommendations for Implementing Change (ERIC) project [2] and classified into concept mapping clusters for ERIC strategies [3]. We created a timeline of implementation activities and identified key individuals who facilitated these activities.

**Results**

15 pairs of pre-post survey measures indicated that initial training was associated with increases in identification as an ACT therapist (Z=-2.12,p=0.035), perceived ability to demonstrate ACT (Z=-3.00,p=0.002), and a trend toward increased use of ACT (Z=-1.90,p=0.055). Qualitative analyses were consistent with the existing literature on facilitators and barriers to EBP adoption in community mental health. ACT use in a recent one-month window was evidenced with 7.9% of progress notes documenting use of ACT (baseline before implementation: 0%) and 32% of eligible clinicians documenting ACT use in progress notes (initial baseline: 0%). Evidence for use of 21 of the 73 ERIC implementation strategies was documented. The strategies are distributed across all 9 concept mapping clusters, with the Train and Educate Stakeholders cluster most represented (5 of 11 strategies). Three key individuals were identified.

**Conclusions**

Practitioner-led implementation is feasible. Implementation strategies can inform practitioner efforts.

**References**

1. Roberson PK, Shema SJ, Mundfrom DJ, Holmes TM. Analysis of paired Likert data: how to evaluate change and preference questions. Fam Med. 1995;27(10):671-675.

2. Powell BJ, Waltz TJ, Chinman MJ, Damschroder LJ, Smith JL, Matthieu MM, Proctor EK, Kirchner JE. A refined complication of implementation strategies: results from the Expert Recommendations for Implementing Change (ERIC) project. Implement Sci. 2015;10:21.

3. Waltz TJ, Powell BJ, Matthieu MM, Damschroder LJ, Chinman MJ, Smith JL, Proctor EK, Kirchner JE. Use of concept mapping to characterize relationships among implementation strategies and assess their feasibility and importance: results from the Expert Recommendations for Implementing Change (ERIC) study. Implement Sci. 2015;10:109.

## A18 Implementation of an educator participatory program for improving work environments on health and wellbeing: a mixed methods approach

### Lisa Sanetti, Alexandra Pierce, Michele Femc-Bagwell, Alicia Dugan

#### Neag School of Education University of Connecticut, Storrs, CT, USA

##### **Correspondence:** Lisa Sanetti (lisa.sanetti@uconn.edu)

**Background**

A chronic and increasing challenge to employee wellness in schools is teacher stress [1]. Teachers are tied with nurses as having the highest rates of daily stress among occupations [2]. Chronic high levels of teacher stress are associated with (a) increased rates of physical and psychological health problems, including anxiety, depression, cardiovascular disease, and poor sleep quality; (b) poor job performance, including absenteeism, negative interactions, poor relationships with students, and poor classroom management, and (c) poor student outcomes, including low rates of academic achievement, lower levels of social adjustment, and increased rates of problem behavior [1,3]. Further, chronic teacher stress is the primary factor associated with the high rate of teachers leaving the profession for reasons other than retirement, which has nearly doubled over the past 25 years, constituting the primary cause of teacher shortages nationwide [4]. A critical need to address teacher health and wellbeing exists, yet, on average, only 31.4% of schools offer workplace health and wellness promotion programs; most of these programs are top-down, one-size-fits-all approaches that are either ineffective or unsustainable [5].

**Materials and methods**

The purpose of this mixed methods study was to implement the Healthy Workplace Participatory Program (HWPP), an evidence-based approach that engages front-line employees (i.e., teachers) and supervisors (e.g., administrators) in a collaborative, iterative design of workplace health and wellness interventions [6].

**Results**

This participatory approach allows for (a) identification of health and wellness issues most salient to employees; (b) development of a wider range of interventions as employees are more aware of complex interactions between their work organization, workplace, and lifestyle; and (c) identification of potential intervention barriers and facilitators; (d) increased buy-in to problem definition and intervention design; and (e) establishment of a supportive organizational culture and processes for a self-correcting and sustainable health and wellness promotion program. The HWPP has been shown to effectively increased employee health and wellbeing in a wide range of worksites [6]; this is the first implementation effort in schools.

**Conclusions**

Results of focus groups as well as formative and summative data related to implementation and intervention processes, strategies, and outcomes across EPIS phases in two 3rd-5th grade elementary schools in the Northeast will be presented.

**References**

1. Flook L, Goldberg S, Pinger L, Bonus K, Davidson R. Mindfulness for teachers: a pilot study to assess effects on stress, burnout, and teaching efficacy. Mind Brain Educ. 2013;7(3):182-195.

2. Gallup. State of America’s Schools 2014. http://www.gallup.com/services/178769/state-america-schools-report.aspx . Accessed 22 July 2018.

3. Roeser RW, Skinner E, Beers J, Jennings PA. Mindfulness training and teachers’ professional development: an emerging area of research and practice. Child Dev Perspect. 2012;6(2):167-173.

4. Goldring R, Taie S, Riddles M. Teacher attrition and mobility: results from the 2012–13 teacher follow-up survey (NCES 2014-077). 2014. http://nces.ed.gov/pubsearch. Accessed 3 April 2016.

5. Naghieh A, Montgomery P, Bonell CP, Thompson M, Aber JL. Organisational interventions for improving wellbeing and reducing work-related stress in teachers. Cochrane Database Syst Rev. 2015;4:CD010306.

6. Robertson M, Henning R, Warren N, Dove-Steinkamp M, Tibirica L, Bizarro A, CPH-NEW Research Team. The intervention design and analysis scorecard: A planning tool for participatory design of integrated health and safety interventions in the workplace. J Occup Environ Med. 2013;55:S86-S88.

## A19 Assessing intermediary organization capacity for active implementation support: development and collaborative early usability appraisal of an intermediary organization capacity assessment tool

### Robin Jenkins, William Aldridge, Rebecca Roppolo

#### Frank Porter Graham Child Development Institute, University of North Carolina at Chapel Hill, Chapel Hill, NC, USA

##### **Correspondence:** Robin Jenkins (robin.jenkins@unc.edu)

**Background**

Many evidence-based practices (EBPs) rely on multiple dissemination supports to assist scaling to achieve population benefits. Intermediary organizations (IOs) are often key in leveraging critical functions in the overall support system to enhance diffusion strategies [1-3]. Despite the prevalence of intermediaries as important accelerators of evidence-based practices, little is known about which IO strategies are most effective in ensuring implementation and scaling success or how strategies link to existing IO capacities [3]. Further, there is a dearth of information regarding IO capacity assessments relative to their capabilities to effectively diffuse EBP’s or to perform active implementation support.

**Materials and Methods**

Working with a public-private partnership (state government agencies and private funders) to scale Triple P statewide in North and South Carolina, both states have selected IOs to enhance statewide Triple P implementation. To assess IO capacity for planning and delivering implementation supports, a tool was needed to establish baseline capacity and to guide support planning. The Intermediary Organization Capacity Assessment (IOCA) was developed as an IO capacity assessment tool aligned with Mettrick et al.’s five observed functions.

**Results**

Early capacity assessment data and collaborative qualitative usability feedback from partners indicate that the tool is demonstrating practical utility toward capacity assessments and planning for ongoing support. The IOCA appears to align well with Mettrick et al.’s functional groups of IO support activities. Early feedback suggests that use of the tool also aids in transferring knowledge of implementation science-informed strategies to IO partners in functionally informative, practical ways.

**Conclusions**

The IOCA is demonstrating good alignment with known classes of IO support functions. It is also providing practical usability relative to understanding baseline IO capacity to deliver ongoing supports for scaling of EBPs. IOs that have experience with the tool report improved understanding of implementation science-informed strategies and tools that can better guide them in their support activities.

**References**

1. Franks RP, Bory CT. Who supports the successful implementation and sustainability of evidence-based practices? Defining and understanding the roles of intermediary and purveyor organizations. New Dir Child Adolesc Dev. 2015; 149:41-56. doi:10.1002/cad.20112

2. Mettrick J, Harburger DS, Kanary PJ., Lieman RB, Zabel,M. Building cross-system implementation centers: a roadmap for state and local child serving agencies in developing Centers of Excellence (COE). Baltimore, MD: The Institute for Innovation & Implementation, University of Maryland. 2015. https://archive.hshsl.umaryland.edu/handle/10713/7379.

3. Proctor E, Hooley C, Morse A, McCrary S, Kim H, Kohl PL. Intermediary/purveyor organizations for evidence-based interventions in the US child mental health: characteristics and implementation strategies. Implement Sci. 2019;14(1):3. doi:10.1186/s13012-018-0845-3

## A20 A tailored implementation approach to improving PTSD care in military treatment facilities: integrating practice-based knowledge and implementation science

### David Riggs^1^, Katherine Dondanville^2^, Elisa Borah^3^, Craig Rosen^4^

#### ^1^Center for Deployment Psychology, Uniformed Services University, Bethesda, MD, USA; ^2^Department of Psychiatry and Behavioral Sciences, University of Texas Health Science Center at San Antonio, San Antonio, TX, USA; ^3^Department of Psychiatry, University of Texas at Austin, Austin, TX, USA; ^4^National Center for PTSD, VA Palo Alto Health Care System, Palo Alto, CA, USA

##### **Correspondence:** David Riggs (driggs@deploymentpsych.org)

**Background**

The panel will discuss integration of practical lessons learned from clinicians and administrators with principles of implementation science to develop a program to increase use of evidence-based psychotherapy (EBP) for PTSD in military treatment facilities (MTFs). Despite efforts to train military providers in EBPs, only a minority of service members receive them [1]. Implementation barriers likely vary across MTFs, which differ in size, resources, command structure, and implementation climate. Increased use of EBPs likely requires a tailored approach that aligns implementation strategies to local conditions [2].

**Materials and Methods**

The Targeted Assessment and Context-Tailored Implementation of Change Strategies (TACTICS) program combines needs assessment, a rubric for aligning implementation strategies to local barriers and facilitators, and external facilitation to help clinics enact a collaboratively developed implementation plan. Through experience working with MTFs, the Center for Deployment Psychology (intermediaries) identified common implementation barriers and potential context-specific strategies to address them. These were augmented with additional relevant strategies from the Expert Recommendations for Implementing Change project [3] and input from experienced implementers. Barriers and facilitators in the resulting TACTICS rubric were then mapped backed to domains of the Consolidated Framework for Implementation Research [4].

After getting leadership approval and identifying a site champion, the five-month TACTICS process involves conducting needs assessment interviews with relevant staff and reviewing clinic data to identify barriers and facilitators, using the TACTICS rubric to identify potential change targets and strategies to address local conditions, and meeting with staff to develop the implementation plan. This is followed by weekly coaching calls (external facilitation) to support the champion in enacting changes to increase use of evidence-based psychotherapy.

**Results**

TACTICS rubric development is completed and is being pilot tested at one site. After this development phase, TACTICS will be tested in a stepped-wedge randomized trial in eight military treatment facilities.

**Conclusions**

Development of the TACTICS program was informed by intermediaries’ practical knowledge from military clinicians, implementation experience, and by implementation science frameworks. If successful, TACTICS provides a barrier-to-solution tailoring framework informed by implementation practitioners, researchers, and local staff.

**References**

1. Hepner KA, Roth CP, Sloss EM, Paddock SM, Iyiewuare PO, Timmer MJ, Pincus HA. Quality of care for PTSD and depression in the military health system: Final report. RAND Health Q. 2018;7(3):3.

2. Powell BJ, Beidas RS, Lewis CC, Aarons GA, McMillen JC, Proctor EK, Mandell DS. Methods to improve the selection and tailoring of implementation strategies. J Behav Health Serv Res. 2017;44(2):177-194. doi:10.1007/s11414-015-9475-6.

3. Powell BJ, Waltz TJ, Chinman MJ, Damschroder LJ, Smith JL, Matthieu MM, Proctor EK, Kirchner JE. A refined compilation of implementation strategies: results from the Expert Recommendations for Implementing Change (ERIC) project. Implement Sci. 2015;10:21. doi:10.1186/s13012-015-0209-1.

4. Damschroder LJ, Aron DC, Keith RE, Kirsh SR, Alexander JA, Lowery, JC. Fostering implementation of health services research findings into practice: a consolidated framework for advancing implementation science. Implement Sci. 2009; 4:50. doi:10.1186/1748-5908-4-50.

## A21 Rubber meets the road: how one intermediary organization uses implementation science to inform training and implementation supports for a large state system of behavioral health

### Sapana Patel^1,2^, Lisa Dixon^1,2^

#### ^1^Department of Psychiatry, Columbia University, New York, NY, USA; ^2^The New York State Psychiatric Institute, New York, NY, USA

##### **Correspondence:** Sapana Patel (sapana.patel@nyspi.columbia.edu)

**Background**

At federal, state, and local levels, stakeholders are focused on developing, disseminating, and implementing evidence-based practices (EBPs). Intermediary organizations are entities that help agencies or systems develop, implement, and sustain evidence-based practices [1]. Little is known about how implementation science frameworks, strategies and tools that are used by intermediary organizations charged with scaling evidence-based practices for a large state system of behavioral health.

**Materials and Methods**

The Center for Practice Innovations (CPI), at Columbia Psychiatry and the New York State Psychiatric Institute, is an intermediary organization whose mission is to support the New York State Office of Mental Health in the use of EBPs throughout community-based mental health agencies in New York State. CPI’s role includes: (a) public awareness, and education; (b) scalable dissemination of training in EBPs; (c) implementation support through learning collaboratives; (d) quality improvement; and (e) outcome evaluation. We will describe empirical approaches to the development, dissemination of scalable training and implementation support for a range of initiatives at CPI.

**Results**

Grounded in the Consolidated Framework for Implementation Research [2], we will present on the CPI practice change model and how the CFIR assists in planning for post-training implementation support and the identification of barriers and facilitators to implementation. Using the published taxonomy, Expert Recommendations for Implementing Change [3], we will describe a range of implementation strategies (e.g., instructional design methods, user-centered design, stakeholder engagement) that inform the development of scalable online training and identify targets for post-training implementation activities. Lastly, we will provide examples of online training evaluation [4] and challenges faced in reporting on the impact of implementation strategies [5-6] within a large system of behavioral healthcare.

**Conclusions**

Although a balancing act, it is possible for intermediary organizations to remain flexible, efficient, and rapid in response to the mission of real-world dissemination and implementation of EBPs and use empirically-driven distance and E-learning and implementation science approaches. There are opportunities for mutual learning, synergy and collaboration to advance the field of implementation science for researchers and practitioners.

**References**

1. Franks RP, Bory CT. Who supports the successful implementation and sustainability of evidence-based practices? Defining and understanding the roles of intermediary and purveyor organizations. New Dir Child Adolesc Dev. 2015; 149:41-56. doi:10.1002/cad.20112

2. Damschroder LJ, Aron DC, Keith RE, Kirsh SR, Alexander JA, Lowery JC. Fostering implementation of health services research findings into practice: a consolidated framework for advancing implementation science. Implement Sci. 2009; 4:50.

3. Powell BJ, Waltz TJ, Chinman MJ, Damschroder LJ, Smith JL, Matthieu MM, Proctor EK, Kirchner JE. A refined compilation of implementation strategies: results from the Expert Recommendations for Implementing Change (ERIC) project. Implement Sci. 2015;10:21.

4. Kirkpatrick DKJ, Kirkpatrick JD. Evaluating training programs: the four levels (3rd Edition). San Francisco, CA: Berrett-koehler Publishers; 2019.

5. Proctor EK, Powell BJ, McMillen JC. Implementation strategies: recommendations for specifying and reporting. Implement Sci. 2013; 8:139.

6. Gold R, Bunce AE, Cohen DJ, Hollombe C, Nelson CA, Proctor EK, Pope JA, DeVoe JE. Reporting on the strategies needed to implement proven interventions: an example from a “real-world” cross-setting implementation study. Mayo Clin Proc. 2016;91(8):1074-83.

## A22 Utilization of train-the-trainer programs to support the sustainability of evidence-based trauma-informed interventions: the perspectives of model developers, trainers, and intermediary agencies within the National Child Traumatic Stress Network

### Shannon Chaplo^1^, George Ake^1,2^, Lisa Amaya-Jackson^1,2^, Byron J. Powell^3^, Ginny Sprang^4^

#### ^1^Department of Psychiatry and Behavioral Science, Duke University Medical Center, Durham, NC, USA; ^2^National Center for Child Traumatic Stress, Washington, DC, USA; ^3^Brown School, Washington University in St. Louis, St. Louis, MO, USA; ^4^Department of Psychiatry, University of Kentucky, Lexington, KY, USA

##### **Correspondence:** Shannon Chaplo (shannon.chaplo@duke.edu)

**Background**

Train-the-Trainer programs (TTTs) refer to “a program or course where individuals in a specific field receive training in a given subject and instruction on how to train, monitor, and supervise other individuals in the approach [1].” TTTs are implementation strategies intended to increase the reach and sustainment of evidence-based interventions in mental health agencies, and address other challenges such as therapist attrition and developer succession planning [2-3]. Several trauma-informed interventions for children have TTTs; however, there is no standardized protocol for developing or delivering TTTs. As the need to disseminate and sustain trauma-informed interventions grows, the need to develop guidelines for TTTs becomes imperative. The objective of this project is to better understand the state of TTTs for trauma-informed interventions utilized by members and consumers of the National Child Traumatic Stress Network (NCTSN).

**Materials and Method**

Duke University study staff partnered with members of the NCTSN Implementation Advisory Committee to develop a survey exploring TTTs in the NCTSN. The survey was designed to gather the perspective of developers of treatments, practices, and curricula; professionals that become trainers through TTTs; and agency training directors that serve as consumers of TTTs. Developers will answer a series of questions about the development and implementation of their TTT program. Trainers and agency directors will be asked about their experience participating in a TTT program. All respondents will be asked about the components of their TTTs, barriers to using or developing TTTs, and facilitators of developing or using TTTs.

**Results**

No results are currently available. The study has secured IRB approval and the survey will launch in April 2019. Results will be analyzed in summer 2019.

**Conclusions**

Surveying each of these audiences will help us to better understand the varying components of TTTs, and barriers and facilitators of their use within the NCTSN. We plan to use the survey results for training purposes and resource development to enhance the use of TTTs within the NCTSN (and in other relevant settings) to implement and sustain trauma-informed interventions.

**References**

1. Pearce J, Mann, MK, Jones, C, van Buschbach, S, Olff, M, Bisson, JI. The most effective way of delivering a Train‐the‐Trainers program: a systematic review. J Contin Educ Health Prof. 2012; 32:215-226.

2. Bero L, Grilli R, Grimshaw J, Harvey E, Oxman A, Thomson M. Closing the gap between research and practice: an overview of systematic reviews of interventions to promote implementation of research findings by health care professionals. BMJ. 1998;317:465–468.

3. Yarber L, Brownson CA, Jacob RR, Baker EA, Jones E, Baumann C, Deshpande AD, Gillespie KN, Scharff DP, Brownson RC. Evaluating a train-the-trainer approach for improving capacity for evidence-based decision making in public health. BMC Health Serv Res. 2015;15:547.

## A23 Nudge yourself: stakeholder design of implementation strategies that leverage insights from behavioral economics

### Briana S. Last, Courtney Benjamin Wolk, Rinad S. Beidas

#### Department of Psychiatry, University of Pennsylvania, Philadelphia, PA, USA

##### **Correspondence:** Briana S. Last (brishiri@sas.upenn.edu)

**Background**

Though several evidence-based practices (EBPs) exist for depression, only a fraction of individuals receive treatment. One major challenge to treatment is the identification of individuals in need of care. In response to this need, health care systems such as the University of Pennsylvania (Penn) have mandated universal screening of depression in primary care settings based on evidence. However, adherence to the mandate at Penn is much lower than anticipated (only 40% of eligible patients are screened). In our study, we partnered with front line clinicians and staff to increase depression screening at Penn using innovative approaches from implementation science and behavioral economics.

**Materials and Methods**

This project will engage in a participatory process with key stakeholders to design implementation strategies to increase universal depression screening in primary care. In particular, we fill focus on designing a subset of implementation strategies—nudges, as they are called in behavioral economics [1]—that alter the choice architecture, or the way options are presented to optimize choices. First, we began by conducting an innovation tournament, a crowdsourcing technique [2], with physicians, nurses, medical assistants, behavioral health clinicians, and front-desk stuff currently involved in administering the electronic depression screener, the two-item Patient Health Questionnaire (PHQ-2) at their practices. The tournament will generate ideas on how to increase the implementation of the PHQ-2. Next, we will fine-tune the ideas generated from the innovation tournament with a team of stakeholders, behavioral economists, and implementation scientists using behavioral economic theory [3]. The product of this project will be a toolkit of implementation strategies, that are theoretically motivated and acceptable to a range of relevant stakeholders, a subset of which will be later refined through a more rigorous piloting process.

**Results**

The innovation tournament closes mid-April, 2019. Ideas from the tournament will be refined into a toolkit of implementation strategies by September 2019.

**Conclusions**

Our study responds to the need for interdisciplinary, theoretically informed, and participatory approaches to designing implementation strategies. The results from our work will shed light on whether these approaches show promise.

**References**

1. Thaler RH, Sunstein CR. Nudge: Improving decisions about health, wealth, and happiness. Revised and Expanded Edition. New York, NY: Penguin Books; 2009.

2. Terwiesch C, Mehta SJ, Volpp KG. Innovating in health delivery: the Penn medicine innovation tournament. Healthc. 2013;1(1-2):37–41.

3. Eldredge LKB, Markham CM, Ruiter RA, Kok G, Fernandez ME, Parcel GS. Planning health promotion programs: an intervention mapping approach. San Francisco: John Wiley & Sons; 2016.

## A24 Using stakeholder values to promote implementation of an evidence-based mHealth intervention for addiction treatment in primary care

### Correspondence: Andrew Quanbeck (arquanbe@wisc.edu)

#### College of Engineering, University of Wisconsin – Madison, Madison, WI, USA

**Background**

The majority of evidence-based practices do not find their way into clinical use, including mobile health (mHealth) technologies. This presentation describes a novel decision-framing model that gives implementers a method for eliciting the perceived gains and losses that different stakeholder groups trade off when faced with the decision of whether to adopt an evidence-based mHealth intervention.

**Materials and Methods**

The decision-framing model integrates insights from behavioral economics [1,2] and game theory [3]. The approach was applied retrospectively in a parent implementation research trial that introduced an mHealth system to 268 patients in three U.S. clinics offering primary and behavioral healthcare services. The mHealth system, called Seva, supports patients with addiction. Individual and group interviews were conducted to elicit stakeholder considerations from 23 clinic staff members and 6 patients who were involved in implementing Seva. Considerations were used to construct “decision frames” that trade off the perceived value of adopting Seva vs. maintaining the status quo from each stakeholder group’s perspective. The face validity of the decision-framing model was assessed by soliciting feedback from the stakeholders whose input was used to build it.

**Results**

Primary implementation considerations were identified for each stakeholder group. Clinic managers perceived the greatest potential gain to be providing better care for patients, and the greatest potential loss to be cost, expressed in terms of staff time, sustainability, and opportunity cost. All clinical staff considered time their foremost consideration—primarily in negative terms (e.g., cognitive burden associated with learning a new system) but potentially positively (e.g., if Seva could automate functions done manually). Patients considered safety (anonymity, privacy, and coming from a trusted source) to be paramount. When considerations were compiled into decision frames that traded off the gains and losses associated with adopting Seva, only one stakeholder group—patients—expressed a positive overall value, and these were the stakeholders who used Seva most.

**Conclusions**

This paper presents a systematic method of inquiry to elicit stakeholders’ considerations when deciding to adopt a new technology. Stakeholder considerations may be used to adapt mHealth interventions and tailor implementation, potentially increasing the likelihood of implementation success for evidence-based practices and technologies.

**References**

1. Tversky A, Kahneman D. The framing of decisions and the psychology of choice. Science 1981; 211(4481):453-8.

2. Kahneman D, Lovallo D, Sibony O. Before you make that big decision… Harv Bus Rev 2011;89(6):50-60.

3. Myerson RB. Game theory: analysis of conflict. Cambridge: Harvard University Press; 1991.

## A25 Applying insights from participatory design to design implementation strategies

### Rinad S. Beidas^1^, Nathaniel Williams^2^, Rebecca Stewart^1^

#### ^1^Department of Psychiatry, University of Pennsylvania, Philadelphia, PA, USA; ^2^School of Social Work, Boise State University, Boise, ID, USA

##### **Correspondence:** Rinad S. Beidas (rbeidas@upenn.edu)

**Background**

Public behavioral health systems have increasingly invested in the implementation of evidence-based practices (EBPs), including Philadelphia’s Department of Behavioral Health. Training and technical assistance continue to be the most commonly used strategies to increase use of EBPs, despite findings that organizational barriers matter. Few organizational implementation strategies exist and little is known about how to best design organizational strategies to increase implementation of EBPs using participatory design approaches. We partnered with front line clinicians to develop organizational implementation strategies to improve EBP implementation in community mental health clinics.

**Materials and Methods**

We engaged in a three-step process to design organizational implementation strategies. First, we launched an innovation tournament to engage clinicians employed within the Philadelphia public behavioral health system to crowd-source how their organizations can support them to use EBPs. We held a community-facing event during which the 6 clinicians who submitted winning ideas presented their ideas to 85 attendees representing a range of stakeholders. Second, we worked with behavioral scientists to refine the ideas to optimize their effectiveness. Third, we launched a system-wide survey targeting approximately 300 stakeholders to elicit preferences for the clinician generated organizational implementation strategies.

**Results**

We report on the outcomes of the innovation tournament and system-wide survey. A total of 65 ideas were submitted in the innovation tournament by 55 participants representing 38 organizations. The most common categories of ideas pertained to training (42%), compensation (26%), clinician support tools (22%), and EBP-focused supervision (17%). Using an innovation tournament to generate ideas for implementation strategies was feasible and acceptable as demonstrated by the high levels of engagement. However, we also identified barriers (e.g., ensuring that the stakeholder voice was adequately represented throughout all stages). The system-wide survey will be launched in March, 2019; and will close April, 2019.

**Conclusions**

The approach that we took in designing implementation strategies is promising. Research is needed to test whether strategies developed via these methods are more effective than strategies developed through competing approaches.

## A26 Leveraging normative pressure to increase data collection among therapists working with children with autism

### David S. Mandell, Heather Nuske, Emily Becker-Haimes

#### Department of Psychiatry, University of Pennsylvania, Philadelphia, PA, USA

##### **Correspondence:** Emily Becker-Haimes (embecker@upenn.edu)

**Background**

Evidence-based practices for children with autism generally follow the principles of applied behavior analysis, which require frequent, systematic data collection. In Philadelphia, as in many systems, children with autism often are accompanied by a one-to-one aide, who is responsible for collecting these data as part of implementing a treatment plan. Direct observations and interviews with these aides and their supervisors confirm that aides rarely collect data in a rigorous manner. These aides often work in isolation and rarely receive consistent supervision, which may lead to the perception that data collection is not expected of them, nor do people in their position collect data in this manner. We use participatory methods combined with innovative methods borrowed from industry that incorporate the principles of behavioral economics to design implementation strategies to increase aides’ data collection.

**Materials and Methods**

We partnered with five community agencies and used time-and-motion study methods, an observational technique drawn from scientific management, to understand how one-to-one aides collect data. This process involved querying aides about the decisions they made regarding data collection in the moment. We used participatory design strategies, including an innovation tournament—a method to crowdsource strategy ideas from stakeholders—and a rapid-cycle approach—a method that involves iterative testing and refining implementation strategies—to increase one-to-one aides’ data collection. We applied theoretical principles from behavioral economics to refine the implementation strategies generated from the innovation tournament and to test them using our rapid cycling approach.

**Results**

Data collection is ongoing. Here we present on the time-and-motion studies and results of our innovation tournament. We provide a framework for the rapid-cycle process that is ongoing at the time of this presentation.

**Conclusions**

This method of data collection in the service of identifying implementation strategies and rapidly testing them holds promise.

## A27 Applications of standardized patient methodology to measure fidelity in an implementation trial of the teen marijuana check-up

### Bryan Hartzler, Denise Walker, Aaron Lyon, Kevin King, Lauren Matthews, Tara Ogilvie, Devon Bushnell, Katie Wicklander

#### University of Washington, Seattle, WA, USA

##### **Correspondence:** Bryan Hartzler (hartzb@uw.edu)

**Background**

A cornerstone of medical education, standardized patients (SPs) are increasingly incorporated in implementation trials for behavior therapies as a highly valid, advantageous approach to fidelity measurement [1]. Such methodological benefits extend to SP involvement in behavioral rehearsal activities often included to support therapy training processes [2]. An ongoing implementation trial examining the Teen Marijuana Check-Up (TMCU) [3], a school-based adaptation of motivational enhancement therapy, incorporates SPs for both purposes [4].

**Materials and Methods**

In this trial, a set of SPs portray marijuana-using adolescent characters in dyadic interactions with participating school-based staff. As components of TMCU training, two SP-involved training cases—each offering consequence-free opportunities for staff to receive performance-based trainer feedback—supplemented an initial workshop. As components of pre- and post-training outcome assessments, two more SP interactions provided behavioral outcome measures. All four SP interactions involved travel to staff workplaces to record a simulated TMCU session, later scored for the following fidelity indices: ratio of reflective listening statements to questions (R:Q), percentage of ‘open-ended’ questions (%OQ), and percentage of ‘complex’ reflective listening statements (%CR).

**Results**

Recruited from seven high schools, twenty staff completed all four SP interactions. Pre-training SP interactions revealed variable staff performances, with two staff members achieving a TMCU proficiency standard by exceeding benchmarks for all three fidelity indices. In SP-involved training cases, this proficiency standard was achieved by eight staff in an initial case, and by six staff in a more challenging latter case. In eventual post-training SP interactions, five staff met the TMCU proficiency standard. As for mean training impact, Cohen’s d effect sizes suggest small-to-medium effects on R:Q (d=.20), %CR (d=.36), and %OQ (d=.43), and documented expected needs for subsequent support of TMCU implementation via purveyor coaching as a targeted form of post-training technical assistance.

**Conclusions**

This trial—wherein SP methodology further extends to monitoring of TMCU fidelity in biannual assessments for two years after training—includes SP roles in outcome assessment and training. Fidelity data from the collective SP interactions evidence sensitivity to hypothesized changes in staff learning, further supporting the use of SPs as means to measure and monitor fidelity in trials examining behavior therapy implementation.

**Trial Registration:** ClinicalTrials.gov NCT03111667

**References**

1. Imel ZE, Baldwin SA, Baer JS, Hartzler B, Dunn C, Rosengren DB, Atkins DC. Evaluating therapist adherence in motivational interviewing by comparing performance with standardized and real patients. J Consult Clin Psychol. 2014; 82(3):472-481.

2. Beidas RS, Cross W, Dorsey S. Show me, don’t tell me: behavioral rehearsal as a training and analogue fidelity tool. Cogn Behav Pract. 2014;21(1):1-11.

3. Walker DD, Stephens RS, Roffman R, Towe S, DeMarce J, Lozano B, Berg B. Randomized controlled trial of motivational enhancement therapy with nontreatment-seeking adolescent cannabis users: a further test of the teen marijuana check-up. Psych Addict Behav. 2011;25(3):474-484.

4. Hartzler B, Lyon AR, Walker DD, Matthews L, King KM, McCollister KE. Implementing the teen marijuana check-up in schools—a study protocol. Implement Sci. 2017;12(103).

## A28 A hybrid type 1 design to facilitate rapid testing and translation of an emergency department-based opioid use disorder intervention through an academic-state government partnership

### Dennis Watson^1^, Alan McGuire^2,3^, Rebecca Buhner^4^, Krista Brucker^5^

#### ^1^College of Medicine, University of Illinois at Chicago, Chicago, IL, USA; ^2^Richard L. Roudebush VAMC, Indianapolis, IN, USA; ^3^Department of Psychology, Indiana University, Indianapolis, IN, USA; ^4^Indiana Division of Mental Health and Addiction, Indianapolis, IN, USA; ^5^Emergency Medicine, Indiana University School of Medicine, Indianapolis, IN, USA

##### **Correspondence:** Dennis Watson (dpwatson@uic.edu)

**Background**

The gravity of the opioid epidemic requires innovative and promising solutions that can be rapidly scaled [1]. Hybrid type 1 designs can speed the translation of such interventions by accomplishing the dual tasks of (a) establishing effectiveness of interventions as they are being rolled out under real-world conditions and (b) identifying determinants of implementation that can assist with planning of future scaling activities [2-3]. The current study aims to accomplish just such a task through the replication and testing of Project Planned Outreach, Intervention, Naloxone, and Treatment (POINT), an ED-based peer support intervention aimed at connecting people with opioid use disorder to medication assisted treatment (i.e., methadone-, buprenorphine-, or naltrexone-facilitated treatment). This study is funded by a unique federal mechanism that aims to improve rapid translation of research to practice through academic-state partnerships. In this presentation, we will provide an overview of our pragmatic hybrid design before focusing on results of the study’s 6-month pilot phase.

**Materials and Methods**

The researchers partnered with the Indiana Division of Mental Health and Addiction to carry out this study. Per the project’s funding mechanism, success of the pilot was to be determined by the achievement of 3 milestones, including our ability to successfully replicate the POINT intervention with 75% fidelity to previously identified critical components within a new implementation context.

**Results**

Overall implementation of the study protocols was successful, with only minor refinements to proposed procedures being required in light of challenges with (a) data access, (b) recruitment, and (c) identification of the expansion hospitals. All three milestones were reached, including 77% fidelity to the original POINT programs’ components. Challenges in implementing protocols and reaching milestones resulted in refinements that improved the study design overall. The subsequent trial will add to the limited but growing evidence on ED-based peer supports.

**Conclusions**

Capitalizing Indiana’s current efforts in order to study an already scaling and promising intervention is likely to lead to faster and more sustainable results with greater generalizability than traditional, efficacy-focused clinical research.

**Trial Registration:** ClinicalTrials.gov NCT03336268

**References**

1. Volkow ND, Collins FS. The role of science in addressing the opioid crisis. N Engl J Med. 2017;377(4):391-394.

2. Westfall JM, Mold J, Fagnan L. Practice-Based Research—“Blue Highways” on the NIH Roadmap. JAMA. 2007; 297(4):403-406.

3. Bernet AC, Willens DE, Bauer MS. Effectiveness-implementation hybrid designs: implications for quality improvement science. Implement Sci. 2013;8(Suppl 1):S2.

## A29 Evaluating associations between implementation barriers, strategies, and program performance: data from 140 substance abuse treatment programs in an integrated healthcare system

### Eric Hermes^1^, Ilse Wiechers^1,2^

#### ^1^Department of Psychiatry, Yale University, New Haven, CT, USA; ^2^Department of Veterans Affairs Office of Mental Health and Suicide Prevention, Washington, DC, USA

##### **Correspondence:** Eric Hermes (eric.hermes@yale.edu)

**Background**

Associations between contextual barriers, implementation strategies, and program performance can be evaluated using data from ongoing quality improvement programs in healthcare operations [1]. The Psychotropic Drug Safety Initiative (PDSI) is a system-wide program guiding quality improvement for psychotropic prescribing in 140 Veterans Health Administration (VHA) facilities. In 2017, PDSI began a program to increase medication assisted therapy (MAT) for Opiate Use Disorder (OUD) and Alcohol Use Disorder (AUD). This analysis characterizes perceived barriers, providing a foundation for analyzing associations between barriers, implementation strategies, and program performance.

**Materials and Methods**

Among six core policies, PDSI provides metrics of local MAT use and requires facilities to identify a champion. Facility assessments are submitted, which identify disorder focus (AUD and/or OUD) and perceived barriers (including 18 barriers previously identified by key informants as well as free text) [2]. Barriers are characterized by Consolidated Framework for Implementation Research (CFIR) construct, selection frequencies, intercorrelations, and associations with facility characteristics [3].

**Results**

All 140 VHA facilities responded: 74 (52.9%) focused on AUD, 47 (33.6%) on OUD, and 19 (13.6%) on both. Frequently selected barriers, including free text, clustered in the “individual characteristic” and “inner setting” CFIR domains: “Patients frequently refuse treatment or referral” (107 [76.4%]) and “Providers have too many competing demands” (98 [70.0%]). Neither disorder focus nor frequency of barrier identification varied significantly with level of MAT use at the facility. There was moderate intercorrelation of selected barriers (Cronbach’s alpha = 0.67). The barriers of “Not enough x-waiver providers” and “MAT required enrollment in intensive treatment programs” were selected more frequently in OUD-focused programs. Associations with specific improvement strategies used and program performance will be reported in June 2019.

**Conclusions**

The most frequently perceived barriers to increasing MAT were characteristics of patients, providers, and the local organization. Associations between barriers, disorder focus, and MAT use were weak. Data suggest a disconnect between perceived barriers and knowledge of organization performance. Linking program barriers, implementation strategies, and performance may be an implicit assumption of healthcare improvement programs. Analyzing these links demonstrates intersections between implementation science research, quality improvement practice, and health system policy.

**References**

1. Rogal SS, Yakovchenko V, Waltz TJ, Powell BJ, Kirchner JE, Proctor EK, Gonzalez R, Park A, Ross D, Morgan TR, Chartier M, Chinman MJ. The association between implementation strategy use and the uptake of hepatitis C treatment in a national sample. Implement Sci. 2017;12(1):60

2. Harris AHS, Ellerbe L, Reeder RN, Bowe T, Gordon AJ, Hagedorn H, Oliva E, Lembke A, Kivlahan D, Trafton JA. Pharmacotherapy for alcohol dependence: perceived treatment barriers and action strategies among Veterans Health Administration service providers. Psychol Serv. 2013;10(4):420–7

3. Damschroder LJ, Aron DC, Keith RE, Kirsh SR, Alexander JA, Lowery JC. Fostering implementation of health services research findings into practice: a consolidated framework for advancing implementation science. Implement Sci. 2009; 4(1):50. doi:10.1186/1748-5908-4-50

## A30 Setting the foundation for successful engagement with implementation strategies: multilevel perspectives from substance use treatment agencies

### Chariz Seijo^1^, Kendal Reeder^1^, Kristine Carandang^1^, Marisa Sklar^1^, Mark Ehrhart^2^, Cathleen Willging^3^, Gregory Aarons^1^

#### ^1^Department of Psychiatry, University of California San Diego, San Diego, CA, USA; ^2^Department of Psychology, University of Central Florida, Orlando, FL, USA; ^3^Pacific Institute for Research and Evaluation, Calverton, MD, USA

##### **Correspondence:** Chariz Seijo (ckseijo@ucsd.edu)

**Background**

A foundation of basic supports and resources is required for the successful implementation of evidence-based practices (EBPs). Inner and outer context determinants for this foundation remain unclear. As part of a cluster randomized trial testing the Leadership and Organizational Change for Implementation (LOCI) intervention for motivational interviewing (MI) implementation across substance use treatment agencies [1], we use qualitative methods to explore experiences of agency executives, supervisors, and providers regarding the multilevel determinants of strategic implementation.

**Materials and Methods**

Preliminary data from 29 individual interviews and 15 focus groups with 10 executives, 23 supervisors, and 80 providers across five agencies were examined. Notes from coaching calls conducted with supervisors randomized to the LOCI condition were explored to further contextualize engagement in the implementation strategy and MI implementation over time. The Framework Method [2] was used to synthesize data within- and between-agencies and identify emergent themes in accordance with the Exploration, Preparation, Implementation, Sustainment (EPIS) framework [3] and Edgar Schein’s organizational culture change model [4].

**Results**

Determinants of LOCI engagement and MI implementation included the structural make-up of the agency and/or clinic, timing of state-wide policy initiatives, and professional role at the agency. Agency executives, supervisors, and providers all agreed that inadequate staffing and high turnover limited the time available for LOCI engagement and MI implementation. Participants detailed how the lack of basic resources, such as not having therapy rooms, negatively impacted their ability to participate in LOCI and implement MI. Additionally, all agreed that changes in policy introduced new requirements (e.g., new EHR, billing and reporting requirements) that interfered with LOCI participation.

**Conclusions**

In order for organizations to engage effectively in implementation strategies like LOCI, a foundation of basic supports and resources is needed. The introduction of such strategic initiatives are held to be a secondary priority after basic organizational needs such as fiscal and operational viability are met. Therefore, understanding the determinants for establishing a foundation for successful EBP implementation in real-world practice is necessary to ensure that implementation strategies are successful and implementation outcomes are achieved. Implications for improving implementation strategies to target these determinants are discussed.

**Trial Registration:** ClinicalTrials.gov NCT03042832

**References**

1. Aarons GA, Ehrhart MG, Moullin JC, Torres EM, Green AE. Testing the Leadership and Organizational Change for Implementation (LOCI) Intervention in substance abuse treatment: a cluster randomized trial study protocol. Implement Sci. 2017;12(1):29.

2. Gale NK, Heath G, Cameron E, Rashid S, Redwood S. Using the framework method for the analysis of qualitative data in multi-disciplinary health research. BMC Med Res Methodol. 2013;13:117.

3. Moullin JC, Dickson KS, Stadnick NA, Rabin B, Aarons GA. Systematic review of the Exploration, Preparation, Implementation, Sustainment (EPIS) framework. Implement Sci. 2019;14(1):1.

4. Schein E. Organizational culture and leadership. 5 ed. Hoboken, NJ: John Wiley and Sons; 2017.

## A31 Involving patients, practitioners, and policy makers to develop a theory-based implementation intervention to increase the uptake of diabetic retinopathy screening

### Fiona Riordan^1^, Emmy Racine^1^, Susan Smith^2^, Aileen Murphy^1^, John Browne^1^, Patricia Kearney^1^, Sheena McHugh^1^

#### ^1^School of Public Health, University College Cork, Cork, Ireland; ^2^Department of General Practice, Royal College of Physicians in Ireland, Dublin, Ireland

##### **Correspondence:** Fiona Riordan (fiona.riordan@ucc.ie)

**Background**

Despite evidence that diabetic retinopathy screening (DRS) is effective [1], uptake remains sub-optimal in many countries, including Ireland [2-5]. Implementation strategies to enhance uptake of interventions like DRS, are not always a good fit for the context in which they are used, or do not align with stakeholder preferences [6]. We report a systematic process combining theory, stakeholder consultation and existing evidence to develop an implementation intervention to increase DRS uptake.

**Materials and Methods**

Target behaviours were identified through stakeholder interviews (n=19), and an audit of screening attendance in two primary care centres. Using patient (n=48) and health care professional (HCP) (n=30) interviews, barriers and enablers were coded using the Theoretical Domains Framework and mapped to behaviour change techniques. The APEASE (affordability, practicability, effectiveness, acceptability, side effects, equity) criteria were used to select intervention components. Effectiveness of components and delivery modes was determined through a rapid evidence review. Feasibility, local relevance and acceptability were determined through a collaborative process; consensus meetings with patients (n=15) and HCPs (n=16), and key stakeholder consultations, including the national DRS programme.

**Results**

Three target behaviours were identified; patient registration by HCP, patient consent for the programme to hold their details, and patient attendance. Patient barriers included confusion between screening and routine eye checks, forgetting, and fear of a negative result. Enablers included a recommendation from friends/family or HCPs, recognising screening importance, and ownership over their condition. HCP barriers included the time to register patients impeded or supported by practice resources, and a lack of readily available information on uptake in their local area/practice. Consensus meeting participants agreed HCP-endorsed reminders and patient information leaflets were acceptable. They felt certain delivery modes (i.e. in-person, phone and letter) were feasible and equitable, while others may exclude some practices and patients (e.g., text messages). The final intervention comprises reimbursement, training, audit/feedback and electronic prompts for HCPs, and HCP-endorsed patient reminders with an information leaflet.

**Conclusions**

A collaborative process involving multiple stakeholder consultations helped shape an intervention deemed acceptable to both patients and HCPs. The feasibility of intervention delivery in real world primary care will be evaluated through a pilot trial.

**References**

1. Cheung N, Mitchell P, Wong TY. Diabetic retinopathy. Lancet. 2010;376(9735):124-136.

2. Zwarenstein M, Shiller SK, Croxford R, Grimshaw JM, Kelsall D, Paterson JM, Laupacis A, Austin PC, Tu K, Yun L, Hux JE. Printed educational messages aimed at family practitioners fail to increase retinal screening among their patients with diabetes: a pragmatic cluster randomized controlled trial [ISRCTN72772651]. Implement Sci. 2014;9(1):87.

3. Millett C, Dodhia H. Diabetes retinopathy screening: audit of equity in participation and selected outcomes in South East London. J Med Screen. 2006;13(3):152-155.

4. Paz SH, Varma R, Klein R, Wu J, Azen SP. Noncompliance with vision care guidelines in Latinos with type 2 diabetes mellitus: the Los Angeles Latino Eye Study. Ophthalmology. 2006;113(8):1372-1377.

5. Saadine JB, Fong DS, Yao J. Factors associated with follow-up eye examinations among persons with diabetes. Retina. 2008,28(2):195-200.

6. Powell BJ, Fernandez ME, Williams NJ, Aarons GA, Beidas RS, Lewis CC, McHugh SM, Weiner BJ. Enhancing the impact of implementation strategies in healthcare: a research agenda. Front Public Health 2019,7(3).

## A32 Results from a randomized trial comparing strategies for helping CHCs implement guideline-concordant cardioprotective care

### Rachel Gold^1^, Arwen Bunce^2^, Stuart Cowburn^2^, James V. Davis^1^, Joan Nelson^2^, Deborah J. Cohen^3^, James Dearing^4^, Michael A. Horberg^5^

#### ^1^Center for Health Research, Kaiser Permanente, Portland, OR, USA; ^2^OCHIN, Portland, OR, USA; ^3^School of Medicine, Oregon Health & Science University, Portland, OR, USA; ^4^Department of Communication, Michigan State University, East Lansing, MI, USA; ^5^Kaiser Permanente - Mid-Atlantic Permanente Research Institute, Rockland, MD, USA

##### **Correspondence:** Rachel Gold (rachel.gold@kpchr.org)

**Background**

Statins can reduce cardiovascular disease (CVD) risk in patients with diabetes (DM), but prescribing often lags behind recommendations. We compared how three increasingly intensive implementation support strategies impacted community health centers’ (CHCs) adoption of electronic health record (EHR) clinical decision support tools targeting guideline-concordant statin prescribing in DM [1-3]. The tools (the ‘CVD Bundle’) were adapted from a previously successful intervention [4].

**Materials and Methods**

In this mixed methods, pragmatic trial, 29 CHCs with a shared EHR were randomized to 3 Arms that received implementation support: 1) Implementation Toolkit (CVD Bundle use instructions; Quality Improvement practice change techniques); 2) Toolkit + in-person training with follow-up webinars; or, 3) Toolkit, training, webinars, + offered practice facilitation. All study CHCs also identified a Champion to oversee related clinic activities. Statin prescription rates were compared across Arms, and with those in >300 additional CHCs which received no implementation support, a non-randomized comparison group. Prescribing (per national guidelines) was measured from 12 months pre-intervention through 36 months post-intervention. We gathered qualitative data from the randomized CHCs via on-site observations, interviews, and phone calls.

**Results**

Statin prescribing increased pre- to post-intervention for all Arms; only Arm 2 demonstrated a statistically significant change relative to comparison CHCs. Prescribing rates improved more in the study CHCs (7%, 8%, and 5% for Arms 1, 2 and 3 respectively) than the comparison CHCs (3%). These differences were not additive – CHCs that received more intensive implementation support did not have greater improvements in prescribing rates. Qualitative data suggest numerous clinic- and intervention-level factors underlying these results. Implementation strategies were not always applied as planned: the Toolkit was infrequently used, webinar attendance was poor, staff turnover was substantial, and few Arm 3 clinics were able to fully benefit from the offered practice facilitation.

**Conclusions**

This is one of the first studies to directly compare implementation strategies. The strategies employed here were associated with small improvements in the study CHCs’ guideline-concordant prescribing. Level of implementation support was less impactful than clinic ability to make changes. Guideline dissemination efforts should evaluate adopters’ needs / preferences so that subsequently deployed implementation strategies are well-received.

**Trial Registration** ClinicalTrials.gov NCT03001713

**References**

1. Powell BJ, McMillen JC, Proctor EK, Carpenter CR, Griffey RT, Bunger AC, Glass JE, York JL. A compilation of strategies for implementing clinical innovations in health and mental health. Med Care Res Rev. 2012;69(2):123-157.

2. Proctor EK, Powell BJ, McMillen JC. Implementation strategies: recommendations for specifying and reporting. Implement Sci. 2013;8:139.

3. Watkins K, Wood H, Schneider CR, Clifford R. Effectiveness of implementation strategies for clinical guidelines to community pharmacy: a systematic review. Implement Sci. 2015;10:151.

4. Gold R, Nelson C, Cowburn S, Bunce A, Hollombe C, Davis J, Muench J, Hill C, Mital M, Puro J, Perrin N, Nichols G, Turner A, Mercer M, Jaworski V, Howard C, Abiles E, Shah A, Dudl J, Chan W, DeVoe J. Feasibility and impact of implementing a private care system’s diabetes quality improvement intervention in the safety net: a cluster-randomized trial. Implement Sci. 2015;10(1):83.

## A33 Main findings from the substance abuse treatment to HIV care (SAT2HIV) project: a type 2 effectiveness-implementation hybrid trial

### Bryan Garner^1^, Stephen Tueller^1^, Steve Martino^2^, Heather Gotham^3^, Kathryn Speck^4^, Michael Chaple^5^, Denna Vandersloot^6^, Michael Bradshaw^1^, Elizabeth Ball^1^, Alyssa Toro^1^, Marianne Kluckmann^1^, Mathew Roosa^7^, James Ford^8^

#### ^1^RTI International, Research Triangle Park, NC, USA; ^2^Department of Psychiatry, Yale University, New Haven, CT, USA; ^3^Department of Psychiatry & Behavioral Sciences, Stanford University, Palo Alto, CA, USA; ^4^Public Policy Center, University of Nebraska, Lincoln, Lincoln, NB, USA; ^5^NDRI, Inc, New York, NY, USA; ^6^The Alcohol and Drug Institute, University of Washington, Seattle, WA, USA; ^7^Roosa Consulting, Syracuse, NY, USA; ^8^Center for Health Systems Research and Analysis, University of Wisconsin, Madison, Madison, WI, USA

##### **Correspondence:** Bryan Garner (bgarner@rti.org)

**Background**

Improving the integration of substance use services within HIV service settings is an important public health concern [1]. To help understand how best to improve the integration of substance use services within HIV service settings, the National Institute on Drug Abuse funded a type 2 effectiveness-implementation hybrid trial entitled the Substance Abuse Treatment to HIV Care (SAT2HIV) Project [2-3]. This presentation focuses on the SAT2HIV Project’s main findings.

**Materials and Methods**

Using a cluster-randomized design, 39 HIV service organizations and their staff were randomized to either implementation-as-usual (IAU) or IAU plus Implementation & Sustainment Facilitation (IAU+ISF). As part of the IAU condition, staff received training, feedback, and coaching in a motivational interviewing-based brief intervention (BI) for substance use. As part of the IAU+ISF, staff received the IAU strategy, as well as participated in external facilitation meetings with an ISF coach. Within each HIV service organization, eligible and consenting clients were randomized to usual care (UC) or UC plus BI (UC+BI). The analytic sample included 678 clients (82% follow-up rate), nested within 78 BI staff, nested within the 39 HIV service organizations. The preparation-phase outcome was staff time-to-proficiency (i.e., a staff-level measure of the number of days between completing the initial training and demonstrating BI proficiency). Implementation-phase outcomes were: staff implementation effectiveness (i.e., a staff-level measure of the consistency and quality of BI implementation) and client substance use at follow-up (i.e., a client-level measure of past-28 day primary substance use).

**Results**

The ISF strategy reduced time-to-proficiency (β = - .66), but this reduction was not significantly less (p < .05) than what was achieved by staff in the IAU condition. However, the ISF did significantly improved implementation effectiveness (β = .73, p < .001) beyond what was achieved in the IAU condition. Moreover, the ISF strategy did significantly improved the BI’s effectiveness for reducing client substance use (β = -2.25, p < .05).

**Conclusions**

Training, feedback, and coaching was sufficient for helping staff demonstrate proficiency in a motivational interviewing-based BI for substance use. However, the ISF strategy was found to help significantly improve implementation effectiveness and help significantly reduce client substance use.

**Trial Registration** ClinicalTrials.gov NCT03120598

**References**

1. Sweeney S. Obure CD, Maier CB, Greener R, Dehne K, Vassall A. Costs and efficiency of integrating HIV/AIDS services with other health services: a systematic review of evidence and experience. Sex Transm Infect. 2012;88(2):85-99.

2. Garner BR, Zehner M, Roosa MR., Martino S, Gotham HJ, Ball EL, Stilen P, Speck K, Vandersloot D, Rieckmann TR, Chaple M, Martin EG, Kaiser D, Ford JH. Testing the implementation and sustainment facilitation (ISF) strategy as an effective adjunct to the Addiction Technology Transfer Center (ATTC) strategy: study protocol for a cluster randomized trial. Addict Sci Clin Pract. 2017;12(1):32. doi:10.1186/s13722-017-0096-7.

3. Garner BR, Gotham HJ, Tueller SJ, Ball EL, Kaiser D, Stilen P, Speck K, Vandersloot D, Rieckmann TR, Chaple M, Martin EG, Martino, S. Testing the effectiveness of a motivational interviewing-based brief intervention for substance use as an adjunct to usual care in community-based AIDS service organizations: Study protocol for a multisite randomized controlled trial. Addict Sci Clin Pract. 2017;12(1): 31. doi:10.1186/s13722-017-0095-8.

## A34 The Integrative Systems Practice for Implementation Research (INSPIRE) model: application to context-appropriate design of a cervical cancer screening program in the Peruvian Amazon

### Valerie Paz-Soldan^1^, Magdalena Jurczuk^1^, Margaret Kosek^2^, Anne Rositch^3^, Graciela Meza^4^, Prajakta Asdul^5^, Laura Nervi^6^, J. Kathleen Tracy^7^, Javier Vasquez^4^, Renso Lopez^4^, Reyles Rios^8^, Joanna Brown^9^, Sandra Soto^9^, Patti Gravitt^7^

#### ^1^Global Community Health and Behavioral Sciences, Tulane University, New Orleans, LA, USA; ^2^Infectious Diseases and International Health, University of Virginia, Charlottesville, VA, USA; ^3^Department of Epidemiology, Johns Hopkins University, Baltimore, MD, USA; ^4^Facultad de Medicina Humana, Universidad Nacional de la Amazonia Peruana, Loreto, Peru; ^5^National Cancer Institute, Bethesda, MD, USA; ^6^College of Population Health, University of New Mexico, Albuquerque, NM, USA; ^7^University of Maryland School of Medicine, Baltimore, MD, USA; ^8^Hospital Apoyo Iquitos “Cesar Garayar García”, Coronel, Portugal; ^9^Asociación Benéfica Prisma, Buenos Aires, Argentina

##### **Correspondence:** Valerie Paz-Soldan (vpazsold@tulane.edu)

**Background**

Despite a growing body of evidence of implementation strategies for evidence-based care, a lack of sufficient detail often impedes successful reproducibility and adequate evaluation of their efficiency [1]. Development and specific description of operational and reproducible strategies are crucial to efficiently and sustainably promoting uptake of evidence-based practice.

**Materials and Methods**

In our effort to design a sustainable cervical cancer screening program in Iquitos, Peru, we developed the Integrative Systems Practice for Implementation Research (INSPIRE) Model, a multifaceted strategy that blends together existing theoretical frameworks and defines specific tools for use at each phase. INSPIRE is a participatory, iterative process involving four phases: system understanding, finding leverage, acting, and learning/adapting. Mixed methods are used to create the shared understanding of the screening system and to facilitate identification of leverage points for change. A systems modeling tool was designed to compare alternative screening systems to facilitate the decision-making process in a design workshop setting and working groups were formed to design new system processes.

**Results**

Through phases 1-3 of the INSPIRE model, we engaged more than 90 multi-level stakeholders in the design of a new and improved screen and treat system. Elaboration of system process maps through triangulation of the mixed-methods data served to create a shared reference of the current system in participatory discussions. Significant leverage opportunities were identified, including reducing fragmentation, inefficiency, and a lack of standardization to increase women’s acceptability of screening and adherence to continuum of care. A variety of interventions were evaluated and ultimately, stakeholders recommended adoption of HPV testing/self-sampling to increase coverage and ablative treatment of all HPV-positive women to reduce loss to follow up.

**Conclusions**

Continued success in engagement of stakeholders in shared decision making, including current development of a detailed implementation plan using similar user-centered design, suggests that using a SPF in designing implementation strategies increases a sense of ownership in the process, which may lead to more sustainable screening programs in LMIC compared with ‘top-down’ approaches.

**Reference**

1. Proctor EK, Powell BJ, McMillen JC. Implementation strategies: recommendations for specifying and reporting. Implement Sci. 2013;8:139. doi:10.1186/1748-5908-8-139

## A35 A secondary analysis of longitudinal state-level support for school-based health centers mental health services

### Tatiana Bustos, Amy Drahota, Kaston Anderson-Carpenter

#### College of Social Science, Michigan State University, East Lansing, MI, USA

##### **Correspondence:** Tatiana Bustos (bustosta@msu.edu)

**Background**

More than 20% of children in the U.S. experience mental health difficulties, with only about 30% receiving adequate mental health (MH) treatment. Moreover, MH service disparities are disproportionate among children who live in low-income areas [1-2]. School-based health centers (SBHCs), a comprehensive service delivery model integrating physical and MH services within school settings, reduce healthcare access barriers by functioning as medical centers for children in low-income areas. While many SBHCs in the U.S. offer some type of MH service, not all centers are equipped to provide needed MH services. MH service variations may be attributed to state-based policies, including funding, oversight support and standards. These outer contextual factors are thought to influence MH service provision by contributing to expansion and sustainment of services over time [3-7]. This study aimed to examine how state-based outer contextual variables influence the number of SBHC-reported MH services over time.

**Materials and Methods**

The external policy and incentive domains of the Consolidated Framework for Implementation Research (CFIR) were used to organize the secondary data analysis of the State Policy Survey, a survey administered to policymakers knowledgeable of SBHC criteria within each state. Specifically, state-based policymakers reported state support for SBHC funding, oversight and policies over four time-points: 2005, 2008, 2010, and 2014, while SBHC personnel reported the number of MH services at these time-points. A total of 4,232 SBHCs within 41 states were included in the study. To account for inter-dependent groups of SBHCs within states, a linear mixed model analysis (LMM) was conducted to identify key variables within the domain of external policy and incentives that were significantly related to the number of SBHC-reported MH services from 2005-2014.

**Results**

Results indicated significant variations in the number of SBHC-reported MH services, accounted by state and time. Notably, most outer contextual variables, with the exception of state general funds on substance use treatment and referral services, were significantly associated with more MH services over time.

**Conclusions**

Findings suggest that there are significant relationships between external policies and the number of MH services being delivered by SBHCs. However, these outer contextual variables had differential impacts depending on MH service type.

**References**

1. Bains RM, Diallo AF. Mental health services in school-based health centers: systematic review. J Sch Nurs. 2016; 32(1):8-19. doi: 10.1177/1059840515590607

2. Brown MB, Bolen LM. School-based health centers: strategies for meeting the physical and mental health needs of children and families. Psychol Sch. 2003;40(3):279-287.

3. Doll B, Nastasi BK, Cornell L, Song SY. School-based mental health services: Definitions and models of effective practice. J Appl Sch Psychol. 2017;33(3):179-194. doi: 10.1080/15377903.2017.1317143

4. Sprigg SM, Wolgin F, Chubinski J, Keller K. School-based health centers: a funder’s view of effective grant making. Health Aff. 2017;36(4):768. doi: 10.1377/hlthaff.2016.1234

5. Anyon Y, Moore M, Horevitz E, Whitaker K, Stone S, Shields JP. Health risks, race, and adolescents’ use of school-based health centers: policy and service recommendations. J Behav Health Serv Res. 2013;40(4):457–468. doi: 10.1007/s11414-013-9356-9.

6. Price OA. School-centered approaches to improve community health: Lessons from school-based health centers. Economic Studies at Brookings. 2016:5:1-17.

7. Damschroder LJ, Aron DC, Keith RE, Kirsh SR, Alexander JA, Lowery JC. Fostering implementation of health services research findings into practice: a consolidated framework for advancing implementation science. Implement Sci. 2009; 4:50. doi:10.1186/1748-5908-4-50.

## A36 Teacher perspectives on the development of the Beliefs and Attitudes for Successful Implementation in Schools for Teachers (BASIS-T)

### Andrew Thayer^1^, James Merle^1^, Madeline Larson^1^, Jenna McGinnis^1^, Clayton Cook^1^, Aaron Lyon^2^

#### ^1^Department of Educational Psychology, University of Minnesota, Twin Cities, Minneapolis, MN, USA; ^2^Department of Psychiatry and Behavioral Sciences, University of Washington, Seattle, WA, USA

##### **Correspondence:** Andrew Thayer (thaye053@umn.edu)

**Background**

Implementation barriers exist at all levels of service provision within an organization, yet implementation is ultimately dependent on individuals [1]. Converging lines of research have demonstrated that individual beliefs and attitudes are associated with implementation outcomes [2-3]. Active-implementation strategies targeting individuals can be effective for preventing implementer drift, yet they occur after implementation is underway. Pre-implementation strategies occurring prior to uptake may be effective in aligning beliefs and attitudes resulting in positive shifts in individuals’ contemplation and intention for implementation [4]. Historically, pre-implementation strategies have produced low implementation outcomes, especially when lacking a strong theoretical foundation [5]. The purpose of this study was to collect stakeholder feedback on the development of a blended pre-implementation strategy, Beliefs and Attitudes for Successful Implementation in Schools for Teacher (BASIS-T), to precede an evidence-based training in classroom management.

**Materials and Methods**

Twenty-two teachers and support staff from three Midwest school districts of diverse urbanicity engaged in a 3.5 hour demonstration of BASIS-T, which is grounded in the Theory of Planned Behavior [4], thus targeting teachers’ attitudes, social norm perceptions, and self-efficacy beliefs. Throughout the demonstration, teachers rated each segment for its acceptability and impact on shifting beliefs and attitudes. Participants completed pre- and post-ratings of their own beliefs, attitudes, and intentions, engaged in a nominal group process to elicit feedback for revising the strategies, and answered open-ended questions.

**Results**

Participant feedback from nominal group processes highlighted modifications to the pre-implementation strategy to improve its impact on beliefs, attitudes and implementation. Participants identified a need for more evocative and engaging activities to better encode the importance of EBP implementation, bolster their self-efficacy, and address social norm perceptions. Average ratings indicated BASIS-T content was highly impactful and acceptable. Relatively lower-rated segments were considered for revision.

**Conclusions**

Pre-implementation strategies represent potentially useful techniques for aligning providers’ beliefs and attitudes prior to implementation to facilitate better adoption. Stakeholder feedback is an effective method for informing the development of these strategies. The results of this demonstration study provided several recommendations and guidelines for how to build effective, school-based pre-implementation strategies to boost EBP adoption.

**References**

1. Aarons GA, Hurlburt M, Horwitz SM. Advancing a conceptual model of evidence-based practice implementation in public service sectors. Adm Policy Ment Health. 2011;38(1):4-23.

2. Cook CR, Lyon AR, Kubergovic D, Wright DB, Zhang Y. A supportive beliefs intervention to facilitate the implementation of evidence-based practices within a multi-tiered system of supports. Sch Ment Health. 2015;7(1):49-60.

3. Stokke K, Olsen NR, Espehaug B, Nortvedt MW. Evidence based practice beliefs and implementation among nurses: a cross-sectional study. BMC Nurs. 2014;13(1):8.

4. Ajzen I. The theory of planned behavior. Organ Behav Hum Decis Process. 1991;50(2):179-211.

5. Yoon KS, Duncan T, Lee SW, Scarloss B, Shapley KL. Reviewing the evidence on how teacher professional development affects student achievement. Issues & Answers. REL 2007-No. 033. Regional Educational Laboratory Southwest (NJ1). 2007.

## A37 Randomized trial to optimize a brief online training and consultation strategy for measurement-based care in school mental health

### Aaron Lyon, Freda F. Liu, Jessica I. Coifman, Heather Cook, Kevin King, Kristy Ludwig, Amy Law, Shannon Dorsey, Elizabeth McCauley

#### University of Washington, Seattle, WA, USA

##### **Correspondence:** Aaron Lyon (lyona@uw.edu)

**Background**

Pragmatic and streamlined implementation strategies are necessary for efficient quality improvement [1]. Training and post-training consultation are cornerstone implementation strategies [2], but are often lengthy and resource-intensive [3]. Further, few studies have evaluated their mechanisms of action [4]. Development and optimization of these strategies requires attention to (a) user experience, to ensure that the strategy is compelling and easy to use; and (b) strategy effectiveness, to ensure that the strategy influences its targeted mechanisms of action. This presentation will present findings from a project that designed and tested a brief, multifaceted online training and post-training consultation strategy to target each strategy’s putative mechanisms of action and support measurement-based care (MBC) practices among school-based mental health clinicians.

**Materials and Methods**

Iterative development of the online training and post-training consultation strategies involved gathering stakeholder input via four rounds of usability testing and two group cognitive walkthrough sessions with representative clinician users. This culminated in randomized trial in which 77 geographically diverse school-based mental health clinicians were randomized to (1) implementation as usual (IAU; no training or consultation, n = 40), or to online training plus one of three consultation dosages: (2) 2 weeks, (3) 4 weeks, or (4) 8 weeks. Consultation included live consultation video calls (once every two weeks) and asynchronous message board discussion. Following training, training mechanisms (knowledge, attitudes, skill), consultation mechanisms (collaboration, responsiveness, accountability), and self-reported clinician MBC practices were tracked for 16 weeks.

**Results**

Preliminary analysis (multilevel modeling) showed that online training led to an immediate increase in MBC knowledge relative to controls (β= .06, p<.05). Following consultation, participants demonstrated greater growth in self-reported MBC skills (β=.028, p<0.01) and attitudes (e.g., perceived benefit of MBC, β=.028, p<0.05) and superior MBC practices (e.g., use of standardized and individualized assessments, β=.013, .032, respectively, p<0.01). Clear consultation dosage effects have yet to emerge from preliminary analyses with 16 (of 32) weeks of follow-up data. It is possible that the consultation groups may become more disparate with longer follow-up.

**Conclusions**

Few studies have examined implementation strategy mechanisms of action. The current findings suggest that the online training and consultation package influenced many of its target mechanisms and also lead to higher MBC practices among school mental health clinicians than IAU. Thus far, preliminary results suggest that shorter durations of consultation may be comparable to longer durations.

**References**

1. Lewis CC, Klasnja P, Powell BJ, Tuzzio L, Jones S, Walsh-Bailey C, Weiner B. From classification to causality: advancing understanding of mechanisms of change in implementation science. Front Public Health. 2018;6:136.

2. Lyon AR, Pullmann MD, Walker SC, D’Angelo G. Community-sourced intervention programs: Review of submissions in response to a statewide call for “promising practices.” Adm Policy Ment Health. 2017;44(1):16-28.

3. Olmstead T, Carroll KM, Canning-Ball M, Martino S. Cost and cost-effectiveness of three strategies for training clinicians in motivational interviewing. Drug Alcohol Depend. 2011;16(1-3):195-202.

4. Williams NJ. Multilevel mechanisms of implementation strategies in mental health: Integrating theory, research, and practice. Adm Policy Ment Health. 2016;43(5):783-798.

## A38 Implementing service cascade models with fidelity: a case study of cross-system collaboration strengths and challenges

### Alicia Bunger, Christy Kranich, Susan Yoon, Lisa Juckett

#### Ohio State University, Columbus, OH, USA

##### **Correspondence:** Alicia Bunger (bunger.5@osu.edu)

**Background**

Service cascade models move individuals across systems through a sequence of screening, assessment, referral, treatment, and monitoring activities. Successful implementation depends on strong collaboration and change across systems, but these models have received limited empirical attention.

**Materials and Methods**

This case study examines fidelity of a cascade model implemented across child welfare and mental health systems (intended to improve children’s access to specialty mental health care), and identifies collaboration strategies and challenges at each stage.

**Results**

Fidelity to initial mental health screening and assessment was high and attributed to service co-location, collaborative workflow planning, and linked data systems. However, fidelity to referral/treatment components was low and associated with case planning challenges, contract disruptions, and workforce shortages.

**Conclusion**

Our findings suggest that fidelity breakdowns at any point in the service cascade can negatively affect clients’ access to services, especially during key service transitions. Implementing these models likely depends on cross-system collaboration approaches that align front-line practice and agency operations at each stage of the model.

## A39 A pragmatic method for costing implementation strategies using the time-driven activity-based costing

### Zuleyha Cidav^1^, Jeffrey Pyne^2^, Geoffrey Curran^2^, David Mandell^1^, Rinad S. Beidas^1^, Jennifer Mautone^1^, Ricardo Eiraldi^1^, Steven Marcus^1^

#### ^1^University of Pennsylvania, Philadelphia, PA, USA; ^2^University of Arkansas for Medical Sciences, Little Rock, AR, USA

##### **Correspondence:** Zuleyha Cidav (zcidav@upenn.edu)

**Background**

Strategies to implement evidence-based practices consume scarce resources and incur costs. Although critical for decision makers with constrained budgets and limited resources, such resource use and cost information are not typically reported. This is at least partly due to a lack of clearly defined and standardized costing methods for use in implementation science. This study presents a pragmatic approach to systemically estimating resource use and costs of implementation strategies using a well-established business accounting system. The method is demonstrated by estimating the first-year implementation costs of a group-based cognitive behavioral therapy program for students with externalizing disorders in six Philadelphia schools.

**Materials and Methods**

Time-driven activity-based costing (TDABC) is combined with the existing guidelines for implementation strategy specification and reporting. Implementation protocol, measures, project notes and key personnel interviews were used to map the implementation process by specifying the strategies with their actors, specific action steps, temporality, and dose. The dose is defined for each action step as the person-hours invested in its completion, and accounts both for frequency and intensity of the action step. Implementation strategy dose is the sum of person-hours on each action step that constitute the strategy. Project resources are identified, and the price per unit person-hour is calculated as per the TDABC. Costs of action steps, strategies and implementation project is reported from a payer perspective.

**Results**

Estimated total cost was $63,842; $10,640 per school. The largest cost incurred was for the communication efforts ($30,691), which involved in-person meetings, phone calls, and email exchanges. Next largest costs were for the stakeholder engagement, consultation/coaching, and supervision, which comprised 19%, 15%, 12% of total costs, respectively. Assessment/evaluation and training constituted the smallest costs, at 4% and 3% respectively.

**Conclusions**

This method allows for inclusion of implementation costs in the efforts of strategy specification, tracking and reporting. It serves as a pragmatic tool to operationalize the conduct of the implementation activities, track the resources consumed and estimate associated costs. It could facilitate the routine incorporation of cost analysis and economic evaluations into implementation research. It provides granular cost information which could be used to identify and address the inefficiencies in the implementation process.

## A40 Shared goal, different languages: communication between implementation researchers and social entrepreneurs

### Enola Proctor^1^, Rachel Tabak^1^, Cole Hooley^1^, Virginia McKay^1^, Emre Toker^2^

#### ^1^Brown School, Washington University in St. Louis, St. Louis, MO, USA; ^2^Arizona State University, Tempe, AZ, USA

##### **Correspondence:** Enola Proctor (ekp@wulst.edu)

**Background**

Implementation science and social entrepreneurship share a common objective of maximizing the uptake of new practices and innovations. Both disciplines offer complementary tools which could be leveraged to improve the ultimate impact of innovations, their scale-up, and sustainment. For example, entrepreneurship’s focus on market assessment, financial outlook, etc. can be coupled with implementation science use of data, models, and methods. However, communication between these fields has been limited. To explore how these complementary groups might learn from each other, this paper presents data about how implementation researchers understand and respond to the kinds of questions entrepreneurs ask about innovation roll-out.

**Materials and Methods**

We conducted one-on-one cognitive interviews [1] with 15 dissemination and implementation researchers recruited from training programs in implementation science to capture their ability to understand and answer key questions from entrepreneurs and refine a tool to support dialogue. Participants were guided through the tool item-by-item. Prompts from the interviewer helped identify problems with question clarity and comprehension. A summary of the responses was developed to identify problematic elements and identify revisions necessary to improve clarity. The discussions tapped the researchers’ perception of demand for the innovation, estimates of benefit, knowledge of who would pay, sustainment challenges, and comfort working with business and entrepreneurial partners.

**Results**

Implementation researchers understood and were able to answer questions about the problem their innovation seeks to solve, their roll-out plans, and stakeholders and team members involved. They reported the following types of questions as easy to understand but hard to answer: who would pay for the innovation, numbers of people who would benefit, and how to sustain the innovation once adopted. Researchers varied in their comfort with technology supports and business/entrepreneurial partnerships.

**Conclusions**

Entrepreneurial partnerships can provide important supports to successful implementation and sustained delivery of interventions, including technology supports, market analysis, and financial investment. Implementation researchers understand the types of questions entrepreneurs pose about their projects, but find those questions difficult to answer, suggesting the importance of establishing interface between these fields. Tools to prepare researchers to interact with entrepreneurs could take these factors into account to facilitate communication between audiences.

**Reference**

1. Willis GB. Cognitive interviewing: A tool for improving questionnaire design. Thousand Oaks, CA: Sage Publications, Inc; 2005.

## A41 Making it happen: implementation efforts for systems level change in child welfare

### Melissa Bernstein^1^, Brent Crandal^1^, Gregory Aarons^2^, Kimberly Giardina^3^

#### ^1^Rady Children’s Hospital, San Diego, CA, USA; ^2^University of California San Diego, San Diego, CA, USA; ^3^County of San Diego Health and Human Services, San Diego, CA, USA

##### **Correspondence:** Melissa Bernstein (mbernstein1@rchsd.org)

**Background**

If diffusion is “letting it happen,” and dissemination is “helping it happen,” implementation is the business of “making it happen” [1]. To make change happen within large systems, relationships between implementation scientists, intermediaries, and system leaders are created. These relationships, which serve as the vehicle for translating science into practice, are often understated and under examined. This panel presents two initiatives designed to implement best practices within a large child welfare service system focused on all three perspectives: system leadership, presented by Kimberly Giardina, MSW; implementation science, presented by Greg Aarons, PhD., and intermediary implementation, presented by Brent Crandal, PhD., and Melissa Bernstein, PhD.

**Materials and Methods**

The two system changes that will provide context for this panel include, first, the Advancing California’s Trauma Informed Systems (ACTS) Initiative. ACTS was developed to create collaborative partnerships with child-welfare county leaders across California to advance trauma- and evidence-informed system change through implementation planning, followed by technical assistance, training, outcome monitoring, and sustainment planning. Second, the Community-Academic Partnerships for the Translational Use of Research Evidence (CAPTURE) project explores how research can be used to improve child welfare policy, programs and practices through a partnership between the University of California at San Diego (UCSD) and the County of San Diego, Health and Human Services Agency, Child Welfare Services.

**Results**

Using a mixed-methods research design, CAPTURE examines the processes that shape instrumental and conceptual use of research evidence (URE) and investigates how change mechanisms influence URE.

**Conclusions**

Guided by the Exploration, Preparation, Implementation, Sustainment (EPIS) implementation framework, we will highlight the translation of implementation science into concrete strategies used to create change within a child welfare system, including leadership engagement, team development, data utilization, stakeholder involvement, quality improvement methods, and the use of consultation.

**Reference**

1. National Implementation Research Network Active Implementation Practice and Science (Issue Brief No. 1). 2016. https://nirn.fpg.unc.edu/sites/nirn.fpg.unc.edu/files/resources/NIRN-Briefs-1-ActiveImplementationPracticeAndScience-10-05-2016.pdf

## A42 A multiple case study of a tailored approach to implementing measurement-based care for depression

### Byron J. Powell^1^, Meredith Boyd^2^, Hannah Kassab^3^, Cara C. Lewis^4^

#### ^1^Brown School, Washington University in St. Louis, St. Louis, MO, USA; ^2^University of California, Los Angeles, Los Angeles, CA, USA; ^3^Ohio University, Athens, OH, USA; ^4^Kaiser Permanente Washington Health Research Institute, Seattle, WA, USA

##### **Correspondence:** Byron J. Powell (bjpowell@wustl.edu)

**Background**

Tailoring implementation strategies to site-specific determinants (barriers and facilitators) is a promising way of improving implementation and clinical outcomes [1]. However, more empirical work is needed to determine whether tailored approaches to implementation are more effective than standard multifaceted strategies, and to develop optimal methods for linking implementation strategies to identified determinants [2]. The NIMH-funded “Implementing Measurement-Based Care (iMBC) for Depression in Community Mental Health” study addressed these gaps by comparing a standard multifaceted strategy to a tailored approach to implementation in a dynamic cluster randomized trial [3]. This study draws upon data from the intervention group (i.e., tailored condition) of the iMBC study to 1) describe how clinics tailored implementation strategies to site-specific barriers, and 2) evaluate the extent to which the implementation strategies they used could plausibly address identified determinants.

**Materials and Methods**

The six clinics in the tailored condition were compared to each other using a multiple case study design. Descriptions of each clinic’s approach to tailoring, the determinants they attempted to address, and the implementation strategies they used were derived from recordings and notes from implementation team meetings across five months. Determinants were deductively coded using the Consolidated Framework for Implementation Research (CFIR) [4] and implementation strategies were deductively coded using an established taxonomy [5]. Plausibility of linkages between barriers and strategies was determined in two ways. First, two authors independently rated plausibility using a 5-point Likert scale. Second, each strategy-determinant linkage was compared to the results of a previous study that established preliminary linkages between CFIR determinants and implementation strategies [6].

**Results**

Four of the six clinics prioritized barriers identified quantitatively during the needs assessment phase of the study, and explicitly selected implementation strategies to address them. Clinics reported using an average of 39 implementation strategies, which were categorized into 26 of the 68 discrete implementation strategies identified by Powell et al. [7]. Plausibility of the linkages between barriers identified and strategies selected will also be presented.

**Conclusions**

This study contributes to implementation science and practice by highlighting strengths and weaknesses of community mental health clinics’ approaches to tailoring implementation strategies, and by suggesting ways in which methods for tailoring could be improved.

**Trial registration:** ClinicalTrials.gov NCT02266134

**References**

1. Baker R, Comosso-Stefinovic J, Gillies C, Shaw EJ, Cheater F, Flottorp S, Robertson N, Wensing M, Fiander M, Eccles MP, Godycki-Cwirko M, van Lieshout J, Jäger C. Tailored interventions to address determinants of practice. Cochrane Database Syst Rev. 2015;4(CD005470):1-118. doi:10.1002/14651858.CD005470.pub3

2. Powell BJ, Beidas RS, Lewis CC, Aarons GA, McMillen JC, Proctor EK, Mandell DS. Methods to improve the selection and tailoring of implementation strategies. J Behav Health Serv Res. 2017;44(2):177-194. doi: 10.1007/s11414-015-9475-6.

3. Lewis CC, Scott K, Marti CN, Marriott BR, Kroenke K, Putz JW, Mendel P, Rutkowski S. Implementing measurement-based care (iMBC) for depression in community mental health: a dynamic cluster randomized trial study protocol. Implement Sci. 2015;10:127. doi:10.1186/s13012-015-0313-2.

4. Damschroder LJ, Aron DC, Keith RE, Kirsh SR, Alexander JA, Lowery JC. Fostering implementation of health services research findings into practice: a consolidated framework for advancing implementation science. Implement Sci. 2009;4:50.

5. Boyd MR, Powell BJ, Endicott D, Lewis CC. A method for tracking implementation strategies: An exemplar implementing measurement-based care in community behavioral health clinics. Behav Ther. 2018;49:525-537. doi:10.1016/j.beth.2017.11.012.

6. Damschroder LJ, Waltz TJ, Abadie B, Powell BJ. Choosing implementation strategies to address local contextual barriers. Implement Sci. 2018;13(Suppl3: A76):37-38.

7. Powell BJ, McMillen JC, Proctor EK, Carpenter CR, Griffey RT, Bunger AC, Glass JE, York JL. A compilation of strategies for implementing clinical innovations in health and mental health. Med Care Res Rev. 2012;69(2):123-157. doi:10.1177/1077558711430690.

## A43 Systematic adaptation of evidence-based interventions: an intervention mapping approach

### Maria E. Fernández^1^, Cam Escoffery^2^, Maya Foster^1^, Patricia Mullen^1^

#### ^1^University of Texas School of Public Health, Houston, TX, USA; ^2^Rollins School of Public Health, Emory University, Atlanta, GA, USA

##### **Correspondence:** Maria E. Fernández (maria.e.fernandez@uth.tmc.edu)

**Background**

Evidence-based public health translation of research to practice is essential to improving the public’s health. Adaptation of evidence-based interventions (EBIs) to promote health and prevent disease is an essential process for implementation and dissemination research and practice. Challenges faced in practice include identifying EBIs that are suitable for new populations and settings and adapting them to fit needs.

**Materials and Methods**

A recently published scoping review by our team summarized 13 adaptation frameworks and identified common steps to guide the adaptation process [1]. We also conducted a systematic review of adapted interventions and described reasons for adaptation according to previously identified categories [2-3]. These studies have shown that while many examples and adaptation models exist, they provide only limited guidance on how to make decisions about what should change and what should remain the same.

**Results**

We present a framework based on the Intervention Mapping protocol that provides step-by-step guidance on selection and adaptation of EBIs [4-5]. The process includes the development of a logic model of change (LMC) based on the community assessment. An LMC is a diagram of what that describes the relationship between changes needed in determinants, behavior and environment to bring about improvements in health and quality of life. The LMC is then compared with the basic features of available EBIs (determinants addressed, resources needed, etc.) to assess potential fit with the new population or setting. Following selection, planners further examine the internal logic of the EBI including the behaviors and environmental conditions that were the targets of the original EBI, the determinants addressed, and the change methods and/ or strategies used. Planners then compare the EBI features to the LMC to determine what needs to be adapted while maintaining change methods used in the original intervention since these often represent the intervention’s core elements.

**Conclusions**

We describe the development and testing of an online tool (I M ADAPT) for finding and adapting evidence based interventions for cancer control. We also describe how we are applying Intervention Mapping and the online tool in a project to improve the use of interventions from the National Cancer Institute’s Research Tested Intervention Programs (RTIPs) resource.

**References**

1. Escoffery C, Lebow-Skelly E, Udelson H, Boing E, Fernandez M, Mullen PD. A scoping study of program adaptation frameworks for evidence-based interventions. Transl Behav Med. 2019;9(1):1-10. doi: 10.1093/tbm/ibx067.

2. Escoffery C, Lebow-Skelley E, Haardoerfer R, Boing E, Udelson H, Wood R., Hartman M, Fernandez ME, Mulled PD. A systematic review of adapted evidence-based interventions globally. Implement Sci. 2018;13:125.

3. Stirman SW, Miller, CJ, Toder K, Calloway A. Development of a framework and coding system for modifications and adaptions of evidence-based interventions. Implement Sci. 2013;8:65.

4. Bartholomew Eldredge LK, Markham CM, Ruiter RAC, Fernández M.E, Kok G, Parcel GS. Planning health promotion programs: an Intervention Mapping approach, 4th ed. San Francisco, CA: Jossey-Bass; 2016.

5. Highfield L, Hartman MA, Mullen PD, Rodriguez SA, Fernandez ME, Bartholomew LK. Intervention Mapping to adapt evidence-based interventions for use in practice: increasing mammography among African American women. Biomed Res Int. 2015;160103. doi:10.1155/2015/160103.

## A44 Co-creation of change in policy and practice: the Community Academic Partnership for Translational Use of Research Evidence (CAPTURE)

### Gregory Aarons^1^, Kimberly Giardina^2^, Danielle Fettes^3^, Margo Fudge^2^, the CAPTURE Steering Committee

#### ^1^University of California, San Diego, San Diego, CA, USA; ^2^Child Welfare Services - County of San Diego Health & Human Services Agency, San Diego, CA, USA; ^3^Child and Adolescent Services Research Center, San Diego, CA, USA

##### **Correspondence:** Gregory Aarons (gaarons@ucsd.edu)

**Background**

Youths in the child welfare (CW) system face a myriad of poor outcomes with regard to social/emotional development and behavioral health. While there is research evidence relevant for CW services, integration and use of such evidence in policy, planning, and service delivery is limited. The Community Academic Partnership for Translational Use of Research Evidence (CAPTURE), is a community-academic partnership to increase use of research evidence in policy, programs, and practice. Guided by the Exploration, Preparation, Implementation, Sustainment (EPIS) framework, CAPTURE engages outer context system level stakeholders, inner context organizational stakeholders and academic partners in a bidirectional partnership. The aims of CAPTURE are: 1) To establish and test the use of a partnership model to increase use of research evidence (URE) in policy, program, and practice, and 2) To identify key mechanisms by which CAPTURE operates including - but not limited to - cultural exchange between researchers and community partners, leadership and organizational change, and use of quality improvement methods to test and put goals into practice.

**Materials and Methods**

Mixed-methods will provide a detailed and nuanced understanding of the process of collaboration and URE in a large public sector service system, and describe the complexity of instantiating URE across system and organization levels. Quantitative data assessing cultural exchange, leadership and climate for URE are being collected by online surveys of providers and stakeholders. Qualitative methods include interviews, focus groups, observation of CAPTURE meetings, and document review.

**Results**

Community-academic partnerships are a promising approach to improving collaboration and integrating research evidence in decision making for policy and practice.

**Conclusions**

A better understanding of ways to develop and establish community-academic partnerships can help to promote their use in other settings.

## A45 Developing a strategic implementation research plan within an integrated healthcare system

### Sara J. Landes^1,2^, JoAnn E. Kirchner^1,2^, Mark Bauer^1,3^, Christopher Miller^1,3^, Mona Ritchie^1,2^, Jeffrey L. Smith^1,2^

#### ^1^Behavioral Health QUERI, Little Rock, AR, USA; ^2^Central Arkansas Veterans Healthcare System, Little Rock, AR, USA; ^3^VA Boston Healthcare System, Boston, MA, USA

##### **Correspondence:** Sara J. Landes (sara.landes@va.gov)

**Background**

The US Department of Veterans Affairs (VA) Quality Enhancement Research Initiative (QUERI) funds quality improvement and program evaluation studies to support implementation and evaluation efforts needed to improve healthcare for our nation’s veterans [1]. QUERI funds programs that focus on areas of care and/or implementation strategies. The Behavioral Health QUERI program, one of 15 currently funded programs, has developed a structured method for strategic planning to match program priorities and implementation research projects with priorities of stakeholders and the healthcare system. Embedded with the organizational structure of the Behavioral Health QUERI are a Stakeholder Council (SC) comprised of veterans of all eras, family members, providers, and local and regional leadership; and a Strategic Advisory Group (SAG) comprised of national healthcare leaders inside and outside of VA and veteran representatives.

**Materials and Methods**

The strategic planning methods are iterative and include stakeholders from multiple levels of the national health care system (e.g., providers, leadership at various levels, veterans, and subject matter experts). The strategic planning methods include identification of priorities of the healthcare system, creation of a planning committee, development of a key stakeholder interview, and identifying key informants. Stakeholder interviews are conducted across multiple layers of the organization (e.g., veterans, local medical center, network level, and national leadership). Qualitative data are synthesized across key question domains and findings are matched to existing initiatives and/or research opportunities by the planning committee. Priorities and potential projects are identified and prioritized with the SC and SAG. These are vetted with a sample of the initial key informants.

**Results**

Behavioral Health QUERI is currently conducting strategic planning. VA priorities have been identified by the SC and SAG. Stakeholder interviews have been completed with three network leaders and three national leaders (mental health operations, suicide prevention, and technology). Results of themes will be presented, with a focus on how to conduct strategic implementation research planning in collaboration with a variety of stakeholders.

**Conclusions**

Strategic implementation research planning is critical to developing a research plan that both helps the healthcare system move forward in its goals and advances implementation science.

**Reference**

1. Stetler CB, Mittman BS, Francis J. Overview of the VA Quality Enhancement Research Initiative (QUERI) and QUERI theme articles: QUERI Series. Implement Sci. 2008;3:8.

## A46 Bridging the implementation research to practice gap: exploring collaboration and solutions between researchers, policy-makers and funders, implementation supports and implementing organisations

### Chair: Jacquie Brown^1^, Discussant: Aaron Lyon^2^, Panelists: Byron Powell^3^, Jenna McWilliam^4^, Arthur Evans^5^

#### ^1^Families Foundation, Hilversum, The Netherlands; ^2^University of Washington, Seattle, WA, USA; ^3^Brown School, Washington University in St. Louis, St. Louis, MO, USA; ^4^Triple P International, Brisbane, Queensland, Australia; ^5^American Psychological Association, Washington, DC, USA

##### **Correspondence:** Jacquie Brown (jacquie.brown@familiesfoundation.net)

**Background**

The intersection between the various perspectives is critical in influencing the extent to which implementation science can be translated into implementation practice. At this juncture of the development of implementation science it is critical that we develop the capacity to integrate the knowledge from each perspective to ensure that implementation science achieves its ultimate goal of “bridging the gap”. Collaboration will promote the awareness, understanding and ability to consider all perspectives whilst further developing the science and supports for application of implementation science.

Having explored the “constructive tension” between implementation researchers/intermediaries/purveyors/implementing organisations at GIC 2017, at which themes were identified that reflected the challenges between implementation science and implementation practice, the themes were furthered discussed at Global Evidence and Implementation Symposium, 2018. Greater insight was generated into the challenges for each perspective and how they might be addressed.

**Materials and Methods**

The panel proposed for SIRC 2019, with active participation from the attendees, will build on the previous discussions. This participatory discussion will focus on solutions, exploring and developing potential next steps to address identified barriers, and build on existing partnerships across perspectives.

The session will open with a brief reference to information gathered at the previous two sessions. Each panelist will then offer thoughts from their perspectives on what might promote greater integration between each perspective.

**Results**

Following the brief presentations, the floor will be opened for a facilitated discussion framed around three questions:

1) What are the actions we can take to increase the collaboration between the various contributors to implementation science and practice?

2) What are the barriers you experience that might interfere with these actions?

3) What are the opportunities/enablers you could use to promote collaborative development of implementation science and practice? The session will close with a summary from the discussant.

**Conclusions**

The objectives for the panel discussion include:

1) Increasing awareness of the needs, challenges and opportunities for collaboration across perspectives

2) Identifying priorities for research and partnerships for action

3) Confirm 3 areas for action for 2019 – 2021

This is a single theme symposium with brief presentations from the panelists followed by open-floor discussion.

## A47 HealthLinks: evaluation challenges and learnings from three organisational perspectives

### Norm Good, Philippa Niven

#### CSIRO, Canberra, Australia

##### **Correspondence:** Norm Good (norm.good@csiro.au)

**Background**

Health care systems across the developed world are currently facing a similar set of challenges. Populations are ageing and chronic illnesses are becoming more prevalent. A relatively small subset of complex patients with chronic medical conditions account for a large proportion of hospital re-admissions and consume a significant number of hospital resources [1]. HealthLinks Chronic Care (HLCC) is an initiative undertaken by the Victorian Department of Health and Human Services (DHHS) to provide a flexible funding model for participating hospitals to convert projected inpatient costs towards new or improved patient centred models of care with the aim of reducing unplanned hospital admissions.

**Materials and Methods**

Six health services within Victoria, Australia have implemented a new or improved model of care unique to their health service needs and the demographic profile of their patient population with an extra three acting as controls. DHHS contracted the Commonwealth Scientific and Industrial Research Organisation (CSIRO) to work on a cosponsored system level evaluation of HLCC. The evaluation is based on the RE-AIM framework [2] and uses a comprehensive mixed methods approach including analysis of routinely collected hospital data using a BACI design [3], a quality of life patient survey, workforce interviews and costings data.

**Results**

The overall aim of the HLCC evaluation is to determine if flexible funding enables health services to develop and implement alternative models to inpatient acute care that provide better experiences and outcomes for patients with chronic conditions, at equal or lower cost.

**Conclusions**

This session will provide an overview of HLCC and aims to describe key challenges and learnings of the trial from three different perspectives:

- DHHS - policy developers/funders: Implications of a pragmatic approach to implementation will be discussed, including the pros and cons of having implemented the trial at several health services and allowing tailored intervention models.
- Health Services - implementers: Results from qualitative focus groups assessing workforce perceptions of key barriers and enablers to implementation will be presented.
- CSIRO - evaluators: The feasibility of evaluation frameworks in a data driven environment will be discussed, including how data lags, data availability and outcome measures impact reporting and short-term policy decisions.

**References**

1. Calver J, Brameld KJ, Preen DB, Alexia SJ, Boldy DP, McCaul KA. High-cost users of hospital beds in Western Australia: a population-based record linkage study. Med J Aust. 2006;184:393–397.

2. Glasgow, RE, Vogt, TM, Boles, SM. Evaluating the public health impact of health promotion interventions: the RE-AIM framework. Am J Public Health. 1999;89:1322-1327

3. Underwood AJ. Beyond BACI: Experimental designs for detecting human environmental impacts on temporal variations in natural populations. Aust. J. Mar. Freshwater Res.1991;42:569-587

## A48 The Department of Defense Practice-Based Implementation Network: developing a framework for matching implementation strategies to barriers in complex healthcare systems

### Kimberly Pratt, Briana Todd, Angela Gray, Jorielle Houston

#### Psychological Health Center of Excellence, Defense Health Agency, Silver Spring, MD, USA

##### **Correspondence:** Kimberly Pratt (kimberly.m.pratt5.ctr@mail.mil)

**Background**

Integrating evidence-based practices into clinical care is essential for mission readiness. However, research demonstrates, that despite the availability of effective clinical interventions, service members frequently do not receive guideline-concordant care that reflects the uptake of the most recent scientific advancements [1]. To improve the access, quality, effectiveness, and efficiency of psychological healthcare for veterans and service members, the Department of Defense (DoD) established the Practice-Based Implementation (PBI) Network. The PBI Network engages healthcare administrators and providers in implementation pilots to evaluate the feasibility and acceptability of DoD-wide implementation of an evidence-based clinical practice, and develops solutions to any implementation challenges that could impede successful uptake and sustained use of the practice change. The purpose of this presentation is to review the PBI Network’s process for mapping implementation strategies to barriers in the Military Health System (MHS).

**Materials and Methods**

This presentation will review a case study showcasing the PBI Network’s efforts to partner with DoD leadership, clinicians, and researchers to enhance access to evidence-based practices in the MHS. The case study will demonstrate the PBI Network’s use of Intervention Mapping (IM) [2] guided by the Consolidated Framework for Implementation Research (CFIR) [3] and the Integrated Promoting Action on Research in Health Services (i-PARIHS) [4] frameworks to develop an implementation program that strategically addresses the unique challenges facing military health providers and leaders.

**Results**

Time constraints, multiple competing demands, and limited resources [5-6] are significant barriers to the uptake and adoption of evidence-based practices in the MHS. Many implementation strategies can be leveraged to overcome these barriers and effectively implement evidence-based practices in the MHS.

**Conclusions**

Implementation of evidence-based intervention is a complicated process. It has been suggested that implementation strategies should be selected and tailored to address the contextual needs of specific efforts; however, there is limited guidance as to how to do this. The PBI Network’s experiences provide insight into methodologies for mapping implementation strategies to address the complex barriers to implementation in the MHS which may have broad application to other similar complex healthcare systems.

**References**

1. Institute of Medicine. Treatment for Posttraumatic Stress Disorder in military and veteran populations: final assessment. National Academies Press. 2014.

2. Bartholomew LK, Parcel GS, Kok G, Gottlieb NH. Intervention mapping: designing theory and evidence-based health promotion programs Mountain View: Mayfield Publishing Company; 2001.

3. Damschroder LJ, Aron DC, Keith RE, Kirsh SR, Alexander JA, Lowery JC. Fostering implementation of health services research findings into practice: a consolidated framework for advancing implementation science. Implement Sci. 2009;4:50.

4. Harvey G, Kitson A. PARIHS revisited: from heuristic to integrated framework for the successful implementation of knowledge into practice. Implement Sci. 2016;11:33.

5. Cook JM, Dinnen S, Thompson R, Ruzek J, Coyne JC, Schnurr PP. A quantitative test of an implementation framework in 38 VA residential PTSD programs. Adm Policy Mental Health. 2016;42(4):462-473.

6. Sadeghi‐Bazargani H, Tabrizi JS, Azami‐Aghdash S. Barriers to evidence‐based medicine: a systematic review. Journal of Eval Clin Pract. 2016;20(6):793–802.

## A49 Optimizing public health interventions by using mechanistic evaluations: a case example from a school-based physical activity implementation trial

### Hopin Lee^1^, Nicole Nathan^2^, Kirsty Hope^2^, Luke Wolfenden^2^

#### ^1^University of Oxford, Oxford, England, UK; ^2^University of Newcastle, Newcastle, New South Wales, Australia

##### **Correspondence:** Hopin Lee (hopin.lee@ndorms.ox.ac.uk)

**Background**

Public health implementation strategies often comprise of multiple components that are combined and delivered as a complex intervention [1]. Within a complex social-ecological system, there are multiple mechanisms by which an intervention could have its effect on the distal implementation outcome. To successfully implement health policies, the implementation strategy must collectively have a causal effect on the mechanisms that drive successful implementation [2]. Understanding these mechanisms are critical to optimizing and scaling complex implementation strategies [3]. The study aim was to understand the mechanisms of a complex implementation strategy on increasing physical activity minutes scheduled by school teachers in New South Wales, Australia [4].

**Materials and Methods**

We conducted a causal mediation analysis [5-6] of a cluster randomised controlled trial conducted in 62 primary schools. Schools were randomly allocated to receive either a complex implementation strategy that included; obtaining executive support, provision of tools and resources, implementation prompts, reminders and feedback; or usual practice. The primary trial outcome was the average minutes of physical activity scheduled by teachers across the school week at 12 months. We estimated path specific effects and average indirect and direct effects of the implementation strategies through four putative mechanisms.

**Results**

The analysis included 62 schools comprising of 215 teachers in the intervention arm and 181 teachers in the control arm. The intervention had a positive effect on knowledge (0.30 [95% CI: 0.15 to 0.46]), environmental context and resources (0.50 [0.31 to 0.69]), social influences (0.18 [0.01 to 0.35]) but did not have an effect on beliefs about consequences (0.07 [-0.03 to 0.17]). All putative mediators were not associated with the primary outcome.

**Conclusions**

Although the implementation strategy caused meaningful improvements in scheduled minutes of physical activity, this effect was not mediated by targeted mechanisms. Future research should explore the role of other potential mechanisms and evaluate system-level mechanisms informed by an ecological framework.

**References**

1. Moore G, Audrey S, Barker M, Bond L, Bonell C, Cooper C, Hardeman W, Moore L, O’Cathain A, Tinati T, Wight D, Baird J. Process evaluation in complex public health intervention studies: the need for guidance. J Epidemiol Community Health. 2014;68(2):101-2. doi:10.1136/jech-2013-202869.

2. Weiner BJ, Lewis MA, Clauser SB, Stitzenberg KB. In search of synergy: strategies for combining interventions at multiple levels. J Natl Cancer Inst – Monogr. 2012;(44):34-41. doi:10.1093/jncimonographs/lgs001.

3. Powell BJ, Fernandez ME, Williams NJ, Aarons GA, Beidas RS, Lewis CC, McHugh SM, Weiner BJ. Enhancing the impact of implementation strategies in healthcare: a research agenda. Front Public Health. 2019;7(3). doi:10.3389/fpubh.2019.00003.

4. Nathan N, Wiggers J, Bauman AE, Rissel C, Searles A, Reeves P, Oldmeadow C, Naylor PJ, Cradock AL, Sutherland R, Gillham K, Duggan B, Chad S, McCarthy N, Pettett M, Jackson R, Reilly K, Herrmann V, Hope K, Shoesmith A, Wolfenden L. A cluster randomised controlled trial of an intervention to increase the implementation of school physical activity policies and guidelines: study protocol for the physically active children in education (PACE) study. BMC Public Health. 2019;19(1):170. doi:10.1186/s12889-019-6492-z.

5. Lee H, Herbert RD, Mcauley JH. Mediation Analysis. JAMA. 2019; 321(7):697-698. doi:10.1001/jama.2018.21973.

6. Imai K, Keele L, Tingley D. A general approach to causal mediation analysis. Psychol Methods. 2010;15(4):309-334. doi:10.1037/a0020761.

## A50 How policy mandates for evidence-based practices filter into clinical routines: a mixed methods study

### Lorella Palazzo^1^, Peter Mendel^2^, Kelli Scott^3^, Cara C. Lewis^1^

#### ^1^Kaiser Permanente Washington Health Research Institute, Seattle, WA, USA; ^2^RAND Corporation, Santa Monica, CA, USA; ^3^Brown University School of Public Health, Providence, RI, USA

##### **Correspondence:** Lorella Palazzo (lorella.g.palazzo@kp.org)

**Background**

Federal and state policies mandating evidence-based practice (EBP) implementation in community mental health settings are increasingly common. Measurement based care (MBC), the use of progress/outcome monitoring measures to guide treatment [1], is one such EBP mandated for use. It is crucial to understand the process by which mandates are received by organizations, as contextual factors may influence mandate implementation success. The goal of this study is to employ mixed methods to characterize how a mandate plays out in community mental health settings that implement MBC using the Patient Health Questionnaire-9 (PHQ-9).

**Materials and Methods**

We utilized data from a cluster randomized trial comparing tailored vs standardized approaches to implementing the PHQ-9 in community mental health settings in two states [2]. Qualitative data were collected through semi-structured interviews with community mental health clinicians (N = 36). Interview questions covered MBC domains (e.g. clinicians’ attitudes towards and usage of MBC practices with the PHQ-9). Quantitative data were obtained from clinician surveys and included demographics and contextual constructs (e.g. norms, structures, processes) known to influence implementation.

**Results**

Data have been collected and are undergoing case-based analysis that relies on: 1. Qualitative thematic analysis to capture clinician descriptions of mandate implementation; 2. Cross-case analysis of qualitative and quantitative data to derive site-level indicators; 3. Qualitative Comparative Analysis (QCA) to identify contextual factors associated with mandate implementation outcomes. [3]

**Conclusions**

Organizations implementing policy mandates for EBPs should consider how site-specific contextual drivers may interact with implementation efforts, affect mandate outcomes, and need to be addressed through targeted implementation strategies.

**Trial registration:** ClinicalTrials.gov NCT02266134

**References**

1. Scott K, Lewis CC. Using measurement-based care to enhance any treatment. Cogn Behav Pract. 2015;22(1):49-59.

2. Lewis CC, Scott K, Marti CN, Marriott BR, Kroenke K, Putz JW, Mendel P, Rutkowski S. Implementing measurement-based care (iMBC) for depression in community mental health: a dynamic cluster randomized trial study protocol. Implement Sci. 2015;10:127.

3. Mendel P, Chen EK, Green HD, Armstrong C, Timbie JW, Kress AM, Friedberg MW, Kahn KL. Pathways to medical home recognition: a qualitative comparative analysis of the PCMH transformation process. Health Ser Res. 2018;53(4):2523-2546.

## A51 Understanding the implementation of evidence-informed policies and practices from a policy perspective: a critical interpretive synthesis

### Heather L. Bullock, John N. Lavis, Michael G. Wilson, Gillian Mulvale, Ashleigh Miatello

#### McMaster University, Hamilton, ON, Canada

##### **Correspondence:** Heather L. Bullock (bullochl@mcmaster.ca)

**Background**

The fields of implementation science and knowledge translation have evolved somewhat independently from the field of policy implementation research, despite calls for better integration [1]. As a result, implementation theory and empirical work do not often reflect the implementation experience from a policy lens nor benefit from the scholarship in all three fields. This means policy makers, researchers and practitioners may find it challenging to draw from theory that adequately reflects their implementation efforts.

**Materials and Methods**

We developed an integrated theoretical framework of the implementation process from a policy perspective by combining findings from these fields using the critical interpretive synthesis method [2]. We began with the compass question: how is policy currently described in implementation theory and processes and what aspects of policy are important for implementation success? We then searched 12 databases as well as grey literature and supplemented these documents with other sources to fill conceptual gaps. Using a grounded and interpretive approach to analysis, we built the framework constructs and used our findings to consider improvements to existing theory.

**Results**

A total of 7850 documents were retrieved and assessed for eligibility and 34 additional documents were identified through other sources. Eighty-two unique documents were ultimately included in the analysis. Our findings indicate that policy is described as: 1) the context; 2) a focusing lens; 3) the innovation itself; 4) a lever of influence; 5) an enabler/facilitator or barrier; or 6) an outcome. Policy actors were also identified as important participants or leaders of implementation. Our analysis led to the development of a two-part conceptual framework, including process and determinant components. We also used our findings to modify the Interactive Systems Framework for Dissemination and Implementation [3]. Finally, we provide an example of how the framework can be applied using a policy implementation case from Ontario, Canada.

**Conclusions**

This framework begins to bridge the divide between disciplines and offers a new way of thinking about implementation processes at the systems level.

**References**

1. Nilsen P, Stahl C, Roback K, Cairney P. Never the twain shall meet? A comparison of implementation science and policy implementation research. Implement Sci. 2013;8:63.

2. Dixon-Woods M, Cavers D, Agarwal S, Annandale E, Arthur A, Harvey J, Hsu R, Katbamna S, Olsen R, Smith L, Riley R, Sutton AJ. Conducting a critical interpretive synthesis of the literature on access to healthcare by vulnerable groups. BMC Med Res Methodol. 2006;6(1):35.

3. Wandersman A, Duffy J, Flaspohler P, Noonan R, Lubell K, Stillman L, Blachman M, Dunville R, Saul J. Bridging the gap between prevention research and practice: the interactive systems framework for dissemination and implementation. Am J Community Psychol. 2008;41(3-4):171-81.

## A52 Integrating research, policy, and practice to implement the largest suicide risk identification strategy in a United States healthcare system

### Bridget Matarazzo, Nazanin Bahraini, Suzanne McGarity, Megan Harvey, Lisa Brenner

#### Rocky Mountain MIRECC for Suicide Prevention, Denver, CO, USA

##### **Correspondence:** Bridget Matarazzo (bridget.matarazzo@va.gov)

**Background**

Research suggests that a significant number of individuals who died by suicide were not identified as psychiatric patients nor were they receiving mental health care; rather, they were often seen in primary care, ED or other medical settings before their death [1-3]. In response to these findings and a Joint Commission Sentinel Event Alert [4], in October 2018, the Department of Veterans Affairs (VA) launched the VA Suicide Risk Identification Strategy (VA Risk ID), the largest population-based screening and evaluation strategy in any United States healthcare system. Successful implementation of VA Risk ID relies on collaboration between researchers, policy makers, and supervisors and providers within the field.

**Materials and Methods**

Consistent with the Evidence-Based System for Innovation Support Logic Model [5], the VA Risk ID implementation team combined tools, training and technical assistance (TA) with a quality assurance measure to develop a robust support system for implementation. Proactive TA is delivered via weekly conference calls (~250 attendees/week) and a support email address (~250 emails/month). A SharePoint site which houses a variety of tools developed for VA Risk ID is also utilized. The team also conducted a webinar series, offering training on the overall strategy and practice components, which was converted into VA online learning system trainings. A fallout report was developed for quality assurance, which provides information about patients who did not receive indicated levels of the screening and evaluation process.

**Results**

The above strategies allowed the implementation team to get real-time feedback from the field, which was then communicated directly to VA policy makers on a weekly basis. As a result, major alterations have been made to the VA Risk ID implementation timeline and requirements.

**Conclusions**

The presenters will discuss how VA Risk ID implementation serves as a rich and useful example of how research, policy, implementation science and practice inform one another to result in the successful implementation of the largest suicide risk screening and evaluation strategy in any United States healthcare system.

**References**

1. Luoma JB, Martin CE, Pearson JL. Contact with mental health and primary care providers before suicide: a review of the evidence. Am J Psychiatry. 2002;159:909–916.

2. Gairin I, House A, Owens D. Attendance at the accident and emergency department in the year before suicide: retrospective study. Br J Psychiatry. 2003;183:28–33.

3. Denneson LM, Kovas AE, Britton PC, Kaplan MS, McFarland BH, Dobscha SK. Suicide risk documented during veterans last Veterans Affairs health care contacts prior to suicide. Suicide Life Threat Behav. 2016;46(3):363-374. doi:10.1111/sltb.12226.

4. The Joint Commission. Detecting and treating suicide ideation in all settings. Sentinel Event Alert. 2016;56:1-7.

5. Wandersman A, Chien VH, Katz J. Toward an evidence-based system for innovation support for implementing innovations with quality: tools, training, technical assistance, and quality assurance/quality improvement. Am J Comm Psychol. 2012;50(3-4):445-459. doi:10.1007/s10464-012-9509-7.

## A53 The role of implementation science in achieving health equity

### Chair: Amanda Farley^7^, Discussant: Lisa Saldana^8^, Panelists: Allison Metz^1^, Ana Baumann^2^, Leopoldo Cabassa^2^, Kimberly DuMont^3^, Beadsie Woo^4^, JD Smith^5^, Inger Burnett-Zeigler^5^, Juan Villamar^5^, Carlos Gallo^5^, Hendricks Brown^5^, Moira McNulty^6^

#### ^1^University of North Carolina, Chapel Hill, Chapel Hill, NC, USA; ^2^Brown School, Washington University at St. Louis, St. Louis, MO, USA; ^3^William T. Grant Foundation, New York, NY, USA; ^4^Annie E. Casey Foundation, Baltimore, MD, USA; ^5^Northwestern University, Evanston, IL, USA; ^6^University of Chicago, Chicago, IL, USA; ^7^University of Birmingham, Birmingham, England, UK; ^8^Oregon Social Learning Center, Eugene, OR, USA

##### **Correspondence:** Allison Metz (allison.metz@unc.edu)

**Background**

Inequities in healthcare are unfair differences between populations in the access, use, quality and outcomes of care. These inequities are persistent, detrimental and costly. Implementation science has great potential to improve the health of communities and individuals that experience disparities. Equitable implementation occurs when strong equity components (including explicit attention to culture, history, values, and needs of the community) are integrated into the principles and tools of implementation science to facilitate quality implementation of effective programs for a specific community or group of communities.

**Materials and Methods**

This presentation includes two approaches to using implementation methods and frameworks to address healthcare inequities. The first approach uses Proctor et al. [1] framework to reframe five elements of implementation science to: 1) focus on reach from the very beginning; 2) design and select interventions for vulnerable populations with implementation in mind; 3) implement what works and develop implementation strategies that can help reduce inequities in care; 4) develop the science of adaptations; and 5) use an equity lens for implementation outcomes.

The second approach discusses three innovative implementation method paradigms to improve scientific and health equity: 1) making efficient use of existing data by applying epidemiologic and simulation modeling to understand what drives disparities and how they can be overcome; 2) designing new research studies that include, but do not focus exclusively on populations experiencing disparities in such areas as cardiovascular disease and co-occurring mental health conditions; and 3) research that focuses exclusively on populations that have experienced high levels of disparities.

**Results**

Conceptual approaches will initiate a much-needed dialogue on how to critically infuse an equity approach in implementation science to proactively address healthcare inequities. These approaches raise numerous barriers for implementation research and how they can exacerbate disparities [2]. This work extends examples in behavioral health [3].

**Conclusions**

Discussion will center on themes for taking action to ensure implementation science amplifies equity. Themes were identified through structured facilitations with 21 researchers and include: employ strategies and build structures that elevate equity concerns; shift funding incentives to value practice expertise and questions; and promote exchanges between researchers and community members.

**References**

1. Proctor E, Silmere H, Raghavan R, Hovmand P, Aarons G, Bunger A, Griffey R, Hensley M. Outcomes for implementation research: conceptual distinctions, measurement challenges, and research agenda. Adm Policy Ment Health. 2011;38(2):65-76.

2. Woodward EN, Matthieu MM, Uchendu US, Rogal S, Kirchner J.E. The health equity implementation framework: proposal and preliminary study of hepatitis C virus treatment. Implement Sci. 2019;14(1), 26.

3. McNulty M, Smith JD, Villamar J, Burnett-Zeigler I, Vermeer W, Benbow N, Gallo C, Wilensky U, Hjorth A, Mustanki B, Schneider J. Implementation research methodologies for achieving scientific equity and health equity. Ethn Dis. 2019;29(Suppl 1):83-92.

## A54 Economic evaluation of implementation strategies: making the business case for implementation science in the real world

### Amy M. Kilbourne^1,2^, Andria Eisman^1^, Daniel Eisenberg^1^

#### ^1^University of Michigan, Ann Arbor, MI, USA; ^*2*^Quality Enhancement Research Initiative, U.S. Department of Veterans Affairs, Ann Arbor, MI, USA

##### **Correspondence:** Amy M. Kilbourne (amykilbo@umich.edu)

**Background**

Implementation strategies are methods used to help provider organizations deploy evidence-based practices. To date, few studies have compared the effectiveness of different implementation strategies, and rarely have they assessed economic impact. Estimating costs of implementation strategies – and comparing those costs to value generated – is crucial if health care providers are to make informed decisions about investment in specific strategies to improve uptake of effective practices.

**Materials/Methods**

We describe two studies involving economic evaluations of the Replicating Effective Programs (REP) implementation strategy. Both studies had adaptive designs in which sites that did not respond to 6 months of REP were randomized to receive additional implementation support (i.e., Facilitation). First, Re-Engage was an adaptive implementation trial comparing REP alone to Enhanced REP (added External Facilitation) to enhance the uptake of a brief care management program for Veterans with serious mental illness among a national cohort of 88 VA facilities. Second, the Adaptive Implementation of Effective Programs Trial (ADEPT) was a cluster-randomized sequential multiple assignment randomized trial (SMART) trial that compared REP only to REP adding External Facilitation or External+Internal Facilitation to improve the uptake of a collaborative care model for mood disorders across 57 community practices in Michigan and Colorado.

**Results**

In the first study, the rate of Re-Engage uptake (number of attempted patient contacts) was greater for enhanced REP sites compared with standard REP sites (41% versus 31%, p=.01). An initial cost analysis found that the additional time cost of Facilitation was 7.3 hours per site, or ~$2500 per 6-month dose of Facilitation. In ADEPT, patients at sites receiving External Facilitation alone compared to External+Internal Facilitation had improved SF-12 and mood symptom scores and higher odds of receiving collaborative care. The added costs of Internal Facilitation did not lead to greater implementation value, suggesting that REP plus External Facilitation was the most cost-effective combination of implementation strategies.

**Conclusions**

We discuss strengths and limitations of the economic evaluations performed in these studies. We also highlight how adaptive and SMART designs are practical ways to assess the costs of implementation strategies and inform more efficient use of these strategies.

**Trial registration:** Current Controlled Trials ISRCTN21059161; ClinicalTrials.gov NCT02151331

## A55 Use of discrete choice experiments to inform stakeholder decision-making about implementation

### Ramzi Salloum^1^, Elizabeth Shenkman^1^, Stephanie Staras^1^, Jordan Louviere^2^, David Chambers^3^

#### ^1^University of Florida, Gainesville, FL, USA; ^*2*^University of South Australia, Adelaide, South Australia, Australia; ^*3*^National Cancer Institute, Bethesda, MD, USA

##### **Correspondence:** Ramzi Salloum (rsalloum@ufl.edu)

**Background**

The discrete choice experiment (DCE) is a stated preference technique from health economics for eliciting individual preferences over hypothetical alternative scenarios. This dynamic approach can be used to systematically measure the health preferences of various stakeholders – such as patients, providers, and administrators – and thus engage those stakeholders’ perspectives in decisions about investing time, money, and resources into implementation. The purpose of this presentation is to discuss the application of DCEs as a stakeholder engagement strategy to inform implementation of evidence-based interventions in health.

**Materials and Methods**

This presentation will cover findings from a recent systematic review by Salloum et al. [1] reporting on DCE applications in implementation research. In addition, the presentation will include examples of DCE applications from cancer prevention and control (e.g., tobacco control and HPV vaccination) that vary by application type. The presentation will discuss considerations for designing and conducting DCEs in implementation research at each of the following stages: (1) identify and characterize alternative scenarios; (2) experimental design to determine choices; (3) data collection; and (4) data analysis and interpretation.

**Results**

DCE applications in implementation research can be categorized into four types: (1) characterizing demand for therapies and treatment technologies; (2) comparing implementation strategies; (3) prioritizing interventions; and (4) incentivizing providers. An example of each application type will be presented. A variety of stakeholders can be engaged using DCEs, including healthcare providers, patients, caregivers, and healthcare administrators. DCEs can be conducted across settings and contexts, including clinical settings (inpatient and outpatient), community-based settings, and at the policy/population level.

**Conclusions**

The use of DCEs to inform implementation of health interventions has been growing in recent years. As DCEs are more widely used in health-related assessments, there is a wide range of applications for them in the area of stakeholder engagement. Using DCEs can inform stakeholder decision making and support successful investment into implementation of health interventions.

**Reference**

1. Salloum RG, Shenkman EA, Louviere JJ, Chambers DA. Application of discrete choice experiments to enhance stakeholder engagement as a strategy for advancing implementation: a systematic review. Implement Sci. 2017;12:140. doi:10.1186/s13012-017-0675-8

## A56 Modeling to learn: conserving staff time when comparing implementation alternatives via simulation

### Lindsey Zimmerman^1^, David Lounsbury^2^, Tom Rust^3^, Craig Rosen^1^, Rachel Kimerling^1^, Jodie Trafton^4^, Steven Lindley^5^, Andrew Holbrook^1^, Stacey Park^1^, Jane Branscomb^6^, Debra Kibbe^6^, James Rollins^7^, Savet Hong^1^

#### ^1^National Center for PTSD, Menlo Park, CA, USA; ^2^Albert Einstein College of Medicine, Bronx, NY, USA; ^3^Center for Healthcare Transformation, Boston, MA, USA; ^4^Program Evaluation Resource Center, Palo Alto, CA, USA; ^5^Veterans Affairs Palo Alto Health Care System, Palo Alto, CA, USA; ^6^Georgia Health Police Center, Georgia State University, Atlanta, GA, USA; ^7^Takouba Security LLC, Seattle, WA, USA

##### **Correspondence:** Lindsey Zimmerman (lindsey.zimmerman@va.gov)

**Background**

For over 15 years, VA has implemented national dissemination efforts to train providers in evidence-based addiction and mental health practices (EBPs). VA mandates EBPs and supports their implementation with substantial investment in infrastructure to support quality improvement (e.g., incentivized VA-wide quality measures), yet only 3-28% of the patient population receives the highest quality care. To improve EBP access, the National Center for PTSD developed a simulation-learning program designed to help frontline teams identify locally calibrated EBP improvement strategies [1]. We piloted participatory system dynamics (PSD) for improving EBP reach based on its effectiveness in business and engineering [2-3].

**Materials and Methods**

PSD synthesizes engagement principles and state of the science technologies for understanding and changing systems. As a systems science method, PSD demonstrates how causal system properties vary as a function of local resources (e.g., financial, personnel). Over the last four years, in partnership with patient, provider, and policy-maker stakeholders, we co-developed a PSD program entitled Modeling to Learn (MTL). MTL supports multidisciplinary frontline teams of providers to address high priority areas (e.g., Suicide Prevention, Access to Care, Opioid Misuse, PTSD) by conserving local staff time in simulation learning models that evaluate and compare implementation scenarios.

**Results**

MTL helped two pilot clinics increase EBP reach with existing local staff resources. Preliminary statistical process control analyses indicate pilot clinics demonstrated a three standard deviation increase in EBP reach and maintained improvement for 12 and 8 months, respectively. We will present example simulation experiments for different VA clinics, highlighting how simulation helps staff optimize local staff resources to meet patients’ needs.

**Conclusions**

Most cost analysis in implementation research focuses on the costs of EBP adoption. But, VA-adopted EBPs and VA budgets facilitate/constrain the resources needed to expand reach. We posit that MTL created greater consensus about change (front-end optimization) and guided more effective investment decisions within local system resources (back-end optimization). Additional study of the MTL national rollout is underway: a multisite cluster randomized trial will test the superiority of MTL over audit-and-feedback for its effectiveness improving the reach of EBPs.

**References**

1. Zimmerman L, Lounsbury DW, Rosen CS, Kimerling R, Trafton JA, Lindley SE. Participatory system dynamics modeling: increasing stakeholder engagement and precision to improve implementation planning in systems. Adm Policy Ment Health. 2016;43(6):1-16. doi:10.1007/s10488-016-0754-1

2. Sterman JD. Learning from evidence in a complex world. Am J Public Health. 2006;96:505–514. doi:10.2105/AJPH.2005.066043

3. Hovmand PS. Community Based System Dynamics. New York, NY: Springer New York; 2014.

## A57 Mixed-method approaches to strengthen economic and cost research methods in implementation science

### Alex R. Dopp^1^, Peter Mundey^2^, Lana O. Beasley^3^, Jane F. Silovsky^2^, Daniel Eisenberg^4^

#### ^1^RAND Corporation, Santa Monica, CA, USA; ^2^University of Oklahoma Health Sciences Center, Oklahoma City, OK, USA; ^3^Oklahoma State University, Stillwater, OK, USA; ^4^University of Michigan, Ann Arbor, MI, USA

##### **Correspondence:** Alex R. Dopp (adopp@rand.org)

**Background**

Guidance on the costs and economic impacts of implementing evidence-based practices is critical to informing investments in implementation efforts. However, the results of traditional methods – such as economic evaluations, discrete choice modeling, and participatory system dynamics modeling – are limited by a remaining “qualitative residual” of contextual information and stakeholders’ perspectives. This residual, which is particularly prevalent in implementation research, cannot be fully captured by the quantitatively-based analyses and models used in these methods. The emergence of qualitative methods for studying economics and costs offers a promising solution.

**Materials and Methods**

We recommend that researchers maximize their contributions related to economics and costs within implementation science by embracing a mixed-methods research agenda that merges traditional quantitative approaches with innovative, contextually grounded qualitative methods. Such studies are exceedingly rare at present.

**Results**

To assist implementation scientists in making use of mixed methods in this research context, we will present an adapted taxonomy that describes the structure and function mixed-method studies relevant to economic and cost research. We will then illustrate the application of mixed methods in exemplar studies that used economic evaluation, discrete choice modeling, or system dynamics methods to study implementation. The examples presented will emphasize the breadth of qualitative methods for data collection (e.g., interviews, focus groups, site visits, review of records, ethnography) and analysis (e.g., content analysis, thematic analysis, grounded theory, case studies) that can be incorporated into studies of implementation costs and economics. Finally, we will review reporting guidelines for these methods (e.g., Consolidated Health Economic Evaluation Reporting Standards) with an emphasis on how to incorporate mixed methods into existing guidelines.

**Conclusions**

By incorporating qualitative methods, implementation researchers can enrich their research on economics and costs with detailed, context-specific information to tell the full story of the economic impacts of implementation. We will end by providing suggestions for building a research agenda in mixed-method economic evaluation, along with more resources and training to support investigators who wish to answer our call to action.

## A58 Developing the adaptation-impact model and translating it for use in practice

### M. Alexis Kirk^1^, Julia E. Moore^2^, Byron J. Powell^3^, Sarah Birken^1^

#### ^1^University of North Carolina at Chapel Hill, Chapel Hill, NC, USA; ^2^The Center for Implementation, Toronto, ON, Canada; ^3^Washington University in St. Louis, St. Louis, MO, USA

##### **Correspondence:** M. Alexis Kirk (akirk@unc.edu)

**Background**

Implementation science is shifting from qualifying adaptations as good or bad towards understanding nuances of adaptations and their impact. Existing adaptation classification frameworks are largely descriptive (e.g., who made the adaptation) and geared towards researchers. They do not help practitioners in decision-making around adaptations – is an adaptation likely to have negative impacts? Should it be pursued? Moreover, they lack constructs to consider potentially disparate impact on intervention and implementation outcomes (e.g., whether an adaptation might improve implementation outcomes but weaken intervention outcomes).

**Materials and Methods**

We consolidated two adaptation frameworks [1-2] and one intervention-implementation outcome framework [3]. We reviewed each framework to refine constructs and group them into domains. We then coded qualitative descriptions of 14 adaptations from an existing intervention being adapted to a new context to test fit of our framework. To bridge the research-practice gap, we then developed guidance to help practitioners in the field apply this framework to adaptation efforts.

**Results**

Our framework has 3 domains and our applied guidance for each domain is as follows:

Criteria for making adaptations: useful in considering whether adaptations will have a positive or negative impact to help decide whether to move forward with the adaptation. Systematic adaptations with a positive valence are more likely to have a positive impact.

Impact of adaptations: useful in considering adaptations’ impact on intervention outcomes (intervention effectiveness) and implementation outcomes (acceptability, cost, etc.). to help anticipate and mitigate negative consequences of adaptations (e.g., plan for implementation of an adaptation that might improve intervention effectiveness but decrease acceptability).

Adaptation Typology: classifies adaptation attributes (e.g., type, nature of adaptation), providing consistency in reporting

**Conclusions**

Our guidance takes a consolidated framework developed for research and translates it to be helpful to practitioners. Our guidance helps practitioners “back-up” and re-think adaptations suspected to have negative impacts and helps practitioners think through “ripple effects” and tradeoffs of adaptations to plan accordingly (e.g., put in place an implementation strategy to monitor/boost fidelity if an adaptation is suspect to have positive impacts on acceptability, but negative impacts on fidelity).

**References**

1. Moore JE, Bumbarger BK, Cooper BR. Examining adaptations of evidence-based programs in natural contexts. J Prim Prev. 2013:34(3):147-161.

2. Stirman SW, Miller CJ, Toder K, Calloway A. Development of a framework and coding system for modifications and adaptations of evidence-based interventions. Implement Sci 2013:8(1):65.

3. Proctor EK, Silmere H, Raghavan R, Hovmand P, Aarons G, Bunger A, Griffey R, Hensley M. Outcomes for implementation research: conceptual distinctions, measurement challenges, and research agenda. Adm Policy Ment Health. 2011;38(2):65-76.

## A59 Striking the right balance: tracking adaptations to community-based prevention programs to enhance guidance to implementers

### Brittany Cooper, Garrett Jenkins, AnaMaria Diaz Martinez

#### Washington State University, Pullman, WA, USA

##### **Correspondence:** Brittany Cooper (brittany.cooper@wsu.edu)

**Background**

The adoption of effective programs is insufficient for achieving positive youth and family outcomes community-based organizations seek; high quality implementation is also critical. However, making decisions about dosage, content, and structure of an evidence-based program as it was originally designed (i.e., fidelity) while adapting to local contexts is challenging and complex, especially under resource strain. Implementers are often left to make these decisions without much empirically-based guidance.

**Materials and Methods**

We used data from an ongoing, large-scale evaluation of Strengthening Families Program (SFP; a 7-week substance use prevention program with youth 10-14 years old and their parents) implemented in natural contexts across Washington State. Our previous work applied and extended two multidimensional coding systems [1-2] to 154 implementer-reported adaptations of SFP [3]. Based on these results, we designed a quantitative measure to track the extent to which implementers modified, added, and deleted program content/processes; the most common types and reasons for adaptations; and the extent to which adaptations proactive or reactive.

**Results**

Preliminary results from 28 SFP implementations show that over half report modifying the program content/processes “a little”, but 60% report “not at all” when asked about addition or deletion of content/processes. The most commonly reported modifications were made to games and activities/icebreakers, and the most commonly reported reason was lack of time/competing demands on time. “Need for a more culturally appropriate program” was reported by 24% despite 39% of implementations being delivered in Spanish or bilingually. Planning in advance “some” or “a lot” for adaptations was reported by 37%, whereas 15% reported “some” or “a lot” for making reactive adaptations.

**Conclusions**

These results help describe the adaptations being made and how/why they are being made. Future analyses will examine links between these dimensions of adaptation and participant engagement, which increasingly is shown to be a critical mediator/moderator between adaptations and program outcomes. Ultimately, this work will help develop a theoretically- and empirically-grounded tool to track real-world adaptations and their impacts, in turn enhancing program implementation guidance on how to strike the right balance between program fidelity and adaptation.

**References**

1. Hill LG, Maucione K, Hood BK. A focused approach to assessing program fidelity. Prev Sci. 2007; 8(1):25-34.

2. Moore JE, Bumbarger BK, Cooper BR. Examining adaptations of evidence-based programs in natural contexts. J Prim Prev. 2013;34(3):147-161.

3. Cooper BR, Shrestha G, Hyman L, Hill LG. Adaptations in a community-based family intervention: replication of two coding schemes. J Prim Prev. 2016;37:33-52.

## A60 Rapid adaptation: making adaptations work for real world systems, services, and science

### Sarah Cusworth Walker^1^, Michael Graham-Squire^2^

#### ^1^University of Washington, Seattle, WA, USA; ^2^Neighborhood House, Seattle, WA, USA

##### **Correspondence:** Sarah Cusworth Walker (secwalkr@uw.edu)

**Background**

To reach scale, public health agencies and researchers will need to partner in new ways to meet the prevention needs of diverse and dynamic communities. Building from concepts proposed in the Dynamic Adaptation Framework [1], common elements [2], and adaptation models [3], we present preliminary results from a codesign process developed to provide tailored, rapid guidance for delivering more culturally congruent prevention services. We present data from a six-month codesign demonstration project in Seattle, WA between the University of Washington and a local social services agency with over 100 years of delivering social welfare and prevention services. The model is proceeding in four stages with distinct data capture at each phase: 1) Identify areas of strain; 2) Derive Core Elements; 3) Develop guidance; 4) Test acceptability.

**Materials and Methods**

We present data on the need for adaptation and the feasibility of the codesign model from the 21 facilitator respondents and the feasibility of the codesign process from community and research participants. Strain was assessed through surveys distributed to 21 GGC facilitators. The surveys asked respondents to score each activity in the curriculum for ease of delivery and need for adaptation. Qualitative responses were coded using the theoretical framework of structure and content adaptations [3].

**Results**

The fidelity of delivery to specific activities within GGC varied significantly, with some activities rarely delivered according to manualized instructions and other always or almost always delivered as written (range of 2.05-4.48 on a 5-point scale with 5 keyed to “always”). Activities rarely delivered as written were more likely to be experiential activities (e.g., group sculpture or scavenger hunt). Activities delivered as intended but rated as poor on cultural responsivity included videos and scripted content delivered by facilitators.

**Conclusions**

A number of structural and content adaptations needs were indicated, including the need to address divergent cultural views about some core assumptions of the GGC model.

**References**

1. Aarons GA, Green AE, Palinkas LA, Self-Brown S, Whitaker DJ, Lutzker JR, Silovsky JP, Hecht DB, Chaffin MJ. Dynamic adaptation process to implement an evidence-based child maltreatment intervention. Implement Sci. 2012;7(1):32-40.

2. Lyon AR, Lau AS, McCauley E, Vander Stoep A, Chorpita BF. A case for modular design: implications for implementing evidence-based interventions with culturally diverse youth. Prof Psychol Res Pract. 2014;45(1):57-66.

3. Stirman SW, Miller CJ, Toder K, Calloway A. Development of a framework and coding system for modifications and adaptations of evidence-based interventions. Implement Sci. 2013;8:65.

## A61 What fidelity data don’t say: types of adopters and resisters in an implementation trial in early care and education classrooms

### Taren Swindle^1^, Julie Rutledge^2^, Geoffrey Curran^1^

#### ^1^University of Arkansas for Medical Sciences, Little Rock, AR, USA; ^2^Louisiana Tech University, Ruston, LA, USA

##### **Correspondence:** Taren Swindle (tswindle@uams.edu)

**Background**

Together, We Inspire Smart Eating (WISE) is a nutrition promotion intervention designed for delivery by early care and education teachers. Data from a prior implementation of WISE showed suboptimal fidelity to its four, key evidence-based practices (i.e., hands-on exposure to fruits and vegetables (FV), use of mascot to promote FV, positive feeding practices, and role modeling). We are currently testing strategies to support uptake of WISE. This study presents preliminary findings on the behaviors observed by teachers.

**Materials and Methods**

A two-arm implementation trial is ongoing in 40 classrooms to implement WISE with a focus on its evidence-based practices. All classrooms are observed on a quarterly basis for fidelity by data collectors trained to 85% reliability. Classrooms in the treatment condition (i.e., enhanced support, N = 17) receive targeted implementation support based on their observed fidelity, which included facilitation. After the first and second quarter of the school year, 5 classrooms from the pool of poorest fidelity performers in the enhanced condition were randomly selected for semi-structured interviews. To date, the second quarter of data collection, 6 months of enhanced support, and both rounds of interviews are complete. Facilitators derived types of adopters and resisters based on analysis of observational and interview data.

**Results**

Four types of adopters and resisters were identified: enthusiastic adopters (35%), over-adapting adopters (24%), soft resisters (17%), and hard resisters (24%). Enthusiastic adopters exhibited positive attitudes towards WISE and moderate to strong fidelity. Over-adapting adopters, while exhibiting positive attitudes toward WISE, made fidelity-inconsistent adaptations that were potentially detrimental (i.e., using mascot to shame children). Soft resisters demonstrated poor to moderate fidelity and showed lack of interest in adopting WISE or receiving facilitation support. Hard resisters were vocal about their complaints in adopting WISE and/or noticeably against receipt of facilitation support; most, but not all, hard resisters had poor fidelity.

**Conclusions**

There are nuances in low fidelity not captured by quantitative scores. Different types of resisters may be best served with different implementation strategies. For example, hard resisters may benefit from strategies to enhance motivation whereas over-adapting adopters may need support for goal setting and/or appropriate adaptations.

**Trial registration:** ClinicalTrials.gov NCT03075085

## A62 Development of an instrument for evaluating implementation efforts and benchmarking regarding person centered care

### Helena Fridberg^1^, Lars Wallin^1,2^, Catarina Wallengren^2^, Henrietta Forsman^1^, Anders Kottorp^3^, Malin Tistad^1^

#### ^1^Dalarna University, Falun, Sweden; ^2^Gothenburg University, Gothenburg, Sweden; ^3^Malmö University, Malmö, Sweden

##### **Correspondence:** Helena Fridberg (hfi@du.se)

**Background**

Policy makers across Sweden are pushing implementation of person centered care (PCC) in health care settings as a way to promote high quality health care across the country [1]. However, there is a lack of valid and reliable instruments to measure and compare patients’ experiences of PCC across health care settings and patient groups. As part of a research project aimed at investigating implementation strategies of PCC at a regional level, we set out to develop a generic instrument to measure patients’ experiences of PCC as an outcome of the implementation efforts [2, 3]. In this project, we collaborate with the Swedish association of local authorities and regions (SALAR) who are responsible for the administration of the largest patient survey in Sweden. When complete, the instrument will be available to healthcare regions for evaluating implementation and development of the PCC approach.

**Materials and Methods**

A mixed methods design is used, entailing the following phases: construction of a preliminary questionnaire based on questions from SALAR’s existing item pool, content validation of items via experts (patients, healthcare practitioners and researchers) in a Delphi study, cognitive interviewing with patients, data collection and psychometric evaluation through Rasch analyses. The last two phases are repeated in two rounds. Finally, the instrument will be handed over to SALAR who will be responsible for translation into seven languages, design of web and paper-based surveys, and procedures for recruitment of patients.

**Results**

Data collection for the first psychometric evaluation will end by mid-April. Preliminary results from the first phase of Rasch analyses are expected by the end of May. The results from the second phase are expected by the end of August. The finalized instrument will be launched by SALAR in the beginning of 2020.

**Conclusions**

We set out to develop a robust and psychometrically sound instrument to measure patients’ perceptions on PCC. The collaboration with SALAR has been fruitful and will greatly expand survey opportunities. We expect the instrument to be widely used for evaluating implementation efforts and benchmarking regarding PCC.

**References**

1. Ekman I, Swedberg K, Taft C, Lindseth A, Norberg A, Brink E, Carlsson J, Dahlin-Ivanoff S, Johansson IL, Kjellgren K, Lidén E, Öhlén J, Olsson LE, Rosén H, Rydmark M, Sunnerhagen KS. Person-centered care--ready for prime time. Eur J Cardiovasc Nurs. 2011;10(4):248-251.

2. Martinez RG, Lewis CC, Weiner BJ. Instrumentation issues in implementation science. Implement Sci. 2014;9:118.

3. Moore L. Britten N. Lydahl D, Naldemirci Ö, Elam M, Wolf A. Barriers and facilitators to the implementation of person centred care in different healthcare contexts. Scand J Caring Sci. 2017;31:662–673

## A63 Implementation practice track: where the rubber meets the road-- novel applications and adaptations of implementation tools and strategies in real world settings

### Robert Franks^1^, Jonathan Scaccia^2^

#### ^1^Judge Baker Children’s Center/Harvard, Boston, MA, USA; ^2^Wandersman Center, Philadelphia, PA, USA

##### **Correspondence:** Robert Franks (rfranks@jbcc.harvard.edu)

**Background**

There are many implementation and dissemination frameworks to help to bridge the gap between research and practice. As implementation science has grown, we must avoid the paradoxical gap between implementation research and implementation practice. This presentation will describe the collaboration between researchers and practitioners in the development and application of organizational readiness and the continued use of the R=MC2 heuristic in research, evaluation, and practice.

**Materials and Methods**

We will first briefly describe the development of a new definition and conceptualization of Organizational Readiness for understanding and facilitating the implementation of innovations into new settings. Derived from work in the Interactive Systems Framework, the R=MC2 model proposes that readiness is not a singular, static condition, but rather a collection of dynamic constructs that includes motivation, general capacity, and innovation-specific capacity. Readiness can be used to monitor and facilitate implementation over time. The Wandersman Center developed a measure (the Readiness Diagnostic Tool) with good initial psychometric validity to help gather comprehensive information about facilitators of a change effort.

**Results**

A goal in the development of the RDT was that it could be used in multiple settings and for different interventions. Therefore, in this joint presentation between a researcher and a practitioner, we will also briefly describe the adaptation and design process for using the RDT in distinct settings and a new study to rigorously validate the measure. The potential to adapt the readiness measure is key for the application of implementation science constructs in real-world conditions. We will talk about how practitioners negotiate tradeoffs in making implementation constructs more specialized and application to their specific setting. Using the RDT as an example, we will share how we collaboratively worked through these adaptation issues.

**Conclusions**

The adaptation process and lessons learned were bidirectional. We will discuss how applied findings in real world settings informed changes in the underlying theory and measure, specifically about what and how the constructs were useful. This presentation will highlight how theory and practice can inform one another in an iterative manner to help to move the science forward while promoting a deeper understanding of implementation facilitators.

## A64 How can implementation quality be evaluated? an example from a pilot initiative in Australian child and family services

### Correspondence: Vanessa Rose (vanessa.rose@ceiglobal.org)

#### Centre for Evidence & Implementation, Sydney, Australia

**Background**

High-quality program implementation is a pre-condition to program effectiveness. However, evaluation of the implementation process is rare, resulting in uncertainty around interpretation of impact evaluations with null effects (i.e. was the program ineffective, or implemented poorly?). We report on an implementation evaluation of the Victorian Government’s pilot of five manualized therapeutic programs for vulnerable families (four developed in the USA) across seven service provider agencies; the first evaluation of this nature and scope in Australia. The aim was to provide an indication of the comprehensiveness, pace and quality of program implementation to inform government decisions about if/ how such programs should be funded, implemented, supported and scaled.

**Materials and Methods**

A real-world mixed-methods observational study design was used. The Stages of Implementation Completion checklist assessed implementation pace and comprehensiveness [1]. Theory-based structured interviews were conducted with agency staff (N=29) to explore program appropriateness, acceptability and feasibility [2]. Implementation strategies were explored with manualized program purveyors [3]. Fidelity data were extracted from agency databases.

**Results**

Most agencies (n=6) were still in early implementation, having not yet achieved sustainability. Highly-concentrated and overlapping implementation activity was observed, reflective of funding pressures, putting implementation quality at risk. The programs were generally well-accepted, perceived as high-quality and a good fit. While most agency staff ‘believed in’ the programs, perceived appropriateness was compromised by the lack of adaptability for Aboriginal and Torres Strait Islander communities. Threats to feasibility included high demands on practitioners and lack of Australian-based implementation support (trainers, consultants). It was too early for valid fidelity assessments.

**Conclusions**

Policy-makers should afford agencies more time/resources to incorporate initiatives into ‘business as usual’. Ongoing monitoring of implementation outcomes is highly recommended to facilitate data-driven decisions about when to start impact evaluation (i.e. when sustainability is achieved, and fidelity has been demonstrated).

**References**

1. Chamberlain P, Brown CH, Saldana L. Observational measure of implementation progress in community based settings: the stages of implementation completion (SIC). Implement Sci. 2011;6(1):116

2. Damschroder LJ, Aron DC, Keith RE, Kirsh SR, Alexander JA, Lowery JC. Fostering implementation of health services research findings into practice: a consolidated framework for advancing implementation science. Implement Sci. 2009;4(1):50.

3. Powell BJ, Waltz TJ, Chinman MJ, Damschroder LJ, Smith JL, Matthieu MM, Proctor EK, Kirchner JE. A refined compilation of implementation strategies: results from the Expert Recommendations for Implementing Change (ERIC) project. Implement Sci. 2015;10:21.

## A65 Implementation science for depression interventions in low- and middle-income countries: a systematic review

### Bradley Wagenaar^1^, Wilson Hammett^1^, Courtney Jackson^1^, Dana Atkins^1^, Jennifer Belus^2^, Christopher Kemp^1^

#### ^1^University of Washington, Seattle, WA, USA; ^2^University of Maryland, College Park, MD, USA

##### **Correspondence:** Bradley Wagenaar (bwagen@uw.edu)

**Background**

Interventions to treat depression are demonstrating effectiveness across a range of low-resource settings globally. Significant investments are being made to decrease the gap between this evidence and its application at scale. Our objectives were to systematically review implementation research targeting depression interventions in low- and middle-income countries (LMICs) and critically assess coverage and scientific gaps.

**Materials and Methods**

PubMed, CINAHL, PsycINFO, and EMBASE were searched for evaluations of depression interventions in LMICs reporting at least one implementation outcome. Study- and intervention-level characteristics were abstracted.

**Results**

A total of 7,034 studies were screened, 589 were assessed for eligibility, and 59 studies published between 2003 and 2017 met inclusion criteria. Most studies were conducted in Sub-Saharan Africa (n=24; 40.7%), followed by South Asia (n=17; 28.8%), and Latin America and the Caribbean (n=12; 20.3%). The majority of studies (n=41; 69.5%) reported outcomes for a depression intervention that was implemented at the pilot/research phase. The majority of studies (n=35; 59.3%) focused on depressive interventions delivered at the facility level, with 22 (37.3%) delivered in the community. Thirty studies (50.9%) utilized non-specialized healthcare workers as the implementing agent. Primary depression intervention modalities were individual psychotherapy (n=20; 33.9%) and multicomponent interventions (n=20; 33.9%). Only 19 studies (32.2%) tested an implementation strategy, with the most common implementation strategy being revising professional roles (n=8; 42.1%). The most common implementation outcomes reported were acceptability (n=39; 66.1%), followed by feasibility (n=22; 37.3%), and fidelity (n=16; 27.1%). No study reported penetration, and only 3 (5.1%) reported adoption or sustainability.

**Conclusions**

Implementation research for depressive interventions in LMICs has focused largely on early-stage implementation outcomes. Most studies have the primary aim of testing evidence-based interventions under pilot researcher-controlled implementation. Future implementation research could prioritize the development and testing of implementation strategies to promote delivery of evidence-based depression interventions in routine care. This would include increased consideration of contextual factors, as well as later-stage implementation outcomes such as cost, penetration, and sustainability. Certain regions, such as Middle East and North Africa, East Asia and Pacific, and Europe and Central Asia could be prioritized for investments given the paucity of research.

## A66 Predicting quality improvement sustainability with artificial neural networks

### Tim Rappon^1^, Erica Bridge^2^, Alyssa Indar^2^, Whitney Berta^2^

#### ^1^University of Toronto, Toronto, ON, Canada; ^2^University of Toronto, Institute of Health Policy, Management and Evaluation, Toronto, ON, Canada

##### **Correspondence:** Tim Rappon (tim.rappon@mail.utoronto.ca)

**Background**

Quality Improvement (QI) initiatives are proposed as key vehicles to shift our health system from disease-focused, episodic care to comprehensive care for older adults living with chronic conditions. With the proportion of older Canadians set to double over the next two decades, the need for QI is pressing, yet there is a dearth of research on whether QI yields long-term changes in practice (sustainment) or benefits (sustainability) for older patients and residents [1]. Our study responds to Proctor et al.’s (2015) call to “test theories, frameworks and models for their ability to explain and predict sustainability” [2].

**Materials and Methods**

We employed a Kohonen self-organizing map (SOM) [3] (an artificial neural network) to identify factors associated with sustainment and sustainability for QI interventions. To create our training dataset, we searched Medline, PsychINFO and CINAHL for articles which reported on the long-term (1+ years post-implementation) sustainability of QI programs targeted to older adults. After screening 3127 abstracts, 54 articles were selected. Two coders independently extracted study characteristics, including organizational & clinical context, implementation & post-implementation strategies, and adaptations. We performed leave-one-out cross-validation using the extracted variables to assess the sensitivity and specificity of the self-organizing map’s predictions of sustainment and sustainability.

**Results**

The SOM achieved 38% sensitivity and 73% specificity for sustainment and 89% sensitivity and 42% specificity with respect to sustainability. The diagnostic odds ratio was not significant for sustainment but was 5.2 (statistically significant p<0.05) for sustainability. We also estimated the relative contribution of different determinants to the prediction of sustainment and sustainability by observing how their omission affected the predictive power of the SOM. Clinical targets, adaptations, post-implementation (sustainability) strategies, and organizational context were all significant predictors of sustainability; however, a larger training set would be required for a predictive model of sustainment and to generalize the SOM beyond QI initiatives in health care for older adults.

**Conclusions**

Our study presents a novel method for investigating relationships between (post-)implementation factors and sustainability, which could be extended (with a larger training dataset) to produce predictions of—and tailored recommendations for—intervention sustainment and sustainability.

**References**

1. Shelton RC, Cooper BR, Stirman SW. The sustainability of evidence-based interventions and practices in public health and health care. Annu Rev Public Health. 2018;39(1):55-76. doi:10.1146/annurev-publhealth-040617-014731.

2. Proctor E, Luke D, Calhoun A, McMillen C, Brownson R, McCrary S, Padek M. Sustainability of evidence-based healthcare: Research agenda, methodological advances, and infrastructure support. Implement Sci. 2015;10(88):1-13.

3. Kohonen T. Self-Organizing Maps. 3rd edition. Berlin: Springer; 2000.

## A67 Pragmatic measures for implementation research: development of the Psychometric and Pragmatic Evidence Rating Scale (PAPERS)

### Cameo F. Stanick^1^, Heather M. Halko^2^, Elspeth A. Nolen^3^, Byron J. Powell^4^, Caitlin N. Dorsey^5^, Kayne D. Mettert^5^, Bryan J. Weiner^3^, Melanie Barwick^6^, Luke Wolfenden^7^, Laura J. Damschroder^8^, Cara C. Lewis^5^

#### ^1^Hathaway-Sycamores Child and Family Services, Pasadena, CA, USA; ^2^University of Montana, Missoula, MT, USA; ^3^University of Washington, Seattle, WA, USA; ^4^Brown School, Washington University in St. Louis, St. Louis, MO, USA; ^5^Kaiser Permanente Washington Health Research Institute, Seattle, WA, USA; ^6^Hospital for Sick Children and University of Toronto, Toronto, ON, USA; ^7^The University of Newcastle, Newcastle, Australia; ^8^VA Ann Arbor Healthcare System, Center for Clinical Management Research (CCMR), Ann Arbor, MI, USA

##### **Correspondence:** Cameo F. Stanick (cstanick@hscfs.org)

**Background**

The incorporation and use of reliable, valid measures in implementation practice, outside of research, will remain limited if measures are not pragmatic. Previous research has identified the need for pragmatic measures, though the pragmatic properties identified were developed using only expert opinion and literature review. Our team carried out four studies with the goal of developing a stakeholder-driven pragmatic rating criteria for implementation measures. We previously published Studies 1 (populating the dimensions of the pragmatic construct via a literature review + stakeholder interviews) and 2 (clarifying the internal structure via concept mapping) that yielded 47 terms and phrases across four categories: Useful, Compatible, Acceptable, and Easy that were culled to 17 terms. This study presents the results of Studies 3 and 4: a Delphi to ascertain stakeholder-prioritized dimensions and a pilot study applying the dimensions as rating criteria.

**Materials and Methods**

Stakeholders (practitioners and implementation intermediaries; N=26) participated in an online modified Delphi and rated the relevance of 17 terms and phrases to the pragmatic construct. The investigative team pruned the list further and developed anchors for the pragmatic properties based on all available data sources (i.e., stakeholder interviews, literature review, concept mapping ratings, Delphi ratings). The final criteria were piloted with 60 existing implementation measures utilizing both empirical and grey literature.

**Results**

The Delphi methodology confirmed the importance of all identified pragmatic measure properties, but provided little guidance on relative importance. Following the Delphi, investigators removed/combined 6 more terms to obtain the final set of 11 criteria across four categories (Useful, Acceptable, Easy, Compatible) and assigned a 6-point rating system to each criterion. Application of the final rating criteria demonstrated sufficient variability across items; the grey literature did not add critical information.

**Conclusions**

This work produced the first stakeholder-driven rating criteria by which measures can be judged to be pragmatic. The Psychometric and Pragmatic Evidence Rating Scale (PAPERS) was developed by combining these pragmatic criteria with psychometric rating criteria from our previous work. Use of PAPERS can inform development of new implementation measures and to assess quality of existing measures.

## A68 Psychometric and pragmatic evaluation of measures of readiness for implementation

### Bryan J. Weiner^1^, Caitlin N. Dorsey^2^, Kayne D. Mettert^2^, Cara C. Lewis^2^

#### ^1^University of Washington, Seattle, WA, USA; ^2^Kaiser Permanente Washington Health Research Institute, Seattle, WA, USA

##### **Correspondence:** Bryan J. Weiner (bjweiner@uw.edu)

**Background**

Systematic measure reviews can facilitate advances in implementation research and practice by locating reliable, valid, pragmatic measures; identifying promising measures needing refinement and testing; and highlighting measurement gaps. Sponsored by Society for Implementation Research Collaboration (SIRC), with funding from the National Institute of Mental Health (NIMH), this review identifies and evaluates the psychometric and pragmatic properties of measures of readiness for implementation and its sub-constructs as delineated in the Consolidated Framework for Implementation Research: leadership engagement, available resources, and access to knowledge and information.

**Materials and Methods**

The systematic review methodology is described fully elsewhere [1]. Consistent with SIRC’s mission and NIMH’s priorities, the review focused on measures used in mental or behavioral healthcare. The review proceeded in three phases. Phase I, data collection, involved search string generation, title and abstract screening, full text review, construct assignment, and measure forward searches. Phase II, data extraction, involved coding relevant psychometric and pragmatic information. Phase III, data analysis, involved two trained specialists independently rating each measure using PAPERS (Psychometric And Pragmatic Evidence Rating Scale) [1]. Frequencies and central tendencies summarized information availability and PAPERS ratings.

**Results**

Searches identified nine measures of readiness for implementation, 24 measures of leadership engagement, 17 measures of available resources, and 6 measures of access to knowledge and information. Information about internal consistency was available for most measures. Information about other psychometric properties was often not available. Ratings for internal consistency were “adequate” or “good.” Ratings for other psychometric properties were less than “adequate.” Information was often available regarding cost, language readability, and brevity. Information was less often available regarding training burden and interpretation burden. Cost and language readability generally exhibited “good” or “excellent” ratings, interpretation burden generally exhibiting “minimal” ratings, and training burden and brevity exhibiting mixed ratings across measures.

**Conclusions**

Measures of readiness for implementation and its sub-constructs used in mental health and behavioral healthcare are unevenly distributed, exhibit unknown or low psychometric quality, and demonstrate mixed pragmatic properties. This review identified a few promising measures, but targeted efforts are needed to systematically develop and test measures that are useful for both research and practice.

**Reference**

1. Lewis CC, Proctor E, Brownson RC. Measurement issues in dissemination and implementation research. In: Brownson RC, Colditz GA, Proctor E, eds. Dissemination and implementation research in health: translating science to practice. 2nd ed. New York: Oxford University Press; 2018.

## A69 Measuring organizational culture and climate: a systematic review

### Byron J. Powell^1^, Kayne D. Mettert^2^, Caitlin N. Dorsey^2^, Mark G. Ehrhart^3^, Gregory A. Aarons^4^, Bryan J. Weiner^5^, Cara C. Lewis^2^

#### ^1^Brown School, Washington University in St. Louis, St. Louis, MO, USA; ^2^Kaiser Permanente Washington Health Research Institute, Seattle, WA, USA; ^3^University of Central Florida, Orlando, FL, USA; ^4^University of California San Diego, San Diego, CA, USA; ^5^University of Washington, Seattle, WA, USA

##### **Correspondence:** Byron J. Powell (bjpowell@wustl.edu)

**Background**

Organizational culture and climate have a long history in management research, and have been shown to impact implementation and clinical outcomes in mental health service settings. The purpose of this systematic review is to identify and evaluate the psychometric properties of measures of these constructs as they are defined by the Consolidated Framework for Implementation Research. Specifically, we aim to review measures of organizational culture, climate, and its subconstructs including tension for change, compatibility, relative priority, organizational incentives and reward, goals and feedback, and learning climate.

**Materials and Methods**

This systematic review was conducted as a part of a larger study, and the full protocol is published elsewhere [1]. The review proceeded in three phases. Phase I, data collection, involved search string generation, title and abstract screening, full text review, construct assignment, and measure forward searches. Phase II, data extraction, involved coding relevant psychometric and pragmatic information. Phase III, data analysis, involved two trained specialists independently rating each measures’ psychometric properties. Frequencies and central tendencies summarized information availability and psychometric ratings.

**Results**

Searches identified 65 measures or subscales assessing molar organizational culture or climate, 6 measures of implementation climate, 4 of which were subscales of other measures; and a number of other subscales for constructs related to the CFIR’s conceptualization of implementation climate, including: 2 assessing tension for change; 6 assessing compatibility; 2 assessing relative priority; 3 assessing organizational incentives and rewards; 3 assessing goals and feedback; and 2 assessing learning climate. Information about internal consistency and norms was available for most measures. Information about other psychometric properties was often not available. Ratings for internal consistency were most often “adequate” or “good.” Ratings for other psychometric properties were typically lower.

**Conclusions**

This review suggests some promising measures of organizational culture, climate, and related constructs; however, it also suggests a lack of conceptual clarity with respect to the differentiation between molar organizational culture and molar organizational climate. Implications for measure development and refinement will be discussed.

**Reference**

1. Lewis CC, Proctor E, Brownson RC. Measurement issues in dissemination and implementation research. In: Brownson RC, Colditz GA, Proctor E, eds. Dissemination and implementation research in health: translating science to practice. 2nd ed. New York: Oxford University Press; 2018.

## A70 A systematic review of outer setting measures in behavioral health

### Sheena McHugh^1^, Eric J. Bruns^2^, Jonathan Purtle^3^, Caitlin N. Dorsey^4^, Kayne D. Mettert^4^, Cara C. Lewis^4^

#### ^1^University College Cork, Cork, Ireland; ^2^University of Washington, Seattle, CA, USA; ^3^Drexel University, Philadelphia, PA, USA; ^4^Kaiser Permanente Washington Health Research Institute, Seattle, CA, USA

##### **Correspondence:** Sheena McHugh (s.mchugh@ucc.ie)

**Background**

One of the main challenges to measurement of implementation-relevant constructs is the mismatch between the level of measurement and the level of analysis [1-2]. This alignment is particularly challenging when measuring constructs relating to the outer setting, given the predominance of self-report in implementation science. The objective of this review is to assess the reliability, validity, and practicality of measures of outer setting and its CFIR-delineated constructs used in mental or behavioral healthcare.

**Materials and Methods**

This review of outer setting measures follows the same study protocol as the other systematic reviews initiated through this larger project [3]. Phase I, data collection, occurred in five steps: a) search string generation, b) title and abstract screening, c) full text review, d) construct assignment, and e) measure forward searches. Particular to this review, during search string generation an additional level was included for each of the CFIR constructs [4]; 1) Patient needs and resources, 2) Cosmopolitanism, 3) Peer Pressure, and 4) External Policy & Incentives. Phase II, data extraction, consisted of coding relevant information to the nine psychometric rating criteria using the Psychometric And Pragmatic Evidence Rating Scales (PAPERS) [3]. Phase III, data analysis, is underway. For each construct, frequencies will be used to summarize the availability of psychometric information for each PAPERS criterion for the measures for that construct. The median and the range of final ratings for each psychometric PAPERS criterion will be used to summarize the psychometric strength of the measures for that construct.

**Results**

Electronic searches yielded four measures of outer setting; four measures of patient needs and resources; eight measures of cosmopolitanism; one measure of peer pressure and six measures of external policy and incentives. Five of these measures were unsuitable for rating. Analysis of the psychometric properties of the remaining measures if ongoing.

**Conclusions**

The outer setting is the CFIR domain with the fewest measures identified. This paper advances implementation science and practice through consideration of how the outer setting should be conceptualized and measured. Outer setting constructs may be more appropriately assessed at a system rather than individual level, and by using direct measurement instead of latent variables.

**References**

1. Emmons KM, Weiner B, Fernandez ME, Tu SP. Systems antecedents for dissemination and implementation: a review and analysis of measures. Health Educ Behav. 2012;39(1):87-105.

2. Lewis CC, Proctor E, Brownson RC. Measurement issues in dissemination and implementation research. In: Brownson RC, Colditz GA, Proctor E, eds. Dissemination and implementation research in health: Translating science to practice. 2nd ed. New York: Oxford University Press; 2018.

3. Lewis CC, Mettert KD, Dorsey CN, Martinez RG, Weiner BJ, Nolen E, Stanick C, Halko H, Powell BJ. An updated protocol for a systematic review of implementation-related measures. Syst Rev. 2018;7(1):66.

4. Damschroder LJ, Aron DC, Keith RE, Kirsh SR, Alexander JA, Lowery JC. Fostering implementation of health services research findings into practice: a consolidated framework for advancing implementation science. Implement Sci. 2009;4(1):50.

## A71 Applying the Theory of Planned Behavior and the Consolidated Framework for Implementation Research to understand therapists’ perceived barriers and facilitators to using trauma narratives

### Hannah Frank^1^, Briana Last^2^, Reem AlRabiah^2^, Jessica Fishman^2^, Brittany Rudd^2^, Hilary Kratz^3^, Colleen Harker^2^, Sara Fernandez-Marcote^2^, Kamilah Jackson^2^, Rinad Beidas^2^

#### ^1^Temple University, Philadelphia, PA, USA; ^2^University of Pennsylvania, Philadelphia, PA, USA; ^3^La Salle University, Philadelphia, PA, USA

##### **Correspondence:** Hannah Frank (08hannahf@gmail.com)

**Background**

Trauma narratives (TN) are a critical component of trauma-focused cognitive-behavioral therapy (TF-CBT; [1]) yet therapists report using TNs infrequently [2]. One causal theory that may explain the infrequent use of TNs is the Theory of Planned Behavior (TPB; [3]). The TPB states that intentions, which are informed by attitudes, subjective norms, and self-efficacy, are the strongest predictor of actual behavior. The TPB also acknowledges that certain contextual factors need to be present in order for intentions to translate into actual behavior. Implementation science frameworks, such as the Consolidated Framework for Implementation Research (CFIR; [4]), provide insight into the types of contextual factors that may affect clinician behavior (e.g., organizational variables). This study aimed to identify barriers to TN use by integrating a causal theory of behavior change (TPB) and a widely used contextual framework (CFIR).

**Materials and Methods**

Sixty-five mental-health therapists working in community settings and trained through a city-wide TF-CBT initiative participated by completing a survey about their use of and beliefs about TNs. Content analysis was conducted to identify common beliefs about TNs. A subset of participants (n=17) completed in-depth qualitative interviews focused on perceptions of TNs. Qualitative interviews were analyzed using an integrated approach informed by the CFIR.

**Results**

While most participants reported high intentions to use TNs, nearly half reported that they did not use TNs in the last six months. The initial survey identified beliefs related to TPB constructs, including clinician attitudes (e.g., TN may worsen symptoms), norms (e.g., caregivers may disapprove), and self-efficacy (e.g., insufficient training). CFIR-informed qualitative interviews yielded themes related to client and family characteristics (e.g., client reluctance, instability in caregivers) and organizational factors (e.g., brief sessions).

**Conclusions**

Results indicate a discrepancy between intentions and TN use. Responses to the survey and qualitative interviews indicate that there are contextual factors that may moderate the relationship between intentions and behaviors. For example, organizational constraints, such as time limitations, may prevent TN use even among therapists with high intentions. Results from this study highlight the importance of integrating theories that address multiple determinants of clinician behavior to identify potential targets for implementation strategies.

**References**

1. Cohen JA, Mannarino AP. Trauma-Focused Cognitive Behavioural Therapy for children and parents. Child Adol Ment Health. 2008;13(4):158-162.

2. Allen B, Johnson JC. Utilization and implementation of Trauma-Focused Cognitive–Behavioral Therapy for the treatment of maltreated children. Child Maltreat. 2011;17(1):80-85.

3. Ajzen I. The theory of planned behavior. Organ Behav Hum Dec Process. 1991;50(2):179-211.

4. Damschroder LJ, Aron DC, Keith RE, Kirsh SR, Alexander JA, Lowery JC. Fostering implementation of health services research findings into practice: A consolidated framework for advancing implementation science. Implement Sci. 2009; 4: 50. doi:10.1186/1748-5908-4-50.

## A72 The relationship between therapist-driven adaptations to evidence-based practices (EBP) and the extensiveness of EBP strategy delivery in community implementation

### Stephanie H. Yu^1^, Lauren Brookman-Frazee^2^, Joanna J. Kim^1^, Miya L. Barnett^3^, Anna S. Lau^1^

#### ^1^University of California, Los Angeles, Los Angeles, CA, USA; ^2^University of California, San Diego, San Diego, CA, USA; ^3^University of California, Santa Barbara, Santa Barbara, CA, USA

##### **Correspondence:** Stephanie H. Yu (stephaniehtyu@ucla.edu)

**Background**

Community therapists adapt evidence-based practices (EBPs) to enhance fit for their complex settings and clients when implemented [1]. Yet, few studies have examined the potential implications therapist-driven adaptations have for the quality of EBP delivery [2]. We examined the extent to which different types of therapist-reported adaptations were associated with the extensiveness of EBP strategy delivery in a system-driven implementation of multiple EBPs.

**Materials and Methods**

Data were drawn from an observational study investigating the sustainment of six EBPs for youth in the Los Angeles County Department of Mental Health [3]. Community therapists (n=103) provided descriptions of any adaptations they made in 680 sessions with 273 clients. Trained coders rated the extensiveness of EBP strategy delivery via the EBP Concordant Care Assessment (ECCA) from session recordings [3]. We examined how different types of therapist adaptations were associated with ECCA extensiveness ratings. Adaptations were categorized as: 1) modifying presentation, 2) integrating (integrating components, combining practices, providing psychoeducation), 3) extending (repeating components, lengthening), 4) reducing (removing, reordering, or shortening components), and 5) other (adaptations that could not be coded within our framework due to lack of fit) [4]. Furthermore, we examined these relationships through an augmenting (modifying presentation, integrating, extending) vs. reducing (removing, reorder, shortening) vs. “other” framework [4].

**Results**

Preliminary analysis revealed that the overall number of therapist adaptations did not significantly predict extensiveness ratings (β = .067, p = .059). However, specific adaptations were related to extensiveness. Modifying presentation was associated with higher extensiveness (β = .098, p = .046), while “other” adaptations were associated with lower extensiveness (β = -.119, p = .034). When compared through an augmenting vs. reducing vs. “other” framework, “other” adaptations predicted lower extensiveness (β = -.127, p = .023).

**Conclusions**

Quality of EBP delivery may be robust to some types of therapist-driven adaptations. In fact, adaptations that tailor the presentation of interventions were associated with more extensive EBP delivery. In contrast, “Other” adaptations were more likely to be associated with lower EBP extensiveness and thus may represent “drift.” Additional analyses to further characterize the content of the “other” adaptations will be included in the proposed presentation.

**References**

1. Stirman SW, Gamarra JM, Bartlett BA, Calloway A, Gutner CA. Empirical examinations of modifications and adaptations to evidence-based psychotherapies: methodologies, impact, and future directions. Clin Psychol. 2017;24:396-420.

2. Escoffery C, Lebow-Skelley E, Haardoerfer R, Boing E, Udelson H, Wood R, Hartman M, Fernandez ME, Mullen PD. A systematic review of adaptations of evidence-based public health interventions globally. Implement Sci. 2018;13:125.

3. Lau AS. Brookman-Frazee L. The 4KEEPS study: identifying predictors of sustainment of multiple practices fiscally mandated in children’s mental health services. Implement Sci. 2016;11:31.

4. Lau A, Barnett M, Stadnick N, Saifan D, Regan J, Stirman SW, Roesch S. Brookman-Frazee L. Therapist report of adaptations to delivery of evidence-based practices within a system-driven reform of publicly funded children’s mental health services. J Consult Clin Psychol. 2017;85(7):664-675.

## A73 One size does not fit all: clinician intentions to implement cognitive-behavioral therapy vary by specific component

### Emily Becker-Haimes, Jessica Fishman, Torrey Creed, Courtney Benjamin Wolk, Nicholas Affrunti, Danielle Centeno, David Mandell

#### University of Pennsylvania, Philadelphia, PA, USA

##### **Correspondence:** Emily Becker-Haimes (embecker@upenn.edu)

**Background**

Developing implementation strategies to increase clinicians’ use of evidence-based practices (EBPs) is important for improving the quality of mental health care. Our prior work with school-based services for youth with autism demonstrated that studying practitioners’ intentions to use specific EBP components rather than their intentions to use broader intervention protocols, may point to levers by which to tailor implementation strategies as a function of the specific EBPs themselves (e.g., their salience, complexity) [1]. We extend this work by examining variability in community clinicians’ intentions to use different components of cognitive-behavioral therapy (CBT). CBT has long been a target of implementation efforts [2-3] yet remains underutilized in community settings [4-5]. CBT comprises multiple, distinct components that vary in their complexity, ranging from relatively simple (e.g., homework review) to more complex interventions (e.g., cognitive restructuring). Examining how intentions vary across specific CBT components may yield insights into how to tailor implementation strategies to increase CBT use in community settings.

**Materials and Methods**

Community mental health providers (N = 149, mean age = 40.8 years, 57.7% female) who had received intensive training and consultation in CBT were surveyed about their intentions to use six key elements of CBT interventions (exposure therapy, cognitive restructuring, behavioral activation, planning homework, reviewing homework, and agenda-setting) for seven different clinical presentations. Clinicians also reported on their intentions to use “EBP” broadly. Demographic and clinical background characteristics also were collected.

**Results**

Analyses are ongoing. Nearly all clinicians reported high intentions to use “EBPs” broadly across all seven clinical presentations. However, intentions varied widely when clinicians reported on specific CBT components. In general, higher intentions were observed for CBT components that were simpler and part of the session structure (e.g., reviewing homework vs. exposure therapy). Additional analyses will examine clinician characteristics as predictors of intentions.

**Conclusions**

Findings highlight variability in clinician intentions across specific elements of CBT and suggest that CBT implementation strategies may need to be tailored as a function of specific intervention components. Results also suggest that implementation efforts may benefit from dismantling complicated interventions, highly specifying the practitioner behavior of interest, and tailoring implementation strategies to each one.

**References**

1. Fishman J, Beidas RS, Reisinger E, Mandell DS. The utility of measuring intentions to use best practices: a longitudinal study among teachers supporting students with autism. J School Health. 2018;88(5):388-95.

2. Novins DK, Green AE, Legha RK, Aarons GA. Dissemination and implementation of evidence-based practices for child and adolescent mental health: a systematic review. J Am Acad Child Adol Psych. 2013;52(10):1009-25.

3. McHugh RK, Barlow DH. The dissemination and implementation of evidence-based psychological treatments: a review of current efforts. Am Psychol. 2010;65(2):73.

4. Smith MM, McLeod BD, Southam-Gerow MA, Jensen-Doss A, Kendall PC, Weisz JR. Does the delivery of CBT for youth anxiety differ across research and practice settings?. Behav Ther. 2017;48(4):501-16.

5. Finley EP, Noël PH, Lee S, et al. Psychotherapy practices for veterans with PTSD among community-based providers in Texas. Psychol Serv. 2018;15(4):442.

## A74 A comparison of consultant effects, activities, and perceptions on therapist fidelity and patient treatment outcomes

### Heidi La Bash^1^, Norman Shields^2^, Tasoula Masina^3^, Kera Swanson^1^, Jiyoung Song^1^, Clara Johnson^1^, Matthew Beristianos^1^, Erin Finley^4,5^, Vanessa Ramirez^5^, Jeanine Lane^3^, Michael Suvak^6^, Candice Monson^3^, Shannon Wiltsey Stirman^1^

#### ^1^National Center for PTSD, VA Palo Alto Health Care System, Palo Alto, CA, USA; ^2^Veterans Affairs Canada, West Montreal, Quebec, Canada; ^3^Ryerson University, Toronto, Ontario, Canada; ^4^South Texas Veterans Health Care System, San Antonio, TX, USA; ^5^University of Texas Health Science Center at San Antonio, San Antonio, TX, USA; ^6^Suffolk University, Boston, MA, USA

##### **Correspondence:** Heidi La Bash (hlabash@gmail.com)

**Background**

Research has demonstrated that workshops alone do not lead to sufficient skill in delivering evidence-based psychotherapies (EBP), and that strategies such as follow-up consultation are needed. Yet, there is little research to inform how to best provide consultation to ensure sustained, high-quality delivery of EBPs.

**Materials and Methods**

The parent randomized controlled implementation trial assessed the impact of three post-cognitive processing therapy (CPT) training workshop conditions (No consultation, Standard consultation without session audio review, Consultation with audio review) on the patient (N=188) PTSD treatment outcomes of participating therapists (n=134) in over 30 routine care settings in Canada. The current mixed-methods study examines associations between consultation activities and therapist fidelity and patient treatment outcomes, as well as the accuracy of consultant perceptions of the skill and engagement of therapist consultees. Consultation occurred weekly for six months. After each recorded call, consultants (n=5) completed a post-call checklist of strategies and rating of perceived levels of enthusiasm, skill, and participation for each consultee. A subset of therapists (n=30) were interviewed at the end of the consultation phase.

**Results**

While there was variability, three primary categories of activities emerged: case conceptualization and intervention planning, feedback on fidelity, and distractions/technical difficulties. Similarities and differences in consultant and therapist perceptions of consultation activities will be presented. Additionally, analyses revealed evidence of a consultant effect on therapist treatment adherence (B=0.439, SE=0.171, p=.012), but not competence (-B=0.341, SE=0.247, p=.168). This will be explored in relation to consultation condition and strategies, including whether different consultants engaged in specific activities more frequently. Finally, we found that while consultants perceived overall improvements over the course of consultation (b = 0.03, t = 6.12, p < .01), their ratings of therapist skill did not predict clinician adherence (b = 0.03, t = 0.46, p = .65), competence (b = 0.03, t = 0.31, p =.76), or patient PTSD symptom change (b = -.12, t = -0.31, p = .76). It is possible that cognitive biases (e.g., halo effect) may reduce the accuracy of consultant perceptions.

**Conclusions**

Practical implications of this and the other study findings will be presented in a broader discussion of barriers/facilitators of EBP sustainment.

**Trial Registration:** Clinicaltrials.gov NCT02449421

## A75 From blank page to local optimization: participatory systems modeling to improve local evidence based practice implementation

### David Lounsbury^1^, Debra Kibbe^2^, James Rollins^3^, Lindsey Zimmerman^4^

#### ^1^Albert Einstein College of Medicine, Yeshiva University, Bronx, NY, USA; ^2^Georgia Health Policy Center, Georgia State University, Atlanta, GA, USA; ^3^Takouba Security LLC, Seattle, WA, USA; ^4^National Center for PTSD, VA Palo Alto Health Care System, Palo Alto, CA, USA

##### **Correspondence:** David Lounsbury (David.lounsbury@einstein.yu.edu)

**Background**

This panel is for implementation practitioners interested in systems science and participatory modeling approaches to evidence-based practice (EBP) implementation. Panelists will review participatory development of “Modeling to Learn (MTL),” a program for improving EBP implementation in the Veterans Health Administration (VA) [1-2]. Study of MTL is underway to determine its effectiveness increasing delivery of evidence-based addiction and mental health care (R01DA046651). Preliminary statistical process control analyses indicate pilot clinics demonstrated a three standard deviation increase in EBP reach and maintained improvement for 12 and 8 months, respectively (R21DA042198).

**Materials and Methods**

Using the MTL example, this panel answers commonly asked questions about systems science approaches to implementation through demonstration. Four years ago, panelists began learning with frontline multidisciplinary teams about determinants of local reach of evidence-based psychotherapies and pharmacotherapies (EBPs). Support for modeling to identify locally tailored implementation plans grew among VA stakeholders, and each panelist joined the project to contribute systems modeling expertise: Dr. Lounsbury as an NIH-funded researcher, Ms. Kibbe as a public health facilitator, and Col. Rollins (Ret.) as an online simulation user interface developer.

**Results**

Panelists will present participatory modeling activities, linking them to free, online open science resources. Dr. Lounsbury will describe initial qualitative group model building exercises, developed in the field of system dynamics [4-5]. These activities illustrate how to determine the right modeling problem via participatory engagement. Ms. Kibbe will describe the participatory principles and pragmatic constraints used to refine MTL online facilitation resources for teams to develop systems thinking skills at national scale [3]; this includes the MTL session guides and fidelity checklist. Col. Rollins will describe design iterations to produce an interface for frontline teams to simulate improvement scenarios using team data. Attendees will be provided access to a demonstration website developed to help users understand how simulation makes local EBP barriers and facilitators more transparent and locally manageable.

**Conclusions**

As discussant, Dr. Zimmerman will synthesize the activities described by panelists in relation to the learning objectives, to help implementation practitioners and intermediaries get started with participatory modeling.

**References**

1. Zimmerman L, Lounsbury D, Rosen C, Kimerling R, Trafton J. Lindley S. Participatory system dynamics modeling: Increasing stakeholder engagement and precision to improvement implementation. Adm Policy Ment Health. 2016;43(6):834-849. doi:10.1007/s10488-016-0754-1

2. Zimmerman L, Lounsbury D, Rosen C, Kimerling R, Holbrook A, Hong S, Branscomb J, Kibbe D, Park S, Kramer R, Barlow DC, Mushiana S, Azevedo K, Yang J, Trafton J, Lindley S, Rust T. 2018, December. Participatory system dynamics for high quality VA addiction and mental health care. Oral presentation in L. Zimmerman (Chair), Participatory modeling approaches to implementation science, 11th Annual Conference on the Science of Dissemination and Implementation in Health, Washington, D.C.

3. Sterman JD. Learning from evidence in a complex world. Am J Public Health. 2006;96:505–514.

4. Hovmand PS. Community Based System Dynamics. New York, NY: Springer New York; 2014.

5. Hovmand PS, Rouwette EAJA, Andersen DF, Richardson GP, Kraus A. Scriptapedia 4.0.6. 2013.

## A76 Building implementation capacity through development of a coaching network

### Kristen Miner, Emily Bilek, Jennifer Vichich, Shawna Smith, Elizabeth Koschmann

#### University of Michigan, Ann Arbor, MI, USA

##### **Correspondence:** Kristen Miner (krminer@med.umich.edu)

**Background**

Mood and anxiety disorders affect approximately 30% of youth [1]. Cognitive Behavioral Therapy (CBT) is an evidence-based treatment for these disorders, but only a fraction of adolescents in need have access to high quality treatment. Access could be substantially increased if school professionals (SPs) were trained to deliver CBT. However, typical professional development and clinical training opportunities are often unsuccessful because they lack post-training support necessary for producing sustained behavioral change. TRAILS is an implementation and training model designed to increase utilization of CBT among SPs. In addition to clinical training and resources, TRAILS is unique in that it also provides in-person coaching to support SPs as they co-lead student CBT skills groups.

**Materials and Methods**

To build capacity for large-scale and sustainable implementation of CBT, TRAILS established a statewide network of expert “coaches”, recruiting primarily from Community Mental Health (CMH) agencies across Michigan. Participation was incentivized via provision of training and CEUs at no cost to potential coaches and up to 10 (non-coaching) clinicians per partner agency. Coaches participated in a daylong CBT training, followed by 12 weeks of individual case consultation from an expert clinician. Coaches were assessed pre- and post- consultation and successful completers were recommended for subsequent training in the TRAILS coaching protocol.

**Results**

TRAILS trained five cohorts of coaches over the course of two years. Of 125 trainees completing consultation, 108 were recommended for coaching based on clinical skill, and 84 were ultimately trained in the TRAILS coaching protocol. TRAILS Coach training led to improvement in CBT expertise (p<0.001) and frequency of use (p<0.001), with 87% of coaches reporting feeling “extremely satisfied” with their training. TRAILS Coaches now span 63 of Michigan’s 83 counties and have been paired with 47 schools across Michigan as part of a NIMH clinical trial.

**Conclusions**

Large-scale implementation efforts often require development of new infrastructure to ensure capacity to support implementation strategy deployment and long-term sustainability. By creating a statewide network of coaches, TRAILS has built the infrastructure necessary to support SPs in learning and delivering CBT to students in need and provides one model for feasible creation of statewide implementation infrastructure.

**Reference**

1. Merikangas KR, He JP, Burstein M, Swanson SA, Avenevoli S, Cui L, Benjet C, Georgiades K, Swendsen J. Lifetime prevalence of mental disorders in U.S. adolescents: results from the National Comorbidity Survey Replication—Adolescent Supplement (NCS-A). J Am Acad Child Adolesc Psychiatry. 2010;49(10):980-9. doi:10.1016/j.jaac.2010. 05.017.

## A77 The parent engagement in evidence-based services questionnaire: advancing our understanding of parental intentions for engaging in evidence-based practice

### Spencer Choy, Jaime Pua Chang, Brad Nakamura

#### University of Hawaii at Manoa, Honolulu, HI, USA

##### **Correspondence:** Spencer Choy (choyskj@hawaii.edu)

**Background**

The Parent Engagement in Evidence-Based Services (PEEBS) [1] is a newly developed questionnaire that aims to assess parent consumer perspectives about evidence-based practices through the theory of planned behavior, a widely-applied model that suggests parental attitudes, perceived behavioral control (PBC), and subjective norms (SN) shape behavioral intentions. Towards the larger goal of better understanding factors associated with parental behavioral intentions for utilizing such services, we present data with a community parent sample of the PEEBS’ factor structure, internal consistencies, and relations with related instruments.

**Materials and Methods**

351 parents (75.8% female; M = 40.4 years old, SD = 7.6; 62.3% Asian) recruited from 15 community outreach efforts in Hawaii completed the PEEBS, the Family Empowerment Scale (FES) [2], and the Parental Attitudes Toward Psychological Services Inventory (PATPSI) [3]. Exploratory factor analysis was conducted with principal axis factoring and oblique rotation, Cronbach alpha coefficients were used to calculate internal consistencies, and Pearson correlations were computed to investigate convergent validity.

**Results**

Exploratory factor analysis suggested a five-factor structure (50 items, alpha = .86). Items grouped along the dimensions of SN (11 items, alpha = .80), treatment barriers (10 items, = .71), treatment knowledge (6 items, alpha = .72), evidence-informed action (11 items, alpha = .83), and PBC (12 items, alpha = .83), that accounted for 38.07% of the total variance. Regarding Pearson bivariate correlations, PEEBS’ PBC was significantly and positively associated with evidence-informed action (r = .50, p < .01) and SN (r = .35, p < .01). As expected, the FES family scale was significantly and positively associated with the PEEBS’ knowledge factor (r = .42, p < .01), PBC (r = .32, p < .01), and evidence-informed action (r = .17, p < .01). PATPSI’s help-seeking intentions were significantly and positively correlated with the PEEBS’ PBC (r = .37, p < .01), evidence-informed action (r = .33, p < .01), knowledge (r = .29, p < .01), and subjective norms (r = .22, p < .01).

**Conclusions**

Based on promising psychometric results, the PEEBS appears to be a potentially useful instrument to understand parent consumers.

**References**

1. Chang JP, Orimoto TE, Selbo-Bruns A, Choy SKJ, & Nakamura BJ. Application of the Theory of Planned Behavior to caregiver consumer engagement in evidence-based services. (under review).

2. Koren PE, DeChillo N, Friesen BJ. Measuring empowerment in families whose children have emotional disabilities: a brief questionnaire. Rehab Psychol. 1992;37(4):305-321.

3. Erlanger EA. The parental attitudes toward psychological services inventory: Adaptation and development of an attitude scale. Comm Ment Health J. 2012;48(4):436-449.

## A78 What works best in practice? the effectiveness of ‘real-world’ facilitation strategies in overcoming evidence-based barriers to implementation

### Lydia Moussa, Katarzyna Musial, Simon Kocbek, Victoria Garcia Cardenas

#### University of Technology Sydney, Sydney, NSW, Australia

##### **Correspondence:** Lydia Moussa (lydia@thechangehub.com.au)

**Background**

Research has shed light into factors affecting the implementation of innovations in practice as well as facilitation strategies that can be utilised during the implementation of these innovations. Change facilitators, however, often use a ‘trial and error’ approach when determining the best strategy to overcome the particular barrier identified. This leads to loss of time, resources and a team’s reduced motivation to successfully implement the innovation. To overcome this challenge, we developed a machine-learning tool that connects evidence-based barriers with the most effective, pragmatic change facilitation strategies, as trialed by change facilitators in the real world.

**Materials and Methods**

A 2-year change program was facilitated across 19 pharmacies around Australia by six facilitators. Facilitators identified barriers to implementation and recorded the strategies they used to overcome these barriers. The barriers were coded according to implementation factors from the Consolidated Framework of Implementation Research [1] and the Theoretical Domains Framework. Strategies were coded according Dogherty et al. facilitation strategy taxonomy [2]. To determine the most effective facilitation strategies, a decision forest [3] algorithm was developed.

**Results**

We collected 1131 data points from six facilitators, which were categorised into 36 barriers to implementation and 111 unique change facilitation strategies. The Decision Forest algorithm highlighted the effectiveness of the facilitation strategies according to the strategy ‘resolve rate’ (RR). The most frequent barrier in the pharmacy practice setting was the ‘inability to plan for change’ (n=184). The strategies used to overcome this barrier were to: a) ‘Manage the different the requirements of each discipline/ role and create ownership’ (RR= 84.23%), b) ‘Provide training’ (RR= 83.30%) c) ‘Adapt area of focus to change’ (RR= 81.17) d) ‘Assist the group to develop ideas and solve problems’ (RR= 80.64%).

**Conclusions**

By understanding that the most prevalent barrier to implementation was an ‘inability to plan for change’, this provides pharmacy practice policy makers, tertiary educators, researchers and change facilitators an idea of where to target strategies to not only overcome, but prevent this barrier from occurring. This tool can be reproduced to understand implementation barriers specific to other industries and the most effective strategies to overcome these.

**References**

1. Damschroder L, Hagedorn H. A guiding framework and approach for implementation research in substance use disorders treatment. Psychol Addict Behav. 2011;25(2):194-205. doi:10.1037/a0022284

2. Dogherty E, Harrison M, Graham I. Facilitation as a role and process in achieving evidence-based practice in nursing: a focused review of concept and meaning. Worldviews Evid Based Nurs. 2010;7(2):76-89. doi:10.1111/j.1741-6787.2010.00186.x

3. Khalilia M, Chakraborty S, Popescu M. Predicting disease risks from highly imbalanced data using random forest. BMC Med Inform Decis Mak. 2011;11(1). doi:10.1186/1472-6947-11-51

## A79 Implementation strategies of a co-designed physical activity program for older adults

### Erica Lau, Joanie Sims-Gould, Samantha Gray, Heather McKay

#### University of British Columbia, Vancouver, British Columbia, Canada

##### **Correspondence:** Erica Lau (erica.lau@ubc.ca)

**Background**

Despite the known health benefits of physical activity (PA), 87% of Canadian older adults do not meet recommended PA guidelines. Community-based PA interventions show promise, but few were scaled-up. With partners, the Active Aging Research Team (AART) co-created Choose to Move (CTM), a 6-month, choice-based health promotion intervention that aims to improve older adults’ social connectedness and mobility through PA. During small scale-up, two community delivery partner organizations delivered 56 CTM programs in 26 urban communities across BC. We previously demonstrated that CTM effectively improved PA, mobility and social connectedness in 458 older adults [1]. The objective of this study was to evaluate effectiveness of CTM implementation strategies to guide broad scale-up (175 programs) of CTM.

**Materials and Methods**

Grounded in implementation frameworks (e.g., QIF [2], Powell et. al. [3]), CTM adopted eight key implementation strategies: 1. develop strong community partnerships; 2. develop an implementation blueprint; 3. ongoing implementation monitoring; 4. promote adaptability; 5. provision of program materials and tools; 6. centralized training and technical assistance; 7. convene advisory committees, and 8. a staged implementation scale-up approach. To assess effectiveness of implementation strategies, we measured four implementation outcomes (reach, participant responsiveness, quality, and delivery partners’ perceptions of the strategy). We administered surveys and conducted interviews with CTM participants (n=42) and delivery partners across four levels of influence (19 decision makers, 6 recreation managers, 27 recreation coordinators, 23 activity coaches) at mid- and post-intervention.

**Results**

CTM implementation strategies were effective across all levels of evaluation. Of 458 participants, 82% attended ≥75% of group meetings; 95% completed ≥70% of check-ins (reach); 75.3% were satisfied with CTM (participant responsiveness). From interviews, CTM was implemented with quality. Participants’ had very positive perceptions of their ACs. Delivery partners noted that intervention materials were appropriate and CTM was flexible and easy to implement. Central support (e.g., training, integration) provided by AART was instrumental to effectively implementing CTM.

**Conclusions**

Implementation strategies must be adapted to provide “best fit” for the delivery context. Our findings support a suite of implementation strategies that promoted scale up a health promotion intervention that other interventions can adopt, modify and evaluate in future.

**References**

1. McKay H, Nettlefold L, Bauman A, Hoy C, Gray SM, Lau E, Sims-Gould J. Implementation of a co-designed physical activity program for older adults: Positive impact when delivered at scale. BMC Public Health. 2018;18(1):1289.

2. Meyers DC, Durlak JA, Wandersman A. The quality implementation framework: A synthesis of critical steps in the implementation process. Am J Comm Psychol. 2012;50(3-4):462-480.

3. Powell BJ, Waltz TJ, Chinman MJ, Damschroder LJ, Smith JL, Matthieu MM, Proctor EK, Kirchner JE. A refined compilation of implementation strategies: Results from the Expert Recommendations for Implementing Change (ERIC) project. Implement Sci. 2015;10:21.

## A80 Innovative funding to achieve reach: pay for success

### Suzanne E. U. Kerns^1^, Mollie Bradlee^2^

#### ^1^University of Denver, Denver, CO, USA; ^2^Colorado Office of Children, Youth, and Families, Denver, CO, USA

##### **Correspondence:** Suzanne E. U. Kerns (suzanne.kerns@du.edu)

**Background**

Large scale dissemination and implementation of evidence-based interventions is limited by funding. Further, agencies are often required to shoulder the burden of costs while monetary benefits are realized in other service sectors. Thus, novel funding strategies are warranted. Pay for Success (PfS), also known as social impact bonds, is one such strategy [1]. Under PfS, governments may leverage private or philanthropic upfront capital to fund interventions, and subsequently repay those funders based on outcomes. However, there are notable considerations as it relates to implementation of this strategy, as described by Lowe et al. (2019) [2]. According to their model, it is easier to achieve agreement at the macro-policy levels, but challenges arise in meso- and micro- levels.

In 2015, the Colorado state legislature approved a law that enabled the state to enter into PfS arrangements. Shortly thereafter, a “Call for Innovation” was released from the Governor’s Office to identify potential PfS projects to improve outcomes for at-risk teens. The present project was one of three selected, and focuses on bringing an evidence-based intervention, Multisystemic Therapy [3], to under-served regions of the state. This presentation includes the perspectives of a state partner and the University-based entity implementing the project.

**Materials and Methods**

The Lowe et al. (2019) [2] framework is used to contextualize the PfS structuring of the initiative, which required alignment of funders, state partners, evaluators, and the project-based implementation team. This is the first PfS initiative that partnered with a non-profit, private university as the primary fiscal agent.

**Results**

This presentation describes the successful launch of the Colorado-based PfS initiative in December, 2018. Three cohorts, each containing two MST teams, are being rolled out over the next two years. The evaluation, on which the success payments are connected, uses a propensity score matching procedure because of the relatively lower population density in the service areas. Over the course of the project, we anticipate over 600 families to be served.

**Conclusions**

Although complicated to enact, PfS may become an important strategy to support large scale dissemination. Strategies to simplify the contracting process and support the meso- and micro-policy tensions is necessary for broad-scale uptake.

**References**

1. Warner ME. Private finance for public goods: Social impact bonds. J Econ Policy Reform. 2013;16(4):303-319.

2. Lowe T. Kimmitt J, Wilson R, Martin M, Gibbon J. The institutional work of creating and implementing Social impact bonds. Policy Politics. 2019;47(2);353-370.

3. Henggeler SW, Pickrel SG, Brondino MJ. Multisystemic treatment of substance-abusing and-dependent delinquents: Outcomes, treatment fidelity, and transportability. Ment Health Ser Res. 1999;1(3):171-184.

## A81 Using common elements and co-creation to enhance implementability of evidence-informed interventions: example from an academic intervention in Norwegian child welfare

### Thomas Engell^1^, Benedicte Kirkøen^1^, Karianne Thune Hammerstrøm^1^, Hege Kornør^2^, Kristine Horseng Ludvigsen^1^, Kristine Amlund Hagen^3^

#### ^1^Regional Centre for Child and Adolescent Mental Health, Trondheim, Norway; ^2^Norwegian Institute of Public Health, Oslo, Norway; ^3^Norwegian Center for Child Behavioral Development, Oslo, Norway

##### **Correspondence:** Thomas Engell (te@r-bup.no)

**Background**

Implementation and sustainment of effective interventions remains a struggle across public services, especially in settings with limited readiness for implementation. Increasing the implementability of interventions can facilitate more successful implementation. There is growing interest in identifying and studying discrete elements that are common across interventions for the purpose of hypothesis generation, intervention optimization and re-design, and implementation in practice. We combine common elements methodology with collaborative design approaches to develop and test implementable evidence-informed interventions tailored to individual and contextual needs. This session will present preliminary results from a common elements-based academic intervention for children in child welfare.

**Materials and Methods**

We used common elements methodology to identify common practice-, process-, and implementation elements in systematically reviewed academic interventions for children at risk. We compared frequencies of the most common elements and combinations in effective interventions with frequencies in ineffective interventions. Using facilitated co-creation with stakeholders, practitioners and user-representatives, we aimed to unify perspectives of researchers, implementers, managers, practitioners, and end-users to develop an implementable academic intervention based on the identified common elements. We developed dynamic fidelity monitoring encouraging flexible use of elements and adaptations, and we co-created blueprints for implementing, evaluating, and sustaining the intervention. We are now conducting a mixed-methods hybrid pragmatic trial where we assess the child welfare contexts’ readiness for implementation, intervention implementability (feasibility, appropriateness, acceptability, usability), implementation quality, and intervention effectiveness.

**Results**

We included 30 effective and 6 ineffective academic interventions for children at risk in a systematic review. We identified 62 practice elements, 49 process elements, and 34 implementation elements used in the interventions. Frequency count values (FVs; inclusion in effective vs ineffective interventions) were calculated for each element and commonly used combinations of elements. Elements and combinations with the highest FVs were used in facilitated co-creation to develop the intervention Enhanced Academic Support (EAS). Preliminary mixed methods results on the implementability of EAS and associations with readiness will be presented at the conference.

**Conclusions**

Combining common elements methodology with collaborative approaches for intervention design can be a viable approach for developing implementable interventions tailored to individual and contextual needs

## A82 Examining the relationship between client engagement challenges and community therapists’ delivery of evidence-based strategies to youth and their caregivers

### Blanche Wright^1^, Anna Lau^1^, Joanna Kim^1^, Resham Gellatly^1^, Mary Kuckertz^2^, Lauren Brookman-Frazee^2^

#### ^1^University of California, Los Angeles, Los Angeles, CA, USA; ^2^University of California, San Diego, La Jolla, CA, USA

##### **Correspondence:** Blanche Wright (bmwright2020@ucla.edu)

**Background**

In the delivery of youth-focused evidence-based practices (EBPs), high levels of client engagement have been positively associated with improved clinical outcomes [1]. In the literature, engagement has been primarily indexed by attendance [2], and more refined qualitative measures of engagement are needed. Thus, in the current study, we used an observational coding system to examine specific in-session client engagement challenges within a county-wide implementation of multiple EBPs in youth mental health services.

**Materials and Methods**

The aims of the study were twofold: (1) characterize the frequency of client engagement challenges and (2) examine whether these challenges are associated with the extent to which community therapists deliver EBP strategies. Extensiveness of EBP strategies was measured via therapist-report and observer-ratings. The sample included 103 therapists who provided recordings of 680 sessions in which they delivered an EBP to 273 youths (Mean age=9.75 years). Using observational coding, in-session youth and parent engagement challenges (i.e., client expressed concerns, refusal to participate in activities) were measured with good reliability (Mean ICC=.64).

**Results**

Across both youth only and youth + parent sessions, 66.29% of sessions had ≥ 1 engagement challenge occur. In parent only and youth + parent sessions, engagement challenges were observed in fewer sessions (30.47%). When examining the association between engagement challenges and therapist delivery of EBP strategies, there was no significant association with occurrence of youth engagement challenges and EBP strategies delivered (indexed by both observer or therapist report). For parent challenges, the relationship was nonsignificant using therapist-reported EBP delivery. In contrast, parent engagement challenges were positively associated with observer-rated therapist delivery of EBP strategies (b=.15; p=.04). This relationship seems to be driven by parents expressing concerns about the relevance/acceptability/helpfulness of the EBP.

**Conclusions**

Overall, these findings imply that youth/parent behaviors that we may conceptualize as engagement challenges do not necessarily derail therapists from EBP delivery in session. Future analyses will further examine the relationship thematically by types of engagement challenges. Discussion will focus on therapist perceptions of client behaviors as engagement challenges, and whether they can be deemed opportunities for alliance building.

**References**

1. Chu BC, Kendall PC. Positive association of child involvement and treatment outcome within a manual-based cognitive-behavioral treatment for children with anxiety. J Consult Clin Psychol. 2004;72(5):821-829. doi:10.1037/0022-006X.72.5.821.

2. Becker KD, Boustani M, Gellatly R, Chorpita BF. Forty years of engagement research in children’s mental health services: multidimensional measurement and practice elements. J Clin Child Adolesc Psychol. 2018;47(1):1-23. doi:10.1080/15374416.2017.1326121.

## A84 Therapist characteristics as predictors of perceptions of Evidence-Based Assessment (EBA)

### Kenny Le^1^, Lauren Brookman-Frazee^2^, Joyce Lui^1^, Mary Kuckertz^2^, Anna Lau^1^

#### ^1^University of California, Los Angeles, Los Angeles, CA, USA; ^2^University of California, San Diego, La Jolla, CA, USA

##### **Correspondence:** Kenny Le (kennyle@psych.ucla.edu)

**Background**

Evidence-based assessment (EBA) can improve clinical outcomes, and it is embedded in many evidence-based practices (EBP) [1]. However, EBA is not well implemented in community practice due to perceived low usefulness [2]. Additionally, therapist characteristics such as non-psychology disciplines and years of practice may be associated with negative EBA attitudes [3]. Research is needed on factors that predict EBA use in community practice and how attitudes change with exposure to EBP. The current study examined how therapist characteristics, use of EBA within the delivery of EBPs, and interactions between these variables may be associated with subsequent perceptions of EBA.

**Materials and Methods**

Therapists (n=117) in an initial survey reported on their use of clinical dashboards, an EBA strategy for repeated assessments of client outcomes to inform treatment planning. They reported on their perceptions of EBA in another survey approximately 10 months later. Separate linear regression models were conducted for each of the four dimensions of EBA perceptions: clinical utility, practicality, benefit to clients and treatment, and harm to clients.

**Results**

Results showed that dashboard use significantly predicted perceptions of clinical utility (b=-0.100, p=0.006). Theoretical orientation moderated the relationship between dashboard use and clinical utility (b=0.107, p=0.028). For therapists who did not identify as having a cognitive/behavioral orientation in their practice, more extensive dashboard use predicted lower perceptions of clinical utility of EBA. For other EBA perceptions, years of practice significantly predicted perceived practicality (b=0.037, p=0.020), where longer years of practice predicted positive perceptions of EBA. Therapist discipline also significantly predicted perceived harm of EBA for clients (b=-0.495, p=0.022), where having a psychology discipline predicted lower perceived harm relative to other disciplines.

**Conclusions**

These results replicate therapist characteristics as important predictors of EBA perceptions and suggest that on-the-job use of EBA strategies can predict future perceptions. However, for some clinicians (non-cognitive/behavioral), mandated use of EBA may further entrench negative perceptions. Non-psychologists and less experienced therapists perceived EBA as burdensome and more harmful to therapeutic process perhaps due to inefficient training or support. Future studies could examine implementation strategies that may promote positive perceptions of EBA and positive outcomes of EBA use for clients.

**References**

1. Scott K, Lewis CC. Using Measurement-Based Care to enhance any treatment. Cogn Behav Pract. 2015; 22(1):49-59. doi:10.1016/J.CBPRA.2014.01.010

2. Jensen-Doss A. Practice involves more than treatment: How can evidence-based assessment catch up to evidence-based treatment? Clin Psychol Sci Pract. 2011;18(2):173-177. doi:10.1111/j.1468-2850.2011.01248.x

3. Jensen-Doss A, Hawley KM. Understanding barriers to evidence-based assessment: Clinician attitudes toward standardized assessment tools. J Clin Child Adolesc Psychol. 2010;39(6):885-896. doi:10.1080/15374416.2010.517169

## A85 Looking beyond the clinic door: examining the relationship between clinic neighborhood characteristics and therapist emotional exhaustion in a large-scale implementation effort

### Mary Kuckertz^1^, Anna Lau^2^, Teresa Lind^1^, Kenny Le^2^, Mojdeh Motamedi^1^, Lauren Brookman-Frazee^1^

#### ^1^University of California, San Diego, La Jolla, CA, USA; ^2^University of California, Los Angeles, Los Angeles, CA, USA

##### **Correspondence:** Mary Kuckertz (mkuckertz@ucsd.edu)

**Background**

In an effort to increase the use of evidence-based practices (EBPs) in community mental health settings, large-scale implementation efforts are becoming increasingly common, prompting efforts to better understand determinants of implementation [1-2]. While much attention has been given to the inner and outer organizational and leaderships contexts that influence implementation efforts, [3-4] there has been less focus on the links between therapist factors and the community neighborhood in which they work. Clients from high risk neighborhoods have been shown to present with more severe symptoms [5] and an increased number of emergent life events that can impact the treatment process [6]. This may influence implementation efforts in the form of therapist burnout, which is linked to higher turnover [7].

**Materials and Methods**

The current study utilized survey data from therapists in Los Angeles County collected within the context of a system-driven multiple EBP implementation effort as well as publicly available data published by the Los Angeles Times “Mapping L.A.” project [8] (e.g., mean income, race/ethnicity, home ownership, education etc.). Clinic addresses were used to identify the neighborhood of the clinic as determined by the “Mapping L.A.” project, and neighborhood characteristics were collected and matched to survey data. Multilevel modeling was used (level 1=therapist, level 2=program, level 3=neighborhood) to examine the relationship between therapist emotional exhaustion and characteristics of the community of the clinic.

**Results**

Analyses showed population density (β=.14, p<.05), median income (β=-.30, p<.05), and home ownership (β=.30, p<.05) of the neighborhood surrounding the clinic to be significant predictors of therapist emotional exhaustion.

**Conclusions**

Results can help target specific implementation supports towards clinics with therapists at higher risk of emotional exhaustion, thus potentially improving the implementation of EBPs.

**References**

1. McHugh RK, Barlow DH. The dissemination and implementation of evidence-based psychological treatments. A review of current efforts. Am Psychol. 2010;65(2):73–84.

2. Starin AC, Atkins MS, Wehrmann KC, Mehta T, Hesson-McInnis MS, Marinez-Lora A, Mehlinger R. Moving science into state child and adolescent mental health systems: Illinois’ evidence-informed practice initiative. J Clin Child Adolesc Psychol. 2014;43(2):169–178.

3. Aarons GA, Farahnak LR, Ehrhart MG. Leadership and strategic organizational climate to support evidence-based practice implementation. In: Beidas RS, Kendall PC, eds. Dissemination and implementation of evidence-based practices in child and adolescent mental health. New York, NY: Guilford Press; 2014: p. 82–97.

4. Aarons GA, Hurlburt M, Horwitz SM. Advancing a conceptual model of evidence-based practice implementation in public service sectors. Adm Policy Ment Health. 2011;38(1):4–23.

5. Anakwenze U, Zuberi D. Mental health and poverty in the inner city. Health Soc Work. 2013;38(3):147-157.

6. Hatch S, Dohrenwend B. Distribution of traumatic and other stressful life events by race/ethnicity, gender, SES and age: A review of the research. Am J Community Psychol. 2007;40(3-4):313–332.

7. Johnson J, Hall LH, Berzins K, Baker J, Melling K, Thompson C. Mental healthcare staff well-being and burnout: A narrative review of trends, causes, implications, and recommendations for future interventions. Int J Ment Health Nurs. 2018;27(1):20-32.

8. Los Angeles Times. Mapping L.A. neighborhoods. 2019; http://maps.latimes.com/neighborhoods/.

## A86 Monitoring treatment engagement: how do providers know when youth and families are engaged in or disengaged from treatment?

### Ellie Wu^1^, Kimberly Becker^1^, Anna Hukill^1^, Bruce Chorpita^2^

#### ^1^University of South Carolina, Columbia, SC, USA; ^2^University of California, Los Angeles, Los Angeles, CA, USA

##### **Correspondence:** Ellie Wu (ew17@email.sc.edu)

**Background**

Poor engagement of youth and families in mental health services is a significant barrier to implementing psychosocial interventions, given that more than 50% of youth drop out of services before the completion of treatment. Detecting engagement problems early is a critical step in preventing premature termination and promoting successful implementation of psychosocial interventions in community settings. Despite this, little is known about how providers assess client engagement in therapy in the absence of structured feedback. The current study examines the indicators that providers use to detect engagement and disengagement in a variety of community mental health settings.

**Materials and Methods**

As a part of a training workshop, 39 mental health providers were asked to report a case example that represented an engagement challenge, a case example that represented an engagement success, and indicators used to assess engagement for each case. Participating providers worked primarily in schools, as well as community mental health centers, juvenile detention centers, and private practices. Case examples and described indicators were coded based on five domains of engagement (relationship, expectancy, attendance, clarity, and homework).

**Results**

Frequencies of coded engagement indicators were examined across engagement challenge and engagement success case examples. Results showed that low attendance was the most commonly reported indicator of disengagement, making up 75% of the indicators coded in the engagement challenge case examples. On the contrary, homework completion was the most commonly reported indicator of positive engagement, making up 69% of the indicators in the engagement success case examples. A figure will show the distribution of the other engagement domains across coded indicators of engagement and disengagement.

**Conclusions**

The purpose of this study was to investigate how providers detect engagement during treatment. While these results are limited due to small sample size and reporting single case examples, our study suggests that providers focus on attendance when assessing client disengagement, and homework completion when assessing client engagement. These results highlight an opportunity to train providers in understanding the multidimensional nature of engagement, so that they may address signs of attitudinal disengagement before they lead to disruptions in attendance and treatment dropout.

## A87 Lay counselor burnout and turnover across systems: are there differences that are important for implementation of task-sharing approaches?

### Leah Lucid^1^, Prerna Martin^1^, Christine L. Gray^2^, Rosemary Meza^1^, Augustine I. Wasonga^3^, Kathryn Whetten^2^, Shannon Dorsey^1^

#### ^1^University of Washington, Seattle, WA, USA; ^2^Duke University, Durham, NC, USA; ^3^ACE Africa Kenya, Bungoma, Kenya

##### **Correspondence:** Leah Lucid (llucid@uw.edu)

**Background**

Less than 1% of children in low- and middle-income countries (LMICs) with mental healthcare needs will receive treatment, in part due to a dearth of trained providers. Task-sharing, in which lay counselors deliver treatment, is an implementation solution; however, more research is needed on counselors’ experience in this role. Although providing trauma treatment has been linked to provider burnout in the US, to our knowledge no one has examined this question with lay counselors in LMICs.

**Materials and Methods**

The present study compares burnout and turnover between lay counselors in two systems in Kenya. Lay counselors included 60 teachers and community health volunteers (CHVs) delivering group-based Trauma-focused Cognitive Behavioral Therapy (TF-CBT) as part of a large NIMH-funded trial. These lay counselors embedded in different systems, with differing supports, role definitions, and time demands, may have differing experiences of adding a counseling role to their existing one (as teacher or CHV). Immediately after completing TF-CBT training, the lay counselors reported their baseline job burnout and turnover intention for their original role as either a teacher or CHV. Approximately six months later, after leading two rounds of TF-CBT groups, they completed post-implementation surveys assessing constructs of interest. Analyses used independent samples t-tests to determine if there were significant differences between teacher and CHV reports.

**Results**

At baseline, CHVs reported significantly higher burnout in their original role than teachers (p<.001) but there was no difference in turnover intention. Post-implementation, teachers reported significantly higher counseling-specific burnout (p=0.005) and compassion fatigue (p=0.007). They also reported higher job turnover intention (p=0.003) than CHVs. There were no differences for post-implementation compassion satisfaction (p=0.91) or intention to continue their counselor role (p=0.04).

**Conclusions**

Our findings contribute to the limited literature on the lived experience of lay counselors providing mental health treatment to families in LMICs. Some aspects of experience differ by system, and these differences may suggest points for intervention to retain lay counselors for scale-up and sustainment or to determine which systems may be more viable for “adding” a counseling role. Retaining lay counselors after investing in their training is critical for efforts aimed at reducing the substantial treatment gap in LMICs.

**Trial Registration:** Clinicaltrials.gov NCT03243396

## A88 Maintaining community partnership and program fidelity while replicating a community-based mental health intervention: a case study of the New Haven MOMS Partnership® replication in Bridgeport, Connecticut, New York City, and Washington D.C

### Sonia Taneja, Megan Smith

#### Yale School of Medicine, New Haven, CT, USA

##### **Correspondence:** Sonia Taneja (sonia.taneja@yale.edu)

**Background**

The Mental health Outreach for MotherS (MOMS) Partnership is a community-academic partnership in New Haven responsible for developing a maternal mental health intervention – a cognitive behavioral group therapy course co-led by a clinician and a community mental health worker at a convenient, neighborhood hub site. MOMS is in the process of expanding to Bridgeport, Connecticut; New York City; and Washington D.C. This expansion highlights the interplay of two common paradigms of program replication – designing program components using community-based participatory research methods and implementing programs with a high degree of fidelity. These two goals are often seen as contradictory, but successful programs are known to appraise both. Our first objective is thus to describe MOMS replication through community partners, identifying which components have been malleable based on community feedback and which were static. Our second objective is to provide the framework to measure program fidelity to the original model.

**Materials and Methods**

Three case studies are presented: the first two discuss lessons learned from early program development in Bridgeport and New York, beginning with iterative co-design of a needs assessment. The third examines D.C. MOMS beginning with the needs assessment distributed through the Technical Assistance for Needy Families (TANF) program. Results of a structural fidelity assessment in D.C., which operationalizes fidelity components including content and process, participant engagement, adherence to goals and needs assessment and theory of change, and acceptability of programs, are also provided.

**Results**

Insights from these case studies span issues of fostering and maintaining positive relationships with community groups, identifying government partners through which replication can be sustainable, but still acceptable to community members, managing capacity constraints particularly in environments that are saturated with nonprofit programming, navigating issues of population representation, and overcoming unforeseen challenges based on context.

**Conclusions**

Centering community nuance while replicating successful mental health interventions is an essential, iterative, and difficult process. Experiences of the early career investigators at MOMS while replicating program in three new cities provide reflections on lessons learned when entering new communities ethically and effectively, learning how to work towards program fidelity while valuing responsiveness to community need.

## A89 Applying a causal model to the implementation of evidence-based practice for autistic adults in community settings

### Brenna Maddox, Rinad S. Beidas, Jessica Fishman, Samantha Crabbe, David Mandell

#### University of Pennsylvania, Philadelphia, PA. USA

##### **Correspondence:** Brenna Maddox (maddoxb@upenn.edu)

**Background**

Cognitive-behavioral therapy (CBT) can improve anxiety and depression in autistic adults [1], who frequently struggle with these co-occurring psychiatric conditions [2]. Most autistic adults do not receive CBT, however, because of a lack of clinicians who are willing and able to treat them. We applied the Theory of Planned Behavior (TPB) [3], a leading model of behavior change, to examine malleable factors that may influence community clinicians’ use of CBT for autistic adults with anxiety or depression. These factors can be targeted with tailored implementation strategies to improve implementation of evidence-based practice [4].

**Materials and Methods**

One hundred clinicians completed an online survey. We used standardized procedures from social psychology to measure clinicians’ intentions, attitudes, norms, and self-efficacy, [5] and adapted them to focus on using CBT with adult clients (both autistic and non-autistic) who present for anxiety or depression treatment.

**Results**

Clinicians reported weaker intentions (p = .001, d = .34), less favorable attitudes (p < .001, d = .69), less descriptive normative pressure (p < .001, d = .39), less injunctive normative pressure (p < .001, d = .66), and worse self-efficacy (p < .001, d = .81) to start CBT with autistic adults than with non-autistic adults. The only significant predictor of intentions to begin CBT with clients (both autistic and non-autistic) who present for anxiety or depression treatment was clinicians’ attitudes (p < .001), with more favorable attitudes predicting stronger intentions.

**Conclusions**

For the purposes of this study, attitudes refer to the clinicians’ perceived advantages and disadvantages of starting CBT with their adult clients with anxiety or depression. This concept is similar to the implementation science constructs of “acceptability” and “appropriateness” [4,6]. Knowing that clinicians’ attitudes strongly predicted intentions is valuable for designing effective, tailored implementation strategies to increase clinicians’ adoption of CBT for autistic adults. For example, an implementation strategy targeting attitudes could include message content to change thinking around the perceived fit of CBT with autistic adults. Social psychologists have successfully used this type of approach to change attitudes about complex behaviors, [5] but it has not yet been applied to improving the implementation of evidence-based practice for autistic adults.

**References**

1. Spain D, Sin J, Chalder T, Murphy D, Happé F. Cognitive behaviour therapy for adults with autism spectrum disorders and psychiatric co-morbidity: a review. Res Autism Spect Dis. 2015;9(1):151-162.

2. Buck TR, Viskochil J, Farley M, Coon H, McMahon WM, Morgan J, Bilder DA. Psychiatric comorbidity and medication use in adults with autism spectrum disorder. J Autism Dev Disord. 2014;44(12):3063-3071.

3. Ajzen I. The theory of planned behavior. Organ Behav Hum Decis Process. 1991;50(2):179-211.

4. Fishman JM, Lushin V, Lawson G, et al. A theory for implementation prediction (TIP): applying causal models of behavior change to specific evidence-based practices. Manuscript under review.

5. Fishbein M, Ajzen I. Predicting and changing behavior: the reasoned action approach. New York, NY: Psychology Press; 2010.

6. Proctor E, Silmere H, Raghavan R, Hovmand P, Aarons G, Bunger A, Griffey R, Hensley M. Outcomes for implementation research: conceptual distinctions, measurement challenges, and research agenda. Adm Policy Ment Health. 2011;38(2):65-76.

## A90 Harnessing implementation science with community-based social service organizations to address depression and social isolation for older adults living in poverty

### Correspondence: Lesley Steinman (lesles@uw.edu)

#### University of Washington, Seattle, WA, USA

**Background**

Late-life depression (LLD) is a major public health issue that impacts older adults, families, communities, and health systems. Often unrecognized or undertreated among older populations, those living in poverty experience disparities in access to LLD care. PEARLS is a home-based collaborative care model (CCM) for LLD developed with social service community-based organizations (CBOs) to reach underserved older adults. Since demonstrating effectiveness via RCT in 2004, [1] we have partnered with CBOs to apply implementation science (IS) tools and strategies to improve PEARLS delivery. One of our current projects is to evaluate whether and how PEARLS may help address the recent “epidemic” of social isolation [2] in five US sites.

**Materials and Methods**

The PEARLS Connect Study is a concurrent mixed methods evaluation. Implementation data will be collected via surveys and semi-structured interviews with 10 CBO administrators and 30 PEARLS practitioners. The interview guide asks about implementation strategies, determinants, adaptations, and outcomes. We will use framework analysis [3-4] to map text data to a priori domains from IS frameworks – ERIC implementation strategies, [5] EPIS implementation determinants (Aarons), QUERI Adapted Stirman [6] Adaptation Framework (QASAF) [7] and implementation outcomes [8]. We will also use inductive thematic analysis to pull out any other key implementation themes that emerge from the conversations with practitioners.

**Results**

Data are currently being collected (Spring 2019) and will be analyzed in Summer 2019. We will report on implementation strategies (planning, educating, financing, restructuring, quality management, policy); practitioner, organization, and contextual determinants influencing implementation; local adaptations (what, when, how, why, by whom, where, and what impact the modification had). We will also share whether PEARLS is acceptable, feasible, and appropriate as an intervention to increase social connectedness. Local CBO partners will share how partnering with implementation scientists has impacted their practice and policymaking, including facilitators, barriers and opportunities to partnership and application of IS.

**Conclusions**

Many of the IS frameworks applied in this study have been developed with clinical or public mental health settings. This scaling-out [9] evaluation will share learnings from researchers and practitioners who are applying IS to improve practice and equity [10] for older adults living in poverty.

**References**

1. Ciechanowski P, Wagner E, Schmaling K, Schwartz S, Williams B, Dierhr P, Kulzer J, Gray S, Collier C, LoGerfo J. Community-integrated home-based depression treatment in older adults: a randomized controlled trial. JAMA (2004) 291(13):1569–77.

2. Holt-Lunstad J. The potential public health relevance of social isolation and loneliness: prevalence, epidemiology, and risk factors. Public Policy Aging Rep. 2017;27(4):127-130.

3. Gale NK, Heath G, Cameron E, Rashid S, Redwood S. Using the framework method for the analysis of qualitative data in multi-disciplinary health research. BMC Med Res Methodol. 2013;13(1):117.

4. Parkinson S, Eatough V, Holmes J, Stapley E, Midgley N. Framework analysis: a worked example of a study exploring young people’s experiences of depression. Qual Res Psychol. 2016;13(2):109-129.

5. Powell BJ, McMillen JC, Proctor EK, Carpenter CR, Griffey RT, Bunger AC, Glass JE, York JL. A compilation of strategies for implementing clinical innovations in health and mental health. Med Care Res Rev. 2012;69(2):123-157.

6. Stirman SW, Miller CJ, Toder K, Calloway A. Development of a framework and coding system for modifications and adaptations of evidence-based interventions. Implement Sci. 2013;8:65

7. Rabin BA, McCreight M, Battaglia C, Ayele R, Burke RE, Hess PL, Frank JW, Glasgow RE. Systematic, multimethod assessment of adaptations across four diverse health systems interventions. Front Public Health. 2018;6:102.

8. Proctor E, Silmere H, Raghavan R, Hovmand P, Aarons G, Bunger A, Griffey R, Hensley M. Outcomes for implementation research: conceptual distinctions, measurement challenges, and research agenda. Adm Policy Ment Health. 2011;38(2):65-76. doi:10.1007/s10488-010-0319-7.

9. Aarons GA, Sklar M, Mustanski B, Benbow N, Brown CH. “Scaling-out” evidence-based interventions to new populations or new health care delivery systems. Implement Sci. 2017;12(1):1-13. doi:10.1186/s13012-017-0640-6.

10. Chinman M, Woodward EN, Curran GM, Hausmann LRM. Harnessing implementation science to increase the impact of health disparity research. Med Care. 2017; 55(Suppl 9 2): S16–S23.

## A91 Reflections on a decade of policy/practice-driven implementation research: strategies for meaningful collaboration

### Correspondence: Sarah Kaye (sarah@kayeimplementation.com)

#### Kaye Implementation & Evaluation, LLC, Washington, DC, USA

**Background**

This presentation aims to discuss three questions of interest to the Society for Implementation Research Collaboration (SIRC) at the 2019 conference: (1) What can implementation researchers, practitioners and intermediaries learn from each other? (2) How can we adapt lessons learned from implementation science in ways that are culturally and contextually appropriate? (3) What are examples of strategies for collaboration between policy makers/funders and implementation experts? Two-way communication between the research world and the real world is vital to minimizing the gap between research and practice. Understanding the needs of policy makers and practitioners is critical to producing research findings that are relevant to the users that studies are intended to support. Moreover, research projects benefit from acknowledging policy, practice, and community expertise--and recognizing community members’ meaningful contributions to a better understanding of implementation strategies, processes, and causal mechanisms.

**Materials and Methods**

Drawing on principles from community-engaged research [1] and research/evaluation capacity-building [2], this presentation offers strategies that implementation researchers might consider when partnering with communities.

**Results**

Examples of these strategies include: Co-designing a theory of change for the intervention(s) and implementation; Utilizing sequential explanatory research designs [3] through a process of read, listen, share, discuss, adapt; Offering community empowerment opportunities through voice and choice about design, measures, and theories/frameworks; Addressing questions and requirements that are critical to policy makers and practitioners; Identifying dissemination strategies that meet the needs of communities.

**References**

1. Wallerstein N, Duran B. Community-based participatory research contributions to intervention research: the intersection of science and practice to improve health equity. Am J Public Health. 2010;100 (Suppl 1):S40-6.

2. Labin SN, Duffy JL, Meyers DC. A research synthesis of the evaluation capacity building literature. Am J Eval. 2012;33(3):307–338. https://doi.org/10.1177/1098214011434608.

3. Creswell JW, Clark VLP. Designing and conducting mixed methods research studies, Second Ed. SAGE Publications. Thousand Oaks, CA; 2010.

## A92 Engaging stakeholders in the development of an intervention to systematically tailor implementation strategies

### Amber Haley^1^, Sheila Patel^1^, Jamie Guillergan^1^, Lisa Amaya Jackson^2^, Mellicent Blythe^3^, Beverly Glienke^3^, Alicia Sellers^4^, Jennifer Grady^4^, Byron J. Powell^5^

#### ^1^University of North Carolina at Chapel Hill, Chapel Hill, NC, USA; ^2^Duke University, Durham, NC, USA; ^3^North Carolina Child Treatment Program, Durham, NC, USA; ^4^National Center for Child Traumatic Stress, Durham, NC, USA; ^5^Washington University in St. Louis, St. Louis, MO, USA

##### **Correspondence:** Amber Haley (ahaley@live.unc.edu)

**Background**

Stakeholder engagement is often considered critical to implementation science [1]. This study illustrates the value of engaging a wide variety of stakeholders in the development of an implementation intervention. This project was funded by the National Institutes of Mental Health to develop and pilot the Collaborative Organizational Approach to Selecting and Tailoring Implementation Strategies (COAST-IS).

**Materials and Methods**

The COAST-IS intervention will equip organizations to use Intervention Mapping to select and tailor implementation strategies to address site-specific determinants of treatment implementation and sustainment [2]. Intervention Mapping is a multistep process that incorporates theory, evidence, and stakeholder perspectives to ensure that intervention components effectively address key determinants of change [3]. The first year of the grant focused on engaging national and local experts, organizational leaders, clinicians, caregivers, and youth in the development of COAST-IS for a trauma-focused treatment. The approaches to engagement in this project range from full partnership to stakeholder consultation. The project is being conducted in partnership with leadership from the UCLA-Duke National Center for Child Traumatic Stress (NCCTS) and the North Carolina Child Treatment Program (NC CTP). NCCTS and NC CTP continue to shape project planning and intervention development through continuous feedback on intervention structure, organizational assessment, and dissemination planning.

**Results**

The research team worked with NC CTP to convene an Organizational Advisory Board (OAB) to solicit the perspectives of organizational stakeholders similar to potential research participants. OAB members reviewed draft intervention materials and provided feedback on the structure and content of the COAST-IS intervention. They continue to influence intervention material development. To incorporate the perspectives of families and youth during intervention development, NCCTS leadership connected the research team with two existing client groups. The Family and Youth Insight Advisory Group and the Youth Task Force met with the research team to discuss barriers to their engagement in trauma-focused treatments and recommend strategies to address these barriers. The research team synthesized these recommendations to share with future intervention participants to promote client-focused implementation.

**Conclusions**

This study illustrates the potential impact of engaging diverse stakeholders in the development of implementation interventions. The study team will continue engagement efforts during intervention testing, planning for future research, and dissemination.

**Trial Registration:** Clinicaltrials.gov NCT03799432

**References**

1. Chambers DA, Azrin ST. Research and services partnerships: partnership: a fundamental component of dissemination and implementation research. Psychiatr Serv. 2013;64(6):509-511.

2. Powell BJ, Beidas RS, Lewis CC, Aarons GA, McMillen JC, Proctor EK, Mandell DS. Methods to Improve the Selection and Tailoring of Implementation Strategies. J Behav Health Serv Res. 2017 Apr;44(2):177-194. doi: 10.1007/s11414-015-9475-6.

3. Eldredge LKB, Markham CM, Ruiter RA, Kok G, Fernandez ME, Parcel GS. Planning health promotion programs: an intervention mapping approach. Hoboken, NJ: John Wiley & Sons;2016.

## A93 Building capacity in advance care planning: an example of collaboration amongst policy-makers, care providers, community organizations, and implementation researchers

### Correspondence: Robin Urquhart (robin.urquhart@nshealth.ca)

#### Dalhousie University, Halifax, Nova Scotia, Canada

**Background**

Advance care planning (ACP) is the process by which patients, alongside their healthcare providers, consider options about future healthcare decisions. Although ACP is associated with improved outcomes as people near end-of-life [1-3], many providers report discomfort with ACP and subsequent goals-of-care (GOC) conversations [4]. There are also many organizational, community, and system barriers to these conversations [5-6]. Changing practice, and ultimately improving patient/family outcomes, will require collaborative efforts from multi-level/sector partners. Through collaboration amongst government, the provincial cancer agency, care providers, a community-based organization, and an implementation scientist, we sought to develop provider capacity in initiating, and increase the frequency of, ACP/GOC conversations with cancer patients.

**Materials and Methods**

Informed by the knowledge-to-action framework [7] and data gathered from various sources, including formal research studies and local context, we co-designed an intervention (communication skills training workshop, clinician guides and documentation tools, and patient/family guide). We tested the intervention with 51 providers in oncology, palliative, and primary care; evaluated it using post-workshop surveys, focus groups, and chart reviews; and adapted it via iterative team dialogue and debriefing.

**Results**

Data from the first two (of five) workshops revealed that most providers were uncomfortable with ACP/GOC conversations and it was premature, at that point, to expect them to complete documentation. Thus, we focused on refining/enhancing the workshop and encouraging use of the clinician/patient guides. Our findings demonstrated increased provider confidence across most ACP/GOC domains and an increased number of ACP/GOC conversations with patients, and provided critical insight for scale-up (e.g., train-the-trainer strategies, integration with existing education events). The adapted intervention was subsequently implemented province-wide. Team reflection and debriefing revealed at least 6 factors critical to successful implementation: 1. underpinned by research and local evidence; 2. driven collaboratively by multi-level/sector stakeholders; 3. guided by a plan but a willingness to adapt as needed; 4. ongoing evaluation; 5. clinical champions; and 6. a dedicated coordinator to bring to all together.

**Conclusions**

Collaboration amongst multi-level/sector stakeholders has the potential to change clinical practice. Evidence from multiple sources is critical to designing an evidence-based, locally-adapted intervention and convincing a myriad of stakeholders to support it.

**References**

1. Detering KM, Hancock AD, Reade MC, Silvester W. The impact of advance care planning on end of life care in elderly patients: randomised controlled trial. BMJ 2010;340:c1345

2. Lautrette A, Darmon M, Megarbane B, Joly LM, Chevret S, Adrie C, Barnoud D, Bleichner G, Bruel C, Choukroun G, Curtis JR, Fieux F, Galliot R, Garrouste-Orgeas M, Georges H, Goldgran-Toledano D, Jourdain M, Loubert G, Reignier J, Saidi F, Souweine B, Vincent F, Barnes NK, Pochard F, Schlemmer B, Azoulay E. A communication strategy and brochure for relatives of patients dying in the ICU. N Engl J Med 2007;356:469-78

3. Wright AA, Zhang B, Ray A, Mack JW, Trice E, Balboni T, Mitchell SL, Jackson VA, Block SD, Maciejewski PK, Prigerson HG. Associations between end-of-life discussions, patient mental health, medical care near death, and caregiver bereavement adjustment. JAMA 2008;300:1665-73

4. Morrison RS, Morrison EW, Glickman DF. Physician reluctance to discuss advance directives. An empiric investigation of potential barriers. Arch Intern Med 1994;154:2311-8

5. Batchelor F, Hwang K, Haralambous B, Fearn M, Mackell P, Nolte L, Detering K. Facilitators and barriers to advance care planning implementation in Australian aged care settings: a systematic review and thematic analysis. Australas J Ageing. 2019;38(3):173-181. doi: 10.1111/ajag.12639.

6. Howard M, Bernard C, Klein D, Elston D, Tan A, Slaven M, Barwich D, You JJ, Heyland DK. Barriers to and enablers of advance care planning with patients in primary care: survey of health care providers. Can Fam Physician. 2018; 64(4):e190-e198

7. Graham ID, Logan J, Harrison MB, Straus SE, Tetroe J, Caswell W, Robinson N. Lost in knowledge translation: time for a map? J Contin Educ Health Prof. 2006;26(1):13-24

## A94 Comparing state mental health agency and state insurance agency directors’ perspectives on the benefits and barriers to inter-agency collaboration related to implementation of federal mental health parity policy

### Katherine Nelson, Jonathan Purtle

#### Drexel University, Philadelphia, PA, USA

##### **Correspondence:** Katherine Nelson (kml383@drexel.edu)

**Background**

There is substantial state-level variation in the implementation of federal mental health parity policy—which requires that insurance companies provided equal coverage for mental and physical health care [1]. One possible reason for this variation could be differences in how state mental health and insurance agencies are collaborating to support implementation [2]. This study aimed to: characterize perceptions of the benefits and barriers to inter-agency collaboration related to implementation of federal parity policy, and compare how these perceptions differ between state mental health agency and state insurance agency directors.

**Materials and Methods**

Web-based surveys of state mental health agency directors (n=43, response rate= 84%) and state insurance agency directors (n=34, response rate= 67%) were conducted in 2017. One item assessed the perceived benefit of collaboration between two the agencies and five items assessed specific barriers to collaboration. All items were explicitly about implementation of federal mental health parity policy. Bivariate analyses assessed differences in the perceptions between state mental health agency and state insurance agency directors.

**Results**

A significantly higher proportion of state mental health agency directors thought that there would be a benefit to inter-agency collaboration than state insurance agency directors (95.4% vs. 67.7%, χ2 p= .001). A significantly higher proportion of state mental health agency directors identified different agency culture (e.g., “norms, values”) (51.2% vs. 24.2%, χ2 p= .017) and different agency terminology (51.2% vs. 18.2%, χ2 p=.003) as major barriers to inter-agency collaboration than state insurance agency directors. For all five barriers to inter-agency collaboration, the proportion of respondents identifying each as a major barrier was higher among state mental health agency directors that state insurance agency directors.

**Conclusions**

State mental health agency directors believe that there is more benefit to inter-agency collaboration related to the implementation of federal mental health parity policy than state insurance agency directors, but also believe that there are more barriers to collaboration. Implementation strategies such as consensus discussions, facilitation between state mental health agencies and state insurance agencies, and the development of an implementation glossary could improve inter-agency collaboration and enhance the implementation of federal mental health parity policy [2-3].

**References**

1. The Kennedy Forum. Evaluating state mental health and addiction parity statutes: a technical report. 2018. https://chp-wp-uploads.s3.amazonaws.com/www.paritytrack.org/uploads/2018/09/KF-Evaluating-State-Mental-Health-Report-0918_web.pdf

2. Purtle J, Borchers B, Clement T, Mauri A. Inter-agency strategies used by state mental health agencies to assist with federal behavioral health parity implementation. J Behav Health Serv Res. 2018; 45(3):516-526.

3. Powell BJ, Waltz TJ, Chinman MJ, Damschroder LJ, Smith JL, Matthieu MM, Proctor EK, Kirchner JE. A refined compilation of implementation strategies: results from the Expert Recommendations for Implementing Change (ERIC) project. Implement Sci. 2015;10(1):21. doi: 10.1186/s13012-015-0209-1

## A95 A rapid review to inform implementation of a behavioral health intervention in primary care: methods and outcomes

### Madeline Larson, Mimi Choy-Brown, Scott Marsalis

#### University of Minnesota, Minneapolis, MN, USA

##### **Correspondence:** Madeline Larson (lars5424@umn.edu)

**Background**

Current methods or approaches used in scientific practice have failed to rapidly and rigorously integrate cultivated knowledge into feasible and sustainable real-world practices and policies [1]. Continuing to use traditional scientific approaches runs the risk of implementation science falling short of expectations [1]. Different methods are needed to foster the rapid integration of science and practice while maintaining rigor [1]. The purpose of this study is to conduct a rapid review of the literature to identify implementation determinants and strategies that impact adoption, implementation, and sustainability of family-based mental health interventions in primary care.

**Materials and Methods**

A rapid review will be conducted that involves a primary literature search of Medline, Embase, PsycInfo, CINAHL databases to identify existing implementation strategies used to foster the uptake and use of family-based interventions in primary care settings. A secondary search will be performed as an iterative process and included bibliographic and grey literature searches of reference lists, authors and specifically the journal, Implementation Science. A systematic approach to data extraction will be used to illuminate key determinants, strategies, and outcomes related to implementation.

**Results**

The results of the rapid review will be presented along with method parameters (e.g., time to complete, cost and resources, streamlining processes). Findings will directly inform an actionable plan to implement the intervention in primary care settings. Results will be used to inform stakeholder decisions regarding implementation supports provided during a multi-site rollout of FBCI in integrated primary care settings throughout the USA.

**Conclusions**

Rapid reviews can be used to integrate implementation research into practice or policy. While it is not exhaustive, rapid reviews provide a pragmatic and systematic approach to synthesizing evidence to inform decision-making in real-world efforts. Future work will compare results of rapid review to results of a gold-standard systematic review.

**Reference**

1. Glasgow RE, Chambers D. Developing robust, sustainable, implementation systems using rigorous, rapid and relevant science. Clin Transl Sci. 2012;5(1):48-55.

## A96 Rural primary care organizational change toward trauma-informed integrated primary care through community partnerships

### Deborah Moon^1^, Eve-Lynn Nelson^2^, Michelle Johnson-Motoyama^3^, Shawna Wright^4^, Becci Akin^4^

#### ^1^University of Pittsburgh, Pittsburgh, PA, USA; ^2^University of Kansas Medical Center, Kansas City, KS, USA; ^3^University of Ohio State, Columbus, OH, USA; ^4^University of Kansas, Kansas City, KS, USA

##### **Correspondence:** Deborah Moon (elpidamoon@gmail.com)

**Background**

Rural children are frequently exposed to adverse experiences, which are associated with negative long-term health outcomes [1-2]. Trauma-informed integrated primary care provides a model of care that takes into account the impact of social determinants of health both at the prevention and treatment levels [3]. Developing into trauma-informed integrated primary care involves complex organizational change processes that are challenging for rural primary care facilities burdened with multiple priorities [4]. Community partnership presents capacity building opportunities for rural primary healthcare clinics in such processes [5-6]. The purpose of this study was to examine facilitators and barriers in rural primary care organizational changes toward Trauma-Informed Integrated Primary Care based on theoretical frameworks of organizational change and implementation science, with an emphasis on partnering as a key process of the organizational change efforts.

**Materials and Methods**

This study was a community engaged organizational case study funded by HRSA, Doris Duke Foundation, and NY Community Trust Fund. The study was conducted in collaboration with a Federally Qualified Health Center in a rural community of a midwestern state, a non-profit organization, and an academic research and development center. Data collection involved key informant interviews, surveys, direct observation of formal and informal interactions with key stakeholders. Data analyses focused on identifying facilitating and/or interfering contexts and mechanisms in the organizational change efforts. This poster focuses on contexts and mechanisms pertaining to the partnering aspect.

**Results**

Facilitating contexts included shared mission and values, commitment to learning and innovation, increased awareness of social determinants of health among leadership, core work group, and transparent communication. The key facilitating mechanisms were leveraged resources and shared expertise that positively influenced change readiness. Major interfering contexts included limited understanding of the workflow, adaptability, and performance measurement. The key interfering mechanism was the culture clash that negatively influenced engaging key stakeholders.

**Conclusions**

Partnering is a key process in rural primary care capacity building to meet the complex healthcare needs of children in under-resourced communities through trauma-informed integrated care. Understanding facilitating and interfering contexts and mechanisms in partnering can inform the process of building strategic partnerships for collective actions toward building a healthy community in under-served regions.

**References**

1. Campbell JA, Walker RJ, Egede LE. Associations between adverse childhood experiences, high-risk behaviors, and morbidity in adulthood. Am J Prev Med. 2016;50(3):344-352.

2. Talbot JA, Szlosek M, Ziller EC. Adverse childhood experiences in rural and urban contexts. University of Southern Maine, Muskie School of Public Service, Maine Rural Health Research Center. 2016. PB-64.

3. Brown JD, King MA, Wissow LS. The central role of relationships with trauma-informed integrated care for children and youth. Acad Pediatr. 2017;17(7):S94-S101.

4. Hummer VL, Dollard N, Robst J, Armstrong MI. Innovations in implementation of trauma-informed care practices in youth residential treatment: a curriculum for organizational change. Child Welfare. 2010; 89(2):79.

5. Airhihenbuwa CO, Shisana O, Zungu N, BeLue R, Makofani DM, Shefer T, Smith E, Simbayi L. Research capacity building: a US-South African partnership. Glob Health Promot. 2011;18(2):27-35.

6. Chavis DM. Building community capacity to prevent violence through coalitions and partnerships. J Health Care Poor Underserved. 1995;6(2):234-245.

## A97 The impact of new national and state insurance policy on implementation of the collaborative care model for perinatal depression: the rubber hits the road for government policy to support behavioral health integration

### Ian Bennett^1^, Ashok Reddy^2,3^, Anna Ratzliff^1^, Stephanie Shushman^4^, Jay Wellington^5^

#### ^1^University of Washington, Seattle, WA, USA; ^2^Centers for Medicare and Medicare Services Innovation Center, Baltimore, MD, USA; ^3^VA Puget Sound Medical Center, Seattle, WA, USA; ^4^Community Health Plan of Washington, Seattle, WA, USA; ^5^UW Medicine Neighborhood Clinics, Seattle, WA, USA

##### **Correspondence:** Ian Bennett (ibennett@uw.edu)

**Background**

The collaborative care model (CoCM), is a highly evidence based complex intervention for management of common mental disorders in primary care. Despite decades of effort to disseminate and implement this model penetration into clinical practice is modest. A major obstacle is the lack of reimbursement for team care activities. In January 2018, a new mechanism for payment of the work of a CoCM team (G and CPT billing codes) was introduced by the US Centers for Medicare & Medicaid Services for Medicare recipients. Washington State has extended this mechanism to Medicaid recipients (Managed Care and fee for service). Perinatal depression (depression in pregnancy and the year postpartum), is the most common clinical maternal disorder. Untreated depression is associated with poor persistent developmental, learning, and mental health outcomes for children. Medicaid funds approximately 50% of perinatal care making the new CoCM codes a viable means of funding care for perinatal depression in this vulnerable population.

**Materials and Methods**

MInD-I (maternal infant dyad-implementation), is an NIMH funded (R01MH108548) implementation trial of CoCM for perinatal depression with five sites in Washington and thirteen outside.

**Results**

Despite the availability of CoCM CPT codes to support the work of team care for depression they have not been widely utilized even in settings with collaborative care already in place. The UW Neighborhood Clinic primary care network undertook steps needed to document and process these codes for billing. The availability of these billing codes was used to support implementation of CoCM for perinatal depression within four sites of this network. New administrative, work flow, and interdisciplinary elements of this care represented challenges.

**Conclusions**

Members of the panel will describe the national and state health policy goals underlying the promulgation of these codes and the current rate of utilization of these codes. The impact of these codes on implementation of CoCM for perinatal depression will be explored from the health system and primary care clinic level provider perspectives. The panel will provide a unique multi-level perspective on the impact of a novel funding policy on the implementation of a highly evidence based complex intervention requiring change in practice organization.

## A98 Active ingredients of implementation: examining the overlap between behaviour change techniques and implementation strategies

### Sheena McHugh^1^, Justin Presseau^2^, Courtney Leucking^3^, Byron J. Powell^4^

#### ^1^University College Cork, Cork, Ireland; ^2^Ottawa Hospital Research Institute, Ottawa, Ontario, Canada; ^3^University of North Carolina at Chapel Hill, Chapel Hill, NC, USA; ^4^Washington University in St. Louis, St. Louis, MO, USA

##### **Correspondence:** Sheena McHugh (s.mchugh@ucc.ie)

**Background**

Efforts to generate an evidence base for implementation strategies are frustrated by insufficient description [1]. The ERIC compilation names and defines implementation strategies [2]; however, further work is required to operationalise strategies to clearly describe the specific actions involved [1]. The purpose of this project is to examine the extent to which strategies can be specified according to behaviour change techniques [3], ‘active ingredients’ of interventions with the potential to change behaviour.

**Materials and Methods**

The primary data source was the definitions of 73 strategies contained in the ERIC compilation [2]. The definition of each strategy was deductively coded using the BCT Taxonomy, [3] which contains 93 discrete techniques with the potential to change behaviour. A typology was developed iteratively to categorise the extent of overlap between strategies and BCTs. Three implementation scientists independently rated their level of agreement with and confidence in the categorisation.

**Results**

During preliminary analysis, 86 BCTs were linked to 73 strategies. Five types of overlap were identified. 1) In 6 instances, there was a direct overlap between strategies and BCTs (e.g., strategy: remind clinicians, BCT: prompts and cues). 2) In 36 instances, there was at least 1 BCT clearly subsumed under the strategy description which could be used to guide initial operationalisation (e.g., strategy: clinical supervision, BCT: restructure social environment). 3) In 42 instances, a BCT(s) was probably subsumed under the strategy given its definition and/or title, but other BCTs were possible depending on how the strategy was operationalised (e.g., strategy: visit other implementation sites, BCT: social comparison). For 8 strategies, there were no BCTs clearly indicated in the strategy definition or title (e.g., strategy: make training dynamic). Finally, 14 strategies did not focus on behaviour change to support implementation (e.g., strategy: access new funding).

**Conclusions**

Many implementation strategies rely on assumptions and inference on the part of the intervention developer, be it researcher or practitioner, to apply them in a setting. This study is the first step towards moving from general descriptions of implementation strategies to full and consistent descriptions of their active ingredients. This is essential to understanding the mechanisms by which implementation strategies exert their effects.

**References**

1. Proctor EK, Powell BJ, McMillen JC. Implementation strategies: recommendations for specifying and reporting. Implementation Science. 2013;8(1):139.

2. Powell BJ, Waltz TJ, Chinman MJ, Damschroder LJ, Smith JL, Matthieu MM, Proctor EK, Kirchner JE. A refined compilation of implementation strategies: results from the Expert Recommendations for Implementing Change (ERIC) project. Implement Sci. 2015;10(1):21.

3. Michie S, Richardson M, Johnston M, Abraham C, Francis J, Hardeman W, Eccles MP, Cane J, Wood CE. The behavior change technique taxonomy (v1) of 93 hierarchically clustered techniques: building an international consensus for the reporting of behavior change interventions. Ann Behav Med. 2013; 46(1):81-95.

## A99 Unpacking and re-packing what we know about barriers and facilitators assessments

### Sobia Khan^1^, Julia Moore^1^, Byron J. Powell^2^

#### ^1^The Center for Implementation, Toronto, Ontario, Canada; ^2^Washington University in St. Louis, St. Louis, MO, USA

##### **Correspondence:** Sobia Khan (sobia.khan@thecenterforimplementation.com)

**Background**

Barriers and facilitators assessments (BFAs) are regarded as a key step in implementation, yet our understanding of how to conduct BFAs effectively has stalled. There is an abundance of literature on barriers and facilitators (BFs) to adopting a multitude of interventions and programs, but these studies do not offer any additional insight on how BFAs can be made more meaningful and rigorous. We argue that BFAs should be reconsidered in order to close important gaps in how barriers and facilitators impact intervention implementation.

**Materials and Methods**

We synthesized current approaches to conducting BFAs, and offered alternate methods and considerations for BFAs.

**Results**

BFAs published in the literature typically either report lists of BFs without describing specific relationships between BFs and implementation or clinical outcomes, or they report how the BFA contributed to program development, planning, adaptation, or evaluation (e.g., BFs were used to select strategies to implement the program). However, these studies have substantial limitations and do little to better interpret BFs, which is necessary to adapt programs and/or systematically develop implementation strategies. First, they are typically conducted before or after implementation, and do not explore the relationship between anticipated versus actual/experienced BFs. Second, they rely primarily upon self-report, although individuals tend to have a poor understanding of their own behavior. Third, they fail to examine dependencies between BFs (e.g., removing one barrier may uncover unintended consequences or “masked” barriers). BFAs would be enhanced by: 1) continuous assessment throughout implementation process; 2) considering their proximity to, and impact on, implementation; 3) integrating prioritization processes; 4) increased use of observation, qualitative, and mixed methods; and 5) integrating systems science methods that facilitate exploration of interdependencies.

**Conclusions**

Given the centrality of BFAs in implementation science, it is critical that we continue to examine how they can be improved methodologically and how they might yield more accurate and actionable data. This study advances the field by presenting concrete suggestions for how BFAs could be improved.

## A100 Demonstrating the value of coincidence analysis for identifying successful implementation strategies

### Sarah Birken^1^, Soohyun Hwang^1^, Laura Viera^2^, Emily Haines^1^, Tamara Huson^1^, Rebecca Whitaker^1^, Lawrence Shulman^3^, Deborah Mayer^1^

#### ^1^University of North Carolina at Chapel Hill, Chapel Hill, NC, USA; ^2^North Carolina Translational and Clinical Sciences Institute, University of North Carolina at Chapel Hill, Chapel Hill, NC, USA; ^3^Commission on Cancer, University of Pennsylvania, Philadelphia, PA, USA

##### **Correspondence:** Sarah Birken (sarah1@email.unc.edu)

**Background**

Coincidence analysis (CNA) is a configurational comparative method similar to qualitative comparative analysis, designed for causal inference when combinations of co-occurring conditions determine outcomes and multiple paths to one outcome may exist – as is often the case in implementation research. To demonstrate its usefulness in implementation research, we will use CNA to identify the strategies that cancer programs have used to develop comprehensive approaches to survivorship care plan implementation. The Commission on Cancer (CoC) requires cancer care providers to develop and deliver survivorship care plans (SCPs) to survivors and their primary care providers (PCPs). Cancer programs’ approaches to implementing SCPs in practice substantially vary, ranging from cursory (i.e., developing SCPs to meet requirements without delivering them to survivors or PCPs) to comprehensive (i.e., promoting adherence to screening and health behavior guidelines and recommended utilization of follow-up care).

**Materials and Methods**

We have characterized cancer programs’ approaches to implementing SCPs using semi-structured telephone interviews with providers/staff in 48 CoC-accredited cancer programs. We are using template analysis, combining a priori and emergent codes, and calibrating qualitative data for CNA purposes (e.g., the presence of a formal survivorship implementation committee). By the time of the conference, we will have used CNA to identify strategies that are unique to cancer programs with comprehensive approaches to implementing SCPs, using cancer programs with cursory approaches as a comparison group. The outcome of CNA will be a parsimonious list of strategies that are associated with comprehensive approaches to SCP implementation.

**Results**

Preliminary qualitative analyses suggest that programs varied in their approaches to SCP implementation. Key variables included human and financial resources and infrastructure, including standing committees and mechanisms of communication and garnering leadership support and provider buy-in. We will present CNA results at the conference.

**Conclusions**

CNA is a promising method for implementation research, where outcomes may be explained by combinations of co-occurring conditions, and when multiple paths to one outcome may exist. CNA findings are policy-relevant since they identify multiple combinations of strategies that diverse organizations may deem appropriate; findings from this study will inform more than 1500 CoC-accredited cancer programs with recommended SCP implementation strategies.

## A101 Exploring variability in implementation leadership and climate across organizational level

### Melina Melgarejo, Jessica Suhrheinrich

#### San Diego State University, San Diego, CA, USA

##### **Correspondence:** Melina Melgarejo (mmelgarejo@sdsu.edu)

**Background**

Nationwide, 576,000 students were served for Autism Spectrum Disorder (ASD) during the 2014-15 school year, an increase of 51% from 2007-08 [1]. Given the significant increase in demand for educational services for students with ASD, there is urgent demand to improve implementation and sustainment of evidence-based practices (EBP) in school settings. However, the organizational and leadership structure for school-based services for ASD is complex and involves a team of providers to account for the complexity of care needed [2-3]. Organizational culture and leadership have been found to impact EBP use in a variety of settings, but less is known about their impact in schools. As a first step toward tailoring implementation intervention for this context, the current proposal explores implementation leadership and implementation climate in relationship with provider factors.

**Materials and Methods**

Participants were 340 school-based providers and administrators who are involved in supporting students with ASD. Participants included 19 High-level administrators (Special Education Directors, District-level Administrators), 112 Mid-level specialists (Autism Specialist, Behavior Specialist, Program Specialist) 15 School-site principals or administrators, 153 Teachers and direct service providers (DSP) and 33 Mental health providers (school psychologist, MFT). To explore the leadership structure within school-based services for ASD and the effect on implementation processes, a survey including the Implementation Climate Scale (ICS) [4] and the Implementation Leadership Scale (ILS) [5] was distributed to participants.

**Results**

Implementation climate and implementation leadership varied by profession. For the Selection for Openness domain on the ICS, Mental health providers (B=-2.28, p=.022), Mid-level specialists (B=-2.09, p=.020), and Teachers/DSP (B=-2.55, p=.004) all reported lower ratings than High-level administrators. For the Educational Supports domain on the ICS, Mental health providers (B=-2.08, p=.035), Teachers/DSP (B=-2.16, p=.010), and School-site principals/administrators (B=-2.70, p=.029) all reported lower ratings than High-level administrators, while Teachers/DSP also reported lower ratings than Mid-level specialists (B=-.99, p=.018). For the Proactive Leadership domain on the ILS, Mental health providers (B=-2.39, p=.003), Mid-level specialists (B=-2.12, p=.002), and Teachers/DSP (B=-1.64, p=.016) all reported lower ratings than High-level administrators.

**Conclusion**

Implementation leadership and implementation climate vary across participants suggesting variability more broadly. Implications for implementation and sustainment of EBPs in school-based services will be discussed.

**References**

1. Annual Disability Statistics Compendium. Special education-students ages 14-21 served under IDEA, Part B, left school, by reason. 2014. http://disabilitycompendium.org/compendium-statistics/special-education.

2. Spillane JP, Healey K. Conceptualizing school leadership and management from a distributed perspective: An exploration of some study operations and measures. Elem School J. 2010;111(2):253-81.

3. Spillane J. Distributed Leadership. San Francisco, CA: Jossey-Bass; 2006.

4. Ehrhart MG, Aarons GA, Farahnak LR. Assessing the organizational context for EBP implementation: The development and validity testing of the Implementation Climate Scale (ICS). Implement Sci. 2014;9(1). doi:10.1186/s13012-014-0157-15.

5. Aarons GA, Ehrhart MG, Farahnak LR. The implementation leadership scale (ILS): Development of a brief measure of unit level implementation leadership. Implement Sci. 2014;9(1). doi:10.1186/1748-5908-9-45.

## A102 A survey of supervisors’ and managers’ practices and needs to support evidence-based practice implementation in large behavioral health care system in New York state

### Sapana Patel^1,2^, Andrea Cole^2^, Paul Margolies^1,2^, Nancy Covell^1,2^, Amy Anderson-Winchell^3^, Lisa Dixon^1,2^

#### ^1^Columbia University, New York, NY, USA; ^2^The New York State Psychiatric Institute, New York, NY, USA; ^3^Access Supports for Living, Middletown, NY, USA

##### **Correspondence:** Sapana Patel (sapana.patel@nyspi.columbia.edu)

**Background**

Managers and supervisors are the lynchpin of success to practice change within any organization. In a time when behavioral health organizations are being asked to shift culture, practice and delivery of care from volume to value, manager and supervisor roles are moving towards ensuring quality of evidence-based practice (EBP) implementation. We developed a survey to assess supervisor and manager experience with and needed supports for EBP implementation in community mental health agencies.

**Materials and Methods**

We created surveys using the implementation science literature [1-3] and guidance provided by the Center for Practice Innovations [4] provider advisory committee. In March 2019, behavioral health agency leadership (N=4) across New York State invited their managers and supervisors to take part in the survey. Both surveys queried level of commitment and preparedness (scored on a Likert scale, e.g., 1 = not at all committed/prepared to 5 = very committed/prepared) to implement EBPs along with needed tools and supports to implement EPBs to fidelity. Surveys also included questions about EBP topics for additional training.

**Results**

Data collection will finish in June 2019. Of the 23 survey respondents (supervisors: n of 13; managers n of 10) half are social workers (n=12). Supervisors report that, although they are highly committed to EBP implementation (M = 4.42, SD = 1.11), they are only moderately prepared to implement EBPs (M = 3.92, SD = 1.11). Needed resources included example case conceptualizations, and workbooks to use with consumers. Managers’ reports were similar: highly committed to EBP implementation (M = 4.80, SD = .40) and moderately prepared to implement EBPs (M = 4.10, SD = .83). Needed resources include worksheets for role modeling and train the trainers to support EBPs. Managers and supervisors identified trauma informed care and shared decision making as EBP topics that they need more training in.

**Conclusions**

Managers and supervisors’ commitment to EBP implementation is necessary, but far from sufficient. Managers and supervisors report feeling only moderately prepared to implement EBP and would benefit from training focusing on their roles in implementation, as well as tools and resources designed to help them.

**References**

1. Birken SA, DiMartino LD, Kirk MA, Shoou-Yih DL, McClelland M, Albert NM. Elaborating on theory with middle managers’ experience implementing healthcare innovations in practice. Implement Sci. 2016; 11(1):2.

2.Dorsey S, Pullman MD, Kerns SEU, Jungbluth N, Mesa R, Thompson K, Berliner L. The juggling act of supervision in community mental: Implications for supporting evidence-based treatment. Adm Policy Ment Health. 2017;44:838-852.

3. Dorsey S, Kerns SEU, Lucid L, Pullman MD, Harrison JP, Berliner L, Thompson K, Deblinger E. Objective coding of content and techniques in workplace-based supervision of an EBT in public mental health. Implement Sci. 2018;13:19.

4. Covell NH, Margolies PJ, Myers RW, Ruderman D, Fazio ML, McNabb LM, Gurran S, Thorning H, Watkins L, Dixon LB. Scaling up evidence-based behavioral health care practices in New York state. Psychiatr Serv. 2014;65(6):713-5. doi: 10.1176/appi.ps.201400071.

## A103 Examining therapist characteristics as moderators of change in ASD knowledge and confidence in a hybrid effectiveness/implementation trial

### Kassandra Martinez^1^, Eliana Hurwich-Reiss^2^, Lauren Brookman-Frazee^2,3^

#### ^1^SDSU/UCSD Joint Doctoral Program in Clinical Psychology, San Diego, CA, USA; ^2^Child and Adolescent Services Research Center, San Diego, CA, USA; ^3^University of California, San Diego, La Jolla, CA, USA

##### **Correspondence:** Kassandra Martinez (kamartin@ucsd.edu)

**Background**

Provider attitudes, including knowledge-of and confidence using evidence-based interventions (EBIs), are important outcomes of training interventions and potential mechanisms of EBI delivery. This study utilized data from a Hybrid Type-1 effectiveness trial of AIM-HI (An Individualized Mental Health Intervention for Children with ASD), an intervention to reduce challenging behaviors in children with Autism Spectrum Disorder (ASD). The following objectives were addressed: 1) examine the effectiveness of AIM-HI training/consultation on changes in therapists’ perceived knowledge and confidence (K&C) of ASD strategies, and 2) examine therapist demographic and professional characteristics as moderators of training effects.

**Materials and Methods**

Data were extracted from a cluster randomized trial. Therapist/client dyads were randomized to AIM-HI or usual care. AIM-HI therapists received training/consultation for 6 months. Therapists (N=156) reported K&C at baseline and 6 months. Three K&C subscales were used in analyses: ASD Knowledge (ASD), (2) Knowledge of EBI ASD strategies (KNOW), and (3) Confidence in EBI ASD strategies (CONF). Repeated-measures ANOVAs were used in analyses*.

**Results**

A main effect of AIM-HI training was found for all K&C constructs; AIM-HI therapists reported greater increases compared to usual care therapists (p<.001). For AIM-HI, therapist role (Staff vs. Trainee) moderated changes in ASD (F(1,154)=4.72, p<.05), KNOW (F(1,154)=11.19, p<.01), and CONF (F(1,154)=6.60, p=.01); trainees reported greater increases than staff. Therapists’ perceived ASD expertise moderated changes in ASD (F(1,154)=11.00, p<.001) and KNOW (F(1,154)=16.29, p< .001); therapists who did not consider themselves ASD specialists reported greater increases than their counterparts. Ethnicity moderated changes in CONF (F(1,154)=7.73, p<.05); minority therapists made more gains compared to White therapists. For usual care therapists, role moderated changes in ASD K&C (F(1,154)=7.4, p<.05); staff therapists reported increases in ASD knowledge, while trainees reported decreases.

**Conclusions**

Therapists who received AIM-HI training reported greater changes in K&C compared to those in usual care. Additionally, for those in AIM-HI, therapist’s cultural (ethnicity) and professional (role and ASD expertise) characteristics moderated the effect of training on K&C. Next steps include combining results with qualitative data to inform adaptations to improve intervention fit and maximize outcomes.

*Results from multiple level modeling accounting for the nested structure of the data will be reported.

**Trial Registration:** Clinicaltrials.gov NCT02416323

## A104 How do you apply implementation science in practice? core competencies for implementation practitioners

### Julia Moore^1^, Diana Kaan^2^, Louise Zitzelsberger^2^, Sobia Khan^1^

#### ^1^The Center for Implementation, Toronto, Ontario, Canada; ^2^Health Canada, Ottawa, Ontario, Canada

##### **Correspondence:** Julia Moore (julia.moore@thecenterforimplementation.com)

**Background**

The field of implementation science has advanced in recent years, but unfortunately this has coincided with a growing divide between the science and practice of implementation. One strategy to bridge this gap is training implementation practitioners to apply implementation science to their initiatives in a thoughtful and proactive way. Effective implementation capacity building should be based on core competencies - the knowledge, skills, attitudes, and behaviors needed to apply implementation science. There is a growing body of literature on core competencies for implementation scientists, but same progress has not been made for core competencies for implementation practitioners. Building applied implementation science capacity at the practitioner level can foster better implementation and overall improved population-level impacts; therefore, understanding the core competencies for applying implementation science at the front line is paramount. The goal of this project was to extrapolate and synthesize core competencies for implementation practitioners.

**Materials and Methods**

We scanned the published and gray literature to identify core competencies for implementation practice. Six documents outlining (or including components of) core competencies for implementation practice were retrieved. Two analysts reviewed each document using a content analysis approach. Competencies relevant to implementation practice were extracted into an abstraction form and consolidated into a list of common competencies. The refined list of competencies was then grouped thematically into overarching implementation “activities” (e.g., understanding the problem, facilitating implementation).

**Results**

We identified 40 core competencies which we categorized into 10 implementation activities: Inspiring Stakeholders and Developing Relationships; Building Implementation Teams; Understanding the Problem; Using Evidence to Inform all Aspects of KT; Assessing the Context; Facilitating Implementation; Evaluation; Planning for Sustainability; Brokering Knowledge; and Disseminating Evidence. Additionally, we identified 5 values or guiding principles for implementation practice, which emerged from the document review. We are building an Implementation Practice Core Competency Tool which will be finalized by September.

**Conclusions**

This presentation will briefly highlight the methods and then focus on how to prioritize and select relevant core competencies for projects and individuals. The competencies can be used as a guide to prioritize capacity building efforts.

## A105 Implementation practitioners: what knowledge, skills and abilities do they need?

### Jenna McWilliam^1^, Jacquie Brown^2^

#### ^1^Triple P International, Brisbane, Australia; ^2^Families Foundation, Hilversum, The Netherlands

##### **Correspondence:** Jenna McWilliam (jenna@triplep.net)

**Background**

The increased influence of implementation science has prompted an important question: if an organisation does not have personnel who are fluent in implementation science how do they apply it? Often an implementation expert, intermediary organisation or consultant is engaged. But who are they and what knowledge, skills and abilities must they have? There is limited literature on the core competencies of implementation practitioners including a lack of common terminology and title. However, there is emerging literature on the role, knowledge, skills and abilities that contribute to effective consultation for implementation [1-2]. This presentation draws on this literature, our experience developing the implementation support capabilities of a purveyor/intermediary organisation (Triple P International) [3] learnings from Triple P International Implementation Consultants (TPI-ICs), who for over five years have supported organisations in the application of implementation science and with implementing organisations and practitioners. The presentation will use data from experience and the competencies and coaching literature to promote discussion about required competencies for implementation practitioners.

**Materials and Methods**

A review was undertaken to examine the role and responsibilities of the TPI-ICs. This included a review of existing literature; a survey of TPI-ICs (n=27) and a review of internal support systems and processes.

**Results**

The following areas were identified as areas of significance for competencies: Partnering with implementing organisations; determining fit; establishing relationships and roles; facilitating implementation planning; monitoring and evaluating; establishing the innovation as usual practice. Results from the survey identified self-reported levels of confidence and competence in knowledge, skills and abilities related to implementation consultation. The review of existing systems and process identified areas for improvement and increased structure to support desired outcomes. Results were then used to inform the development of TPI-IC Competencies, and a comprehensive IC Management and Support Process.

**Conclusions**

The presentation will describe knowledge-base, processes and characteristics for IC Competencies, IC Management and Support Process. It aims to promote discussion on future areas of practice development as well as exploring ways that additional research could help advance our understanding and further develop the field.

**References**

1. Metz A, Louison L, Ward C, Burke K. National Implementation Research Network. Global Implementation Specialist Practice Profile: Skills and Competencies for Implementation Practitioners. 2017. https://www.effectiveservices.org/downloads/Implementation_specialist_practice_profile.pdf. Accessed 10 August 2017.

2. Proctor EK, Landsverk J, Baumann AA, Mittman BS, Aarons GA, Brownson RC, Glisson C, Chambers D. The implementation research institute: training mental health implementation researchers in the United States. Implement Sci. 2013;8(1):105. doi:10.1186/1748-5908-8-105.

3. McWilliam J, Brown J, Sanders MR, Jones L. The Triple P implementation framework: the role of purveyors in the implementation and sustainability of evidence-based programs. Prev Sci. 2016;17(5):636-645. doi:10.1007/s11121-016-0661-4.

## A106 Barriers to implementation of evidence-based treatments for posttraumatic stress disorder at 12-months post training

### Mariya Zaturenskaya^1^, Sebastian Bliss^1^, Katherine Dondanville^2^, Brooke Fina^1^, Vanessa Jacoby^1^, Jeremy Karp^1^, Arthur Marsden^1^

#### ^1^University of Texas Health Science Center at San Antonio, San Antonio, TX, USA; ^2^UT Health San Antonio, San Antonio, TX, USA

##### **Correspondence:** Mariya Zaturenskaya (zaturenskaya@uthscsa.edu)

**Background**

Despite rigorous efforts to disseminate evidence-based treatments (EBTs) for posttraumatic stress disorder (PTSD) in community settings and major medical systems such as the Veterans Health Administration, penetration rates for these treatments have been suboptimal [1-2]. Understanding of provider-related and client-related barriers and challenges to EBT implementation is crucial to improving EBT reach with community mental health settings. This is a naturalistic study of perceived implementation barriers and challenges for EBTs for PTSD in a sample of community providers who were trained in cognitive processing therapy or prolonged exposure for PTSD by the STRONG STAR Training Initiative (SSTI), a comprehensive competency-based training program designed to disseminate EBTs.

**Materials and Methods**

To date, 42 community-based mental health providers completed a survey 12 months after completing an in-person SSTI training workshop. The follow-up survey assessed the barriers to initiating evidence-based treatments as well as the challenges with implementing EBTs with PTSD clients.

**Results**

At 12-month post-training, 64% of providers endorsed at least one client-related barrier, while 19% reported at least one therapist-related barrier to initiating an EBT with clients diagnosed with PTSD. The most common barrier to initiating treatment was client’s declining the use of an EBT (57% of providers). The barrier endorsed the least was discomfort introducing EBTs (0%). When looking at challenges to implementing EBT for PTSD, 83% of providers reported at least one challenge, with 55% of providers reporting 1-2 challenges, and 29% reporting 3 or more. The most common challenges to implementing an EBT included: client disinterest in engaging in EBT (50%), a difficulty obtaining an appropriate referral (45%), a lack of clients with PTSD on caseload (31%), and a difficulty taking time away from regular work to attend consultation call (14%). The challenge endorsed the least was the lack of motivation to use a new treatment (2%).

**Conclusions**

The results of this study suggest that the majority of community providers report experiencing challenges with both, EBT initiation and implementation. Understanding barriers to initiation and implementation can guide trainers and organizations in addressing provider concerns thereby improving dissemination and implementation efforts.

**References**

1. Marques L, Dixon L, Valentine SE, Borba CPC, Simon NM, Stirman SW. Providers’ perspectives of factors influencing implementation of evidence-based treatments in a community mental health setting: a qualitative investigation of the training—practice gap. Psychol Serv. 2016;(3):322-331

2. Rosen CS, Matthieu MM, Stirman SW, Cook JM, Landes S, Bernardy NC, Chard KM, Crowley J, Eftekhari A, Finley EP, Hamblen JL, Harik JM, Kehle-Forbes SM, Meis LA, Osei-Bonsu PE, Rodriguez AL, Ruggiero KJ, Ruzek JI, Smith BN, Trent L, Watts BV. A review of studies on the system-wide implementation of evidence-based psychotherapies for posttraumatic stress disorder in the Veterans Health Administration. Adm Policy Ment Health. 2016;43(6):957-977.

## A107 Training future mental health professionals in managing and adapting practice, an evidence-informed system of care

### Julia Cox^1^, Michael Southam-Gerow^2^

#### ^1^University of California, Los Angeles, Los Angeles, CA, USA; ^2^Virginia Commonwealth University, Richmond, VA, USA

##### **Correspondence:** Julia Cox (coxjr4@vcu.edu)

**Background**

High quality mental health services do not reach the youth who need them, leading to efforts to implement effective treatments more broadly. One focus of these efforts concerns training the mental health workforce, of which masters-level social workers represent a large proportion. However, the curricula of master’s in social work (MSW) programs do not often emphasize evidence-based approaches. One possible solution is Managing and Adapting Practice (MAP; PracticeWise, LLC), a system that allows clinicians to (1) identify clinically indicated evidence-based programs by searching a growing evidence-base of randomized controlled trials (RCTs) and (2) build individualized evidence-informed treatment plans by focusing on common practice elements. MAP may also address the concerns about manual-based programs (e.g., inflexibility). Although some MSW programs have integrated MAP, the benefits of MAP training within MSW education have not yet been evaluated. This project evaluated multiple mechanisms of training [1] in a semester-long MSW-focused MAP course relative to curriculum-as-usual control at a large public university.

**Materials and Methods**

Participants were advanced MSW students (mean age = 27, SD = 5.8; 92.3% women; 59% white) either enrolled in the MAP course (n = 17) or enrolled in curriculum-as-usual (n = 22). The MAP course was co-taught by an expert MAP trainer and a MAP-trained social worker. Pre- and post-semester, participants completed a battery that included: (1) role-plays with standardized patients that were videotaped and coded using the Therapy Observational Coding System of Child Psychotherapy – Revised Strategies scale [2]; (2) a written task that was subsequently coded to assess participants’ clinical decision-making skills during different phases of a standardized case; and (3) attitudinal factors that may be predictive of future MAP usage, such as attitudes toward evidence-based practice [3] and the acceptability and feasibility of MAP [4].

**Results**

Results indicate significant uptake of cognitive and behavioral therapeutic strategies in the MAP condition. Overall, participants endorsed positive attitudes toward evidence-based practice broadly and MAP specifically.

**Conclusions**

Findings may be used to inform the development of more effective evidence-informed curriculum for masters-level clinical programs and future workforce training initiatives. Methodological considerations may inform advances in instrumentation to measure multidimensional training outcomes.

**References**

1. McLeod BD, Cox JR, Jensen-Doss A, Herschell A, Ehrenreich-May J, Wood JJ. Proposing a mechanistic model of training and consultation. Clin Psychol Sci Pract. 2018;25. doi:10.1111/cpsp.12260

2. McLeod BD, Smith MM, Southam-Gerow MA, Weisz JR, Kendall PC. Measuring treatment differentiation for implementation research: the Therapy Process Observational Coding System for Child Psychotherapy Revised Strategies scale. Psychol Assess. 2015;27(1):314-25. doi:10.1037/pas0000037

3. Aarons GA. Mental health provider attitudes toward adoption of evidence-based practice: the Evidence-Based Practice Attitude Scale (EBPAS). Ment Health Serv Res, 2004;6(2):61-74.

4. Chafouleas SM, Briesch AM, Neugebauer SR, Riley-Tillman TC. Usage rating profile – intervention (revised). Storrs, CT: University of Connecticut; 2011.

## A108 Lessons learned: a data-driven approach to supervision and training of stakeholders

### Stephanie Moore, Laura Clary, Kimberly Arnold, Steven Sheridan, Tamar Mendelson

#### Johns Hopkins University, Baltimore, MD, USA

##### **Correspondence:** Stephanie Moore (smoore99@jhmi.edu)

**Background**

Few evidence-based practices (EBPs) are successfully installed into school settings [1]. Integrating implementation considerations into early stages of intervention evaluation and collaborating with relevant stakeholders are recommended to reduce the gap between EBP evaluation, adoption, implementation, and sustainability [2-4]. As part of a school-based prevention trial, school stakeholders participated in intervention training and implementation with a goal of sustaining the intervention at the schools after the study’s conclusion. This presentation explicates lessons learned via the study of stakeholder involvement, which in-turn informed subsequent implementation and evaluation efforts.

**Materials and Methods**

Over three years, 20 urban public schools were recruited for a randomized trial assessing two wellness programs, one targeting eighth graders’ emotion regulation and decision-making (RAP Club) and the other health education (Healthy Topics). At each school, middle-school teachers and school-based mental health providers were recruited as intervention “co-facilitators in training.” One school mental health provider per school received training in RAP Club, and one middle school teacher per school received training in Healthy Topics (N = 40 across all schools). Stakeholder attendance and engagement during intervention training, supervision calls, and intervention sessions were recorded. School characteristics (e.g., organizational health), stakeholder interviews, and process notes further informed our investigation of stakeholder engagement in training and implementation.

**Results**

Most stakeholders attended intervention training; however, attendance on supervision calls was limited. Two-thirds of school personnel were regularly present during intervention sessions, but fewer than half actively participated. Stakeholder participation and engagement increased each year of the trial. Individual- and school-level factors were related to participation in training, supervision, and implementation. Findings will be used to refine personnel training and supervision (e.g., augmenting structure, explicating goals) for the final year of trial implementation.

**Conclusion**

Evaluating stakeholder involvement in intervention training and implementation has been critical in informing this research team’s approach to stakeholder training and supervision to support implementation and sustainability. Our findings illustrate both challenges and opportunities for increased stakeholder involvement in implementation. Factors influencing stakeholder participation, assessed during controlled trials, can be leveraged to inform subsequent evaluations, as well as program adoption, implementation, and sustainability.

**Trial registration:** Clinicaltrials.gov NCT03906682

**References**

1. Lyon AR. Implementation Science and Practice in the Education Sector. 2017. https://education.uw.edu/sites/default/files/Implementation Science Issue Brief 072617.pdf. Accessed 20 March 2019.

2. Curran GM, Bauer M, Mittman B, Pyne JM, Stetler C. Effectiveness-implementation hybrid designs. Med Care. 2012;50:217–26.

3. Goldstein H, Olswang L. Is there a science to facilitate implementation of evidence-based practices and programs? Evid Based Commun Assess Interv. 2017;11:55–60.

4. Beidas RS, Stewart RE, Adams DR, Fernandez T, Lustbader S, Powell BJ, Aarons GA, Hoagwood KE, Evans AC, Hurford MO, Rubin R, Hadley T, Mandell DS, Barg FK. A multi-level examination of stakeholder perspectives of implementation of evidence-based practices in a large urban publicly-funded mental health system. Adm Policy Ment Health. 2016;43:893–908.

## A109 Who takes advantage of training initiatives: are we just preaching to the choir and whistling in the wind?

### Brigid Marriott, Jack Andrews, Kristin Hawley

#### University of Missouri, Columbia, MO, USA

##### **Correspondence:** Brigid Marriott (bmvv5@mail.missouri.edu)

**Background**

Numerous implementation initiatives have endeavored to bridge the research-to-practice gap [1-2]. However, the reach of these implementation initiatives has rarely been studied. In the current study, we describe a county-wide youth mental health (MH) initiative supported by a voter-approved sales tax. This initiative aims to improve access to effective youth MH services by providing free training, consultation, and support in evidence-based practices (EBPs) to MH service providers. The current study has three aims: 1) describe the providers reached by the initiative, 2) examine which training activities providers engage in (i.e., formal workshops; learning collaboratives; individual consultation), and 3) explore differences in providers (e.g., discipline; attitudes; knowledge) who do and do not invest in training activities.

**Materials and Methods**

Participants (N = 523) were community MH providers who completed a web-based baseline assessment prior to registering for the EBP trainings. Measures included demographics, clinical practice information, self-reported confidence, organizational climate [3], and EBP knowledge [4], attitudes [5], and practice [6].

**Results**

The initiative reached over 500 providers who were part of over 100 different organizations and private practices. Registered providers were predominantly master’s level (N = 277, 53.06%), representing social work (N=178, 34.10%), counseling (N=140, 26.82%), psychology (N=61, 11.69%), and other MH disciplines (N=143, 27.34%). Some 159 (34.40%) were fully licensed MH providers, 69 (13.19%) post-degree but unlicensed, 119 (22.75%) student trainees, and 176 (33.65%) none or other types of licensure (e.g., RN, MD). Providers on average had been providing MH services for 8.13 years (SD = 8.52, range = 0 to 45). Registered providers participated most frequently in formal workshops (69.79%, N=365,) and less often in small group learning collaboratives (7.07%, N=37) and individual consultation (6.88%, N=36). Initial findings showed significant, positive associations between baseline EBP attitudes (r=.11, p=.01) and knowledge (r=.13, p<.01) and the number of formal workshops attended.

**Conclusions**

The initiative reached a high proportion of MH providers and organizations; however, far fewer actually participated in any training activities. The more in-depth, personal training and support components were the least utilized. Implications for voluntary implementation initiatives within community MH care will be discussed.

**References**

1. Dorsey S, Berliner L, Lyon AR, Pullmann MD, Murray LK. A statewide common elements initiative for children’s mental health. J Behav Health Serv Res. 2016;43(2):246–261.

2. Jensen-Doss A, Hawley KM, Lopez M, Osterberg LD. Using evidence-based treatments: The experiences of youth providers working under a mandate. Prof Psychol Res Pract. 2009;40(4):417.

3. Ehrhart MG, Aarons GA, Farahnak LR. Assessing the organizational context for EBP implementation: The development and validity testing of the Implementation Climate Scale (ICS). Implement Sci. 2014;9(1):157.

4. Stumpf RE, Higa-McMillan CK, Chorpita BF. Implementation of evidence-based services for youth: Assessing provider knowledge. Behav Modif. 2009;33(1):48–65.

5. Aarons GA, Cafri G, Lugo L, Sawitzky A. Expanding the domains of attitudes towards evidence-based practice: The evidence based practice attitude scale-50. Adm Policy Ment Health. 2012;39(5):331–340.

6. Cho E, Wood PK, Taylor EK, Hausman EM, Andrews JH, Hawley KM. Evidence-based treatment strategies in youth mental health services: Results from a national survey of providers. Adm Policy Ment Health. 2019;46(1):71–81.

## A110 Expanding hybrid designs for implementation research: intervention, implementation strategy, and context

### Christopher Kemp^1^, Bradley Wagenaar^1^, Emily Haroz^2^

#### ^1^University of Washington, Seattle, WA, USA; ^2^Johns Hopkins University, Baltimore, MD, USA

##### **Correspondence:** Christopher Kemp (kempc@uw.edu)

**Background**

Successful implementation reflects the interplay between intervention, implementation strategy, and context [1]. Hybrid effectiveness-implementation studies allow investigators to assess intervention effects on patient health alongside implementation strategy effects on implementation outcomes [2-4], though the role of context as a third independent variable (IV) is incompletely specified.

**Materials and Methods**

Our objective is to expand the hybrid effectiveness-implementation framework to include mixtures of all three types of IVs: intervention, implementation strategy, and context. We propose to use I to represent the IV of intervention, IS to represent implementation strategy, and C to represent context.

**Results**

The expanded framework specifies nine two-variable hybrid designs: I/is, I/IS, IS/i, IS/c, IS/C, C/is, C/i, I/C, and I/c. We describe four in detail: I/is, IS/c, IS/C, and C/is. We also specify seven three-variable hybrid designs that follow from the two-variable designs. We argue that many studies already meet our definition of two- or three-variable hybrids.

**Conclusions**

Our proposal builds naturally from the typology proposed by Curran et al. [2], but offers a more complete and clear specification of designs that might be of interest to implementation researchers. We need studies that are designed and powered to measure the implementation-related effects of variations in contextual determinants, both to advance the science and to optimize delivery of interventions in the real world. Prototypical implementation studies that evaluate the effectiveness of an implementation strategy, in isolation from its context, risk perpetuating the persistent gap between evidence and practice, as they will not generate essential context-specific knowledge around implementation, scale-up, and de-implementation.

**References**

1. Pfadenhauer LM, Gerhardus A, Mozygemba K, Lysdahl KB, Booth A, Hofmann B, Wahlster P, Polus S, Burns J, Brereton L. Making sense of complexity in context and implementation: The Context and Implementation of Complex Interventions (CICI) framework. Implement Sci. 2017;12(1):21.

2. Curran GM, Bauer M, Mittman B, Pyne JM, Stetler C: Effectiveness-implementation hybrid designs: Combining elements of clinical effectiveness and implementation research to enhance public health impact. Med Care. 2012; 50(3):217.

3. Proctor E, Silmere H, Raghavan R, Hovmand P, Aarons G, Bunger A, Griffey R, Hensley M. Outcomes for implementation research: conceptual distinctions, measurement challenges, and research agenda. Adm Policy Ment Health. 2011;38(2):65-76.

4. Powell BJ, Waltz TJ, Chinman MJ, Damschroder LJ, Smith JL, Matthieu MM, Proctor EK, Kirchner JE. A refined compilation of implementation strategies: Results from the Expert Recommendations for Implementing Change (ERIC) project. Implement Sci. 2015;10(1):21.

## A111 The use of the PARIHS framework in implementation research and practice – a citation analysis of the literature

### Anna Bergström^1^, Anna Ehrenberg^2^, Ann Catrine Eldh^3^, Ian Graham^4^, Kazuko Gustafsson^1^, Gillian Harvey^5^, Alison Kitson^6^, Jo Rycroft-Malone^7^, Lars Wallin^2^

#### ^1^Uppsala University, Uppsala, Sweden; ^2^Dalarna University, Falun, Sweden; ^3^Linköping University, Linköping, Sweden; ^4^University of Ottawa, Ottawa, Ontario, Canada; ^5^University of Adelaide, Adelaide, Australia; ^6^Flinders University, Adelaide, Australia; ^7^Bangor University, Bangor, Wales, United Kingdom

##### **Correspondence:** Anna Bergström (anna.bergstrom@kbh.uu.se)

**Background**

The Promoting Action on Research Implementation in Health Services (PARIHS) framework was developed two decades ago and conceptualizes successful implementation (SI) as a function (f) of the evidence (E) nature and type, context (C) quality and the facilitation (F), [SI = f (E,C,F)] [1-4]. Despite a growing number of citations of theoretical frameworks including the PARIHS, details of how theoretical frameworks are used remains largely unknown. This review aimed to enhance the understanding of the breadth and depth of the use of the PARIHS framework.

**Materials and Methods**

This citation analysis departed from four core articles representing the key stages of the framework’s development. The citation search was performed in Web of Science and Scopus. After exclusion, we undertook an initial assessment aimed to identify articles using PARIHS and not only referencing any of the core articles. To assess this, all articles were read in full. Further data extraction included capturing information about where (country/countries and setting/s) PARIHS had been used, as well as categorizing how the framework was applied. Also, strengths and weaknesses, as well as efforts to validate the framework, were explored in detail.

**Results**

The citation search yielded 1,163 articles. After applying exclusion criteria, 1,059 articles were read in full, and the initial assessment yielded a total of 259 articles reported to have used the PARIHS framework. These articles were included for data extraction. The framework had been used in a variety of settings and in both high-, middle- and low-income countries. With regards to types of use, 28% used the PARIHS in planning and delivering an intervention, 49% in data analysis, 55% in the evaluation of study findings, and/or 46% in any other way. Further analysis showed that its actual application was frequently partial, and generally not well elaborated.

**Conclusions**

In line with previous citation analysis of the use of theoretical frameworks in implementation science we found a rather superficial description also of the use of the PARIHS. Thus, we propose the development and adoption of reporting guidelines on how framework(s) are used in implementation studies, with the expectation that it enhances the maturity of implementation science.

**References**

1. Kitson A, Harvey G, McCormack B. Enabling the implementation of evidence-based practice: a conceptual framework. Qual Health Care. 1998;7(3):149-58.

2. Rycroft-Malone J, Kitson A, Harvey G, McCormack B, Seers K, Titchen A, Estabrooks C. Ingredients for change: revisiting a conceptual framework. BMJ Quality Saf. 2002;11(2):174-80.

3. Rycroft-Malone J, Harvey G, Seers K, Kitson A, McCormack B, Titchen A. An exploration of the factors that influence the implementation of evidence into practice. J Clin Nurs. 2004;13(8):913-24.

4. Kitson AL, Rycroft-Malone J, Harvey G, McCormack B, Seers K, Titchen A. Evaluating the successful implementation of evidence into practice using the PARiHS framework: theoretical and practical challenges. Implement Sci. 2008;3:1.

## A112 How are health policy implementation outcomes measured quantitatively? a review protocol

### Peg Allen^1^, Cole Hooley^1^, Meagan R. Pilar^1^, Cara C. Lewis^2^, Kayne D. Mettert^2^, Caitlin N. Dorsey^2^, Jonathan Purtle^3^, Stephanie Mazzucca^1^, Alexandra B. Morshed^1^, Ana Baumann^1^, Maura M. Kepper^1^, Ross C. Brownson^1^

#### ^1^Brown School, Washington University in St. Louis, St. Louis, MO, USA; ^2^Kaiser Permanente Washington Health Research Institute, Seattle, WA, USA; ^3^Drexel University, Philadelphia, PA, USA

##### **Correspondence:** Peg Allen (pegallen@wustl.edu)

**Background**

Evidence about effective strategies in clinical care and population health is growing, with a number of evidence-based policy approaches now recommended in the Community Guide [1] and other systematic reviews. But understanding lags on how best to implement recommended policies to reap the full population health benefits. Information is limited on how to quantitatively measure policy implementation outcomes [2]. To address this gap a systematic review has begun to identify and rate quantitative measures of health policy implementation outcomes and predictors.

**Materials and Methods**

We are reviewing published academic journal articles to identify the state of quantitative measurement of health policy implementation. To guide the systematic measures review, we combined two frameworks impacting policy implementation: 1) for internal context, the Consolidated Framework for Implementation Research; [3] and 2) for external context, Bullock’s policy implementation determinants framework (under review).

We are applying Lewis et al.’s measures review protocol and PAPERS rating system [4]. We searched these databases: CINAHL Plus, Medline, PsychInfo, PAIS, ERIC, and Worldwide Political. The four search strings included multiple search terms for: health, public policy, implementation, and measurement. We will code measures to implementation outcomes [5] and predictors. Inclusion criteria: 1) empirical study of the implementation of public policies already passed or approved addressing physical or behavioral health; 2) quantitative self-report or archival measures utilized; 3) peer-reviewed journal publication 1995 through April 2019; and 4) English language text.

**Results**

We will screen abstracts April-June 2019. In July-August 2019 we will review and extract full texts. We will present yields, characteristics of included articles, and description of several identified quantitative policy implementation outcome measures. We will show plans for fall/winter 2019 pragmatic measure rating, summarization, and web-based posting. We seek feedback from policy implementers and researchers on remaining procedures. We especially want feedback on design of a publicly available web-based summary of identified measures and pragmatic properties to ensure usefulness to policy implementers and researchers. We are collaborating with SIRC on methodology and dissemination.

**Conclusions**

The measures summary is intended to stimulate further assessment of health policy implementation outcomes and predictors to help practitioners and researchers spread evidence-informed policies to improve population health.

**References**

1. The Community Guide. Guide to community preventive services. 2019. https://www. thecommunityguide.org. Accessed 25 March 2019.

2. Watson DP, Adams EL, Shue S, Coates H, McGuire A, Chesher J, Jackson J, Omenka OI. Defining the external implementation context: an integrative systematic literature review. BMC Health Serv Res. 2018;18(1):209

3. Damschroder LJ, Aron DC, Keith RE, Kirsh SR, Alexander JA, Lowery JC. Fostering implementation of health services research findings into practice: a consolidated framework for advancing implementation science. Implement Sci. 2009;4:50.

4. Lewis CC, Mettert KD, Dorsey CN, Martinez RG, Weiner BJ, Nolen E, Stanick C, Halko H, Powell BJ. An updated protocol for a systematic review of implementation-related measures. Syst Rev. 2018;7(1):66.

5. Proctor E, Silmere H, Raghavan R, Hovmand P, Aarons G, Bunger A, Griffey R, Hensley M. Outcomes for implementation research: conceptual distinctions, measurement challenges, and research agenda. Adm Policy Ment Health. 2011; 38(2):65-76.

## A113 Development and evaluation of an instrument to measure fidelity to implementation of collaborative care in primary care clinics

### Erin LePoire, Anna Ratzliff, Diane Powers, Deborah J. Bowen

#### University of Washington, Seattle, WA, USA

##### **Correspondence:** Erin LePoire (lepoire2@uw.edu)

**Background**

Collaborative Care (CoCM) has been shown to be an effective way to treat depression and other mental illness in primary care and other settings. Evaluating whether or not clinics that receive training and technical assistance to implement CoCM maintain fidelity to the core components proven in other research to predict better patient outcomes has received little attention. A scalable measure clinics can use to measure fidelity is needed. This analysis discusses the creation and evaluation of an instrument specifically designed to measure multiple domains of CoCM fidelity.

**Materials and Methods**

Development of the CoCM fidelity tool occurred in three steps. Step one was development of a rubric that was utilized during in-person site visits with a group of HRSA-funded clinics. In Step two the rubric domains were adapted based on the outcomes of these site visits and incorporated into a qualitative interview guide administered to care managers trained in CoCM within the preceding six months to assess fidelity. Step three focused on converting the rubric to a self-administered format so it can be used by organizations to self-assess fidelity to core CoCM components.

**Results**

In Step one, clinics had an average rubric score of 3.09 “core features Implemented” (range 2.16-4.59) out of a possible 5.0 which indicates “exceptional Implementation.” Step two transcript data from qualitative interviews revealed that care managers are able to link their clinic’s current CoCM processes to core CoCM concepts in the fidelity rubric. Findings from development of the rubric and qualitative interviews will be presented. The Step three self-administered rubric will be tested in 24 clinics across the United States participating in a CoCM implementation. We will compare this data to clinic outcomes in order to determine validity.

**Conclusions**

A self-administered instrument to assess CoCM fidelity in primary care clinics is feasible and further evaluation will allow us to connect use of the rubric to patient-level clinical outcomes and provider-level outcomes among all members of the CoCM team (care manager, primary care provider, psychiatric consultant).

## A114 Measurement of implementation strategies for pharmacy benefits management MUET initiatives to optimize medication management

### Anju Sahay^1^, Francesca Cunningham^2^, Peter Glassman^2^, Von Moore^2^, Muriel Burk^2^, Parisa Gholami^1^, Shoutzu Lin^1^, Brian Mittman^3^, Paul Heidenreich^1,4^

#### ^1^MedSafe QUERI Program, Palo Alto VA Health Care System, Palo Alto, CA, USA; ^2^VA Office of Pharmacy Benefits Management Services, Washington, DC, USA; ^3^Kaiser Permanente, CA, USA; ^4^Stanford University, Stanford, CA, USA

##### **Correspondence:** Anju Sahay (anju.sahay@va.gov)

**Background**

Effective implementation strategies are critical for understanding and improving outcomes. VA’s Office of Pharmacy Benefits Management Service (PBM) has a national Medication Use Evaluation Tracker (MUET) web application designed to provide close to real-time summary-level patient data (monthly to quarterly) to reduce potentially unsafe or unnecessary medication. Focusing on the developmental phase of formative evaluation, in the context of five current MUET initiatives, we collaborated with PBM to understand their use, and the value of seven pre-identified strategies to implement them: provider education, academic detailing, electronic reminders, patient specific care plan, draft orders, patient mailings and calling patients.

These five MUET initiatives were Dimethyl Fumarate (DMF), New Mineralocorticoid Receptor Antagonist (MRA), Prasugrel or Ticagrelor Treatment Duration >12 months (PRAT), Women of Childbearing Age on Warfarin (WoW) and Direct Oral Anticoagulants (DOAC).

**Materials and Methods**

In collaboration with PBM, in 2017 for the DMF initiative (n=143) and in 2018 for the remaining four initiatives (n=142) all VISN Pharmacy Executives (VPEs) emailed web-based surveys to a designated pharmacist at each of their facilities. Goal was to understand which among the seven strategies were being used by the facilities to implement the specific MUET initiative, and their perceived value. We also assessed barriers for facilities not using the implementation strategies. Response rates were as follows: 2017 (n=127, 89.0%) and 2018 (n=131, 93.2%).

**Results**

The most commonly used implementation strategies by pharmacists were provider education (26.6%), patient mailings (23.5%), using electronic reminders (21.2%) and entering draft orders (14.3%). Comparatively, pharmacists perceived provider education as being most useful (27.6%) along with having patient specific care plans (24.6%) and using electronic reminders (18.8%).

Pharmacists at facilities which did not implement one or more strategies reported barriers like time-consuming/not enough staff (43.6%), they didn’t believe this would work (22.7%), to implement they needed help from other services/departments (18.6%) and some pharmacists believed this was inappropriate work for a pharmacist (14.9%).

**Conclusions**

Pharmacists perceived provider education as the most useful strategy to monitor medication safety for their patients. Formative evaluation focuses on the identification and adoption of best practices to improve medication safety for the Veterans.

## A115 Defining and developing measures to assess public health program sustainability

### Sarah Moreland-Russell, Rebecca Vitale, Elizabeth Zofkie

#### Brown School, Washington University in St. Louis, St. Louis, MO, USA

##### **Correspondence:** Sarah Moreland-Russell (smoreland-russell@wustl.edu)

**Background**

Many recent Dissemination and Implementation Science studies have neglected to observe what happens to programs once they have been implemented. This has contributed to the lack of a cohesive and succinct definition of sustainability for public health programs. While certain studies define sustainability as the continuation of programmatic activities over time, others conceptualize sustainability as the continued delivery of benefits to target populations and the maintenance of collaborative structures within communities [1]. Ultimately, conflicts between these definitions disrupt the continuity of program sustainability research and focus. With public health funding in perpetual jeopardy, a cohesive, solidified definition of program sustainability has never been more necessary.

**Materials and Methods**

The study began with an extensive systematic literature review of program sustainability research, including empirical research, case studies, fieldwork, and commentaries. This process outlined various proposed definitions of sustainability and cataloged organizational metrics tied to sustainability outcomes. The target audience for the current study utilized evidence-based state tobacco control (TC) programs. Therefore, the second part of the methodology included consultations and interviews with key tobacco control specialists, sustainability experts, academics, and practice-oriented professionals. These interviews were then cross-referenced with the literature to find commonalities between theory and practice. Finally, the study team collected and analyzed federal progress reports submitted annually by these TC programs. The items outlined in these reports were referenced back to the previously identified sustainability metrics [2-3]. The organizational metrics described in the literature were aligned to the evidence-based Program Sustainability Framework. This framework defines the internal and external factors operationalized into eight domains that affect a program’s capacity for sustainability [4-5].

**Results**

Through this process, institutionalization emerged as the primary measure of sustainability found in both the literature and dialogue. To this effect, the establishment of a program through formal organizational rules and funding was widely perceived to ensure the delivery of continued program initiatives and benefits. The results further distinguish between programmatic, organizational, community-level factors, and funder support, suggesting sustainability planning must account for institutional scope.

**Conclusions**

This concise definition will enable valid, empirical comparisons of sustainability across programs in various public health contexts.

**References**

1. Scheirer MA. Is sustainability possible? A review and commentary on empirical studies of program sustainability. Am J Eval. 2005; 26:320-47.

2. Vitale R, Blaine T, Zofkie E, Moreland-Russell S, Combs T, Brownson R, Luke D. Developing an evidence-based program sustainability training curriculum: a group randomized, multi-phase approach. Implement Sci. 2018;13(1):126.

3. Savaya R, Spiro S. Predictors of sustainability of social programs. Am J Eval. 2012; 33(1):26-43

4. Luke D, Calhoun A, Robichaux C, Elliott M, Moreland-Russell S. The Program Sustainability Assessment Tool: A new instrument for public health programs. Prev Chronic Dis. 2014;11:130-184.

5. Schell S, Luke D, Schooley M, Elliott M, Mueller N, Bunger A. Public health program capacity for sustainability: a new framework. Implement Sci. 2013;8(15).

## A116 Pragmatic measurement of the quality of healthcare provider patient-centered behavior change counseling using the behavior change counseling index (BECCI)

### Doyanne Darnell, Kaylie Diteman, Dylan Fisher, Lea Parker, Allison Engstrom, Christopher Dunn

#### University of Washington, Seattle, WA, USA

##### **Correspondence:** Doyanne Darnell (darnelld@uw.edu)

**Background**

Training healthcare providers in Motivational Interviewing or similar patient-centered behavioral interventions is increasingly popular [1]; however, gold-standard methods to assess skill acquisition and ongoing quality assessment are laborious and impractical in busy healthcare settings. We examined the utility of a brief measure requiring modest training, the Behavior Change Counseling Index (BECCI) [2], to pragmatically capture provider skill in patient-centered alcohol counseling.

**Materials and Methods**

The present study includes a multidisciplinary sample of routine trauma center providers (N = 69) trained to counsel trauma patients about risky alcohol use as part of a 25-site National Institutes of Health-funded pragmatic trial of a collaborative care intervention [3]. Providers were predominantly White (79%) females (87%) with at minimum a bachelor’s degree. Providers completed a pre-training 20-minute standardized patient role-play in which they counseled a patient actor about alcohol use. At the end of the role-play, the standardized patient actor completed the brief (<5 minute) 12-item BECCI measure. Audio recordings of the role-plays were subsequently coded by an objective rater using the Motivational Interviewing Treatment Integrity Scale (MITI), a longer gold-standard measure that requires intensive training [4]. No previous studies have directly compared the BECCI and the MITI. We examined correlation coefficients (Spearman’s rho for skewed MITI variables) between overall BECCI scores and MITI empathy and summary scores.

**Results**

The overall BECCI scores were highly and statistically significantly (p < .05) correlated with key patient-centered counseling style MITI scores (empathy r = .70, spirit r = .74, MI-adherent r = .51) and the behavioral count scores of percent open questions. (rs = .56) and reflection-to-question ratio (rs = .60). BECCI scores were moderately and statistically significantly correlated with percent open questions (rs = .33).

**Conclusions**

The BECCI is a pragmatic measure of patient-centered behavior change counseling that may be useful for routine use in healthcare settings to assess counseling quality. Given that the BECCI does not require extensive training it may be used by either a trainer/supervisor or peer to pragmatically assess various training sessions (e.g., behavioral rehearsal [5]) as well real patient interactions (live or audio-recorded).

**References**

1. Lundahl B, Moleni T, Burke BL, Butters R, Tollefson D, Butler C, Rollnick S. Motivational interviewing in medical care settings: a systematic review and meta-analysis of randomized controlled trials. Patient Educ Couns. 2013;93(2):157-68.

2. Lane C, Huws-Thomas M, Hood K, Rollnick S, Edwards K, Robling M. Measuring adaptations of motivational interviewing: the development and validation of the behavior change counseling index (BECCI). Patient Educ Couns. 2005;56(2): 166-73.

3. Zatzick DF, Russo J, Darnell D, Chambers DA, Palinkas L, Van Eaton E, Wang J, Ingraham LM, Guiney R, Heagerty P, Comstock B. An effectiveness-implementation hybrid trial study protocol targeting posttraumatic stress disorder and comorbidity. Implement Sci. 2016;11(1):58.

4. Moyers TB, Martin T, Manuel JK, Miller WR, Ernst D. Revised global scales: motivational interviewing treatment integrity 3.1. 1 (MITI 3.1. 1). Unpublished manuscript, University of New Mexico, Albuquerque, NM. 2010.

5. Beidas RS, Cross W, Dorsey S. Show me, don’t tell me: Behavioral rehearsal as a training and analogue fidelity tool. Cogn Behav Pract. 2014;21(1):1-1.

## A117 Getting to fidelity: identifying core components of implementation facilitation strategies

### Correspondence: Jeffrey Smith (jeffrey.smith6@va.gov)

#### Behavioral Health QUERI, North Little Rock, AR, USA

**Background**

To ensure appropriate transfer of successful implementation strategies from research to policy and practice, it is important to use tools or processes to measure and support fidelity to a given strategy’s core components [1]. Unfortunately, this aspect of implementation science is underdeveloped and infrequently applied [2]. Implementation facilitation (IF) is a dynamic strategy involving interactive problem-solving and support to help clinical personnel implement and sustain a new program or practice that occurs in the context of a recognized need for improvement and a supportive interpersonal relationship [3]. Identifying core components of IF is a foundational step in efforts to develop tools to assess fidelity to the strategy.

**Materials and Methods**

First, we conducted a scoping literature review to identify the range of activities applied in IF strategies. PubMed, CINAHL, and Thompson Scientific Web of Science databases were searched for English-language articles that included the term “facilitation” or other commonly used terms for the strategy published from January 1996 – December 2015. Initially, 1,489 citations/abstracts were identified and screened for relevance by two independent reviewers. Ultimately, 135 articles (from 94 studies) were identified for abstraction of data on facilitator characteristics and roles/activities, clinical setting, patient population, clinical innovation targeted for implementation, and implementation outcomes. Next, we engaged an Expert Panel in a rigorous 3-stage modified Delphi process to develop consensus on core IF activities for high complexity and low complexity clinical innovations in three implementation phases (pre-implementation, implementation, sustainment).

**Results**

Based on review of the literature for the 94 studies, 32 distinct IF activities were identified. The Expert Panel identified 8 of the 32 IF activities as core for the Pre-Implementation Phase, 8 core IF activities for the Implementation Phase, and 4 core IF activities for the Sustainment Phase. A prototype IF Fidelity Tool based on the core activities has been developed for piloting.

**Conclusions**

Core IF activities were identified based on a comprehensive literature review and a rigorous consensus development process with an expert panel. Effective transfer of successful IF strategies from research to policy and practice requires tools to help ensure fidelity to core components of the strategy.

**References**

1. Michie S, Fixsen D, Grimshaw JM, Eccles MP. Specifying and reporting complex behaviour change interventions: the need for a scientific method. Implement Sci 2009; 4:40. doi: 10.1186/1748-5908-4-40

2. Slaughter SE, Hill JN, Snelgrove-Clarke E. What is the extent and quality of documentation and reporting of fidelity to implementation strategies: a scoping review. Implement Sci 2015;10:129. doi: 10.1186/s13012-015-0320-3

3. Powell BJ, Waltz TJ, Chinman MJ, Damschroder LJ, Smith JL, Matthieu MM, Proctor EK, Kirchner JE. A refined compilation of implementation strategies: results from the Expert Recommendations for Implementing Change (ERIC) project. Implement Sci. 2015;10(1):21. doi: 10.1186/s13012-015-0209-1

## A118 Implementation fidelity and sustainability of midwife-led antenatal consultation: preliminary results

### Anja Siegle^1^, Friederike zu Sayn-Wittgenstein^2^, Martina Roes^3^

#### ^1^University of Witten Herdecke, Witten, North Rhine-Westphalia, Germany; ^2^University of Applied Science Osnabrück, Osnabrück, Germany; ^3^German Centre for Neurodegenerative Diseases, Bonn, Germany

##### **Correspondence:** Anja Siegle (anja.siegle@med.uni-heidelberg.de)

**Background**

All over the word medical interventions in child-birth are increasing [1]. Since 2014, in Germany, there exists a national nursing expert standard to promote physiological child-birth, which demands antenatal consultation conducted by midwives who are employed by a hospital. National expert standards define an evidence-based and practitioners consented quality level using Donabedian’s model of structure, process and outcome criteria [2]. During the pilot implementation period (6 month in 2015) in 13 German hospitals, the antenatal consultation was not evaluated. Thus, to what extent antenatal consultation was implemented and how implementation success looks like remained unclear. The aim of this study is to investigate implementation fidelity (adherence, participant responsiveness) [3] and sustainability (benefits, institutionalization, development) [4] of antenatal consultation in two hospitals.

**Materials and Methods**

A mixed-methods design has been chosen, including a quantitative content analysis of consultation documents (n=154) and 34 qualitative semi structured interviews with midwifes, pregnant women, physicians and managers in two hospitals in Germany. A descriptive analysis was undertaken for the documents. The interviews were analyzed using framework analysis [5].

**Results**

Adherence is higher in hospital B, which had a longer timeframe for implementation than hospital A. Furthermore, hospital B had already experience in consultations. Participant responsiveness was very positive in both hospitals. In both hospitals, the interviewed persons saw benefits. Institutionalization is also given in both hospitals, but differs regarding time frame and consultation process. A need for evaluation of the change over time of the needs of women and tailoring interventions to these needs was seen in hospital B, but not in hospital A.

**Conclusions**

Implementing antenatal consultation in German hospitals is feasible but it needs more time than the pilot implementation of 6 month. Based on the study, it seems that a longer time period (~ 12 months) and a positive attitude to adapt to new developments increases implementation outcomes. Furthermore, flexibility in applying the 4-step implementation model, securing resources, and convincing all stakeholders might have had an impact on feasibility. Additionally, there is a need to evaluate antenatal consultation after the woman gave birth.

**References**

1. Miller S, Abalos E, Chamillard M, Ciapponi A, Colaci D, Comandé D, Diaz V, Geller S, Hanson C, Langer A, Manuelli V, Millar K, Morhason-Bello I, Castro CP, Pileggi VN, Robinson N, Skaer M, Souza JP, Vogel JP, Althabe F. Beyond too little, too late and too much, too soon: a pathway towards evidence-based, respectful maternity care worldwide. Lancet. 2016;388(10056):2176-2192.

2. DNQP. Methodisches Vorgehen zur Entwicklung, Einführung und Aktualisierung von Expertenstandards in der Pflege und zur Entwicklung von Indikatoren zur Pflegequalität auf Basis von Expertenstandards. Osnabrück 2015.

3. Dusenbury L, Brannigan R, Falco M, Hansen WB. A review of research on fidelity of implementation: implications for drug abuse prevention in school settings. Health Educ Res. 2003;18(2):237-256.

4. Fleiszer AR, Semenic SE, Ritchie JA, Richer MC, Denis JL. The sustainability of healthcare innovations: a concept analysis. J Adv Nurs. 2015;71(7):1484-1498.

5. Gale NK, Heath G, Cameron E, Rashid S, Redwood S. Using the framework method for the analysis of qualitative data in multi-disciplinary health research. BMC Med Res Methodol. 2013;13(1):117.

## A119 Preventing facilitator burnout: strategies for more sustainable process improvement

### Tanya Olmos-Ochoa^1^, David Ganz^1,2^, Jenny Barnard^1^, Lauren Penney^3,4^, Neetu Chawla^1^

#### ^1^Veterans Affairs Greater Los Angeles, Los Angeles, CA, USA; ^2^University of California Los Angeles, Los Angeles, CA, USA; ^3^South Texas Veterans Health Care System, San Antonio, TX, USA; ^4^University of Texas Health Science Center, Houston, TX, USA

##### **Correspondence:** Tanya Olmos-Ochoa (tolmos5@gmail.com)

**Background**

A substantial evidence base supports the use of practice facilitation as an effective strategy to enable implementation of evidence-based practices and related quality improvement (QI) efforts in learning healthcare systems [1-3]. Yet, challenges with implementing and maintaining facilitation exist and may impede efforts to grow and sustain an experienced facilitator workforce. This study identifies potential challenges facilitators may experience when working with QI teams in real world settings and recommends strategies to address these challenges.

**Materials and Methods**

The Coordination Toolkit and Coaching (CTAC) project is a VA-funded QI initiative to improve patient experience of care coordination in primary care. Using a cluster-randomized design, 12 primary care clinics were randomized to either a passive strategy (access to the CTAC online toolkit) or an active strategy (distance-based coaching plus access to the toolkit). Over a 12-month period, two facilitators delivered weekly, one-hour coaching calls to six clinics implementing a QI project of the clinic’s choice. Data sources included facilitator reflections catalogued after all coaching calls (n=232) and notes from debrief sessions between facilitators.

**Results**

We identified nine facilitation stressors: lack of progress/follow-through; changes to the coached team; emotion/frustration directed at the facilitator; mismatched expectations between the facilitator and coached team; managing project timeline and deliverables; supporting QI methods and data collection; managing team dynamics; promoting effective communication; and documenting implementation and facilitation processes. Given these stressors, we recommend that facilitators: continually re-assess process improvement activities and QI methods (e.g., aligning goals with project timeline); moderate discussions to help anticipate and resolve common challenges to process improvement (e.g., staffing turnover, within-team conflict); support teams with appropriate data collection and analysis; and set aside time to self-reflect (e.g., debrief sessions), discuss (e.g., with a co-facilitator), and make necessary adjustments to their facilitation process.

**Conclusions**

Understanding how facilitation affects facilitators and providing facilitators with tools to address stressors are essential for sustainability of QI and other process improvement efforts, and for continued use of facilitation as an implementation strategy. Identifying facilitation stressors and strategies to overcome them may enhance the development and maintenance of an experienced facilitator workforce to support the next generation of process improvement.

**References**

1. Harvey G, Kitson A. Implementing evidence-based practice in healthcare: a facilitation guide. New York: Routledge; 2015.

2. Harvey G, Kitson A. PARIHS revisited: from heuristic to integrated framework for the successful implementation of knowledge into practice. Implement Sci. 2016;11:33.

3. Baskerville NB, Liddy C, Hogg W. Systematic review and meta-analysis of practice facilitation within primary care settings. Ann Fam Med. 2012;10(1):63-74.

## A120 What makes an enabling context for mental health delivery? differential workload adjustment to sustain task-sharing delivery across education and health sectors in a low resource setting

### Grace Woodard^1^, Noah Triplett^1^, Christine Gray^2^, Rosemary Meza^1^, Prerna Martin^1^, Leah Lucid^1^, Kathryn Whetten^2^, Gabrielle Jamora^1^, Augustine Wasonga^3^, Cyrilla Amanya^3^, Shannon Dorsey^1^

#### ^1^University of Washington, Seattle, WA, USA; ^2^Duke University, Durham, NC, USA; ^3^ ACE Africa Kenya, Bungoma, Kenya

##### **Correspondence:** Grace Woodard (gracesw@uw.edu)

**Background**

Evidence suggests mental health interventions can be effectively delivered via task-sharing in low-resource settings with high need for mental health interventions; [1–3] however, research is needed to identify approaches to sustain the delivery in these settings. [4]

**Materials and Methods**

We examine qualitative reports of lay counselors experienced in delivering group-based trauma-focused cognitive behavioral therapy (TF-CBT) for orphaned children and adolescents in western Kenya. We analyze implementation policies and practices (IPPs) associated with delivering TF-CBT in the health and education sectors in order to determine impactful and feasible IPPs to sustain task-sharing delivery in a low-resource setting. Eighteen teachers and 18 community health volunteers (CHVs; N = 36) participated in qualitative interviews after delivering two groups of TF-CBT. Thematic coding for IPPs was conducted by a team including one PI. Interviews were double-coded and discussed to consensus; a third coder was consulted when discordant. Less than half (n = 17) of the interviews were in Swahili and were coded by a member of the study team fluent in Swahili and English; then, all Swahili interviews were translated verbally and discussed to consensus with a PI.

**Results**

Workload adjustment emerged as a critical and feasible IPP for sustaining task-sharing in the education sector: 83% of teachers (n = 15/18) indicated that limited or no workload adjustment was a barrier to implementation. However, it was minimally important in the health sector, with only 17% of CHVs (n = 3/18) indicating workload adjustment was a barrier. Teachers at urban schools (n = 6) were more likely to report workload adjustment as a facilitator than teachers at rural schools (n = 12). Examples of workload adjustments include adjustments of individual schedules (68% urban teachers versus 0% rural teachers), adjustment of school schedules (50% versus 8%), and exemption from meetings (50% versus 17%). Sustainable implementation strategies are needed to address large-scale health inequities in low-resource settings. [5] We found differential use and importance of workload adjustment in two sectors (both unique and overlapping), which enables tailored implementation support depending on the sector and setting (urban, rural).

**Conclusions**

Our results can inform future implementation and sustainment of task-sharing interventions in low resource settings.

**Trial Registration** ClinicalTrials.gov NCT01822366

**References**

1. Murray LK, Skavenski S, Kane JC, Mayeya J, Dorsey S, Cohen JA, Michalopoulos LT, Imasiku M, Bolton PA. Effectiveness of trauma-focused cognitive behavioral therapy among trauma-affected children in Lusaka, Zambia: a randomized clinical trial. JAMA Pediatr. 2015;169(8):761-769. doi:10.1001/ jamapediatrics.2015.0580

2. World Health Organization. Task Shifting. Global Recommendations and Guidelines. Geneva, Switzerland; 2008. https://www.who.int/workforcealliance/knowledge/resources/ taskshifting_guidelines/en/.

3. O’Donnell K, Dorsey S, Gong W, Ostermann J, Whetten R, Cohen JA, Itemba D, Manongi R, Whetten K. Treating maladaptive grief and posttraumatic stress symptoms in orphaned children in Tanzania: group-based trauma-focused cognitive–behavioral therapy. J Trauma Stress. 2014;27(6):664-671. doi:10.1002/jts.21970

4. Munodawafa M, Mall S, Lund C, Schneider M. Process evaluations of task sharing interventions for perinatal depression in low and middle income countries (LMIC ): a systematic review and qualitative meta-synthesis. 2018:18(1):205.

5. Eaton J, McCay L, Semrau M, Chatterjee S, Baingana F, Araya R, Ntulo C, Thornicroft G, Saxena S. Scale up of services for mental health in low-income and middle-income countries. Lancet. 2011;378(9802):1592-1603. doi:10.1016/S0140-6736(11)60891-X

## A121 The benefits of ad hoc adaptations in implementation science: community-based practices can support delivery of a family therapy intervention in Eldoret, Kenya

### Bonnie Kaiser^1^, Julia Kaufman^2^, Johnathan Taylor Wall^3^, Elsa Friis-Healy^2^, Byron Powell^4^, David Ayuku^5^, Eve Puffer^2^

#### ^1^University of California San Diego, San Diego, CA, USA; ^2^Duke University, Durham, NC, USA; ^3^Duke Global Health Institute, Durham, NC, USA; ^4^Brown School, Washington University in St. Louis, St. Louis, MO, USA; ^5^Moi University, Eldoret, Kenya

##### **Correspondence:** Bonnie Kaiser (bfullard@gmail.com)

**Background**

A key question in implementation science is how to balance adaptation and fidelity in translating interventions to new settings. Most psychological interventions carried out in low-and-middle-income countries (LMICs) were originally developed in high-income countries. There is growing consensus regarding the importance of, and processes for, planned adaptations so that interventions are delivered in contextually sensitive ways. However, little research has examined ad hoc adaptations, or those that occur spontaneously in the course of intervention delivery. A key question is whether ad hoc adaptations ultimately contribute to or detract from intervention effectiveness. This study aimed to (a) identify ad hoc adaptations made during delivery of a family therapy intervention and (b) assess whether they promoted or hindered intervention goals.

**Materials and Methods**

Tuko Pamoja (Swahili: “We are Together”) is an evidence-based family therapy intervention aiming to improve family dynamics and mental health, being delivered in Eldoret, Kenya. Tuko Pamoja is delivered by lay counselors, who are afforded a degree of flexibility in the way they present intervention content and the practices they use in therapy sessions. This study used transcripts of therapy sessions with 14 families to develop a taxonomy of ad hoc adaptations used by counselors. We first identified and characterized these adaptations. Then, we evaluated to what extent they were in the spirit of the intervention or went against the goals of the intervention.

**Results**

Ad hoc adaptations included the incorporation of metaphors and proverbs, religious content, self-disclosure, examples and role models, discussing interpersonal relationships outside of the family, and community dynamics and resources. For the most part, practices were Tuko Pamoja-promoting, though Tuko Pamoja-contrary practices were also identified.

**Conclusions**

Identifying helpful ad hoc adaptations and incorporating them into interventions could improve acceptability, feasibility, and effectiveness.

**Trial Registration** ClinicalTrials.gov NCT03360201

## A122 Adaptations to an evidence-based health promotion practice implemented nationally in routine mental health settings

### Kelly Aschbrenner^1^, Gary Bond^2^, Sarah Pratt^1^, Stephen Bartels^3^

#### ^1^Geisel School of Medicine at Dartmouth, Hanover, NH, USA; ^2^Weststat, Rockville, MD, USA; ^3^Massachussetts General Hospital, Boston, MA, USA

##### **Correspondence:** Kelly Aschbrenner (kelly.aschbrenner@dartmouth.edu)

**Background**

There is increasing recognition that local program adaptations may be instrumental to sustaining evidence-based interventions in routine clinical practice. [1] However, few empirical studies have documented naturally occurring adaptations made during the implementation process by providers and agencies in health care settings. Our research directly addresses the SIRC conference theme, “Where the Rubber Meets the Road,” by identifying and categorizing provider-initiated adaptations to an evidence-based health promotion practice implemented nationally in routine mental health care settings.

**Materials and Methods**

Our team conducted semi-structured telephone interviews with program staff from 35 behavioral health organizations 24 months after they implemented InShape, a manualized evidence-based health promotion practice for persons with serious mental illness, within the context of an NIMH-funded implementation study. The interview protocol included questions that assessed core fidelity components of the InSHAPE model, with probes used to explore any adaptations made to core components. An adaptation was defined as a change to the intervention content or method of delivery that was not specified in the original treatment manual. We explored the reasons why an adaptation was made as well as who initiated it at the agency. Two investigators independently reviewed interview transcripts to identify adaptations to the program.

Adaptations to InSHAPE included hybrid individual and group programs, home-based exercise programs, technology-based enhancements, such as mobile fitness apps and wearable activity trackers, and use of peer support specialists to deliver program components. The next level of analysis will involve classifying adaptations as fidelity-consistent (i.e., changes that do not significantly alter core model elements) and fidelity-inconsistent adaptations (i.e., changes that reduce the delivery of core model elements), [2] and categorizing the drivers of adaptations (e.g., client-driven, financially-driven). We will then evaluate the impact of these adaptations on client-level health outcomes.

**Results**

Evidence-based practices are often modified by agencies when translated from research environments to real world health care settings. [3] However, the impact of these adaptations on client health outcomes is not well understood.

**Conclusions**

By identifying and characterizing site-specific adaptations, we will be able to explore the relationship of adaptations to program-and participant-level outcomes and sustainability of the InSHAPE program at the completion of the study.

**References**

1. Chambers DA, Glasgow RE, Stange KC. The dynamic sustainability framework: addressing the paradox of sustainment amid ongoing change. Implement Sci. 2013;8:117.

2. Wiltsey Stirman S, A Gutner C, Crits-Christoph P, Edmunds J, Evans AC, Beidas RS. Relationships between clinician-level attributes and fidelity-consistent and fidelity-inconsistent modifications to an evidence-based psychotherapy. Implement Sci. 2015;10(1):115.

3. Escoffery C, Lebow-Skelley E, Haardoerfer R, Boing E, Udelson H, Wood R, Hartman M, Fernandez ME, Mullen PD. A systematic review of adaptations of evidence-based public health interventions globally. Implement Sci. 2018;13(1):125.

## A123 Evidence-based quality improvement for accelerating patient-centered medical home implementation: impact on patient-provider communication

### Alexis Huynh, Danielle Rose, Martin Lee, Catherine Chanfreau-Coffinier, Karleen Giannitrapani, Lisa Rubenstein, Susan Stockdale

#### Veterans Health Administration, Bedford, MA, USA

##### **Correspondence:** Alexis Huynh (alexis.huynh@va.gov)

**Background**

High-quality patient-provider communication is foundational to patient-centered care and a core component of the Patient-Centered Medical Home (PCMH) [1]. The PCMH model could disrupt patient-provider communication by shifting communication responsibilities to non-provider PCMH team members [2,3]. We introduced Evidence-Based Quality Improvement for PCMH transformation (EBQI-PCMH) at seven Veterans Affairs (VHA) primary care practices. Quality improvement (QI) methods to address PCMH implementation challenges included improving patient-provider communication. This paper examines EBQI-PCMH effectiveness for improving patient-provider communication over time, compared with standard PCMH implementation (PCMH-only).

**Materials and Methods**

We used a non-randomized stepped wedge design in which sites entered in three phases, 6-8 quarters apart. We compared Veterans’ experiences at 10 VHA practices (seven EBQI-PCMH versus three PCMH-only) on patient-provider communication. In PCMH-only transformation, providers and staff in all primary care sites received training in motivational interviewing and patient-centered communication. In EBQI-PCMH sites researchers partnered with clinical leaders to support local development of QI projects addressing patient-provider communication. We used repeated cross-sections of nationally-administered patient experience surveys from 2009-2015 to assess EBQI-PCMH impacts (N=34,193). Outcome measures included patient ratings of four provider communication skills: 1) explaining information (EXPLAIN), 2) listening (LISTEN), 3) showing respect (RESPECT), and 4) spending enough time (TIME), and rated as optimal for scores 9-10 vs lower. Predictors included time, EBQI-PCMH implementation, and length of exposure to EBQI-PCMH. We compared EBQI-PCMH to PCMH-only practice sites using multi-level, multivariate modelling controlling for patient and site characteristics and weighted for non-response.

**Results**

Patient ratings of all provider communication skills improved with longer exposure to EBQI-PCMH, adjusting for patient and site characteristics. Each additional quarter of exposure to EBQI-PCMH was associated with improved odds of optimal communication: 2.75% increase in EXPLAIN, 2.95% increase in LISTEN, 2.70% increase in RESPECT, and 2.29% increase in TIME. For example, over the span of the evaluation period (23 quarters), the predicted probability of higher rating for EXPLAIN was 76%, 73%, and 68%, for EBQI-PCMH for Phase 1, 2, 3 versus 63% for PCMH-only.

**Conclusions**

EBQI-PCMH that engages leaders, providers and staff in researcher-supported QI to accelerate patient centered transformation can be effective in improving patient-provider communication.

**References**

1. Stewart M, Brown J, Donner A, et al. The impact of patient-centered care on outcomes. J Fam Prac. 2000;49(9):796-804.

2. Rubenstein LV, Stockdale SE, Sapir N, Altman L, Dresselhaus T, Salem-Schatz S, Vivell S, Ovretveit J, Hamilton AB, Yano EM. A patient-centered primary care practice approach using evidence-based quality improvement: rationale, methods, and early assessment of implementation. J Gen Intern Med. 2014; 29(2):589-597.

3. Reddy A, Canamucio A, Werner RM. Impact of the patient-centered medical home on veterans’ experience of care. Am J Manag Care. 2015;21(6):413-421.

## A124 Facilitators’ perspectives on facilitation successes and challenges in a quality improvement initiative

### Neetu Chawla^1^, David Ganz^1^, Jenny Barnard^1^, Lauren Penny^2^, Tanya Olmos-Ochoa^1^

#### ^1^VA Greater Los Angeles, Los Angeles, CA, USA; ^2^Veteran’s Health Administration South Texas, San Antonio, TX, USA

##### **Correspondence:** Neetu Chawla (neetu.chawla@va.gov)

**Background**

Quality improvement efforts and implementation science use facilitation as an effective implementation strategy. Limited work has examined the successes and challenges of this strategy from the facilitator’s perspective, which could shed light on evaluation of facilitation effectiveness and success of implementation efforts [1-2].

**Materials and Methods**

We conducted thematic analysis on qualitative data from the Coordination Toolkit and Coaching (CTAC) project, a multi-site quality improvement initiative within the VA healthcare system. Two CTAC facilitators (“coaches”) logged their perceptions of the successes and challenges in a “reflection” template completed after each weekly one-hour coaching call over the 12-month project period. Given CTAC is ongoing, this analysis examines the successes and challenges identified for one coached site (n=41 reflections). Two members of the project team independently coded the reflections to identify common themes related to successes and challenges resulting from the coaching process.

**Results**

We identified 15 total themes related to successes or challenges, of which six were categorized as both a success and a challenge: Project participation and engagement; Communication between coach and coached team members; Managing team dynamics; Conflict resolution; Time management; and Call productivity (e.g., progress on CTAC deliverables, project tasks, or products). For example, for the theme of moderating team dynamics, coaches described obtaining “buy-in from all the stakeholders and facilitating the discussions between them” as a success but simultaneously noted that “balancing nursing priorities/frustrations with administrative staff’s priorities/frustrations” was a challenge. Similarly, for the theme of communication, coaches noted “encouraging more people to speak up” as a success but also “getting them to be more verbal during the call” as a challenge.

**Conclusions**

Facilitation is increasingly used as an implementation strategy, yet the successes and challenges experienced by facilitators during the facilitation process are not well-defined [3]. Given that effective facilitation may lead to positive implementation outcomes, facilitation success should be examined carefully. Our findings indicate that some aspects of coaching can be assessed as both successes and challenges, highlighting the complexity of the facilitation process. Better understanding facilitation effectiveness will support a more nuanced conceptualization of how implementation efforts that use facilitation either fail or succeed.

**Trial Registration** ClinicalTrials.gov NCT03063294

**References**

1. Harvey G, McCormack B, Kitson A, Lynch E, Titchen A. Designing and implementing two facilitation interventions within the ‘Facilitating Implementation of Research Evidence (FIRE)’ study: a qualitative analysis from an external facilitators’ perspective. Implement Sci. 2018;13(1):141.

2. Rycroft-Malone J, Seers K, Eldh AC, et. al. A realist process evaluation within the Facilitating Implementation of Research Evidence (FIRE) cluster randomised controlled international trial: an exemplar. Implement Sci. 2018;13(1):138.

3. Baskerville NB, Liddy C, Hogg W. Systematic review and meta-analysis of practice facilitation within primary care settings. Ann Fam Med. 2012;10(1):63-74.

## A125 Building an impactful implementation support model to scale-up a quality improvement program in long-term care

### Andrea Chaplin, Sam MacFarlane

#### Public Health Ontario, Toronto, Ontario, Canada

##### **Correspondence:** Andrea Chaplin (andrea.chaplin@oahpp.ca)

**Background**

A provincial agency is working to scale an organizational improvement program that supports long-term care homes overcome barriers to aligning with evidence based practices related to the assessment and management of urinary tract infections [1-2]. These practices, if addressed, could help reduce the overuse of antibiotics that are contributing to antibiotic resistance and increased risk of antibiotic side effects. The initial pilot of this program involved agency staff delivering in-person support to an implementation team that was established in 12 long-term care homes. With over 600 long-term care homes in the province of Ontario, a more efficient implementation support model was needed. The purpose of this phase of the project was to apply best practices from implementation science to develop and evaluate a new implementation model that could be used to scale-up the program.

**Materials and Methods**

The Quality Implementation Framework [3] and evidence-based system for innovation support [4] were used to inform the development of implementation supports at the agency and long-term care home level. Five agency staff conducted readiness conversations and delivered group-based online implementation training sessions to leads from 44 long-term care homes. Two online surveys were administered to the leads from each home to assess fidelity to the program recommendations and to gather feedback on the quality of the training sessions.

**Results**

Participation rates were variable, with only 29% of long-term care homes attending all three scheduled sessions. Of the homes that continued with the program, over 70% had adopted implementation strategies designed to support readiness and buy-in for the practice changes. In April, data from the final survey will be analyzed to describe what program strategies were used by participating homes based on a fidelity measurement tool established for the program.

**Conclusions**

An online and group-based implementation support model has proven to be efficient in reaching more homes; however, there is a need to assess the implications of this higher touch approach on program fidelity. This model has raised questions about how an intermediary can support stakeholders secure buy-in, plan for sustainability, and be inspired to adopt approaches from implementation science.

**References**

1. Chambers A, MacFarlane S, Zvonar R, Evans G, Moore JE, Langford BJ, Augustin A, Cooper S, Quirk J, McCreight L, Garber G. Recipe for antimicrobial stewardship success: Using intervention mapping to develop a program to reduce antibiotic overuse in long-term care. Infect Control Hosp Epidemiol. 2019;40(1):24-31.

2. Brown AK, Chambers A, MacFarlane S, Langford B, Leung V, Quirk J, Schwartz KL, Garber G. Reducing unnecessary urine culturing and antibiotic overprescribing in long-term care: outcomes of an implementation science informed before and after study. CMAJ Open. 2019;7(1);E174–E181.

3. Meyers DC, Durlak JA, Wandersman A. The quality implementation framework: a synthesis of critical steps in the implementation process. Am J Community Psychol. 2012;50(3-4):462-80.

4. Wandersman A, Chien VH, Katz J. Toward an evidence-based system for innovation support for implementing innovations with quality: tools, training, technical assistance, and quality assurance/quality improvement. Am J Community Psychol. 2012;50(3-4):445-459.

## A126 Early lessons from formative evaluation of an implementation intervention to improve reach of evidence-based psychotherapies for PSTD

### Princess Ackland^1,2^, Shannon Kehle-Forbes^1,2,3^, Matthew Yoder^3,4^, Robert Orazem^1^, Nancy Bernardy^3,5^, Jessica Hamblen^3,5^, Craig Rosen^3,6^, Siamak Noorbaloochi^2^, Barbara Clothier^1^, Sean Nugent^1^, Paula Schnurr^3,5^, Nina Sayer^1,2^

#### ^1^Minneapolis VA Health Care System, Minneapolis, MN, USA; ^2^University of Minnesota, Minneapolis, MN, USA; ^3^National Center for PTSD, VA Palo Alto Health Care System, Palo Alto, CA, USA; ^4^Medical University of South Carolina, Charleston, SC, USA; ^5^Dartmouth College, Hanover, NH, USA; ^6^Stanford University, Stanford, CA, USA

##### **Correspondence:** Princess Ackland (princess.ackland@va.gov)

**Background**

We used toolkit-guided external facilitation to improve access to evidence-based psychotherapies (EBPs) for PTSD in two outpatient PTSD clinics with low reach of EBPs (≤15% Veterans with PTSD) at baseline. The Promoting Action on Research Implementation in Health Services framework informed the implementation strategy and evaluation [1]. The objective of this study is to describe preliminary results from the formative evaluation.

**Materials and Methods**

Developmental evaluation data included pre-site visit interviews with 4-6 key informants and baseline data on the primary implementation outcome—EBP reach, defined as the percentage of unique patients who receive a session of Prolonged Exposure or Cognitive Processing Therapy in the PTSD clinic. Implementation-focused evaluation data was extracted from a facilitation log used to track facilitation activities, time spent in these activities and contact with the champion and other local staff. Progress-focused evaluation data included monthly audit and feedback reports on EBP reach, based on administrative data, and narrative review of goal attainment recorded in the site-specific guide. Interpretive evaluation data included post-intervention interviews with the same key informants interviewed at baseline. Interviews were analyzed using rapid turn-around approach [2].

**Results**

EBP reach more than doubled during the 6-month intervention period in both clinics. Clinic A achieved its reach goal of 20% in month 2 and continued to increase linearly to 36% at month 6. Clinic B’s reach increased slightly then plateaued until month 6 when it achieved its goal of 25%. External facilitation hours were 70% greater in Clinic A. Clinic A implemented organizational changes consistently over the 6 months while Clinic B enacted significant changes shortly before the 6-month reach increase. Clinic A’s champion was more committed and empowered to make organizational changes compared with Clinic B’s champion. Implementation strategies associated with reach at both sites included audit and feedback reports, an in-person site visit at project launch, and toolkit resources.

**Conclusions**

Improvement trajectories may not be consistent across sites. Implementation interventions should vary in duration according to local champion characteristics. Toolkit-guided external facilitation accompanied by a strong local champion has the potential to help clinics reorganize to improve reach of EBPs to patients.

**References**

1. Rycroft-Malone J. Promoting action on research implementation in health services (PARIHS). In: Rycroft-Malone J, Bucknall T, editors. Models and frameworks for implementing evidence-based practice: linking evidence to action. Hoboken, NJ: John Wiley & Sons; 2010. p. 109-135.

2. Hamilton AB. Qualitative methods in rapid turn-around health services research. VA HSR&D Cyberseminar, December 2013.

## A127 Addressing social disconnection among frequent users of community hospital emergency departments: a statewide implementation evaluation

### Rani Elwy^1^, Elisa Koppelman^1^, Victoria Parker^2^, Chris Louis^1^

#### ^1^Boston University, Boston, MA, USA; ^2^University of New Hampshire, Durham, NH, USA

##### **Correspondence:** Rani Elwy (rani_elwy@brown.edu)

**Background**

Chapter 224 of the Commonwealth of Massachusetts Acts of 2012 [1] authorized Massachusetts to establish the Community Hospital Acceleration, Revitalization, and Transformation (CHART) investment program. The Massachusetts Health Policy Commission (HPC) oversees the CHART program, which awarded $120 million to 27 community hospitals to develop innovations aimed at enhancing the delivery of efficient, effective care, and readying them for value-based care [2].

Objective: Through a contract with the HPC, we conducted an implementation evaluation of CHART innovations between 2016-2018. Through this evaluation, we examined how CHART stakeholders described social disconnection, a public health priority, and which levels of a social connection framework CHART innovations addressed (structural, functional, quality or multilevel) [3] among frequent emergency department (ED) users.

**Materials and Methods**

Qualitative interviews with 236 stakeholders (hospital managers, CHART providers, staff, and community partners) one year post CHART implementation were audiorecorded and transcribed verbatim. Interviews were analyzed using a directed content analysis approach [4]. We assessed reliability and validity of our coding frame through joint coding by four analysts on two transcripts. Data were then mapped to the levels of the social connection framework. Each coded transcript was discussed in depth until consensus on coding definitions was reached. Following this process, six analysts independently coded between 30-40 transcripts. Data were checked and entered into the NVivo software package for ease of organization and reporting.

**Results**

Social disconnection, described as “loneliness” and “social isolation” by stakeholders, led patients to the ED for problems not always related to their physical health. These definitions mapped to the structural level of the social connection framework. Innovations involving home visit programs, elder services interventions, workflow changes in the ED, and regular telephone follow-ups provided functional level emotional and tangible support. Stakeholders did not mention relationship distress or quality of relationships in describing social disconnection or hospital innovations.

**Conclusions**

Innovations to address high ED use, according to stakeholders, provided functional level emotional and tangible support to address structural level definitions of social disconnection. Future work should examine the sustainability of these innovations in a value-based healthcare climate, and the effectiveness of these programs on reducing ED utilization.

**References**

1. The 191st General Court of the Commonwealth of Massachusetts. Acts of 2012. Chapter 224. An act improving the quality of health care and reducing costs through increased transparency, efficiency and innovation. https://malegislature.gov/Laws/SessionLaws/Acts/2012/Chapter224. Accessed 31 March 2019.

2. Louis C, Bachman S, Roby D, Melby L, Rosenbloom D. The transformation of community hospitals through the transition to value-based care: lessons from Massachusetts. Am J Account Care. 2017;5(4):26-30.

3. Holt-Lundstadt J, Robles TF, Sbarra DA. Advancing social connection as a public health priority in the United States. Am Psychol. 2017;72(6):517-530.

4. Hsieh HF, Shannon SE. Three approaches to qualitative content analysis. Qual Health Res. 2005; 15(9):1277-1288.

## A128 Evaluation of The Implementation Game^©^: a learning and planning resource

### **Correspondence:** Melanie Barwick (melanie.barwick@sickkids.ca)

#### The Hospital for Sick Children, Toronto, Ontario, Canada

**Background**

The presentation will share evaluation findings for a new planning and learning resource to support implementation of evidence into care. Implementation is a complex process with many moving parts, and many practitioners and organizations struggle to do it successfully. The Implementation Game© (TIG) supports autonomous, self-direct implementation by simplifying the process into five main components to provide an implementation planning experience for an identified scenario or implementation endeavor. It is based on key implementation theories, models, and frameworks [1-4].The Implementation Game is relevant to any discipline because the concepts are high level. The Game components include a game board, playing cards, and an implementation worksheet to capture the plan. The goal is either to learn, or to plan, or both. The presentation will provide an overview of the Game and preliminary evaluation data.

**Materials and Methods**

An online survey has been shared with 36 (and counting) individuals who either purchased or received a copy of The Implementation Game© beginning in December 2018. The survey captures evidence of use, usefulness, spread, quality, and satisfaction.

**Results**

Currently in data collection.

**Conclusions**

Evaluation results will be used to refine the Game and to inform a prototype for an online software platform that is under development by the author.

**References**

1. Damschroder LJ, Aron DC. Keith RE, Kirsh SR, Alexander JA, Lowery JC. Fostering implementation of health services research findings into practice: a consolidated framework for advancing implementation science. Implement Sci. 2009;4:50.

2. Myers DC, Durlak JA, Wandersman A. The Quality Implementation Framework: a synthesis of critical steps in the implementation process. Am J Community Psychol. 2012;50(3-4):462-80.

3. Powell BJ, Waltz TJ, Chinman MJ, Damschroder LJ, Smith JL, Matthieu MM, Proctor EK, Kirchner JE. A refined compilation of implementation strategies: results from the Expert Recommendations for Implementing Change (ERIC) project. Implement Sci. 2015;10(1):21.

4. Proctor E, Silmere H, Raghavan R, Hovmand P, Aarons G, Bunger A, Griffey R, Hensley M. Outcomes for implementation research: conceptual distinctions, measurement challenges, and research agenda. Adm Policy Ment Health. 2011;38(2):65-76. doi: 10.1007/s10488-010-0319-7.

## A129 Understanding when consultation supports teachers in implementing a prevention program in South African high schools: moderators and outcomes

### Mojdeh Motamedi^1^, Linda Caldwell^2,3^, Edward Smith^2,3^, Lisa Wegner^3^, Joachim Jacobs^3^

#### ^1^University of California, San Diego, CA, USA; ^2^The Pennsylvania State University, University Park, PA, USA; ^3^University of the Western Cape, Cape Town, South Africa

##### **Correspondence:** Mojdeh Motamedi (momotamedi@ucsd.edu)

**Background**

Despite implementation research on supporting evidence-based prevention programs in high-income countries, research is lacking on consultation support in schools in low-resourced countries like high schools surrounding Cape Town, South Africa [1]. This study is part of a larger factorial design implementation trial of HealthWise, a teacher taught program for preventing youths’ risky sexual and substance use behaviors [2].

**Materials and Methods**

After initial randomization, 22 schools with 33 teachers received the consultation condition while 26 schools with 41 teachers did not. The consultation condition included three meetings between the consultant and a teacher representative per school, text message reminders, support kits with prepared HealthWise materials, and lesson plans integrating HealthWise content. Teachers self-reported how much content they delivered and adapted, and students’ interests in HealthWise lessons during 9th grade. Observer coded videos captured teachers’ fidelity to HealthWise curriculum. School risk was calculated using publicly available data on school and community safety, poverty, density and geographical location. Post-intervention qualitative interviews with the consultant, 11 teachers and 4 principals expand on quantitative findings and broader policy and community priorities.

**Results**

Based on as-treated regression analyses, teachers in the consultation condition reported delivering more HealthWise content (B = .13, p < .01) but did not differ in their observed fidelity. Moderation analyses found teachers with lower educational degrees who received the consultation condition reported more student interest in HealthWise (B = -.17, p < .01), and teachers in higher risk schools that received the consultation condition reported more adaptation (B = .22, p < .01). Initial qualitative findings suggest there was a need to adapt, especially to address higher risk school needs. Additionally, the consultant’s interview suggested racial, educational, and gender differences may play a role in teachers’ receptivity to consultation.

**Conclusions**

Findings suggest even a low dose of consultation support can facilitate implementation outcomes in this context. As this study occurred during South African education policy changes regarding teaching life skills in high schools [3], we will discuss how consultation can support implementation to meet community, teacher and student needs in the context of policy changes, as well as potential shortcomings of consultation.

**Trial Registration** ClinicalTrials.gov NCT00336180

**References**

1. Powell BJ, Waltz TJ, Chinman MJ, Damschroder LJ, Smith JL, Matthieu MM, Proctor EK, Kirchner JE. A refined compilation of implementation strategies: results from the Expert Recommendations for Implementing Change (ERIC) project. Implement Sci. 2015;10(1):21. doi:10.1186/s13012-015-0209-1.

2. Caldwell L, Smith E, Collins L, Graham J, Lai M, Wegner L, Vergnani T, Matthews C, Jacobs J. Translational research in South Africa: Evaluating implementation quality using a factorial design. Child Youth Care Forum. 2012;41(2):119-136.

3. Maharajh LR, Nkosi T, Mkhize MC. Teachers’ experiences of the implementation of the Curriculum and Assessment Policy Statement (CAPS) in three primary schools in KwaZulu Natal. Africa’s Public Serv Deliv Perform Rev. 2017;4(3): 371. doi:10.4102/apsdpr.v4i3.120.

## A130 Adoption of trauma-focused interventions within rural schools

### Heather Halko^1^, Kaoru Powell^1^, Erika Burgess^1^, Cameo Stanick^2^, Kaitlyn Ahlers^1^, Anisa Goforth^1^

#### ^1^University of Montana, Missoula, MT, USA; ^2^Hathaway-Sycamores Child and Family Services, Los Angeles, CA, USA

##### **Correspondence:** Heather Halko (heather.halko@umontana.edu)

**Background**

High rates of childhood trauma exposure (over 68%) create significant concern given the negative outcomes associated with trauma-related symptoms [1-2]. Numerous trauma-focused evidence-based practices (EBPs) have been developed; however, little is known about why school systems, especially those serving rural areas, adopt (or do not adopt) trauma-focused EBPs. This qualitative study explored factors that might influence the adoption of trauma-focused interventions among clinicians working in rural schools using two implementation science frameworks: the Consolidated Framework for Implementation Research (CFIR) and the Implementation Outcome Framework (IOF) [3-4].

**Materials and Methods**

A semi-structured protocol was used to interview school-based clinicians (N = 12) about their knowledge, views, and adoption of trauma-focused interventions. Specific attention was given to IOF outcomes known to influence innovation adoption (i.e., acceptability, appropriateness, and feasibility) [5]. Transcripts were double coded using a deductive content analysis approach and a CFIR- and IOF-based coding manual.

**Results**

Every participant (100%) reported adopting some form of mental health intervention to treat symptoms of posttraumatic stress within their school setting, though only 25% had adopted a trauma-focused EBP. One participant (8.33%) was also working in a school that declined an opportunity to adopt the practice of delivering trauma-focused care. Thematic analyses revealed that most participants reported the same acceptability and appropriateness factors as both facilitators and barriers to adoption of trauma-focused interventions in rural schools. Nine participants (75%) believed that it was not feasible to implement trauma-focused EBPs within their current school system. Several CFIR constructs (e.g., cosmopolitanism, structural characteristics, leadership engagement, access to knowledge and information, available resources, relative priority, self-efficacy) were commonly identified as influencing the feasibility of implementing trauma-focused interventions within a rural school.

**Conclusions**

The acceptability and appropriateness of delivering trauma-focused care within school settings appears to positively influence the adoption of trauma-focused interventions within rural schools. However, limited feasibility of implementing trauma-focused EBPs within rural schools might be negatively influencing adoption. These results have the capacity to inform a targeted approach to select implementation strategies that could enhance the adoption of trauma-focused EBPs within schools, thereby increasing the accessibility of trauma-focused care in rural areas.

**References**

1. Copeland WE, Keeler G, Angold A, Costello EJ. Traumatic events and posttraumatic stress in childhood. Arc Gen Psychiatry. 2007;64:577-584.

2. Loeb J, Stettler EM, Gavila T, Stein A, Chinitz S. The Child Behavior Checklist PTSD scale: Screening for PTSD in young children with high exposure to trauma. J Trauma Stress. 2011;24(4):430-434.

3. Damschroder LJ, Aron DC. Keith RE, Kirsh SR, Alexander JA, Lowery JC. Fostering implementation of health services research findings into practice: a consolidated framework for advancing implementation science. Implement Sci. 2009;4:50.

4. Proctor E, Silmere H, Raghavan R, Hovmand P, Aarons G, Bunger A, Griffey R, Hensley M. Outcomes for implementation research: conceptual distinctions, measurement challenges, and research agenda. Adm Policy Ment Health. 2011;38(2):65-76.

5. Chor KH, Wisdom JP, Olin SC, Hoagwood KE, Horwitz SM. Predictors of innovation adoption. Adm Policy Ment Health. 2015;42(5):545-573.

## A131 The impact of stakeholder alignment of the organizational implementation context in schools

### Elissa Picozzi^1^, Chayna Davis^1^, Jill Locke^1^, Mark Ehrhart^2^, Eric Brown^3^, Clay Cook^4^, Aaron Lyon^1^

#### ^1^University of Washington, Seattle, WA, USA; ^2^University of Central Florida, Orlando, FL, USA; ^3^University of Miami, Coral Gables, FL, USA; ^4^University of Minnesota, Minneapolis, MN, USA

##### **Correspondence:** Elissa Picozzi (epicozzi@uw.edu)

**Background**

Organizational factors are critical to successful implementation of evidence-based behavioral health interventions. Previous research has demonstrated that the alignment or misalignment between leadership and providers on organizational constructs (e.g., implementation leadership) impacts successful implementation [1]. In particular, positive misalignment, which occurs when leaders rate the implementation context significantly lower than the staff, may facilitate the successful implementation of evidence-based practices, and negative misalignment may hinder these processes. This paper examines alignment between school administrators and their staff on implementation leadership (i.e., specific leadership behaviors that support or inhibit effective implementation) and implementation climate (i.e., shared norms and expectations among staff related to implementation).

**Materials and Methods**

The OASIS study collected data for alignment in 35 schools (6 school districts in 3 states). Leadership (n=35) and staff (n=289) were asked to rate the implementation leadership (IL) and implementation climate (IC) of their site. Alignment and directionality of alignment (i.e., a positive relationship between leadership and staff ratings, or negative misalignment) between these two groups was calculated as less than a half standard deviation in the difference between mean scores [2-3]. Fidelity assessments for two universal behavioral health prevention programs were then conducted, and further analysis will determine if alignment directionality affects implementation fidelity.

**Results**

A significant proportion of sites reported negative misalignment between leadership and staff. 37.1% of sites reported negative misalignment on overall perceptions of IL, and 51.4% reported negative misalignment on overall perceptions of IC. Additionally, negative misalignment across the seven IL subscales (i.e., proactive, knowledgeable, supportive, perseverant, communication, vision, and availability) ranged from 25.7% to 51.4%, and negative misalignment across the seven IC subscales (i.e., focus, rewards, use of data, integration, existing support, recognition, and educational support) ranged from 34.3% to 60.0%.

**Conclusions**

This paper illuminates important findings with implications for implementation research and practice. First, negative misalignment was found for over half of the schools, indicating a likely need to improve communication and collaboration across levels. Second, although fewer in number, positive alignment and positive misalignment was noted, indicating opportunities to study the factors associated with optimal alignment on key organizational implementation constructs.

**References**

1. Aarons GA, Ehrhart MG, Farahnak LR, Sklar M. Aligning leadership across systems and organizations to develop a strategic climate for evidence-based practice implementation. Annu Rev Public Health. 2014; 35(1):255-274. doi:10.1146/annrev-publhealth-032013-182447

2. Aarons GA, Ehrhart MG, Torres EM, Finn NK, Beidas R. The humble leader: Association of discrepancies in leader and follower ratings of implementation leadership with organizational climate in mental health. Psychiatr Serv Wash DC. 2017;68(2):115-122. doi:10.1176/appi.ps.201600062

3. Lyon AR, Cook CR, Brown EC, Locke J, Davis C, Ehrhart M, Aarons GA. Assessing organizational implementation context in the education sector: confirmatory factor analysis of measures of implementation leadership, climate, and citizenship. Implement Sci. 2018;12(1):5.

## A132 LIFT Together with Boys Town: an implementation system bridging schools, providers, and researchers

### Jasney Cogua^1^, W. Alex Mason^2^

#### ^1^Boys Town - LIFT Together, Boys Town, NE, USA; ^2^Boys Town Child and Family Translational Research Center, Boys Town, NE, USA

##### **Correspondence:** Jasney Cogua (jasney.cogua@boystown.org)

**Background**

For over 100 years, Boys Town has provided services for children who have suffered adverse experiences, trauma, and other challenges [1]. Since 2012, Boys Town has been working to implement a comprehensive prevention strategy that goes beyond serving individual youth and families to impacting targeted populations to build well-being through the LIFT Together program [2].

**Materials and Methods**

LIFT Together is a community-based implementation system that convenes schools, service providers, and researchers to facilitate the delivery of a multi-tier, multi-component intervention package. The intervention package uses school [3] - and family-based [4-6] programs to generate school-wide impact. Outcomes are measured at the school population level (such as preventing and reducing school disciplinary referrals or increasing parental engagement at schools), instead of upon individual children and families. This intervention system is being implemented within highly vulnerable communities in three sites: South Omaha, Nebraska; North Las Vegas, Nevada; and Pawtucket, Rhode Island.

**Results**

This presentation will include:

1) A brief explanation of the LIFT Together System (processes, components, and outcomes).

2) A description of the lessons learned and challenges faced in the implementation of LIFT Together from the perspectives of the school, collaborating providers, and researchers. Examples include developing a common understanding of the goals and processes, establishing protocols for access to the critical populations, and implementing practices for school engagement.

3) A description of data collection processes for the evaluation of LIFT Together implementation and goals (e.g., integrating school and service provider data to evaluate outcomes).

**Conclusion**

This presentation provides an illustration of implementation practice in real-world settings in the delivery and evaluation of a systematic, community-based system for mobilizing schools to address student concerns with a tiered package of school- and family-based programs. This presentation will not only illuminate the specific nature of LIFT Together, but it also will elucidate contextual factors for local success and highlight the types of expertise and knowledge needed to facilitate local partnerships and implement initiatives that benefit vulnerable youth and families in community settings.

**References**

1. Thompson RW, Daly DL. The Family Home Program: An adaptation of the Teaching-Family Model at Boys Town. In: Whittaker JK, Del Valle JF, Holmes L, editors. Therapeutic residential care with children and youth: developing evidence-based international practice London and Philadelphia: Kingsley Publishers; 2015. p. 113- 123.

2. Mason WA, Cogua JE, Thompson RW. Turning a big ship: unleashing the power of prevention within treatment settings. J Soc Social Work Res. 2018; 9(4): 765-781. https://doi.org/10.1086/700847

3. Oliver, RM, Lambert MC, Mason WA. A pilot study for improving classroom systems within schoolwide positive behavior support. J Emot Behav Disord. 2019; 27(1):25–36. https://doi.org/10.1177/ 1063426617733718

4. Mason WA, January S-A., Fleming CB, Thompson RW, Parra GR, Haggerty KP, Snyder JJ. Parent training to reduce problem behaviors over the transition to high school: Tests of indirect effects through improved emotion regulation skills. Child Youth Serv Rev. 2016;61:176-183.

5. Anderson L, Ringle JL, Ross JR, Ingram SD, Thompson RW. Care coordination services: a description of an alternative service model for at-risk families. J Evid Inf Soc Work. 2017;14:217-228. http:doi.org/ 10.1080/23761407.2017.1306731

6. Patwardhan I, Duppong Hurley K, Thompson RW, Mason WA, Ringle JL. Child maltreatment as a function of cumulative family risk: Findings from the intensive family preservation program. Child Abuse Negl. 2017; 70:92–99. http://doi.org/ 10.1016/j.chiabu.2017.06.010

## A133 Engaging underserved communities in implementation research: strategies for success in the Adaptive School-based Implementation of CBT (ASIC) Trial

### Amy Rusch, Jennifer Vichich, Kristen Miner, Seoyoun Choi, Michael Prisbe, Elizabeth Koschmann, Celeste Liebrecht, Amy Kilbourne, Shawna Smith

#### University of Michigan, Ann Arbor, MI, USA

##### **Correspondence:** Amy Rusch (amyrusch@med.umich.edu)

**Background**

Implementation studies are often criticized for engaging only early adopter sites, thus limiting study generalizability. Better implementation science requires understanding optimal tactics for engaging stakeholders in implementation research. Adaptive School-based Implementation of CBT (ASIC) is a large-scale randomized trial designed to test different implementation strategies to support school professional (SP) delivery of cognitive-behavioral therapy (CBT) in high schools across Michigan [1]. We analyzed methods used to recruit SPs at more than 100 diverse schools for ASIC participation and describe successful strategies used in this large-scale implementation study across different settings [2] and stages of change [3].

**Materials and Methods**

Schools were recruited to ASIC over a 6-month period. A post-hoc process evaluation of recruitment was conducted. Metrics collected include quantitative measures (e.g., number of attempts) and qualitative feedback from recruiters on successful recruitment strategies. Following recruitment, data were analyzed to identify patterns in successful recruitment efforts and codify effective strategies for recruiting SPs to ASIC.

**Results**

With a goal of recruiting 100 schools, ASIC reached out to 272 schools identified as candidates and ultimately, SPs at 114 schools were recruited over 6 months. The average SP required 5 contacts before agreeing to participate (range: 1-16). Following early low recruitment numbers, the study team mobilized seven clinicians and research assistants with mental health service experience, as well as members of a statewide CBT coaching network to reach out to schools. Leveraging these existing community partnerships served to significantly increase recruitment success, with average number of schools recruited increasing from 6/month prior to coach involvement to 39/month after. Further, discussion with SPs about their concerns regarding participation also proved helpful, as most were related to implementation of CBT in their work, rather than study participation. Discussing the experiences of past program participants, as well as program flexibility, helped assuage these concerns. Notably, SPs were generally not persuaded by discussion of study incentives ($330 over 18 months), but rather by empirical evidence related to student mental health improvement.

**Conclusions**

Engagement of community members and personalized recruitment efforts were necessary to overcome barriers to study participation among schools. This resulted in engaging SPs from diverse school settings and exceeding recruitment targets.

**References**

1. Kilbourne AM, Smith SN, Choi SY, Koschmann E, Liebrecht C, Rusch A, Abelson JL, Eisenberg D, Himle JA, Fitzgerald K, Almirall D. Adaptive school-based Implementation of CBT (ASIC): clustered-SMART for building an optimized adaptive implementation intervention to improve uptake of mental health interventions in schools. Implement Sci. 2018;13(1):119.

2. Weiner BJ. A theory of organizational readiness for change. Implement Sci. 2009;4.1:67.

3. Glanz K, Bishop DB. The role of behavioral science theory in development and implementation of public health interventions. Ann Rev Public health. 2010;31:399-418.

## A134 The role of outer context factors in the state-wide implementation of evidence-based practices for students with Autism Spectrum Disorder

### Allison Nahmias^1^, Melina Melgarejo^2^, Patricia Schetter^1^, Jessica Suhrheinrich^2^, Jennica Li^1^, Shaun Jackson^1^, Aubyn Stahmer^1^

#### ^1^UC Davis MIND Institute, Sacramento, CA, USA; ^2^San Diego State University, San Diego, CA, USA

##### **Correspondence:** Allison Nahmias (asnahmias@ucdavis.edu)

**Background**

Although evidence-based practices (EBPs) for children with Autism Spectrum Disorder (ASD) exist, there are significant challenges with implementing these interventions in community settings. The California Autism Professional Training and Information Network (CAPTAIN) is a statewide cross-agency implementation team with the goal of scaling up use of EBPs for ASD using train-the-trainer methodology. Data on model effectiveness and mechanisms of action are limited. This project investigates the role of outer context factors (organizational culture, leadership, structure, and resources) on implementation team member training performance using the EPIS [1] Framework.

**Materials and Methods**

101 directors from 87 Special Education Local Plan Areas (SELPAs, organizations that facilitate Special Education services in California) completed the Implementation Climate Scale (ICS), Implementation Leadership Scale (ILS), and ASD EBP Resource Assessment Tool. 194 CAPTAIN members reported on the frequency and quality of training and coaching. Generalized Estimating Equations were used to examine differences in implementation climate, leadership, and resources in regards to SELPA structure and size and their association with CAPTAIN member performance.

**Results**

ICS, ILS, and ASD EBP Resource scores varied by SELPA size. Large SELPA directors reported better implementation climate in regards to focus on EBPs and existing supports to deliver EBPs than small SELPAs, and better educational support for EBPs compared to medium SELPAs (p-values < .05). Large SELPA directors also reported higher proactive, supportive and perseverant implementation leadership than small SELPAs, and higher knowledgeable and proactive implementation leadership than medium SELPAs (p-values < .05). Large SELPAs also reported greater partnerships with community stakeholders related to ASD EBP use than small SELPAs (p = .01). ICS scores varied by whether the SELPA consisted of one school district (single) or multiple school districts (multi), with multi-district SELPAs reporting higher selection for EBPs and selection for openness than single-district SELPAs (p-values < .02). Proactive leadership was a significant predictor of CAPTAIN performance (B = 3.77, p = .04).

**Conclusions**

Implementation leadership and climate vary across organizations suggesting variability more broadly. Proactive leadership relates to frequency and quality of EBP training and coaching in schools. Matching targeted implementation efforts to context and organizational functioning will be discussed.

**Reference**

1. Aarons GA, Hurlburt M, Horwitz SM. Advancing a conceptual model of evidence-based practice implementation in public service sectors. Adm Policy Ment Health. 2010;38(1):4-23. doi:10.1007/s10488-010-0327-7.

## A135 Do student characteristics affect teachers’ decisions to use 1:1 instruction?

### Heather Nuske^1^, Melanie Pellecchia^1^, Viktor Lushin^1^, Keiran Rump^1^, Max Seidman^1^, Rachel Ouellette^2^, Diana Cooney^1^, Brenna Maddox^1^, Gwendolyn Lawson^1^, Amber Song^1^, Erica Reisinger^1^, David Mandell^1^

#### ^1^University of Pennsylvania, Philadelphia, PA, USA; ^2^Florida International University, Miami, FL, USA

##### **Correspondence:** Heather Nuske (hjnuske@upenn.edu)

**Background**

One-to-one instruction is a critical component of evidence-based practices (EBPs) for students with autism spectrum disorder (ASD) [1], but is not used as often as recommended. As described in the Consolidated Framework for Implementation Research [2], an important outer setting characteristic when considering EBPs is clients’ needs based on their characteristics. Indeed, student characteristics may affect teachers’ decisions to select a treatment and/or implement one-to-one instruction [3]. This study examined whether teachers’ reported use of one-to-one discrete trial training (DTT) and pivotal response training (PRT) was associated with students’ clinical and demographic characteristics.

**Materials and Methods**

Participants were kindergarten-through-second-grade autism support teachers (n=80) and children aged 5-9 years with ASD (n=228). All teachers received training and consultation in the EBPs, and rated children in several symptom domains using the Pervasive Developmental Disorders Behavior Inventory. Children were assessed on cognitive and language abilities using the Differential Abilities Scales, and on self-regulation difficulties using the Behavioral Interference Coding Scheme. Each month, teachers reported on their use of two EBPs with each student during the past week.

**Results**

Children’s higher sensory symptoms, lower social approach, lower verbal skills and higher self-regulation difficulties were associated with more frequent 1:1 DTT and PRT; significant child symptom domains each explained 8-15% of the variance in reported receipt of treatment. Children’s age, sex and race were not statistically significant predictors of children’s receipt of these EBPs.

**Conclusions**

These results provide an example of a situation where client characteristics seem to influence providers’ use of EBPs. More obviously impaired students received more of each EBP. The findings beg the questions of whether teachers are accurate in their decisions regarding who benefits from 1:1 instruction, and whether children should be matched to specific types of 1:1 instruction based on their clinical characteristics. To the extent that experts think that less obviously impaired students would benefit from 1:1 instruction, those working with teachers should address practical, attitudinal and structural barriers to providing 1:1 instruction to a larger proportion of students. This study highlights the importance of considering client characteristics in the development and study of implementation strategies, across EBPs and contexts.

**References**

1. Odom SL, Collet-Klingenberg L, Rogers SJ, Hatton DD. Evidence-based practices in interventions for children and youth with autism spectrum disorders. Prev Sch Fail. 2010;54(4):275–282.

2. Damschroder LJ, Aron DC. Keith RE, Kirsh SR, Alexander JA, Lowery JC. Fostering implementation of health services research findings into practice: a consolidated framework for advancing implementation science. Implement Sci. 2009; 4:50.

3. Stahmer AC, Collings NM, Palinkas LA. Early intervention practices for children with autism: Descriptions from community providers. Focus Autism Other Dev Disabl. 2005;20(2):66–79.

## A136 Graduate school training in structured cognitive behavioral therapy protocols predicts greater evidence based psychotherapy reach

### Jiyoung Song^1^, Hector Garcia^2^, Erin Finley^3^, Shannon Wiltsey Stirman^4^

#### ^1^National Center for PTSD, VA Palo Alto Health Care System, Palo Alto, CA, USA; ^2^VA Texas Valley Coastal Bend Health Care System, Harlingen, TX, USA; University of Texas Health Science Center San Antonio, San Antonio, TX, USA; ^4^Stanford University, Stanford, CA, USA

##### **Correspondence:** Jiyoung Song (sjiyoung@sas.upenn.edu)

**Background**

The Veterans Health Administration recommends that patients with PTSD receive either of the two evidence-based psychotherapies (EBPs), Cognitive Processing Therapy (CPT) and Prolonged Exposure (PE). However, in one survey, clinicians who completed the national PE training program reported that they only treated one or two patients at a time with PE [1]. In another survey, 69% of the clinicians who were trained in CPT provided CPT “rarely” or “less than half the time” [2]. Because underutiliziation of the EBPs leads to fewer patients receiving the optimal treatments, it is important to identify and remediate clinician-level barriers that lead to low reach.

**Materials and Methods**

In our current study, we surveyed clinicians across the United States who primarily work on PTSD clinical teams (PCTs; [3]). They reported whether they received graduate school training (GST) in structured cognitive behavioral therapy (CBT) protocols and rated agreement to the following clinician-level barriers to EBPs: discomfort of exposing patients to distress during PE and concerns of patients’ difficulty understanding CPT. We conducted mediation analyses with bootstrapping between GST, EBP barriers, and EBP usage.

**Results**

We found that GST (b = -0.27, t(222) = -2.08, p = .04) led to lower discomfort of exposing patients to distress during PE, and in turn, lower discomfort (b = -6.84, t(222) = -4.83, p < .001) predicted greater PE usage. GST and PE usage had a significant average causal mediation effect (b = 1.83, 95% CI [0.00, 4.10], p = .05). Because GST (b = 0.13, t(222) = 0.82, p = .41) was not significantly associated with the CPT barrier, there was no statistical ground to test for the mediation between GST, CPT barrier, and CPT usage.

**Conclusions**

Our results indicate that clinicians who used structured CBT protocols as part of their graduate school training are less likely to shy away from an EBP even when it might ask them of discomfort of exposing their patients to distress. To increase EBP reach, policy makers should promote for the inclusion of using structured CBT protocols in graduate school curriculums.

**References**

1. Ruzek JI, Eftekhari A, Crowley J, Kuhn E, Karlin BE, Rosen CS. Post-training beliefs, intentions, and use of prolonged exposure therapy by clinicians in the Veterans Health Administration. Adm Policy Ment Health. 2017;44(1):123-132.

2. Chard KM. Cognitive processing therapy train-the-trainer workshop. Cincinnati, OH. 2014.

3. Garcia HA, DeBeer BR, Mignogna J, Finley EP. Treatments Veterans Health Administration PTSD specialty program providers report their patients prefer: The role of training and theoretical orientation. Psychol Trauma. 2019;11(8):837–841. https://doi.org/10.1037/tra0000442

## A137 Creating academic and organizational synergy within public education to support statewide scale up of EBP for students with Autism Spectrum Disorder

### Jessica Suhrheinrich^1,2^, Patricia Schetter^3,4^, Ann England^4,5^, Melina Melgarejo^1^, Allison Nahmias^3^, Aubyn Stahmer^3^

#### ^1^San Diego State University, San Diego, CA, USA; ^2^Child and Adolescent Services Research Center San Diego, CA, USA; ^3^UC Davis MIND Institute, Sacramento, CA, USA; ^4^California Autism Professional Training and Information Network, CA, USA; ^5^CA Department of Education, Sacramento, CA, USA

##### **Correspondence:** Jessica Suhrheinrich (jsuhrheinrich@sdsu.edu)

**Background**

Nationwide, 616,234 students were served for Autism Spectrum Disorder (ASD) during 2017-18, an increase of 55% from 2007-08 [1]. Although evidence-based practices (EBPs) for individuals with ASD exist [2], use in community settings is limited. The California Autism Professional Training and Information Network (CAPTAIN) is a statewide cross-agency collaboration with the goal of scaling up use of EBPs for ASD. CAPTAIN has over 400 members representing 140 school and community agencies who commit to training, coaching and engaging in collaboration.

**Methods and Materials**

CAPTAIN began as a clinical initiative 6 years ago, then further developed under the influence of implementation science methodology. The Exploration, Planning, Implementation and Sustainment framework (EPIS) [3] has impacted targeted strategy use for statewide scale up of EBPs by informing the development of key partnerships, implementation goals, and collaborative processes within CAPTAIN.

**Results**

The panel will highlight how education policy and implementation data have influenced CAPTAIN practices and procedures within a community-academic partnership. These factors will be presented by purveyors, intermediaries and implementation science researchers. The founding co-coordinator of CAPTAIN and a purveyor of this initiative will share information about the policies that have influenced the development of the network and how key partnerships have been formed. A fellow founding co-coordinator of CAPTAIN that serves as an intermediary in the CAPTAIN project will share how implementation science and the EPIS Model have informed the CAPTAIN implementation goals and procedures and how this information has been shared with state and local agencies through continuous improvement cycles. The research director for CAPTAIN will present current funded research initiatives involving CAPTAIN and will facilitate discussion.

**Conclusions**

Mixed-methods data will be presented, informed by an internal survey of CAPTAIN members (n=414), a statewide survey of educational professionals and administrators (n=1700), and qualitative focus group outcomes from providers and CAPTAIN members (n=30).

A subset of outcomes will be presented with a focus on multiple perspectives on barriers, implementation leadership and implementation climate, implementation strategy use, and the distribution of decision making across organizational levels.

**References**

1. U.S. Department of Education, EDFacts Data Warehouse (EDW): IDEA Part B Child Count and Educational Environments Collection, file specifications 002 and 089. 2017-2018. https://www2.ed.gov/ programs/osepidea/618-data/static-tables/2017-2018/part-b/child-count-and-educational-environment/1718-bchildcountandedenvironment-18.xlsx. Accessed 11 July 2018

2. Wong C, Odom SL, Hume KA, Cox AW, Fettig A, Kucharczyk S, Brock ME, Plavnick JB, Fleury VP, Schultz TR. Evidence-based practices for children, youth, and young adults with autism spectrum disorder: a comprehensive review. J Autism Dev Disord. 2015;45(7):1951-1966. doi:10.1007/s10803-014-2351-z.

3. Aarons GA, Hurlburt M, Horwitz SM. Advancing a conceptual model of evidence-based practice implementation in public service sectors. Adm Policy Ment Health. 2010;38(1):4-23. doi:10.1007/s10488-010-0327-7.

## A138 Meta-analysis of implementation strategy effectiveness on general education teacher adherence to evidence-based practices

### James Merle, Clayton Cook, Andrew Thayer, Madeline Larson, Sydney Pauling, Jenna McGinnis

#### University of Minnesota, Minneapolis, MN, USA

##### **Correspondence:** James Merle (merle016@umn.edu)

**Background**

Intervention research in education science has produced a plethora of evidence-based practices (EBP) to improve student social, emotional, and behavioral (SEB) outcomes [1]. To ensure students benefit from these practices, implementation strategies (techniques and methods to improve implementation outcomes) [2], have been developed to bolster school-based practitioners’ EBP treatment integrity. These include action planning, prompts and reminders, coaching, and performance feedback [3-6]. However, because these strategies are often delivered simultaneously, mechanisms by which they produce effects remains unknown, hindering further understanding of causal relationships and efficient service delivery. Therefore, the purpose of this study was to conduct a meta-analysis categorizing and analyzing the effectiveness of discrete school-based implementation strategies across service provision levels to inform limited school resource allocation and identify future directions for research.

**Materials and Methods**

Published studies of strategies used to increase teacher implementation of SEB EBPs were included. Effect sizes were calculated, and a robust variance estimation meta-regression model (RVE) was used to hierarchically analyze average effects and conduct moderator analyses [7]. Funnel plots and Egger’s test were used to assess publication bias [8].

**Results**

Preliminary results of 31 single-subject studies indicate that active-implementation strategies targeting performance deficits were effective overall for increasing teacher adherence to EBPs above baseline and pre-implementation training alone (Hedge’s g = 2.45). Maintenance strategies, such as dynamic fading of supports, while sparse, indicated effective sustainment of implementation behavior (g = 0.8). Performance-feedback was the most common strategy (n = 23; 74%), though preliminary results indicate it may not be the most effective strategy across all intervention tiers. Twenty group-design studies were included and analyses are underway.

**Conclusions**

Teacher treatment integrity improved with added supports; however, once supports were removed, implementation decreased in over 40% of the studies that collected follow-up data. As is true in the greater implementation literature, [9], further methods for sustaining implementation are needed. Gradual fading reduced implementer drift, and practitioners should consider it with active-implementation supports. This study contributes to the broader implementation literature by providing discrete strategy effectiveness of practitioner-level performance-based implementation strategies and informs future research categorizing and analyzing implementation strategies within existing frameworks [2].

**References**

1. Owens JS, Lyon AR, Brandt NE, Warner CM, Nadeem E, Spiel C, Wagner M. Implementation science in school mental health: key constructs in a developing research agenda. School Ment Health. 2014;6(2):99-111.

2. Powell BJ, Waltz TJ, Chinman MJ, Damschroder LJ, Smith JL, Matthieu MM, Proctor EK, Kirchner JE. A refined compilation of implementation strategies: results from the Expert Recommendations for Implementing Change (ERIC) project. Implement Sci. 2015;10(1):21. doi:10.1186/s13012-015-0209-1.

3. Bethune KS. Effects of coaching on teachers’ implementation of tier 1 school-wide positive behavioral interventions and support strategies. J Posit Behav Interv. 2017;19(3):131-142.

4. Collier-Meek MA, Fallon LM, Defouw ER. Toward feasible implementation support: E-mailed prompts to promote teachers’ treatment integrity. School Psychol Rev. 2017;46:379-394.

5. Collier-Meek MA, Sanetti LM, Boyle AM. Providing feasible implementation support: direct training and implementation planning in consultation. School Psychol Forum. 2016;10.1:106-119.

6. Sanetti LMH, Collier-Meek MA, Long ACJ, Byron J, Kratochwill TR. Increasing teacher treatment integrity of behavior support plans through consultation and implementation planning. J School Psychol. 2015;53: 209-229.

7. Hedges LV, Tipton E Johnson MC. Robust variance estimation in meta‐regression with dependent effect size estimates. Res Synth Method. 2010;1:39-65. doi:10.1002/jrsm.5

8. Egger M, Davey Smith G, Schneider M, Minder, C. Bias in meta-analysis detected by a simple, graphical test. BMJ. 1997;315(7109):629-34.

9. Moullin JC, Dickson KS, Stadnick NA, Rabin B, Aarons GA. Systematic review of the Exploration, Preparation, Implementation, Sustainment (EPIS) framework. Implement Sci. 2019; 14:1.

## A139 Ready, willing, and able? exploring education researcher engagement in dissemination

### Taylor Koriakin, Sandra Chafouleas, Lisa Sanetti, Jennifer Dineen

#### University of Connecticut, Storrs, CT, USA

##### **Correspondence:** Taylor Koriakin (taylor.koriakin@uconn.edu)

**Background**

Getting Research into Policy and Practice [1] (GRIPP) is a continued concern in education with several studies citing limited knowledge and use of evidence-based practices (EBP) by school practitioners [2, 3]. Although studies exist evaluating practitioners’ perspectives on GRIPP [4], there are presently no studies examining researchers’ approaches to dissemination. Given that they generate the evidence-base for school-based practice, an understanding of how researchers communicate their findings is an important piece in understanding implementation of EBPs. The purpose of the present study was to understand education researchers’ engagement in dissemination activities targeting non-research audiences.

**Materials and Methods**

School psychology and special education researchers (n = 226) at Research Intensive institutions completed on online survey related to their dissemination practices. Respondents answered items pertaining to their most frequently used dissemination modalities, target audiences, barriers, and time dedicated to dissemination. Participants were also asked to rank which dissemination activities they perceived as having the greatest impact on education practice.

**Results**

Over half of sample (59.9%) reported spending less than two hours per week on dissemination activities targeting non-research audiences. Participants were asked to rank order their most frequently used dissemination practices and indicated that academic journal articles (rated as primary dissemination activity by 63% of sample) and conference presentations (rated primary activity by 15%) were the most frequently used modalities for dissemination. Although participants reported that they felt that professional development sessions, meetings with stakeholders, and practitioner-focused books have the greatest impact on educational practice, they were not as frequently utilized by respondents as peer-reviewed articles and conference sessions. Common barriers reported by respondents were limited time to dedicate to dissemination (rated as primary barrier by 37% of sample) and that dissemination is a low priority at institutions (rated as primary barrier by 20%).

**Conclusions**

Overall, respondents reported engaging in low rates of dissemination targeting applied, non-research audiences. With limited time, education researchers appear to focus their resources on activities associated with promotion and tenure (e.g., publishing peer-reviewed journal articles). Although participants valued the importance of dissemination targeting those in applied settings, they reported that these activities are largely not valued by their institutions.

**References**

1. Hoover SA. When we know better, we don’t always do better: facilitating the research to practice and policy gap in school mental health. Sch Ment Health. 2018;10(2):190-198.

2. Burns MK, Ysseldyke JE. Reported prevalence of evidence-based instructional practices in special education. J Spec Educ. 2009;43:3-11.

3. Stormont M, Reinke W, Herman K. Teachers’ characteristics and ratings for evidence-based behavioral interventions. Behav Disorders. 2011;37:19-29.

4. Dagenais C, Lysenko L, Abrami PC, Bernard RM, Ramde J, Janosz M. Use of research-based information by school practitioners and determinants of use: a review of empirical research. Evid Policy. 2012;8:285-309.

## A140 Extending implementation science to the rural school setting

### Benjamin Ingman^1,2^, Elaine Belansky^1,2^

#### ^1^Center for Rural School Health & Education, Denver, CO, USA; ^2^University of Denver, Denver, CO, USA

##### **Correspondence:** Benjamin Ingman (benjamin.ingman@du.edu)

**Background**

Children in rural America face unique health disparities compared to their urban counterparts, including higher substance use; sexual activity and teen pregnancy; and suicide rates [1-3]. While there are numerous evidence-based practices (EBPs) K-12 schools can implement to promote students’ physical, social-emotional, and academic well-being [4-11], little is known about the contextual factors that facilitate or inhibit rural school districts’ selection of EBPs.

Our center is currently facilitating 21 high-poverty, rural school districts through a strategic planning process to create comprehensive health and wellness plans that include EBPs for physical and mental health. The process is completed by a school district task force comprised of school administrators, teachers, students, parents, and community members under the guidance of an external facilitator.

In this study, we adapted the Consolidated Framework for Implementation Research (CFIR)[12] to the rural school context to understand the contextual factors associated with school districts’ selection of EBPs. We explore (1) the EBPs selected by these rural school districts, (2) the prevalence of contextual factors amongst districts, and (3) the association of contextual factors with EBPs selected.

**Materials and Methods**

We have developed (and are currently administering) an 81 item survey to evaluate the contextual factors of implementation for this initiative. This pen and paper survey, which was designed for rural schools completing the planning process, accounts for all 39 CFIR constructs. The survey is administered at the end of the final meeting of the process and completed by members of the 21 rural school district task forces. All survey data will be collected by May 2019.

**Results**

Results will reveal the prevalence and relationships of contextual factors and EBPs selected by rural school districts as a result of the process.

**Conclusions**

These results will be instructive concerning the importance of particular contextual factors as they apply to the selection of EBPs in rural schools. Additionally, the survey developed and administered provides an example of evaluating CFIR constructs in K-12 school settings, which contributes to the growing SIRC instrument review project.

**References**

1. Atav S, Spencer G. Health risk behaviors among adolescents attending rural, suburban, and urban schools: a comparative study. Fam Community Health. 2002;25:53-64.

2. Lambert D, Gale JA, Hartley D. Substance abuse by youth and young adults in rural America. J Rural Health. 2008; 24:221-8.

3. Health Management Associates. Community conversations to inform youth suicide prevention. 2018: https://coag.gov/sites/default/files/final_youth_suicide_in_colorado_report_10.01.18.pdf. Accessed 18 March 2019.

4. Botvin GJ, Griffin KW. Life skills training: Empirical findings and future directions. J Prim Prev. 2004; 25:211-232.

5. Caldarella P, Shatzer RH, Gray KM, Young KR, Young EL. The effects of school-wide positive behavior support on middle school climate and student outcomes. Res Mid-Level Educ. 2011;35:1-14.

6. Celio CI, Durlak J, Dymnicki A. A meta-analysis of the impact of service-learning on students. J Experiential Education. 2011;34:164-181.

7. Durant N, Harris SK, Doyle S, Person S, Saelens BE, Kerr J, Norman GJ, Sallis JF. Relation of school environment and policy to adolescent physical activity. J Sch Health. 2009;79:153-159.

8. Evans A, Ranjit N, Rutledge R, Medina J, Jennings R, Smiley A, Hoelscher D. Exposure to multiple components of a garden-based intervention for middle school students increases fruit and vegetable consumption. Health Promot Pract. 2012;13:608-616.

9. Hadlaczky G, Hökby S, Mkrtchian A, Carli V, Wasserman D. Mental Health First Aid is an effective public health intervention for improving knowledge, attitudes, and behaviour: a meta-analysis. Int Rev Psychiatry. 2014;26:467-475.

10. Kelly CM, Mithen JM, Fischer JA, Kitchener BA, Jorm AF, Lowe A, Scanlan C. Youth mental health first aid: a description of the program and an initial evaluation. Int J Ment Health Syst. 2011;5:1-9.

11. Kirby D, Laris BA. Effective curriculum based sex and STD/HIV education programs for adolescents. Child Dev Perspect. 2009;3:21-29.

12. Damschroder LJ, Aron DC. Keith RE, Kirsh SR, Alexander JA, Lowery JC. Fostering implementation of health services research findings into practice: a consolidated framework for advancing implementation science. Implement Sci. 2009; 4:50.

## A141 Community mental health providers’ use of parent training with Medicaid-enrolled families of children with autism

### Diondra Straiton, Brooke Ingersoll

#### Michigan State University, East Lansing, MI, USA

##### **Correspondence:** Diondra Straiton (straiton@msu.edu)

**Background**

Evidence-based parent training, in which providers systematically train parents to implement specific intervention strategies with their child, is an underutilized treatment for children with autism spectrum disorder (ASD) [1]. In 2012, Michigan passed the Medicaid Autism Benefit for Behavioral Health Treatment, which provides funds for Medicaid-enrolled children with ASD for applied behavior analysis (ABA) services, including parent training. However, a review of billing data shows that few parent training sessions have been billed under the Medicaid Autism Benefit, despite providers having the ability to bill for the service at a high reimbursement rate. To our knowledge, no other study to date has examined reasons for why parent training for ASD is underutilized within community mental health settings.

**Materials and Methods**

This mixed-methods project examined the use of parent training during ABA under the Michigan Medicaid Autism Benefit for children with ASD under age 21. Descriptive statistics and multiple regression were used to analyze Medicaid claims data for 879 children and survey data from 97 ABA providers. Content analysis was used to analyze open-ended survey items and thematic analysis was used to analyze interviews from a subset of 13 providers.

**Results**

Results demonstrated that: a) frequency of parent training encounters was very low, b) ABA providers’ conceptualization of parent training is inconsistent with the literature; c) providers report using evidence-based parent training strategies at a moderate-to-high level on the survey, but infrequently spontaneously mention those strategies in interviews; d) providers use sessions for other purposes; e) providers report having limited related training experiences; f) and providers report numerous barriers and facilitators which are related to their reported extensiveness of parent training.

**Conclusions**

Barriers and facilitators were identified at the family-, provider-, and organization-levels and map well onto the Consolidated Framework for Implementation Research, including outer setting (policy regarding Medicaid Autism Benefit), inner setting (agency-level characteristics), and the individuals involved (providers). Results from this study will be used to design an intervention to increase uptake of evidence-based parent training in this system.

**Reference**

1. Hume K, Bellini S, Pratt C. The Usage and perceived outcomes of early intervention and early childhood programs for young children with Autism Spectrum Disorder. Top Early Child Spec Educ. 2005;25(4):195-207. doi:10.1177/ 02711214050250040101

## A142 A multi-component home visitation program to prevent child maltreatment: effects on parenting and child functioning

### Elizabeth Demeusy, Jody Manly, Robin Sturm, Sheree Toth

#### Mt. Hope Family Center, University of Rochester, Rochester, NY, USA

##### **Correspondence:** Elizabeth Demeusy (elizabeth.demeusy@rochester.edu)

**Background**

No single approach can meet the multi-dimensional needs of impoverished, high-risk families. With this in mind, the Building Healthy Children (BHC) intervention program was designed as a collaborative community initiative to prevent child maltreatment and support healthy development in newborns of young mothers [1-2]. This inter-agency collaborative was comprised of medical, university and community partners, with a strong focus exporting evidence-based models and translating research to practice. BHC was designed as a home visitation program that employed a tiered model of service delivery based on families’ individual needs, in order to effectively and efficiently deliver outreach and evidence-based treatment models. These treatment models addressed parenting (Parents as Teachers), attachment (Child-Parent Psychotherapy), and maternal depression (Interpersonal Psychotherapy for Depression), and each has received substantial evidentiary support [3-6].

**Materials and Methods**

The current study utilizes a longitudinal follow-up design, to examine the effects of BHC on parenting and child behavior once the child is in elementary school (6-10 years old). The anticipated sample size is 100 children (45% male) and their biological mothers (baseline age, M=19). In addition, data is also being collected from the child’s teacher regarding child behavior in the classroom in order to provide a multi-informant perspective across settings. Thus far, data has been collected from approximately 50 families. Data collection is scheduled to conclude by 7/1/19.

**Results**

This presentation will examine the long-term effects of BHC on child maltreatment, out-of-home placement and harsh and inconsistent parenting, as well as positive parenting practices. In addition, it will examine the effects of BHC on child externalizing behavior and self-regulation.

**Conclusions**

The information gleaned from this study will help us to better understand how to develop effective inter-agency partnerships in order to increase accessibility to high-quality, evidence-based mental health services for vulnerable children and families. This effectiveness study provides information about implementing efficacious interventions within existing community infrastructure. Finally, the results of this study will help us to better understand how to prevent violence against children, and to foster a safe and healthy caregiving environment for children to grow and thrive.

**References**

1. Paradis HA, Sandler M, Manly JT, Valentine L. Building healthy children: Evidence-based home visitation integrated with pediatric medical homes. Pediatrics. 2013;132(Suppl 2):S174-S179.

2. Toth SL, Manly JT. Bridging research and practice: challenges and successes in implementing evidence-based preventive intervention strategies for child maltreatment. Child Abuse Negl. 2011;35(8):633-636.

3. Cicchetti D, Rogosch FA, Toth SL. Fostering secure attachment in infants in maltreating families through preventive interventions. Dev Psychopathol. 2006;18(03):623-649.

4. Toth SL, Rogosch FA, Oshri A, Gravener-Davis J, Sturm R, Morgan-López AA. The efficacy of interpersonal psychotherapy for depression among economically disadvantaged mothers. Dev Psychopathol. 2013;25(4pt1):1065-1078.

5. Wagner MM, Clayton SL. The Parents as Teachers program: results from two demonstrations. Future Child. 1999; 9(1):91-115.

6. Weissman MM, Markowitz JC, Klerman GL. Comprehensive guide to interpersonal psychotherapy. New York: Basic Books; 2000.

## A143 Screening for perinatal depression and anxiety: the Appalachian provider’s perspective on current practice and barriers

### Mira Snider, Shari Steinman

#### West Virginia University, Morgantown, WV, USA

##### **Correspondence:** Mira Snider (mdh0054@mix.wvu.edu)

**Background**

Postpartum Anxiety (PPA) and Postpartum Depression (PPD) are common mental health issues that go largely undiagnosed and untreated in the United States [1]. There are no federal policies requiring screening of new mothers, and only thirteen states have passed legislature or convened task forces to promote screening for PPD in perinatal care. Similar efforts have not been made to promote PPA screening [2]. Although screening is recognized as a valuable practice that may increase new mothers’ access to mental health care, there is limited data available on how this legislature has translated to real-world screening and referral practice. The current study examines current screening and referral practices of perinatal providers in West Virginia -- a state that has taken legislative action to promote depression screening in perinatal women. Barriers to screening and referral that could be targeted by future implementation or advocacy efforts are also explored.

**Materials and Methods**

Approximately 50 perinatal providers in urban and rural settings across West Virginia will be recruited to complete an online survey. Current screening and referral practices for PPA and PPD, perspectives on best screening practices, and barriers to discussing mental health issues with patients will be assessed. Providers’ perceived feasibility, acceptability, norms, and intention to use screening tools or referrals with perinatal women will also be examined.

**Results**

Data collection for the current study is ongoing. Preliminary analyses on a small subset of providers have indicated that obstetrician/gynecologists in urban clinics screen a majority of patients for anxiety and depression symptoms, but providers do not consistently conduct screening at each visit. Approximately 4-5% of providers’ caseloads were referred for mental health care in the past year, and referral rates were higher for patients endorsing depression symptoms compared to patients endorsing anxiety symptoms. Providers reported limited time as a barrier to screening, and a lack of consistent screening as a barrier to providing mental health referrals to women.

**Conclusions**

Perinatal providers in West Virginia screen for depression and anxiety; however, screening and referral may be inconsistent across disorders. Having limited time to screen patients may interfere with mandated screening in perinatal care.

**References**

1. Accortt EE, Wong MS. It is time for routine screening for perinatal mood and anxiety disorders in obstetrics and gynecology settings. Obstet Gynecol Surv. 2017;72(9):553-568.

2. Rowan PJ, Duckett SA, Wang JE. State mandates regarding postpartum depression. Psychiatr Servic. 2015; 66(3):324-328.

## A144 Factors influencing implementation of the Alma program: structured peer mentoring for depressed perinatal Spanish-speaking women

### Rachel Vanderkruik, Sona Dimidjian, Caitlin McKimmy

#### CU Boulder, Boulder, CO, USA

##### **Correspondence:** Rachel Vanderkruik (rachel.vanderkruik@colorado.edu)

**Background**

To address a critical gap in resources for depressed Latina pregnant and early parenting women in rural settings, we co-designed and evaluated the Alma Program utilizing an innovative model of “task-sharing” [1], where peers are trained to mentor perinatal women experiencing depression. In-depth examination of the extent to which contextual factors influence implementation outcomes has the potential to advance understanding of the relationships among contextual adaptation, success of a program, and dissemination to new settings.

**Materials and Methods**

We conducted a case study of the Alma program to identify the organizational, contextual, and cultural factors that served as barriers or facilitators to the implementation of the Alma program within a rural Colorado community. Key informants who participated in the case study included staff and administrators from partner organizations, members of the research team, local community members, and Alma peer mentors. All key informants (N=15) completed a survey that assessed the organizational and contextual factors known to influence the implementation of programs, developed using the Practical, Robust, Implementation and Sustainability Model (PRISM) [2]. All key informants were given the opportunity to participate in individual interviews or focus groups to explore qualitatively factors associated with the successful implementation of Alma, and to follow-up on findings from the survey.

**Results**

We will identify key facilitators and barriers that were identified at the following contextual levels: broader community/environment, organization, program, participant. For example, at the broader community/environment level, the most commonly reported facilitator for Alma program implementation (92.3% of the sample) was, “a need for mental health resources in the community and the most commonly reported barrier (38.5% of the sample) was, “poor communication streams in the community.”

**Conclusions**

The organizational and program characteristics were identified to be overall key facilitators for implementation of the Alma program in this setting, whereas the broader community and environment context was identified to be an overall barrier. Specific barriers and facilitators within each of these levels are identified, which could inform broader implementation and spread of the program to new sites.

**References**

1. Chowdhary N, Sikander S, Atif N, Singh N, Ahmad I, Fuhr DC, Rahman A, Patel V. The content and delivery of psychological interventions for perinatal depression by non-specialist health workers in low and middle income countries: a systematic review. Best Pract Res Clin Obstet Gynaecol. 2014;28(1):113-33.

2. Feldstein AC, Glasgow RE. A practical, robust implementation and sustainability model (PRISM) for integrating research findings into practice. Jt Comm J Qual Patient Saf. 2008;34:228–43.

## A145 Fidelity to motivational interviewing and caregiver engagement in the Family Check-Up 4 Health Program: longitudinal associations in a hybrid effectiveness-implementation trial

### Cady Berkel^1^, JD Smith^2^, Anne Mauricio^1^, Jenna Rudo-Stern^1^, Liz Alonso^1^, Sara Jimmerson^1^, Lizette Trejo^1^

#### ^1^Arizona State University, Tempe, AZ, USA; ^2^Northwestern University Feinberg School of Medicine, Chicago, IL, USA

##### **Correspondence:** Cady Berkel (cady.berkel@asu.edu)

**Background**

This study examines fidelity to the Family Check-Up 4 Health (FCU4Health) in a randomized hybrid trial evaluating effects on the prevention of excess weight gain in pediatric primary care [1]. The program includes a comprehensive assessment, followed by a feedback and motivation session, in which Motivational Interviewing (MI) is used to engage families in follow-up parenting modules and community-based programs to address social determinants of health. The COACH observational rating system assesses fidelity to the FCU4Health. In a prior trial, COACH ratings have been associated with higher concurrent ratings of parent engagement, and in turn, longitudinal improvements in parenting and child behaviors [2]. Further, coordinators with more training had better MI skills, and this was positively linked with parent engagement [3] suggesting that MI skills are a core component of change. This study extends prior work to advance the science of fidelity assessment when implementing a preventive parenting program.

**Materials and Methods**

The trial includes families (n=240) with children ages 6-12 years identified in pediatric primary care with elevated body mass index (BMI) for age and gender (≥85th percentile). 68% of parents were Latino and 38% spoke Spanish. After completing the baseline assessment, families were randomized to the FCU4Health program (n=140) or usual care (n=100). The FCU4Health involved 3 feedback sessions, with parenting support sessions and referrals to community programs (e.g., nutrition, physical activity, social services, health/mental health programs), over a 6-month period.

**Results**

COACH coding of this sample is nearly complete. Analyses will be completed in July 2019. We first plan to examine the temporal associations between the FCU4Health coordinator’s use of MI in the 3 feedback sessions with caregiver engagement over time using a cross-lagged panel model. Second, using multiple regression, we will examine associations between ratings of MI skills with families’ engagement in community-based programs, accounting for salient family demographic factors such as income, insurance, parent health, and acculturation markers.

**Conclusions**

This study will inform the ways in which fidelity to MI is associated with behavior change in an evidence-based parenting program—adding valuable knowledge to the field that has implications for fidelity monitoring, coordinator training, and program development.

**Trial Registration** ClinicalTrials.gov NCT03013309

**References**

1. Smith JD, Berkel C, Jordan N, Atkins DC, Narayanan SS, Gallo C, Grimm KJ, Dishion TJ, Mauricio AM, Rudo-Stern J, Meachum MK, Winslow E, Bruening MM. An individually tailored family-centered intervention for pediatric obesity in primary care: study protocol of a randomized type II hybrid implementation-effectiveness trial (Raising Healthy Children study). Implement Sci. 2018;13(11):1–15. doi:10.1186/s13012-017-0697-2

2. Smith JD, Dishion TJ, Shaw DS, Wilson MN. Indirect effects of fidelity to the Family Check-Up on changes in parenting and early childhood problem behaviors. J Consult Clin Psychol. 2013;81(6):962–974. doi:10.1037/a0033950

3. Smith JD, Rudo-Stern J, Dishion TJ, Stormshak EA, Montag S, Brown K, Ramos K, Shaw DS, Wilson MN. Effectiveness and efficiency of observationally assessing fidelity to a family-centered child intervention: a quasi-experimental study. J Clin Child Adolesc Psychol. 2019;48(1):16-28. doi:10.1080/15374416. 2018.1561295

## A146 Disseminating evidence-based treatments: teen psychopathology and treatment history moderate caregiver perceptions of EBT

### Margaret Crane^1^, Sarah Helseth^2^, Kelli Scott^2^, Sara Becker^2^

#### ^1^Temple University, Philadelphia, PA, USA; ^2^Brown University, Providence, RI, USA

##### **Correspondence:** Margaret Crane (margaret.crane@temple.edu)

**Background**

To effectively promote evidence-based practice (EBP), it is important to consider whether caregivers understand and view the concept favorably. Previous research found that caregivers with lower education, with lower SES, and from a minority racial group were less likely to correctly define EBP and had a less favorable view of EBP [1]. This study examined how caregivers define, value, and prefer to describe EBP, and how responses varied based on caregiver and teen psychopathology and treatment history.

**Materials and Methods**

Caregivers (N=411; 86% female; 88% non-Hispanic Caucasian) concerned about their teen’s (age 12-19) substance use completed an online survey as part of a larger study. Caregivers selected the correct definition of EBP, indicated their preference for describing EBP, and indicated whether they valued EBP treatment principles (proven vs. individualized treatment; treatment process vs. treatment outcome). Chi-square analyses evaluated caregiver responses by caregiver and teen treatment history, and teen mental health and substance use problems. Multivariate logistic regressions examined which variables were associated with the greatest likelihood of response selection.

**Results**

Most caregivers correctly defined EBP, preferred the concept of “therapy based on evidence”, preferred proven over individualized treatment, and valued the outcome over the process of therapy. Caregivers who had received mental health therapy were more likely to correctly define EBP (p<.01), and those whose teens had received mental health therapy less strongly valued the outcome over the process of therapy (p<.01). Multivariate analyses revealed that having a teens with legal problems and substance use problems significantly influenced how strongly caregivers preferred the term “therapy based on evidence”. Caregivers whose teens had internalizing problems and legal problems less strongly favored proven treatment over an individualized approach (ps<.01); only teen legal problems was significant in the multivariate analysis.

**Conclusions**

Although most caregivers correctly defined and valued EBP, caregiver and teen treatment history and teen psychopathology moderated this effect. Most notably, caregivers of teens with psychopathology symptoms and a therapy history were less likely to value principles of EBP. Caregivers of teens with substance use and legal problems also tended to prefer other terms over EBP.

**References**

1. Becker SJ, Weeks BJ, Escobar KI, Moreno O, DeMarco CR, & Gresko SA. Impressions of “Evidence-Based Practice”: a direct-to-consumer survey of caregivers concerned about adolescent substance use. Evid Based Pract Child Adolesc Ment Health. 2018; 3: 70–80. doi:10.1080/23794925.2018.1429228

## A147 Implementing a child mental health intervention in child welfare: CW staff perspectives on feasibility and acceptability

### Geetha Gopalan^1^, Kerry-Ann Lee^2^, Tricia Stephens^1,3^, Mary Acri^4^, Cole Hooley^5^, Caterina Pisciotta^4^

#### ^1^Hunter College, New York City, NY, USA; ^2^University of Maryland, College Park, MD, USA; ^3^City University of New York, New York City, NY, USA; ^4^New York University School of Medicine, New York City, NY, USA; ^5^Brown School, Washington University, St. Louis, MO, USA

##### **Correspondence:** Geetha Gopalan (ggopalan@hunter.cuny.edu)

**Background**

Children with behavior difficulties reared by child welfare (CW)-involved families often fail to receive needed mental health (MH) treatment due to limited service capacity and chronic engagement difficulties [1]. Task-shifting strategies (World Health Organization, 2008) can increase MH treatment access by relocating treatment to alternative service platforms (e.g., CW service settings) and utilizing a non-specialized workforce (e.g., CW caseworkers) to deliver MH interventions while supervised by trained clinicians. In this study, an evidence-based, multiple family group intervention (The 4Rs and 2Ss Program for Strengthening Families [4R2S]; [2-4]) to reduce child disruptive behavioral difficulties was modified so that it could be delivered by CW caseworkers without advanced MH training. This study examines the degree to which CW staff perceived delivering 4R2S in CW placement prevention services as feasible and acceptable, as well as those factors reported to facilitate or hinder feasibility and acceptability.

**Materials and Methods**

This mixed methods study collected quantitative and qualitative data from caseworkers (n=6), supervisors (n=4) and administrators (n=2). Quantitative and qualitative data focused on feasibility (e.g., participant flow, treatment fidelity, treatment attendance, CW staff perspectives on feasibility and appropriateness) and acceptability (CW staff perspectives on acceptability and attitudes towards evidence-based practices). Descriptive statistics compared quantitative data to pre-determined study benchmarks for high feasibility and acceptability. Qualitative data were coded into relevant a priori (feasibility, acceptability) and emergent themes. Mixed methods analytic strategies focused on integration at the analysis stage, comparing quantitative and qualitative findings side-by-side to identify points of convergence or expansion.

**Results**

Results indicate that CW staff perceived implementing a modified version of the 4R2S in placement prevention services as generally feasible and acceptable, with some exceptions (e.g., inconsistent family attendance). Factors facilitating feasibility and acceptability include agency and logistical support, provider characteristics, 4R2S content & strength-based focus, 4R2S ease of use, and perceived benefits for families and providers. Barriers to feasibility and acceptability included family characteristics, eligibility/recruitment procedures, logistical challenges, as well as issues with external supervision.

**Conclusions**

Findings from these key stakeholders can inform similar efforts to implement child MH EBPs within CW services.

**References**

1. Gopalan G, Chacko A, Franco LM, Dean-Assael KM, Rotko L, Marcus SM, Hoagwood KE, McKay M. Multiple Family Group service delivery model for youth with disruptive behaviors: child outcomes at 6-month follow-up. J Child Fam Stud. 2015;24(9):2721-2733.

2. Chacko A, Gopalan G, Franco L, Dean-Assael K, Jackson J, Marcus S, Hoagwood K, McKay M. Multiple Family Group service model for children with disruptive behavior disorders: child outcomes at post-treatment. J Emot Behav Disord. 2015;23(2):67-77.

3. Gopalan G, Goldstein L, Klingenstein K, Sicher C, Blake C, McKay M. Engaging families into child mental health treatment: updates and special considerations. J Can Acad Child Adolesc Psychiatry. 2010;19(3): 182-196.

4. Gopalan G. Feasibility of improving child behavioral health using task-shifting to implement the 4Rs and 2Ss program for strengthening families in child welfare. Pilot and Feasibility Stud. 2016;2(21). doi 10.1186/s40814-016-0062-2.

## A148 Increasing quality of care in Norwegian child welfare institutions: a quantitative analysis of factors from the high performance cycle and a test of job engagement

### Per Jostein Matre^1^, Rita Kylling^2^, Pamela Waaler^3^, Hans Nordahl^4^, Kitty Dahl^3^

#### ^1^Drammen Municipality, Drammen, Norway; ^2^Metanoia Mestring & Utvikling, Borgen, Norway; ^3^RBUP Øst og Sør, Oslo, Norway; ^4^Norwegian University of Science and Technology (NTNU), Trondheim, Norway

##### **Correspondence:** Per Jostein Matre (Per.Jostein.Matre@drmk.no)

**Background**

Seventy-six present of youth living in child welfare institutions in Norway meet criteria for 1 or more psychiatric disorders [1]. This high prevalence of emotional distress presents a significant challenge for Norwegian child welfare services. In order to provide effective help, institutional employees must be motivated, trained, and engaged.

From 2011 to 2014, a systematic implementation called Module-Based Support (MBS) was adopted to increase the quality of institutional care through therapist training and supervision. The current study examines which program factors contributed to therapist development and job engagement.

**Materials and Methods**

A quantitative cross-sectional design was utilized. The independent variable was job engagement, and High Performance Cycle (HPC) factors [2] including: demands, performance, contingent rewards, consequences, and job satisfaction were the dependent variables. Employees and leaders in Norway’s Region North working day and/or evening shifts participated. In 2011, 320 employees and 12 leaders were recruited (62% women, 38% men). The number of participants decreased to 230 in 2013 due to organizational restructuring.

Participants completed the Empowered Thinking Questionnaire (ETQ) 3 times between 2011 and 2014. Question topics involved: leader support, goal orientation, self-efficacy, attachment to the organization, job engagement, job satisfaction, demands, and organizational practice [3].

**Results**

A Structural Equation Modelling (SEM) analysis was conducted to test the relationship between job engagement and the dependent variables. The analysis demonstrated a significant relationship between demands and performance (b = .54), performance and contingent rewards (b = .85), contingent rewards and job satisfaction (b = .94), contingent rewards and job engagement (b = .58), and job satisfaction and consequences (b = .88). However, significant relationships were not found between job engagement and self-efficacy (b = .04), nor job engagement and job satisfaction (b = .02).

**Conclusions**

The results from the SEM analysis suggest that there are significant relationships between job engagement and HPC factors, which indicates that the HPC model is a valid tool for improving care quality in Norwegian child welfare institutions. Future implementation of the HPC model has significant implications for future policy developments, institutional practitioners, and implementation researchers.

**References**

1. Kayed NS, Jozefiak T, Rimehaug T, Tjelflaat T, Brubakk AB, Wichstrøm L. Psykisk helse hos barn og unge i barnevernsinstitusjoner. Trondheim, Norway: NTNU Regionalt Kunnskapssenter for Barn og Unge – Psykisk Helse og Barnevern; 2015

2. Kylling R. En kvantitativ analyse av High Performance Cycle og en test av jobbengasjement som et supplement til modellen, i barnevernsinstitusjoner ved Region-Nord. [master’s thesis]. Oslo, Norway: OsloMet University; 2014

3. Locke EA. Latham GP. New Developments in Goal Setting and Task Performance. New York, NY: Routledge; 2013.

## A149 Understanding rewards to facilitate implementation: perceptions of rewards and incentives across two government-supported systems in Kenya

### Noah Triplett^1^, Grace Woodard^1^, Christine Gray^2^, Leah Lucid^1^, Prerna Martin^1^, Rosemary Meza^1^, Kathryn Whetten^2^, Tyler Frederick^1^, Cyrilla Amanya^3^, Augustine Wasonga^3^, Shannon Dorsey^1^

#### ^1^University of Washington, Seattle, WA, USA; ^2^Duke University, Durham, NC, USA; ^3^ACE Africa Kenya, Mumias, Kenya

##### **Correspondence:** Noah Triplett (nst7@uw.edu)

**Background**

Evidence suggests mental health interventions can be effectively delivered via task-sharing in low-resource settings [1]. However, research focused on how to embed evidence-based treatments in government-funded systems to enable population-level scale-up and sustainment is limited [2,3].

**Materials and Methods**

We examined implementation policies and practices (IPPs) associated with facilitating group-based trauma-focused cognitive behavioral therapy (TF-CBT) for children and adolescents within the health and education systems in Kenya. Eighteen teachers and 18 community health volunteers (CHVs) from each system (N = 36) participated in qualitative interviews after delivering 2 consecutive TF-CBT groups. Interviews examined several IPPs, including rewards and incentives. Thematic coding was conducted by a team including the study’s principal investigator. Interviews were double-coded and discussed to consensus; a third coder was consulted when discordant.

**Results**

Rewards and incentives were perceived differently between systems. Teachers highlighted rewards and incentives within their profession. Most teachers felt they were able to be more effective teachers due to participating in the intervention (72%), while only 44% of CHVs reported improvement in their role due to participation. As salaried professionals, teachers did not receive compensation for participation; however, as volunteers, the CHVs received a stipend, which was endorsed by 61% as a reward/incentive. Further, CHVs—often considered the health system’s extension into the community—perceived additional rewards and incentives in relation to their communities that teachers did not. Nearly half of CHVs (44%) reported receiving rewards and incentives from outside their job (e.g., gifts/acknowledgment from community members), whereas only 2 teachers (11%) reported rewards/incentives from outside their profession. CHVs reported benefit to their personal life slightly more frequently than teachers (56% v 44%).

**Conclusions**

Encouraging participation and sustaining task-sharing interventions requires understanding how rewards and incentives are perceived. For professionals, like teachers, emphasizing rewards and incentives related to their profession may encourage participation and enable sustainment. For non-salaried volunteers, highlighting rewards and incentives from their communities might be more beneficial. In both systems, counselors reported similar levels of personal benefit, like using skills to manage their own grief, suggesting participation goes beyond profession and stipend to add value to counselors’ personal lives.

**Trial Registration** ClinicalTrials.gov NCT01822366

**References**

1. Patel V, Chowdhary N, Rahman A, Verdeli H. Improving access to psychological treatments: lessons from developing countries. Behav Res Ther. 2011;49(9):523-528.

2. Fairall L, Zwarenstein M, Thornicroft G. The applicability of trials of complex mental health interventions. In: Thornicroft G, Patel V, editors. Global Mental Health Trials. Oxford, UK: Oxford University Press; 2014.

3. Betancourt TS, Chambers DA. Optimizing an era of global mental health implementation science. JAMA Psychiatry. 2016;73(2):99-100.

## A150 A protocol for building mental health implementation capacity for Malawian and Tanzanian researchers and policymakers

### Christopher Akiba^1^, Vivian Go^1^, Victor Mwapasa^2^, Mina Hosseinipour^1^, Brad Gaynes^1^, Alemayehu Amberbir^3^, Michael Udedi^2^, Brian Pence^1^

#### ^1^University of North Carolina at Chapel Hill, Chapel Hill, NC, USA; ^2^University of Malawi College of Medicine, Blantyre, Malawi; ^3^Dignitas International, Zomba, Malawi; ^4^Malawi Ministry of Health, Lilongwe, Malawi

##### **Correspondence:** Christopher Akiba (akiba@live.unc.edu)

**Background**

Mental health disorders in low and middle-income countries (LMICs) account for nearly the same disease burden as HIV/AIDS [1,2]. While efficacious mental health treatments exist, access is severely limited [3]. This treatment gap is fueled by structural determinants rooted in a lack of research and policy capacity.

**Materials and Methods**

The goal of this abstract is to describe the capacity building procedures and preliminary results of the sub-Saharan Africa Regional Partnership (SHARP) for Mental Health Capacity Building. SHARP 1) strengthens implementation skills among Malawian and Tanzanian mental health researchers and policymakers to successfully apply research on evidence-based mental health programs into routine practice and 2) supports dialogue between researchers and policymakers leading to efficient and sustainable scale-up of mental health services. SHARP comprises five capacity building components for mental health researchers and policymakers including: 1) introductory and advanced short courses focused on implementation science, evidence-based mental health interventions, and grant writing; 2) a multifaceted dialogue platform; 3) an on-the-job training program; 4) annual pilot grants; and 5) mentorship courses. The impact of the program will be measured using dose, participant knowledge, participant satisfaction, and participant academic output.

**Results**

A group of 21 researchers and policymakers attended the introductory short courses (implementation science and evidence-based mental health interventions) in June 2018. Post-test knowledge scores increased by 87% and 15% respectively and received average user satisfaction ratings of 89% and 85%. Pilot grants focused on implementation science and mental health were awarded to 4 teams of one researcher and one policymaker in August 2018. Also, SHARP partners delivered 3 journal clubs to 41 researchers and policymakers in 2018.

**Conclusions**

Given the widespread lack of evidence-based mental health interventions brought to scale in LMICs, the experiences gained from the SHARP Capacity Building Program in Malawi and Tanzania will hold meaningful implications for a model of capacity building that could be replicated in other LMICs. If impactful, the SHARP Capacity Building Program could be used to sustainably increase the knowledge, skills, and mentorship capabilities of researchers and policymakers regarding evidence-based mental health treatment.

**Trial Registration** ClinicalTrials.gov NCT03711786

**References**

1. Patel V. Mental health in low- and middle-income countries. Br Med Bull. 2007;81–82(1):81–96. doi: 10.1093/bmb/ldm010.

2. Baxter AJ, Patton G, Scott KM, Degenhardt L, Whiteford HA. Global epidemiology of mental disorders: what are we missing? PloS One. 2013;8(6):e65514. doi: 10.1371/journal.pone.0065514.

3. Hanlon C, Luitel NP, Kathree T, Murhar V, Shrivasta S, Medhin G, Ssebunnya J, Fekadu A, Shidhaye R, Petersen I, Jordans M, Kigozi F, Thornicroft G, Patel V, Tomlinson M, Lund C, Breuer E, De Silva M, Prince M. Challenges and opportunities for implementing integrated mental health care: a district level situation analysis from five low- and middle-income countries. PLoS One. 2014;9(2):e88437. doi: 10.1371/ journal.pone.0088437.

## A151 The Health Equity Implementation Framework: proposal and preliminary study

### Eva Woodward^1^, Monica Matthieu^1^, Uchenna Uchendu^2^, Shari Rogal^3^, JoAnn E. Kirchner^4^

#### ^1^VA Center for Mental Healthcare Outcomes Research, Little Rock, AR, USA; ^2^Health Management Associates, Naples, FL, USA; ^3^VA Center for Health Equity Research & Promotion, Pittsburgh, PA, USA; ^4^VA Behavioral Health QUERI, Little Rock, AR, USA

##### **Correspondence:** Eva Woodward (enwoodward@uams.edu)

**Background**

Researchers could benefit from methodological advancements to advance uptake of new treatments while also improving health equity. A determinants framework for healthcare disparity implementation challenges is essential to understand an implementation problem and select implementation strategies.

**Materials and Methods**

We integrated and modified two conceptual frameworks—one from implementation science and one from healthcare disparities research to develop the Health Equity Implementation Framework. We applied the Health Equity Implementation Framework to a historical health disparity challenge--Hepatitis C Virus (HCV) and its treatment among Black patients seeking care in the U.S. Department of Veterans Affairs (VA). A specific implementation assessment at the patient level was needed to understand barriers to increasing uptake of HCV treatment, independent of cost. We conducted a preliminary study to assess how feasible it was for researchers to use the Health Equity Implementation Framework. We applied the framework to design the qualitative interview guide and interpret results. Using quantitative data to screen potential participants, this preliminary study consisted of semi-structured interviews with a purposively selected sample of Black, rural-dwelling, older adult VA patients (N=12), living with HCV, from VA medical clinics in the Southern part of the United States.

**Results**

The Health Equity Implementation Framework was feasible for implementation researchers. Barriers and facilitators were identified at all levels including the patient, provider (recipients), patient-provider interaction (clinical encounter), characteristics of treatment (innovation), and healthcare system (inner and outer context). Some barriers reflected general implementation issues (e.g., poor care coordination after testing positive for HCV). Other barriers were related to healthcare disparities and likely unique to racial minority patients (e.g., testimonials from Black peers about racial discrimination at VA). We identified several facilitators, including patient enthusiasm to obtain treatment because of its high cure rates, and VA clinics that offset HCV stigma by protecting patient confidentiality.

**Conclusions**

The Health Equity Implementation Framework showcases one way to modify an implementation framework to better assess health equity determinants as well. Researchers may be able to optimize the scientific yield of research inquiries by identifying and addressing factors that promote or impede implementation of novel treatments while also improving health equity.

## A152 Mental health disparities in CBT implementation

### Suh Jung Park, Hannah Listerud, Perrin Fugo, Emily Becker-Haimes, Rinad S. Beidas

#### University of Pennsylvania, Philadelphia, PA, USA

##### **Correspondence:** Suh Jung Park (suhp@sas.upenn.edu)

**Background**

The broader health care literature shows substantial racial and ethnic disparities in the implementation of evidence-based practices (EBPs) [1], but there is a paucity of literature examining disparities in mental health care implementation. Cognitive-behavioral therapy (CBT) is a gold-standard EBP for multiple youth mental health conditions yet is variably implemented in community mental health settings [2]. To date, little work has examined whether variability in CBT implementation differs as a function of client race and ethnicity; such findings would suggest the possibility of mental health disparities to target in future implementation research.

**Materials and Methods**

We examined the relationship between therapists’ fidelity to CBT and clients’ race and ethnicity in community mental health settings using data drawn from a larger study. Therapists (N=72, median age=34 years (IQR 29-44), 74% female, 69% white non-Hispanic/Latinx) audio-recorded CBT therapy sessions for three unique clients. Sessions were coded for the extent to which therapists delivered six CBT interventions (psychoeducation, cognitive education, cognitive distortion, functional analysis of behavior, relaxation strategies, and coping skills; other CBT interventions were excluded due to low use rates across the sample) using the Therapy Process Observational Coding System for Child Psychotherapy-Revised Strategies Scale. We classified clients into two groups: non-Hispanic/Latinx whites (N=53) and racial/ethnic minority (N=144) and examined whether therapist CBT scores differed as a function of client racial/ethnic minority status.

**Results**

Therapists used more relaxation strategies when treating racial/ethnic minority clients than when treating non-Hispanic/Latinx white clients (t=-2.847, p=.005). No other differences were observed (all ps>.05).

**Conclusions**

Results did not suggest that therapists in community settings used CBT differentially based on clients’ race and ethnicity. Study limitations include an overall restricted range of CBT intervention use, regardless of client racial/ethnic minority status. Implications for CBT implementation will be discussed.

**References**

1. Roth A, Fonagy P. What works for whom? A critical review of psychotherapy research. London, UK: Guilford; 1996.

2. Hoagwood K, Burns BJ, Kiser L, Ringeisen H, Schoenwald SK. Evidence-based practice in child and adolescent mental health services. Psychiatr Serv. 2001;52(9),1179-1189. doi:10.1176/appi.ps.52.9.1179

## A153 Putting it at their fingertips: mechanisms for increasing adoption of a technology-based performance management tool in a community mental health organization

### Cameo F. Stanick, Janine Quintero, Amanda Gentz, Gina Perez, Debbie Manners

#### Hathaway-Sycamores Child and Family Services, Los Angeles, CA, USA

##### **Correspondence:** Cameo F. Stanick (cstanick@hscfs.org)

**Background**

Research has demonstrated that simply giving providers progress data on their clients produces an effect size for clinically significant change [1-2]. However, the adoption of such innovations may be dependent on specific implementation constructs. Hathaway-Sycamores Child and Family Services (HSCFS) is one of the largest nonprofit community mental health and child welfare agencies in Los Angeles County serving thousands of youth and families. Within this context the current study describes a two-phase pilot project for evaluating the mechanisms underlying implementation of a performance management ‘dashboard,’ which allows key metrics to be tracked at multiple organizational levels (i.e., leadership to individual clinicians).

**Materials and Methods**

Participants in the first phase of the pilot included 11 staff and the second phase included 28 staff across two sites at HSCFS. In the first phase of the pilot, the dashboards were distributed weekly and created manually in Excel by agency staff. The second phase focused on identifying and implementing a technology tool for developing and distribution the dashboards using Microsoft’s Power BI, where data updated daily. To examine mechanisms that may impact implementation, Perceptions of Adopting an IT Innovation [3] and Readiness for Organizational Change [4] were administered before and after each phase.

**Results**

Results of the implementation measures showed that participants in the second phase had positive attitudes toward adoption (M=5.3, SD=.83). They also indicated high leadership support for implementing the dashboard (M=5.2, SD=1.1). Based on the changes implemented in the second phase (automating the dashboard; changing training strategies), participants reported positive attitudes particularly regarding conveying the usefulness of the tool (M=5.7, SD=.86). Despite the positive attitudes utilization of the tool decreased in the second phase.

**Conclusions**

Understanding which mechanisms underlie technology innovation adoption is critical for identifying how to aid stakeholders ‘getting ready’ for practices that may be different from current behavior. Despite research demonstrating the impact of attitudes on clinician behavior [5] positive perceptions of compatibility may not be enough to influence adoption at all levels. For instance, as this implementation launches agency wide, it will be important to address de-implementation of other tools. Limitations to the current project include a limited sample size and preliminary nature of the project.

**References**

1. Carlier IVE, Meuldijk D, Van Vliet IM, Van Fenema E, Van der Wee NJA, Zitman FG. Routine outcome monitoring and feedback on physical or mental health status: evidence and theory. J Eval Clin Pract. 2012;18:104-110.

2. Bickman L, Kelley SD, Breda C, de Andrade AR, Riemer M. Effects of routine feedback to clinicians on mental health outcomes of youth: results of a randomized trial. Psychiatr Serv. 2011;62:1423-1429.

3. Moore GC, Benbasat I. Development of an instrument to measure the perceptions of adopting an information technology innovation. Inf Syst Res. 1991;2(3):192-222.

4. Holt D, Armenakis A, Field H, Harris H. Readiness for organizational change: The systematic development of a scale. J Appl Behav Sci. 2007;43(2):232-255.

5. Okamura KH. Therapists’ knowledge of evidence-based practice: Differential definitions, measurement, and influence on self-reported practice (Order No. AAI10587357). 2018. https://search-proquest-com.weblib.lib.umt.edu: 2443/docview/1951458808?accountid=14593

## A154 Implementing internet-based CBT as part of a digital stepped care service in Australian general practice

### Isabel Zbukvic^1^, Elizabeth Hanley^1^, Fiona Shand^1^, Helen Christensen^1^, Nicole Cockayne^1^, Christiaan Viis^2^, Josephine Anderson^1^

#### ^1^Black Dog Institute, Randwick, Australia; ^2^Vrije Universiteit Amsterdam, Amsterdam, Netherlands

##### **Correspondence:** Isabel Zbukvic (i.zbukvic@blackdog.org.au)

**Background**

iCBT is an effective resource for GPs, who are often the first contact for people experiencing mental ill-health and psychological distress [1]. However, use of iCBT in primary care is not always routine. This study aims to improve GP and patient engagement with the evidence-based iCBT program myCompass [2], through tailored implementation of Black Dog Institute’s digital stepped care service StepCare [3]. This study forms part of the international implementation research project, ImpleMentAll [4].

**Materials and Methods**

StepCare is a digital mental health service designed for general practice. Using a smart tablet, patients screen for symptoms of depression, anxiety and alcohol misuse in the waiting room. Treatment recommendations based on symptom severity are sent from the tablet to the GP in real-time; patients with mild symptoms are recommended the self-guided iCBT program myCompass. Implementation-as-usual for the StepCare Service uses a train-the-trainer model, with collaboration between the PHN and Black Dog Institute. Implementers in IT, practice support, research, and project management operate within an integrated model of knowledge translation to roll-out the service using a phased approach.

**Results**

Data collection for this study is currently underway. Following a stepped-wedge trial design, a tailored implementation intervention (ItFits-toolkit) will be tested across 12 organizations in 9 countries over a period of 27 months. Longitudinal repeated-measures design will allow comparison of a baseline period of implementation-as-usual versus tailored implementation. Monthly implementation reporting captures activities commenced/stopped and barriers over time. Normalisation of GP use of myCompass as part of the StepCare service will be measured quarterly using the NoMAD and ORIC. Outcome measures will also include patient uptake of myCompass via StepCare as well as implementation costs. Implementer experience using the ItFits-toolkit will also be assessed at each site, along with a process evaluation of the ImpleMentAll trial.

**Conclusions**

The StepCare Service uses a technology solution to overcome known barriers to GP and patient engagement with online mental health care. This study will provide invaluable evidence on the effectiveness of tailored implementation strategies aimed at further improving engagement with iCBT in primary care.

**References**

1. Parslow RA, Lewis V, Marsh B. The general practitioner’s role in providing mental health services to Australians, 1997 and 2007: findings from the national surveys of mental health and wellbeing. Med J Aust. 2011;195(4):205-209.

2. Proudfoot J, Clarke J, Birch MR, Whitton AE, Parker G, Manicavasagar V, Harrison V, Christensen H, Hadzi-Pavlovic D. Impact of a mobile phone and web program on symptom and functional outcomes for people with mild-to-moderate depression, anxiety and stress: a randomised controlled trial. BMC Psychiatry. 2013;13:312.

3. Institute BD. StepCare Service: A digital mental health screening tool for patients in general practices. 2018. https://www.blackdoginstitute.org.au/clinical-resources/health-professional-resources/stepcare-service.

4. ImpleMentAll. 2017. http://www.implementall.eu/. Accessed 27 Feb 2019.

## A155 Development of a multicomponent implementation strategy for eScreening

### James Pittman^1,2,3^, Borsika Rabin^1,3^, Niloofar Afari^1,2,3^, Elizabeth Floto^4^, Erin Almklov^1^, Laurie Lindamer^1^

#### ^1^VA Center of Excellence for Stress and Mental Health, San Diego, CA, USA; ^2^VA San Diego Healthcare System, San Diego, CA, USA; ^3^University of California, San Diego, San Diego, CA, USA; ^4^VA Roseburg Health Care System, Roseburg, OR, USA

##### **Correspondence:** James Pittman (james.pittman@va.gov)

**Background**

eScreening is a VA mobile health technology that provides customized and automated self-report health screening via iPad, clinical alerts, patient feedback and medical record integration [1]. eScreening supports early identification of health problems and measurement-based care initiatives. We set out to evaluate an implementation strategy for this technology-based, self-screening tool that has shown promise for improving the provision of healthcare services and patient outcomes. The Lean/Six Sigma Rapid Process Improvement Workshop (RPIW) and related playbook are commonly used in VA quality improvement efforts. We used this approach as an implementation strategy to prepare adopters of eScreening in VA facilities.

**Materials and Methods**

We adapted a Lean Six Sigma RPIW [2] to develop an implementation strategy. Our multicomponent implementation strategy consisted of a modified RPIW, a playbook (roadmap for programs to implement eScreening, including how to conduct their own RPIW), internal champions, and external facilitation through bi-monthly calls, a site visit, and technical assistance. We conducted a feasibility study in 2 VHA clinics to evaluate the impact of RPIW on implementation. We gathered qualitative data from site visits and consultation calls, and conducted post implementation interviews. Two of our team members independently reviewed qualitative data using a rapid analytic approach to identify challenges and solutions.

**Results**

The implementation strategy was used with slight variation across the two sites. One site conducted a comprehensive RPIW (Site 1) and the other relied on the other components of the implementation strategy (Site 2). Data from the pilot revealed three types of implementation challenges that occurred at both sites: technology-related, system level, and educational. Workflow and staffing resources were challenges only at Site 2. All four types of implementation barriers were resolved using the external facilitator and the playbook.

**Conclusions**

Findings suggest that the use of the modified RPIW may have solved workflow/staffing issues more efficiently than locally identified strategies. The modified RPIW to implement new programs in VA health care systems shows promise. External facilitation helped overcome challenges with or without the RPIW. Our findings support prior research highlighting the importance of considering multiple change mechanisms of implementation strategies in mental health services [3].

**References**

1. Pittman JOE, Floto E, Lindamer L, Baker DG, Lohr JB, Afari N. VA escreening program: technology to improve care for post-9/11 veterans. Psychol Serv. 2017; 14(1):23-33.

2.Koning H, Verver JPS, Heuvel J, Bisgaard S, Does RJMM. Lean Six Sigma in healthcare. J Healthc Qual. 2006;28(2):4–11.

3.Williams N. Multilevel mechanisms of implementation strategies in mental health: integrating theory, research, and practice. Adm Policy Ment Health. 2015;43(5):783-796.

## A156 Implementation of an automated text messaging system for patient self-management in the Department of Veterans Affairs: a qualitative study

### Vera Yakovchenko, Timothy Hogan, Lorilei Richardson, Beth Ann Petrakis, Christopher Gillespie, Derek Bolivar, D. Keith McInnes

#### Department of Veterans Affairs, Bedford, MA, USA

##### **Correspondence:** Vera Yakovchenko (vera.yakovchenko@va.gov)

**Background**

The Department of Veterans Affairs (VA) is currently deploying an automated texting system (ATS) to support patient self-management. Guided by the Non-adoption, Abandonment, Scale-Up, Spread, and Sustainability Framework (NASSS) which is intended to support the evaluation of novel technologies, we conducted a qualitative study to examine barriers and facilitators to national rollout of the ATS.

**Materials and Methods**

Semi-structured interviews were conducted with 33 providers and 38 patients at formative and summative stages of ATS implementation. Interviews explored the roles of site personnel in ATS implementation, processes for enrolling patients, and ATS user experiences. Interviews were recorded and transcribed verbatim. Data were analyzed via qualitative content analysis using emergent coding and a priori codes based on the NASSS framework.

**Results**

We identified themes across NASSS domains: 1) Condition: perceptions of patient appropriateness for the ATS were guided by texting experience and health complexity rather than potential benefit; 2) Technology: for providers, although the ATS resides outside the electronic health record, use was generally not considered laborious; 3) Value Proposition: patient-driven demand for the ATS was limited; 4) Adopters: providers recommended more efficient ATS enrollment processes to reduce workload; 5) Organization: providers did not have observable results from the ATS early in the implementation phase, noting that such evidence of patient progress/use could enhance uptake among other providers; 6) Wider System: despite being a national program, autonomy at the local level yielded varied experiences with the ATS; and 7) Embedding and Adaptation Over Time: once using the ATS, providers recognized potential for use with other conditions.

**Conclusions**

This is among the first studies to explore implementation of VA’s ATS and through the lens of the NASSS framework. The NASSS framework highlighted how the system can be better embedded into current practices, which patients might benefit most from its functionality, and which aspects of ATS messages are potentially most relevant to self-management. Mobile phone SMS texting is rapidly becoming an accepted means of asynchronous communication between healthcare systems and patients. Our findings reveal that VA’s ATS has potential to expand the reach of VA care; however, providers require additional support to adopt, implement, and sustain ATS use.

## A157 Beyond journals – using visual abstracts to promote wider research dissemination

### Adam Hoffberg^1^, Joe Huggins^1^, Audrey Cobb^1^, Jeri Forster^1,2^, Nazanin Bahraini^1,2^

#### ^1^Rocky Mountain MIRECC, Denver, CO, USA; ^2^University of Colorado, Boulder, CO, USA

##### **Correspondence:** Adam Hoffberg (adam.hoffberg@va.gov)

**Background**

Many academic and journal organizations disseminate research via social media to increase accessibility and reach a wider audience. With the widespread utilization of Twitter, more research is needed to study the extent to which social media strategies influence outcomes on awareness and readership of journal publications. “Visual Abstracts” have been adopted by some organizations as a novel approach to increase engagement with academic content. Visual Abstracts are a visual representation of key methods and findings found in a traditional written publication [1]. This study will help organizations understand the potential impact of adopting Visual Abstracts into their social media dissemination efforts. Potential pitfalls will also be discussed.

**Materials and Methods**

A prospective, case-control crossover design was utilized to randomize n=50 journal publications comparing Twitter posts with a Visual Abstract to those with simple screen grab of the PubMed abstract. We used native Twitter Analytics to track the outcomes of impressions, retweets, total engagements, and link clicks about 28 days post-Tweet, and Altmetric It to track additional alternative metric outcomes.

**Results**

As of this submission, n=47 out of n=50 articles have been randomized, with complete follow-up data available for n=33 publications. Preliminary analyses indicate that overall, Visual Abstract tweets on average have 432 more impressions, 2 additional retweets, 1 additional link click, 5 additional total engagements, and increase the Altmetric score by 3 compared with Text Tweets. Full results from the study will be analyzed and presented.

**Conclusions**

Conclusions are pending, but it is expected that in line with results from prior studies [2,3] we will find a significant association between the use of Visual Abstract tweets and increased dissemination on social media. These findings may provide further evidence that Twitter is an effective platform for research dissemination and highlight the importance of social media for suicide prevention researchers and other stakeholders to communicate findings. Future efforts will be discussed to implement Visual Abstracts at scale and refine processes to maximize engagement.

**References**

1. Ibrahim AM. A primer on how to create a visual abstract. 2016. www.SurgeryRedesign.com/resources.

2. Ibrahim A, Lillemoe K, Klingensmith M, Dimick J. Visual abstracts to disseminate research on social media. Ann Surg. 2017;266(6):e46-e48. doi: 10.1097/SLA.0000000000002277

3. Lindquist LA, Ramirez-Zohfeld V. Visual Abstracts to Disseminate Geriatrics Research Through Social Media. J Am Geriatr Soc. 2019. 7(6):1128-1131. doi:10.1111/jgs.15853

## A158 Using web- and mobile technology to track implementation of evidence-based mental health treatments in schools

### Elizabeth Koschmann, Emily Berregaard, Shawna Smith, Seoyoun Choi, Amy Rusch, John Hess

#### University of Michigan, Ann Arbor, MI, USA

##### **Correspondence:** Elizabeth Koschmann (felizabe@med.umich.edu)

**Background**

Forty percent of youth experience mental illness [1], yet access to evidence-based treatments (EBTs) is limited by a shortage of trained providers, poor treatment fidelity, and low consumer help-seeking knowledge [2-3]. School-based delivery of EBTs can improve access [4] and research demonstrates that EBTs can be delivered effectively by school-based mental health professionals (SMHPs) [5]. Implementation strategies, such as consultation, coaching, and facilitation, promote adoption and sustainment of EBTs in schools [6-8], however, evaluation of implementation interventions requires collection of appropriate implementation metrics [9] that is hindered in schools by absence of a unified, acceptable reporting system.

**Materials and Methods**

TRAILS is a statewide implementation model designed to increase SMHP delivery of CBT, and incorporates didactic instruction, technical support, and in-person coaching. To enable program evaluation, TRAILS developed a web application, the TRAILS Dashboard, which allows SMHPs to easily record weekly delivery of treatment components, self-reported fidelity, and track student clinical outcomes. Self-report data are cross-validated with standardized assessments and observer (Coach) ratings to inform TRAILS implementation efforts.

**Results**

Development of the TRAILS Dashboard included concept design, wireframing, user testing, and build. Currently, the Dashboard is being utilized within a randomized implementation-effectiveness trial, ASIC, evaluating implementation strategies. All collection of implementation (SMHP-level) and clinical effectiveness (student-level) data occurs through the Dashboard, as does management of student suicide risk. A coach dashboard documents delivery of coaching elements as well as observational ratings of SMHP treatment fidelity. 169 SMHPs are actively using the Dashboard, recording their weekly CBT delivery since November 2018; and have identified 1,347 student participants for clinical outcomes data collection. Response rates for weekly data are consistently above 80% independent of implementation strategy treatment arm.

**Conclusions**

The TRAILS Dashboard presents a novel solution to a common problem in non-clinical implementation research—the lack of a unified system for tracking implementation outcomes. Deployment within the ASIC study allows testing of basic utility and ease of use, but ongoing initiatives are informing future functions to further tailor TRAILS implementation support, namely dynamic training targeting SMPH skill deficiencies, “nudges” to increase treatment frequency or fidelity, and prompts recommending specific treatment components for students based on clinical data.

**References**

1. Merikangas KR, He JP, Burstein M, Swanson SA, Avenevoli S, Cui L, Benjet C, Georgiades K, Swendsen J. Lifetime prevalence of mental disorders in U.S. adolescents: results from the National Comorbidity Survey Replication—Adolescent Supplement (NCS-A). J Am Acad Child Adolesc Psychiatry. 2010;49(10):980-9. doi: 10.1016/ j.jaac.2010.05.017.

2. Beidas RS, Kendall PC. Dissemination and implementation of evidence-based practices in child and adolescent mental health. New York: Oxford University Press; 2014

3. Curran GM, Bauer M, Mittman B, Pyne JM, Stetler C. Effectiveness-implementation hybrid designs: combining elements of clinical effectiveness and implementation research to enhance public health impact. Med Care. 2012;50(3):217-226.

4. Owens JS, Lyon AR, Brandt NE, Warner CM, Nadeem E, Spiel C, Wagner M. Implementation science in school mental health: key constructs in a developing research agenda. Sch Ment Health. 2013;6(2):99-111.

5. Masia Warner C, Colognori D, Brice C, Herzig K, Mufson L, Lynch C, Reiss PT, Petkova E, Fox J, Moceri DC, Ryan J, Klein RG. Can school counselors deliver cognitive-behavioral treatment for social anxiety effectively? A randomized controlled trial. J Child Psychol Psychiatry. 2016;57(11):1229-1238. doi:10.1111/jcpp.12550.

6. Powell BJ, Beidas RS, Lewis CC, Aarons GA, McMillen JC, Proctor EK, Mandell DS. Methods to improve the selection and tailoring of implementation strategies. J Behav Health Serv Res. 2017;44(2):177-194. doi: 10.1007/s11414-015-9475-6.

7. Kilbourne AM, Smith SN, Choi SY, Koschmann E, Liebrecht C, Rusch A, Abelson JL, Eisenberg D, Himle JA, Fitzgerald K, Almirall D. Adaptive School-based Implementation of CBT (ASIC): clustered-SMART for building an optimized adaptive implementation intervention to improve uptake of mental health interventions in schools. Implement Sci. 2018;13(1):119. doi: 10.1186/s13012-018-0808-8.

8. Koschmann ES, Abelson JL, Kilbourne AM, Smith SN, Fitzgerald K, Pasternak A. Implementing evidence-based mental health practices in schools: feasibility of a coaching strategy. (under review).

9. Proctor E, Silmere H, Raghavan R, Hovmand P, Aarons G, Bunger A, Griffey R, Hensley M. Outcomes for implementation research: conceptual distinctions, measurement challenges, and research agenda. Adm Policy Ment Health. 2011;38(2):65-76. doi: 10.1007/s10488-010-0319-7.

## A159 Formative research findings from the metallic Improved Cooking Stove (ICS) installation project for improving respiratory health in Manekharka, Nepal

### Jayoung Park^1^, Prabin Raj Shakya^1^, Sugy Choi^2^, Jongho Heo^3^, Woong-Han Kim^1^

#### ^1^Seoul National University College of Medicine, Seoul, South Korea; ^2^Boston University, Boston, MA, USA; ^3^National Assembly Futures Institute, Seoul, South Korea

##### **Correspondence:** Jayoung Park (jayoungpark91@gmail.com)

**Background**

In Nepal, the second highest ranking risk factor for death and disability combined is indoor air pollution (IAP) [1]. IAP is a risk factor for numerous diseases including pneumonia, stroke, ischemic heart disease, chronic obstructive pulmonary disease (COPD), and lung cancer [2]. Improved Cooking Stove (ICS) is known to reduce the exposure to IAP [3], but its implementation can have its challenges based on its context and setting. The JWLEE CGM and Dhulikhel Hospital collaborated to conduct formative research prior to the implementation of the project. This study presents the results of the baseline survey for phase 1 and qualitative results.

**Materials and Methods**

A pragmatic hybrid type 1 design with a step-wedged trial is used. The study is focused on ward 4 for Panchpokhari-Thanpalkot rural municipality, Sindupalchowk District in the north-eastern part of Nepal. Out of all 480 households, a total of 363 households and a total of 663 household individuals were surveyed for the baseline study and data were collected digitally through CommCare HQ. The household-level questions included the main cook’s fuel use, cooking dynamics, and health symptoms. The individual-level questions asked for fuel use of household members and their quality of life. To assess the implementation of facilitators and challenges, the planning team conducted focus groups of key stakeholders and community leaders to plan the intervention specifications. Data were analyzed qualitatively using NVivo.

**Results**

The baseline survey results showed that 211 (59.8%) were currently using three-stone fire and 219 (79.0%) were using wood for their primary cooking fuel. 343 (97.2%) participants used charcoal as their alternative option for cooking fuel. 226 (64.0%) of the participants preferred a cookstove design with less smoke. The qualitative analysis revealed that enhancing cultural sensitivity and cultural relevance for adoption, implementation, and maintenance are relevant to effectiveness. Perceptions of complexibility can become a potential barrier in implementing ICS.

**Conclusions**

This baseline study shows that the ICS project can potentially have an impact on the targeted households. Ensuring the adoption of optimal and appropriate technologies by conducting formative research can lead to facilitation of the intervention and improvement in the quality of the overall project.

**References**

1. University of Washington Institute for Health Metrics and Evaluation. Nepal. 2018. http://www. healthdata.org/nepal. Accessed 25 February 2019.

2. World Health Organization. Household air pollution and health. 2018. Available at: https://www.who. int/news-room/fact-sheets/detail/household-air-pollution-and-health. Accessed 1 April 2019

3. Jeuland M, Pattanayak SK Bluffstone RA. The economics of household air pollution. Annu Rev Resour Economics. 2015;7:81-108. doi:10.1146/annurev-resource-100814-125048

## A160 Ontario neurotrauma foundation’s journey: from traditional funder to impact driven funding

### Helene Gagne, Judith Gargaro

#### Ontario Neurotrauma Foundation, Toronto, Canada

##### **Correspondence:** Helene Gagne (helene.gagne@onf.org)

**Background**

Increasingly it is important to align funding priorities with practice and system realities. It is necessary to ground research and knowledge generation activities in real-world settings and engage broad stakeholders with interest in the research results in program and system planning, delivery and implementation [1]. As a funder we still aim to identify knowledge gaps and share the results with key stakeholders, but we recognize that knowledge translation/mobilization although necessary cannot be enough to bring evidence-based findings into practice.

**Materials and Methods**

Our approach has moved to funding fewer projects addressing practice gaps and priorities in healthcare with a view to implementing results and innovations, using an implementation science framework. The funding approach has further evolved from funding only individual projects to now funding collaborative and system-level initiatives. Evidence exists that implementation processes rooted in implementation science can be effective, but there are issues of sustainability if the implementation is not embedded at the system level [2]. It is necessary to foster partnerships and involve broad stakeholders in all phases of implementation.

The key pillars of our integrative approach are knowledge generation, knowledge mobilisation and effective implementation approaches. As a funder this approach works well as we can specify the type of services that we provide and can be nimble to respond to real-world challenges. Sometimes it is not possible to apply the full spectrum of implementation science activities from beginning to end; action must occur where it is needed to increase capacity of implementing organisations within the system they work in. All our efforts are designed with a view for scalability and sustainability [3].

**Results**

Within our three streams of activity (injury prevention, acquired brain injury and spinal cord injury) we have amassed a wealth of knowledge around implementation at the local and system levels and have developed an implementation support service resource to coordinate our implementation efforts in an explicit and consistent way.

**Conclusions**

Examples will be discussed that illustrate our systematic approach across the three streams and the lessons learned in moving from traditional funding to impact funding.

**References**

1. Rycroft-Malone J, Burton CR, Bucknall T, Graham ID, Hutchinson AM, Stacey D. Collaboration and co-production of knowledge in healthcare: opportunities and challenges. Int J Health Policy and Manag. 2016;5(4):221–3. doi: 10.15171/ ijhpm.2016.08.

2. Nilson P. Making sense of implementation theories, models and frameworks. Implement Sci. 2015;10(53). doi: 10.1186/s13012-015-0242-0.

3. Sustainable Improvement Team and the Horizons Team. Leading large-scale change: a practical guide. 2018. https://www.england.nhs.uk/wp-content/uploads/2017/09/practical-guide-large-scale-change-april-2018-smll.pdf. Accessed 2018 March 2019.

## A161 New directions for implementation strategy design: applying behavioral economic insights to design EBP implementation strategies in community mental health settings

### Vivian Byeon^1^, Rebecca Stewart^1^, Rinad Beidas^1^, Briana Last^1^, Katelin Hoskins^1^, Nathaniel Williams^2^, Alison Buttenheim^1^

#### ^1^University of Pennsylvania, Philadelphia, PA, USA; ^2^Boise State University, Boise, ID, USA

##### **Correspondence:** Vivian Byeon (vivianbyeon@gmail.com)

**Background**

The field of behavioral economics provides ro bust explanations for why individuals choose and behave as they do [1], yet little work has applied these insights to the development of implementation strategies that influence community mental health clinicians’ choices and behavior with regard to evidence-based practice. In this study, a team of behavioral scientists and frontline mental health clinicians in the city of Philadelphia used a systematic approach to the identification of behavioral barriers to EBP use in order to develop novel implementation strategies for community mental health settings. This poster describes our approach and results.

**Materials and Methods**

We followed a four-step process to 1) define our target problem; 2) map the relevant decisions and actions underlying the behavior; 3) brainstorm hypothesized behavioral barriers using previously-collected contextual inquiry data (implementation strategy ideas that therapists generated during an innovation tournament) [2-3], linked to specific behavioral science constructs; and 4) conduct rapid validation of hypothesized barriers through expert consultation, literature review, and a clinician focus group.

**Results**

Drawing on the crowdsourcing data from clinicians, the investigative team generated 156 hypotheses of behavioral barriers to EBP use. Two investigators then de-duplicated and synthesized hypotheses down to a list of 21. We are currently in the last stages of the rapid validation process with community clinicians and will present the final hypotheses to support implementation strategy design in our poster session.

**Conclusions**

To our knowledge, this is the first study incorporating principles of behavioral economics and participatory design to the development of implementation strategies. This systematic, rigorous, and innovative process will allow us to develop candidate implementation strategies from the insights of clinicians. Results from this participatory and behavioral process will drive the design of tailored implementation strategies that will explicitly address and overcome the identified behavioral barriers.

**References**

1. Tversky A, Kahneman D. The framing of decisions and the psychology of choice. Science. 1981; 211(4481):453-458.

2. Terwiesch C, Ulrich K. Innovation tournaments: creating and selecting exceptional opportunities. Boston, MA: Harvard Business Review Press; 2009.

3. Beidas, RS, Volpp, KG, Buttenheim, AN Marcus SC, Olfson M, Pellecchia M, Stewart RE, Williams NJ, Becker-Haimes EM, Candon M, Cidav Z, Fishman J, Lieberman A, Zentgraf K, Mandell D. Transforming mental health delivery through behavioral economics and implementation science: protocol for three exploratory projects. JMIR Res Prot. 2019;8(2): e12121.

## A162 Examining how different engagement procedures in facilitated interprofessional collaborative processes optimize type-2 diabetes prevention in routine primary care

### Alvaro Sanchez^1^, Gonzalo Grandes^2^, Susana Pablo^2^, Arturo García-Álvarez^2^

#### ^1^Osakidetza-Basque Health System, Bilbao, Spain; ^2^BioCruces Bizkaia Health Research Institute, Osakidetza, Bizkaia, Spain

##### **Correspondence:** Alvaro Sanchez (alvaro.sanchezperez@osakidetza.eus)

**Background**

Most efficient procedure to engage and guide healthcare professionals in collaborative processes that seek to optimize practice is unknown [1]. The PREDIAPS project aims to assess the effectiveness and feasibility of different engagement procedures to perform a facilitated interprofessional collaborative process to optimize type-2 diabetes prevention in routine Primary Care [2].

**Materials and Methods**

Randomized cluster implementation trial conducted in nine PHC centers from the Basque Health Service. All centers received training on effective healthy lifestyles promotion. Headed by a local leader and an external facilitator, centers conducted a collaborative structured process to adapt the intervention and its implementation to their specific context [3]. One of the groups was allocated to apply this strategy globally, promoting the cooperation of all health professionals from the beginning. The other performed it sequentially, centered first on nurses, who lately seek the pragmatic cooperation of physicians. All patients without diabetes aged ≥30 years old with a known CVD risk factor and an abnormal glucose level (≥110-125 mg/dl) who attended centers during the study period were eligible for program inclusion. Main outcome measures focus on changes in T2D prevention practice indicators after 12 months.

**Results**

Exposition rate of professionals to the implementation strategy actions were similar in both groups but higher in nurses (86%) than in physicians (75%). After 12 months, 2916 eligible at risk patients attended at least once to their family physician, of which 401 (13.8%) have been addressed by assessing their healthy lifestyles in both comparison groups. The proportion of attending patients at risk of T2D receiving a personalized prescription of a healthy lifestyle change (N=214; 7.3%) was slightly higher in the Sequential (7.8%; range 5.5%-10.8%) than in the Global group (6.3%; range 5.8%-6.7%). The proportion of patients receiving a lifestyle prescription from those assessed is also higher in the Sequential than in the Global group (55% vs. 50%).

**Conclusions**

Preliminary results showed that the reach of the implanted intervention programs derived by the PREDIAPS implementation strategy is acceptable but slightly higher in the Sequential group. Center’s organizational context has determined implementation results (professional commitment, work overload, multiple corporative initiatives, staff turn-over, etc.).

**References**

1. Lau R, Stevenson F, Ong BN, Dziedzic K, Treweek S, Eldridge S, Everitt H, Kennedy A, Qureshi N, Rogers A, Peacock R, Murray E. Achieving change in primary care—causes of the evidence to practice gap: systematic reviews of reviews. Implement Sci. 2016;11:40.

2. Sanchez A, Grandes G, Pablo S, Espinosa M, Torres A, García-Alvarez A; PREDIAPS Group. Engaging primary care professionals in collaborative processes for optimising type 2 diabetes prevention practice: the PREDIAPS cluster randomised type II hybrid implementation trial. Implement Sci. 2018;13(1):94.

3. Grandes G, Sanchez A, Cortada JM, Pombo H, Martinez C, Balagué L, Corrales MH, de la Peña E, Mugica J, Gorostiza E; PVS group. Collaborative modeling of an implementation strategy: a case study to integrate health promotion in primary and community care. BMC Res Notes. 2017;10(1):699

## A163 An approach to align barriers and implementation strategies to accelerate adoption of evidence-based practice: CVD risk calculator adoption in primary care

### Laura-Mae Baldwin^1^, Leah Tuzzio^2^, Allison Cole^1^, Erika Holden^2^, Jennifer Powell^3^, Michael Parchman^2^

#### ^1^University of Washington, Seattle, WA, USA; ^2^Kaiser Permanente Washington Health Research Institute, Seattle, WA, USA; ^3^Powell & Associates, Ashville, NC, USA

##### **Correspondence:** Laura-Mae Baldwin (lmb@uw.edu)

**Background**

Healthy Hearts Northwest (H2N), one of seven AHRQ-funded EvidenceNOW cooperatives, is a pragmatic clinical trial to test different strategies for implementing evidence-based interventions to improve heart health in primary care practice [1]. One intervention was a virtual educational outreach visit (EOV) to increase use of cardiovascular disease (CVD) risk calculation to inform statin use for prevention of CVD [2]. Five physician educators conducted 30-minute EOVs in 44 H2N practices and elicited 13 barriers to implementing CVD risk calculation.

**Materials and Methods**

To compare the implementation strategies that implementation scientists and primary care clinicians chose as most likely to overcome barriers to implementing a CVD risk calculator in practice.

Modified nominal group exercise involving implementation scientists and primary care clinicians, with synthesis of the exercise results Participants: 5 implementation scientists and 26 clinicians from primary care clinics in the WWAMI region Practice and Research Network.

Every implementation scientist and clinician chose their top 5 implementation strategies from a list of the 73 evidence-based, published strategies from the Expert Recommendations for Implementing Change (ERIC) study [3] for the 13 barriers. For each barrier, we examined the degree of agreement among ≥30% of clinicians, ≥30% of scientists, or ≥30% of both on chosen strategies.

**Results**

≥ 30% of clinicians and/or scientists chose 39/73 top implementation strategies they felt were the most important to address barriers to CVD risk calculation. Implementation scientists chose 34 of these 39 top strategies; clinicians chose 21. Scientists and clinicians agreed on the choice of 14 of the top implementation strategies. On average, a total of 7 top implementation strategies were chosen by either scientists or clinicians for each barrier; however, scientists and clinicians agreed on only 1 of these top strategies.

**Conclusions**

Implementation scientists and clinicians generally choose different implementation strategies to overcome barriers to implementing a CVD risk calculator in practice. Collaboration between these stakeholders could guide the choice of a broader range of strategies to overcome barriers to evidence-based practice.

**Trial Registration:** ClinicalTrials.gov NCT02839382

**References**

1. Parchman ML, Anderson ML, Dorr DA, Fagnan LJ, O’Meara ES, Tuzzio L, Penfold RB, Cook AJ, Hummel J, Conway C, Cholan R, Baldwin L-M. A randomized trial of external practice support to improve cardiovascular risk factors in primary care. Ann Fam Med. 2019;17(Supp 1):S40-S49.

2. Baldwin LM, Fischer MA, Powell J, Holden E, Tuzzio L. Fagnan LJ, Hummel J, Parchman ML. Virtual educational outreach intervention in primary care based on the principles of academic detailing. J Contin Educ Health Prof. 2018; 38(4):269-275.

3. Powell BJ, Waltz TJ, Chinman MJ, Damschroder LJ, Smith JL, Matthieu MM, Proctor EK, Kirchner JE. A refined compilation of implementation strategies: results from the Expert Recommendations for Implementing Change (ERIC) project. Implement Sci. 2015;10(1):21. doi: 10.1186/s13012-015-0209-1.

## A164 Learning by doing: implementing the VA Cardiovascular Toolkit and learning to fail better

### Bevanne Bean-Mayberry^1^, Erin Finley^2^, Alison Hamilton^1,3^, Tannaz Moin^1,3^, Melissa Farmer^1,3^

#### ^1^VA Greater Los Angeles, Los Angeles, CA, UA; ^2^STVHCS/UTHSA, San Antonio, TX, USA; ^3^University of California, Los Angeles, Los Angeles, CA, USA

##### **Correspondence:** Bevanne Bean-Mayberry (bevanne.bean-mayberry@va.gov)

**Background**

Cardiovascular (CV) disease is the main cause of death in American women and CV risk factors are often less controlled in women compared to men. To address CV risk among women Veterans, we developed a CV toolkit comprised of a patient screener, an electronic health record template, and a health education group tailored for women to target CV goal-setting. We describe how iterative cycles of adaptation during implementation allows us to improve fit with local context, address barriers, and increase engagement with CV toolkit components.

**Materials and Methods**

Guided by Replicating Effective Programs (REP) enhanced with complexity theory, we are conducting an 18-month CV Toolkit study in four sites to increase engagement in CV risk reduction services. During implementation, we monitor staff/provider use of the template and track patient engagement in VA services. Periodic reflections are used to review monthly progress and document multilevel stakeholder engagement and sense-making.

**Results**

Despite early and frequent engagement, there was limited utilization of the patient screener and template over a one-year period at the first site, with staff and providers reporting difficulty remembering to use these tools. While providers made frequent referrals to health education group, participation was low, and remained low even after employing several strategies to increase participation, including audio-care calls and secure messaging. Implementation at later sites indicated that scheduling patients for group classes increased participation and clinical reminders helped care teams incorporate CV toolkit into routine workflow; these suggestions were then incorporated at the first site. Stakeholder (patient, provider and operational partners) feedback led us to develop a telephone facilitated group to increase engagement.

**Conclusions**

The emphasis on nonlinear cycles of tailoring and adaptation in REP gave our team a structured process by which to proactively learn from early failures, engaging stakeholders consistently in identifying pragmatic solutions that aligned with routine workflow. These solutions became real-time modifications to the implementation process to mold tools to the local context, observe uptake and document outcomes.

**Trial Registration:** ClinicalTrials.gov NCT02991534

## A165 Longitudinal assessment of Expert Recommendations for Implementing Change (ERIC) strategies in the uptake of evidence-based practices for Hepatitis C treatment in the Veterans Administration

### Vera Yakovchenko^1^, Rachel Gonzalez^1^, Angela Park^1^, Timothy Morgan^1^, Maggie Chartier^1^, David Ross^1^, Matthew Chinman^2^, Shari Rogal^1^

#### ^1^Department of Veterans Affairs, Bedford, MA, United States; ^2^RAND Corporation, Pittsburgh, PA, USA

##### **Correspondence:** Vera Yakovchenko (vera.yakovchenko@va.gov)

**Background**

To increase access to evidence-based treatments for hepatitis C (HCV), the Department of Veterans Affairs (VA) established a national collaborative composed of regional teams of providers, leaders, and staff tasked with conducting local implementation strategies to increase HCV treatment initiations. The aim of this longitudinal evaluation was to assess how site-level implementation strategies were associated with HCV treatment initiation.

**Materials and Methods**

A representative from each VA site (N=130) was asked in four consecutive fiscal years (FYs) to complete an online survey examining use of 73 implementation strategies organized into nine clusters as described by the Expert Recommendations for Implementing Change (ERIC) study. The number of Veterans initiating treatment for HCV, or “treatment starts”, at each site was captured using administrative data. Descriptive, nonparametric, and multivariate analyses were conducted on the respondents in FY15 (N=80), FY16 (N=105), FY17 (N=109), and FY18 (N=88).

**Results**

Of 130 sites, 127 (98%) responded at least once and 54 (42%) responded across all four years. A mean of 25±14 strategies were endorsed in FY15, 28±14, 26±15, and 35±26 in FY16, FY17, and FY18, respectively. While the number of strategies increased over time, the correlation between number of strategies and HCV treatment decreased over time. The most commonly endorsed strategies across all years were: data warehousing techniques, tailoring strategies to deliver HCV care, and intervening with patients to promote uptake and adherence to HCV treatment. One strategy (“make efforts to identify early adopters to learn from their experiences”) was significantly associated with treatment starts in all four years. In FY15, strategies were focused on developing interrelationships, FY16 focused on using evaluative and iterative strategies, FY17 focused on training and educating stakeholders, and FY18 focused on providing interactive assistance. The important strategies in each year were then mapped to Exploration, Preparation, Implementation, Sustainment framework stages.

**Conclusions**

This evaluation represents the first large-scale four-year assessment of implementation strategies nationwide. Surveying providers about ERIC strategies is a feasible way to understand the associations between strategies and clinical outcomes over time. These results add to our understanding of the implementation strategies used over time and across stages of planning, implementation, and sustainability.

**Trial Registration** ClinicalTrials.gov NCT04178096

## A166 Implementing pharmacy-located hpv vaccination: findings from pilot projects in five U.S. states

### William Calo^1^, Parth Shah^2^, Melissa Gilkey^3^, Robin Vanderpool^4^, Sarah Barden^5^, William Doucette^6^, Noel Brewer^3^

#### ^1^Pennsylvania State University, University Park, PA, USA; ^2^Fred Hutchinson Cancer Research Center, Seattle, WA, USA; ^3^University of North Carolina, Chapel Hill, NC, USA; ^4^University of Kentucky, Lexington, KY, USA; ^5^Michigan Pharmacists Association, Lansing, MI, USA; ^6^University of Iowa, Iowa City, IA, USA

##### **Correspondence:** William Calo (wcalo@phs.psu.edu)

**Background**

Up-to-date human papillomavirus (HPV) vaccination in the US has increased since the vaccine’s introduction over a decade ago to 49% of adolescents ages 13-17 in 2017 [1]. However, vaccination coverage remains far below the Healthy People 2020 goal of 80% for adolescents ages 13-15 [1]. As a strategy to improve uptake, the President’s Cancer Panel [2] and the National Vaccine Advisory Committee [3] have recommended expanding HPV vaccine provision in pharmacies. Pharmacies are promising alternative settings for HPV vaccination because of their population reach, convenience, and existing infrastructure for vaccine delivery. As a result, pilot projects conducted in five states aimed to demonstrate the utility of pharmacy-located HPV vaccination services for adolescents. To date, no study we are aware of has documented the experiences of implementing HPV vaccination programs in real-world pharmacy settings. We sought to document challenges and opportunities of implementing pharmacy-located HPV vaccination services in five US states.

**Materials and Methods**

We evaluated the success of the pilot projects by mapping reported results to key implementation science constructs: service penetration, acceptability, appropriateness, feasibility, fidelity, adoption, and sustainability [4,5]. Pilot projects were planned in North Carolina (k=2 pharmacies), Michigan (k=10), Iowa (k=2), Kentucky (k=1), and Oregon (no pharmacy recruited) with varying procedures and recruitment strategies. Sites had open enrollment for a combined 12 months.

**Results**

Despite substantial efforts in these states, only 13 HPV vaccine doses were administered to adolescents and three doses to age-eligible young adults. We identified two major reasons for these underperforming results. First, poor outcomes on service penetration and appropriateness pointed to engagement barriers: low parent demand and engagement among pharmacy staff. Second, poor outcomes on feasibility, adoption, and sustainability appeared to result from administrative hurdles: lacking third party reimbursement (i.e., billing commercial payers, participation in Vaccines for Children program) and limited integration into primary care systems.

**Conclusions**

In summary, pilot projects in five states all struggled to administer HPV vaccines. Opportunities for making pharmacies a successful setting for adolescent HPV vaccination include expanding third party reimbursement to cover all vaccines administered by pharmacists, increasing public awareness of pharmacists’ immunization training, and improving care coordination with primary care providers.

**References**

1. Walker TY, Elam-Evans LD, Yankey D, Markowitz LE, Williams CL, Mbaeyi SA, Fredua B, Stokley S. National, regional, state, and selected local area vaccination coverage among adolescents aged 13–17 years—United States, 2017. MMWR Morb Mortal Wkly Rep. 2018;67(33):909.

2. President’s Cancer Panel. HPV vaccination for cancer prevention: progress, opportunities, and a renewed call to action. a report to the President of the United States from the C=chair of the President’s Cancer Panel. Bethesda, MD. 2018.

3. National Vaccine Advisory Committee. Recommendations to address low HPV vaccination coverage rates in the United States. June 9, 2015.

4. Gerke D, Lewis E, Prusaczyk B, Hanley C, Baumann A, Proctor E. 2017. Eight toolkits related to Dissemination and Implementation. Implementation Outcomes. https://sites.wustl.edu/wudandi/. Accessed 28 Nov 2018.

5. Proctor E, Silmere H, Raghavan R, Hovmand P, Aarons G, Bunger A, Griffey R, Hensley M. Outcomes for implementation research: conceptual distinctions, measurement challenges, and research agenda. Adm Policy Ment Health. 2011;38(2):65-76.

## A167 Identifying facilitators and barriers for the implementation of a complex intervention for patients with metastatic lung cancer

### Anja Siegle^1^, Corinna Jung^1^, Nicole Deis^2^, Jasmin Bossert^1^, Katja Krug^1^, Michel Wensing^1^, Jana Jünger^2^, Michael Thomas^1^, Matthias Villalobos^1^

#### ^1^Heidelberg University Hospital, Heidelberg, Germany; ^2^The German National Institute for State Examinations in Medicine, Pharmacy and Psychotherapy, Mainz, Germany

##### **Correspondence:** Anja Siegle (a.siegle@gmx.net)

**Background**

The German National Cancer Plan and the American Society of Clinical Oncology (ASCO) [1] recommend early integration of palliative care and advance care planning for patients with metastatic lung cancer. To address these recommendations, the Heidelberg Milestone communication approach (MCA) has been developed [2]. MCA is a complex intervention involving tandems of physicians and nurses. It aims at providing coherent care along the disease trajectory and integrates palliative care early. The MCA has been implemented in a theory-led way [3] in the in- and out-patient departments of a comprehensive cancer center (hospital setting). While the degree of the implementation success is still under scrutiny, the importance of identifying barriers and facilitators of the MCA is already apparent. The aim of this study is to identify barriers and facilitators of the MCA implementation.

**Materials and Method**

A qualitative content analysis [4] of written minutes (n= 47) of implementation meetings with nurses, doctors and hospital managers is conducted. The data analysis comprises open coding, development of main categories, identification of sub categories and application of these categories. As a theoretical framework for implementation evaluation of the MCA, the Consolidated Framework for Implementation research (CFIR) [5] is used. MAXQDA is used to organize the collected data.

**Results**

Preliminary results will be presented on aspects facilitating or hindering implementation in the following dimensions: characteristics of the intervention (e.g. adaption, fit), inner (e.g. communications, climate, readiness) and outer (e.g. economic, political, social context) settings, individuals involved (e.g. interaction between individuals, influence of individual or organizational behavior) and implementation process (and sub-processes).

**Conclusions**

Knowing facilitators and barriers of the MCA supports future implementation processes in this hospital. Understanding this implementation process helps to identify determinants for successful implementation processes of the MCA in other organizations.

**References**

1. Peppercorn JM, Smith TJ, Helft PR, Debono DJ, Berry SR, Wollins DS, Hayes DM, Von Roenn JH, Schnipper LE; American Society of Clinical Oncology. American Society of Clinical Oncology statement: toward individualized care for patients with advanced cancer. J Clin Oncol. 2011;29(6):755-760.

2. Siegle A, Villalobos M, Bossert J, Krug K, Hagelskamp L, Krisam J, Handtke V, Deis N, Jünger J, Wensing M, Thomas M. The Heidelberg Milestones Communication Approach (MCA) for patients with prognosis <12 months: protocol for a mixed-methods study including a randomized controlled trial. Trials. 2018;19(1):1-13.

3. Grol R, Wensing M, Eccles M, Davis D. Improving patient care: the implementation of change in health care. 2nd ed. Chichester: John Wiley & Sons, Ltd; 2013.

4. Kuckartz U. Qualitative text analysis: a guide to methods, practice and using software. London: Sage Publications Ltd; 2014.

5. Damschroder LJ, Aron DC, Keith RE, Kirsh SR, Alexander JA, Lowery JC. Fostering implementation of health services research findings into practice: a consolidated framework for advancing implementation science. Implement Sci. 2009; 4:50.

## A168 Using mixed methods to adapt and evaluate the implementation of a comprehensive tobacco-free workplace program within behavioral health care facilities in Texas

### Isabel Martinez Leal^1^, Kathy Le^1^, Daniel O’Connor^1^, Bryce Kyburz^2^, Virmarie Corrrea-Fernández^1^, Teresa Williams^2^, Lorraine Reitzel^1^

#### ^1^University of Houston, Houston, TX, USA; ^2^Integral Care, Austin, TX, USA

##### **Correspondence:** Isabel Martinez Leal (imarti31@central.uh.edu)

**Background**

Despite the highest rates of tobacco use and tobacco-related morbidity and mortality, smokers with behavioral health disorders rarely receive tobacco dependence treatment within behavioral health care settings. Taking Texas Tobacco Free (TTTF) has successfully targeted this disparity by delivering a multi-component, tobacco-free workplace program providing policy implementation and enforcement, education, provider training in tobacco screenings and treatments, and nicotine replacement therapies to behavioral health clinics across Texas [1-3]. Via a mixed methods [4,5] approach we used a formative evaluation process to adapt implementation strategies to local contexts and evaluated program outcomes and characterized processes influencing implementation in two local mental health authorities serving 17 clinics.

**Materials and Methods**

Varied data collection included pre and post-implementation leader, provider, and staff surveys; and pre, mid, and post-implementation provider, staff and consumer focus groups. During implementation, data were collected via various logs (tobacco screenings, nicotine replacement therapy delivery) to monitor program content delivery.

**Results**

All clinics adopted a 100% tobacco-free workplace policy, integrated tobacco screenings into routine practice, delivered evidence-based interventions, dispensed nicotine replacement therapies to consumers and staff, and recorded significant increases in provider knowledge on how to address tobacco dependence. Pre, mid, and post-implementation qualitative findings served to: 1) develop program strategies (educational tools, videos) and materials (brochures, posters) adapted to local contexts and populations and address barriers; 2) adjust delivery systems of key components to enhance implementation; 3) understand reasons for success or failure to implement specific practices, respectively; and 4) reveal program integration into clinic culture, enhancing sustainability.

**Conclusions**

TTTF has proven successful in integrating tobacco cessation interventions into regular clinical practice to address tobacco use within behavioral health clinics. Mixing methods involved program adopters and recipients as collaborators who directly impacted implementation by shaping the intervention to their individual context and needs. Collaboration of such key stakeholders was vital to increasing program fit, ownership, adoption and sustainability; closing the gap between research and practice. These findings contribute to the development of flexible strategies and tailored interventions responsive to real-world conditions in diverse settings which better address implementation barriers thus enhancing the effectiveness and sustainability of a tobacco-free workplace program.

**References**

1. Correa-Fernández V, Wilson WT, Kyburz B, O’Connor DP, Stacey T, Williams T, Lam CY, Reitzel LR. Evaluation of the Taking Texas Tobacco Free Workplace Program within behavioral health centers. Transl Behav Med. 2019;9(2):319-327

2. Correa-Fernández V, Wilson WT, Shedrick DA, Kyburz B, L Samaha H, Stacey T, Williams T, Lam CY, Reitzel LR. Implementation of a tobacco-free workplace program at a local mental health authority. Transl Behav Med. 2017;7(2):204-211.

3. Samaha HL, Correa-Fernández V, Lam C, Wilson WT, Kyburz B, Stacey T, Williams T, Reitzel LR. Addressing tobacco use among consumers and staff at behavioral health treatment facilities through comprehensive workplace programming. Health Promot Prac. 2017;18(4):561-570.

4. Teddlie C, Tashakkori A. Major issues and controversies in the use of mixed methods in the social and behavioral sciences. Handbook of mixed methods in social & behavioral research. Thousand Oaks, CA: SAGE; 2003. p. 3-50.

5. Palinkas LA, Aarons GA, Horwitz S, Chamberlain P, Hurlburt M, Landsverk J. Mixed method designs in implementation research. Adm Policy Ment Health. 2011;38(1):44-53.

## A169 Building practitioner competency in implementation science to drive comprehensive cancer control planning

### **Correspondence:** Margaret Farrell (farrellm@mail.nih.gov)

#### National Cancer Institute, Bethesda, MD, USA

**Background**

A greater understanding and uptake of implementation science frameworks and measures can help both cancer control practitioners and researchers leverage crucial insights into how to best deliver research-based initiatives in the complex communities where they are crucially needed. In April, the National Cancer Institute (NCI) released Implementation Science at a Glance, a workbook to introduce practitioners and policymakers to the building blocks of implementation science. This resource is a natural extension of our experiences funding [1] and training researchers in implementation science as well as perspectives NCI gained through this work [2].

**Materials and Methods**

A preliminary draft of the resource was reviewed by fifty-eight cancer control researchers and practitioners for clarity and concept. The final version reflected advances both in our understanding of implementation science and how to communicate it to support and inform the work of cancer control practitioners.

**Results**

Case studies illustrate implementation science in practice, provide lessons learned in the field, and brief practitioners about the components of IS including evidence-based interventions, fidelity, adaptations, stakeholder engagements, theories, models, and frameworks, strategies, evaluation, and sustainability.

**Conclusions**

This presentation will outline how Implementation Science at a Glance illustrates implementation science frameworks, models and measures to help drive community and organizational transformation and, in turn, develop broader disparities-reducing implementation strategies. By providing insights supporting greater uptake of Implementation science research designs, the resource offers rigorous methods that could accelerate the pace at which equity is achieved in real-world practice.

**References**

1. Neta G, Sanchez MA, Chambers DA, Phillips SM, Leyva B, Cynkin L, Farrell MM, Heurtin-Roberts S, Vinson C. Implementation science in cancer prevention and control: a decade of grant funding by the National Cancer Institute and future directions. Implement Sci. 2015;10(1):4

2. National Cancer Institute. Implementation Science at a glance: a guide for cancer control practitioners. [Bethesda, MD]: US Department of Health and Human Services, National Institutes of Health, National Cancer Institute, 2019. NIH Publication Number 19-CA-8055.

## A170 PharmFIT: assessing the feasibility of a pharmacy-based fecal immunochemical test kit distribution program to increase colorectal cancer screening access

### Mary Wangen^1^, Catherine Rohweder^1^, Rachel Ceballos^2^, Renee Ferrari^1^, Rachel Issaka^2^, Dan Reuland^1^, Jennifer Richmond^1^, Sara Rubio Correa^1^, Parth Shah^2^, Stephanie Wheeler^1^, Alison Brenner^1^

#### ^1^University of North Carolina at Chapel Hill, Chapel Hill, NC, USA; ^2^Fred Hutchinson Cancer Research Center, Seattle, WA, USA

##### **Correspondence:** Mary Wangen (wange062@live.unc.edu)

**Background**

Rural populations have lower rates of colorectal cancer (CRC) screening [1-3] and sub-optimal access to preventive care services [4]. In North Carolina, geospatial analyses have revealed that sub-optimal access to health services in rural regions significantly related to higher rates of CRC mortality in rural “hotspots” [4]. As such, identifying alternative health care settings in rural areas that could deliver CRC screenings may be one way to alleviating this health inequity. Pharmacies may be an opportune setting to distribute fecal immunochemical test (FIT) kits for CRC screening in rural areas as one way to improve access to CRC screening in communities with poorer access to traditional care delivery settings. FIT kits are a guideline-recommended screening test that patients can complete at home [5-7]. This formative work will assess the feasibility and acceptability of delivering FIT kits for CRC screening in pharmacy settings (PharmFIT).

**Materials and Methods**

We are conducting semi-structured interviews with key informants to elicit: 1) knowledge, attitudes, and perceptions of a PharmFIT intervention; 2) barriers and facilitators to implementing PharmFIT; and 3) recommendations for implementation strategies that would support successful delivery of a PharmFIT intervention. We are interviewing 12 primary care providers (PCP), 12 pharmacists and pharmacy technicians, and 12 patients (n=36). PCP and pharmacy staff interviews are focused on care coordination and follow-up processes and procedures, whereas patient interviews are focused on the acceptability and relative advantage of receiving FIT kits from pharmacies. Interviews are audio-recorded, transcribed and independently coded by two team members using a directed content analysis approach [8, 9] PRISM [10] and Diffusion of Innovations Theory guide analysis and organization of themes.

**Results**

Pharmacies are well-positioned to increase access to preventive health services such as colorectal cancer screening. Patients, pharmacists, and primary care providers have voiced support for an extended role for pharmacists in delivering FIT kits for colorectal cancer screening.

**Conclusions**

Results from this study can be used to elucidate key care coordination and follow-up issues for primary care providers and pharmacy staff and to identify implementation strategies [11] needed to target identified barriers (e.g., training pharmacists) to test intervention implementation and effectiveness.

**References**

1. Siegel RL, Sahar L, Robbins A, Jemal A. Where can colorectal cancer screening interventions have the most impact? Cancer Epidemiol Biomarkers Prev. 2015;24(8):1151-1156.

2. Cole AM, Jackson JE, Doescher M. Urban-rural disparities in colorectal cancer screening: cross-sectional analysis of 1998-2005 data from the Centers for Disease Control’s Behavioral Risk Factor Surveillance Study. Cancer Med. 2012; 1(3):350-6.

3. Blake KD, Moss JL, Gaysynsky A, Srinivasan S, Croyle RT. Making the case for investment in rural cancer control: An analysis of rural cancer incidence, mortality, and funding trends. Cancer Epidemiol Biomarkers Prev. 2017;26(7):992-997.

4. Kuo TM, Meyer AM, Baggett CD, Olshan AF. Examining determinants of geographic variation in colorectal cancer mortality in North Carolina: A spatial analysis approach. Cancer Epidemiol. 2019;59:8-14.

5. Pignone M, Rich M, Teutsch SM, Berg AO, Lohr KN. Screening for colorectal cancer in adults at average risk: a summary of the evidence for the U.S. Preventive Services Task Force. Ann Intern Med. 2002;137(2):132-141.

6. Knudsen AB, Zauber AG, Rutter CM, Naber SK, Doria-Rose VP, Pabiniak C, Johanson C, Fischer SE, Lansdorp-Vogelaar I, Kuntz KM. Estimation of benefits, burden, and harms of colorectal cancer screening strategies: modeling study for the US Preventive Services Task Force. JAMA. 2016;315(23):2595-2609.

7. US Preventive Services Task Force, Bibbins-Domingo K, Grossman DC, et al. Screening for colorectal cancer: US Preventive Services Task Force recommendation statement. JAMA. 2016;315(23):2564-2575.

8. Beebe J. Rapid assessment process: an introduction. Lanham: AltaMira Press; 2001.

9. Krippendorff K. Content Analysis: an introduction to its methodology 2ed. Thousand Oaks, CA: Sage; 2004.

10. Feldstein AC, Glasgow RE. A practical, robust implementation and sustainability model (PRISM) for integrating research findings into practice. Jt Comm J Qual Patient Saf. 2008;34(4):228-243.

11. Powell BJ, Waltz TJ, Chinman MJ, Damschroder LJ, Smith JL, Matthieu MM, Proctor EK, Kirchner JE. A refined compilation of implementation strategies: results from the Expert Recommendations for Implementing Change (ERIC) project. Implement Sci. 2015;10(1):21.

## A171 A systematic review of the barriers and enablers to implementation of menu labelling interventions from a food service industry perspective

### Claire Kerins^1^, Sheena McHugh^2^, Jennifer McSharry^1^, Catherine Hayes^3^, Caitlin M. Reardon^4^, Fiona Geaney^2^, Ivan J. Perry^2^, Suzanne Seery^5^, Colette Kelly^1^

#### ^1^National University of Ireland Galway, Galway, Ireland; ^2^University College Cork, Cork, Ireland; ^3^Trinity College Dublin, Dublin, Ireland; ^4^Ann Arbor VA Center for Clinical Management Research, Ann Arbor, MI, USA; ^5^National Institute for Prevention and Cardiovascular Health, Galway, Ireland

##### **Correspondence:** Claire Kerins (c.kerins2@nuigalway.ie)

**Background**

Menu labelling has gathered growing public and legislative support in response to the increased consumption of foods outside the home and the associated risks of overweight and obesity. A recent systematic review has shown menu labelling effects consumer food choice and the food industry behaviour [1]. Several countries have introduced menu labelling policies on a voluntary or mandatory basis; however, challenges to implementation have arisen (e.g. poor uptake, inaccurate nutritional information). The aim of this systematic review was to synthesise the evidence on the barriers and enablers to menu labelling implementation from the food industry perspective.

**Materials and Methods**

The review adopted the ‘best fit’ framework synthesis approach, designed for policy urgent questions [2]. No restrictions applied to publication type, study design, data collection method, language or publication year. At least two independent reviewers performed study selection, data extraction and quality appraisal. A combination of deductive coding, using the Consolidated Framework for Implementation Research [3] as the a priori framework, and inductive analysis, using secondary thematic analysis were undertaken.

**Results**

The overall process led to the construction of an adapted version of the CFIR. Of the 2,806 articles identified, 17 studies met the eligibility criteria. Most frequently cited barriers were coded to the CFIR constructs ‘Consumer Needs & Resources’ (e.g. lack of customer demand and understanding) and ‘Compatibility’ (e.g. lack of standardised recipes, limited space on menus). Commonly cited facilitators were coded to the CFIR constructs ‘Relative Advantage’ (e.g. improved business image/reputation) and ‘Consumer Needs & Resources’ (e.g. customer demand, enabling healthier food choices). Relationships between constructs (across and within domains) were also evident. The revised framework, based on the final list of constructs from the deductive and inductive coding, maintained many of the essential elements of the CFIR but a number of (sub)constructs with no supporting data were removed and newly developed constructs incorporated.

**Conclusions**

Findings from this review provide a foundation for selecting and tailoring implementation strategies to improve adoption, implementation, sustainment, and scale-up of menu labelling interventions. Moreover, in refining the CFIR, this review provides a theoretical contribution to help advance the field of implementation science.

**References**

1. Shangguan S, Afshin A, Shulkin M, Ma W, Marsden D, Smith J, Saheb-Kashaf M, Shi P, Micha R, Imamura F, Mozaffarian D; Food PRICE Project. a meta-analysis of food labeling effects on consumer diet behaviors and industry practices. Am J Prev Med. 2019;56(2):300-314.

2. Kerins C, McSharry J, Hayes C, Perry IJ, Geaney F, Kelly C. Barriers and facilitators to implementation of menu labelling interventions to support healthy food choices: a mixed methods systematic review protocol. Syst Rev. 2018; 7(1):88.

3. Damschroder LJ, Aron DC, Keith RE, Kirsh SR, Alexander JA, Lowery JC. Fostering implementation of health services research findings into practice: a consolidated framework for advancing implementation science. Implement Sci. 2009; 4(1):50.

## A172 Multiple stakeholders’ perspectives on screening older adults for malnutrition and food insecurity in an emergency department setting

### Jessa Engelberg^1^, Andrea Morris^1^, Aileen Aylward^2^, Rayad Shams^2^, Tim Platts-Mills^2^

#### ^1^West Health Institute, La Jolla, CA, USA; ^2^University of North Carolina, Chapel Hill, NC, USA

##### **Correspondence:** Jessa Engelberg (jengelberg@westhealth.org)

**Background**

Malnutrition is common among older adults and contributes to poor health and premature death [1-3]. Malnutrition is a complex condition with medical and social risk factors [4-6], including food insecurity. We are developing and implementing a two-phase screening study to identify older patients at-risk for malnutrition and food insecurity in the emergency department (ED) and connect them to a community-based organization (CBO) to address social needs.

**Materials and Methods**

During Phase 1, multiple stakeholder perspectives (i.e., ED, patient, CBO) were collected on how best to implement a sustainable ED-based screening process that considers the complexities and rapid pace of the ED. ED stakeholders included registered nurses (RNs), nursing assistants (NAs), social workers (SWs). To understand ED perspectives, research staff conducted semi-structured interviews (SSIs) with interview guides developed using the Consolidated Framework for Implementation Research (CFIR) [7] Guides included constructs from four CFIR domains (i.e., intervention characteristics, inner setting, characteristics of individuals, process). SSIs were transcribed and analyzed using framework-guided rapid analysis [8-9]. To understand the patient perspective, research staff tested screening questions with 75 older patients to identify how many are at-risk, and gauge receptivity to screening and referrals within the ED. Research staff worked with a CBO to understand their perspective on data sharing, referral pathways and “closing-the-loop.”

**Results**

Nine SSIs (n=3 RNs, n=2 NAs, n=4 SWs) were analyzed. Common themes related to constructs from CFIR domains were identified, including building the screener into the EHR, comparing to existing screening processes, and educating ED staff. A critical finding was that NAs, not RNs, should screen patients. From the sample of patients screened, approximately 35% were positive for malnutrition, 18% for food insecurity and 8% for both. Patients were receptive to being screened in the ED and indicated they would be willing to receive help connecting to CBOs. The CBO informed the development of referral workflows, particularly bidirectional communication and datapoints to facilitate data sharing.

**Conclusions**

The stakeholders’ perspectives informed the workflows that will be implemented and evaluated in Phase 2, including NAs screening, SWs connecting patients to the CBO, and the CBO sharing updates on the status of services provided to patients.

**References**

1. Pereira GF, Bulik CM, Weaver MA, Holland WC, Platts-Mills TF. Malnutrition among cognitively intact, noncritically ill older adults in the emergency department. Ann Emerg Med. 2015;65(1):85-91

2. Saka B, Kaya O, Ozturk GB, Erten N, Karan MA. Malnutrition in the elderly and its relationship with other geriatric syndromes. Clin Nutr. 2010;29(6):745-748

3. Agarwal E, Ferguson M, Banks M, Batterham M, Bauer J, Capra S, Isenring E. Malnutrition and poor food intake are associated with prolonged hospital stay, frequent readmissions, and greater in-hospital mortality: results from the Nutrition Care Day Survey 2010. Clin Nutr. 2013;32(5):737-745

4. Burks CE, Jones CW, Braz VA, Swor RA, Richmond NL, Hwang KS, Hollowell AG, Weaver MA, Platts-Mills T. Risk factors for malnutrition among older adults in the emergency department: a multicenter study. J Am Geriatr Soc. 2017;65(8):1741-1747.

5. Agarwal E, Miller M, Yaxley A, Isenring E. Malnutrition in the elderly: a narrative review. Maturitas. 2013;76(4):296-302

6. National Academies of Sciences E, and Medicine (NASEM). Meeting the dietary needs of older adults: Exploring the impact of the physical, social, and cultural environment: Workshop summary. Washington, DC: The National Academies Press; 2016.

7. Damschroder LJ, Aron DC. Keith RE, Kirsh SR, Alexander JA, Lowery JC. Fostering implementation of health services research findings into practice: a consolidated framework for advancing implementation science. Implement Sci. 2009; 4:50.

8. Gale RC, Wu J, Erhardt T, Bounthavong M, Reardon CM, Damschroder LJ, Midboe AM. Comparison of rapid vs in-depth qualitative analytic methods from a process evaluation of academic detailing in the Veterans Health Administration. Implement Sci. 2019;14(1):11.

9. Keith RE, Crosson JC, O’Malley AS, Cromp D, Taylor EF. Using the consolidated framework for implementation research (CFIR) to produce actionable findings: a rapid-cycle evaluation approach to improving implementation. Implement Sci. 2017;12:15.

## A173 Effective implementation strategies for male engagement in a program for Zambian couples experiencing IPV and substance mis-use

### Laura Eise^1^, Stephanie Skavenski van Wyk^2^, Jeremy C. Kane^2^, Kristina Metz^2^, Laura K. Murray^2^

#### ^1^University of Washington, Seattle, WA, USA; ^2^Johns Hopkins University, Baltimore, MD, USA

##### **Correspondence:** Laura Eise (laura.m.eise@gmail.com)

**Background**

The link between violence and substance use is well established. However, the violence literature has largely evaluated prevention programs, or those focused on women only. The need to include male counterparts is recognized, yet challenging. Evidence indicates that males disconnect from health-care services at high rates and, furthermore, information specific to low- and middle-income countries (LMIC) is scarce. In order to offer effective care, we must better understand how to engage men in these situations, which may include economic barriers, pervasive societal attitudes, and self-stigma to access available services.

**Materials and Methods**

We recently completed a randomized controlled trial (RCT) of a transdiagnostic psychotherapy (the Common Elements Treatment Approach; CETA) delivered to Zambian couples who indicated recent intimate partner violence (IPV), as well as male substance misuse. In order to recruit participants within a concentrated period (i.e., 12 weeks), we worked closely with a local partner that had successfully engaged men in an alcohol mis-use program. The partner utilized a multi-tiered process of community word-of-mouth and engagement by respected peers, with an emphasis on a non-stigmatizing process of screening.

**Results**

Throughout the study, there were high rates of male engagement and significant treatment effects both for the reduction of violence and alcohol use. To gain a more in-depth understanding of the RCT findings, including the male engagement and retention, we qualitatively explored mechanisms of behavior change related to male perpetration of IPV. We sampled adult men and women from the 123 couples randomized to the intervention arm. We conducted 30 first round interviews (16 women; 14 men) and then re-interviewed 20 participants (13 women; 7 men). In addition, we conducted 4 focus groups (2 women; 2 men). We also analyzed an implementation log maintained by study staff documenting engagement and retention strategies, challenges, and successes throughout the study.

**Conclusions**

Based on the results of this data collection and analysis, our poster will highlight the successful implementation strategies used in this RCT on IPV and substance use, present the data documenting the engagement and retention of male participants, and summarize the qualitative responses from men themselves that highlight key facilitators and barriers to engagement.

**Trial Registration:** ClinicalTrials.gov NCT02790827

## A174 Changing policy and practice in substance use treatment clinics through the implementation of a tailored, comprehensive tobacco free workplace program

### Lorraine Reitzel^1^, Bryce Kyburz^2^, Isabel Leal^1^, Kathy Le^1^, Virmarie Correa-Fernandez^1^, Teresa Williams^2^, Daniel O’Connor^1^, Ezemenari Obasi^1^, Kathleen Casey^2^

#### ^1^University of Houston, Houston, TX, USA; ^2^Integral Care, Austin, TX, USA

##### **Correspondence:** Lorraine Reitzel (lrreitzel@uh.edu)

**Background**

Despite elevated tobacco use rates among individuals in treatment for substance use disorders in Texas, only 70.2% of treatment clinics screen consumers for tobacco use, 55.4% provide cessation counseling, 24% offer nicotine replacement therapies, and 34.3% have a tobacco-free workplace policy [1]. Comprehensive tobacco-free workplace programs that include all of these evidence-based strategies are known to be effective in reducing tobacco use among this vulnerable population [1]. Taking Texas Tobacco Free is one such tobacco-free workplace program that has been successfully implemented within hundreds of behavioral health clinics in Texas (www.takingtexastobaccofree. 65(8):1741-1747 com) [2-5]. In 2017, we were funded to expand the program to dedicated substance use treatment centers.

**Materials and Methods**

Taking Texas Tobacco Free implementation includes a tobacco-free workplace policy along with the provision of education and specialized provider training to enable the institution of regular tobacco-use assessments and treatment provision or referral for tobacco dependence. A mixed-methods, formative evaluation process is used to understand clinic-specific facilitators and potential barriers, which guides the implementation strategies within diverse contexts. Consultation, practical guidance, and treatment resources are provided and mechanisms for program sustainability are emphasized.

**Results**

Enrolled clinics (N=8) serve ~70,000 unique consumers annually, including some special groups (e.g., sexual minorities, women with children). Thus far, Taking Texas Tobacco Free has educated 1,119 professionals through 61 discrete sessions and reached 92,521 individuals through passive material dissemination. Each clinic has adopted a 100% tobacco-free workplace policy, integrated tobacco-use assessments into routine practice, delivered evidence-based interventions, and dispensed nicotine replacement therapies to consumers and staff. Recruitment is ongoing.

**Conclusions**

Taking Texas Tobacco Free is an effective comprehensive tobacco control program that has proven successful in implementing tobacco-free workplace policies, training providers in tobacco cessation interventions and in integrating those interventions into regular clinical practice within substance use treatment clinics. This presentation will describe the program, participating clinics, data-based strategies used to tailor implementation within each setting, accomplishments to date (e.g., knowledge gained by staff and clinicians, tobacco-use assessments provided, pre- versus post-implementation changes in clinician behavior), and lessons learned during the implementation process that can guide program dissemination in other settings and states.

**References**

1. Marynak K, VanFrank B, Tetlow S, Mahoney M, Phillips E, Jamal Mbbs A, Schecter A, Tipperman D, Babb S. Tobacco cessation interventions and smoke-free policies in mental health and substance abuse treatment facilities — United States, 2016. MMWR Morb Mortal Wkly Rep. 2018;67:519–523.

2. Correa-Fernández V, Wilson WT, Kyburz B, O’Connor DP, Stacey T, Williams T, Lam CY, Reitzel LR. Evaluation of the Taking Texas Tobacco Free Workplace Program within behavioral health centers. Transl Behav Med. 2019;9(2):319-327

3. Correa-Fernández V, Wilson WT, Shedrick DA, Kyburz B, L Samaha H, Stacey T, Williams T, Lam CY, Reitzel LR. Implementation of a tobacco-free workplace program at a local mental health authority. Transl Behav Med. 2017;7(2):204-211.

4. Samaha HL, Correa-Fernández V, Lam C, Wilson WT, Kyburz B, Stacey T, Williams T, Reitzel LR. Addressing tobacco use among consumers and staff at behavioral health treatment facilities through comprehensive workplace programming. Health Promot Prac. 2017;18(4):561-570.

5. Centers for Disease Control and Prevention (CDC). Promising policies and practices to address tobacco use by persons with mental and substance use disorders: Texas Provides NRT as part of a range of tobacco cessation measures in mental health treatment settings. 2018. https://www.cdc.gov/tobacco/disparities/ promising-policies-and-practices/pdfs/osh-behavioral-health-promising-practices-20160709-p.pdf. Accessed 7 March 2019.

## A175 Conducting a process evaluation of a statewide opioid prescribing policy: applying the Consolidated Framework for Implementation Research across all phases of data collection and analysis

### Natalie Blackburn^1^, Elizabeth Joniak-Grant^2^, Maryalice Nocera^3^, Jada Walker^3^, Shabbar Ranapurwala^3^

#### ^1^UNC-Chapel Hill, Chapel Hill, NC, USA; ^2^National Coalition of Independent Scholars, Battleboro, VT, USA; ^3^UNC Injury Prevention Research Center, University of North Carolina at Chapel Hill, Chapel Hill, NC, USA

##### **Correspondence:** Natalie Blackburn (nblackbu@live.unc.edu)

**Background**

Opioid dependence and overdose are serious public health concerns in the United States [1]. States have sought to identify and implement policy interventions to address these growing public health problems. One policy intervention identified has been to set prescribing limits such that physicians prescribe no more than five days’ supply for post-surgical acute pain in an effort to reduce patient misuse and overdose [2]. As such in 2017 North Carolina passed the Strengthen Opioid Misuse Prevention (STOP) act which limits prescriptions to three to five-day supplies of opioids for acute post-surgical pain. The purpose of this study is to understand the barriers and facilitators in the passage and implementation of the STOP act in North Carolina.

**Materials and Methods**

Three groups were identified as key figures in the passage and implementation of the STOP act: government officials, hospital administrators, and opioid prescribers. Using the Consolidated Framework for Implementation Science (CFIR) [3] we developed three separate interview guides to be administered in one-on-one interviews. Two researchers developed the guides by reviewing all constructs within the CFIR and identifying construct-derived questions that would be most salient for each interview group. Questions were adapted to fit a policy context as well as fitting the role of the individual in policy implementation.

**Results**

This research project is currently in the data collection stage. Interviews will be conducted from March to May 2019; analysis will begin in June 2019. In addition to using the CFIR to design the interview guides, the CFIR will inform our coding of interview data and the development of analytical themes. Interviews will be analyzed across groups in order to summarize the perspectives and identify unifying or diverging ideas that may inform how the law is being implemented in the state.

**Conclusions**

Few studies have used the CFIR throughout the data collection process from development of interview guides to development of qualitative codebooks and conducting analysis. Given the growing number of States proposing laws to limit opioid prescribing, understanding the experience of North Carolina in implementing the STOP act will inform how states might support their key partners for more effective policy implementation.

**References**

1. Rudd RA, Aleshire N, Zibbell JE, Gladden RM. Increases in drug and opioid overdose deaths—United States, 2000–2014. Am J Transplant. 2016;16(4):1323-1327.

2. Dowell D, Haegerich TM, Chou R. CDC guideline for prescribing opioids for chronic pain—United States, 2016. JAMA. 2016;315(15):1624-1645.

3. Damschroder LJ, Aron DC. Keith RE, Kirsh SR, Alexander JA, Lowery JC. Fostering implementation of health services research findings into practice: a consolidated framework for advancing implementation science. Implement Sci. 2009; 4:50.

## A176 Development and psychometric testing of the capacity to Treat Co-Occurring Chronic Pain and Opioid Use Disorder (CAP-POD) Questionnaire

### Allyson Varley^1^, Stefan Kertesz^1^, Andrea Cherrington^1^, Aerin deRussy^2^, April Hoge^2^, Kevin Riggs^1^, Peter Hendricks^1^

#### ^1^University of Alabama at Birmingham, Birmingham, AL, USA; ^2^Birmingham VA Medical Center, Birmingham, AL, USA

##### **Correspondence:** Allyson Varley (avarley@uab.edu)

**Background**

Patients with the combination of chronic pain and opioid use disorder have unique needs and may present a challenge for clinicians and health care systems. Primary care providers’ (PCPs) capacity to deliver high quality, evidence-based care for this important subpopulation is unknown. This study’s objective was to develop and test a survey of PCP capacity to treat co-occurring chronic pain and OUD.

**Materials and Methods**

Capacity to Treat Co-Occurring Chronic Pain and Opioid Use Disorder (CAP-POD) questionnaire items were developed over a 2-year process including literature review, semi-structured interviews, expert panel review, and pilot testing. A national sample of PCPs (MD, DO, NP, PA) were recruited via email to complete an online survey that included the 44-item CAP-POD questionnaire. Response options ranged from 1 (strongly disagree) to 7 (strongly agree). CAP-POD items were analyzed for dimensionality and inter-item reliability. We compared mean scores across provider characteristics (education, setting, years’ experience) to identify potential gaps in capacity.

**Results**

509 PCPs from across the US completed the questionnaire. Principal component analysis resulted in a 22-item questionnaire. Twelve more items were removed because of their influence on coefficient alphas, resulting in a 10-item questionnaire with 4 domains: 1) Motivation to Treat patients with chronic pain and OUD (α =.87, M=3.49,SD=1.48); 2) Trust in Evidence (α =.87, M=5.67, SD=1.03); 3) Assessing Risk (α =.82, M=5.45, SD=1.19); and 4) Patient Access to therapies (α =.79; M=3.06, SD=1.47). Mean scores across the four scales differed significantly (p<.001).

**Conclusions**

We developed a short, 10-item questionnaire that assesses the capacity of PCPs to implement best practice recommendations for the treatment of co-occurring chronic pain and OUD. The questionnaire and scales demonstrated adequate validity and good inter-item reliability. PCPs reported moderate trust in evidence of treatments for co-occurring chronic pain and OUD, and in their ability to identify patients at risk. Conversely, they had low desire to treat these patients, and see their patients’ access to relevant services as suboptimum. These data imply a service shortfall that will likely require fixing with additional training, service design, and incentives. The questionnaire provides a brief, validated evaluation tool for such interventions.

## A177 Tailoring practice facilitation to optimize alcohol-related care in hepatology clinics: barriers and facilitators and feedback on an implementation intervention

### Ann Marie Roepke^1,2^, Madeline Frost^1^, George Ioannou^1,2^, Judith Tsui^2,3^, Jennifer Edelman^4^, Bryan Weiner^2^, Amy Edmonds^1,2^, Emily Williams^1,2,5^

#### ^1^VA Puget Sound Health Care System, Seattle, WA, USA; ^2^University of Washington, Seattle, WA, USA; ^3^Harborview Medical Center, Seattle, WA, USA; ^4^Yale University, New Haven, Connecticut, USA; ^5^Seattle-Denver COIN, Seattle, WA, USA

##### **Correspondence:** Ann Marie Roepke (ann.roepke2@va.gov)

**Background**

Unhealthy alcohol use exacerbates and complicates treatment of chronic liver disease [1]. Yet, evidence-based alcohol-related care is inconsistently delivered in hepatology clinics [2]. Informed by research supporting practice facilitation as an effective implementation strategy in primary care, we aim to tailor practice facilitation to implement evidence-based alcohol-related care in four Veterans Affairs (VA) hepatology clinics [3]. Here we describe barriers and facilitators garnered from qualitative interviews with key stakeholders at 2 of 4 clinics to inform intervention tailoring.

**Materials and Methods**

We recruited key stakeholders (n=23) including clinicians (MD, NP), clinical staff (RN, LPN, MSW), and administrators responsible for caring for Veterans with liver conditions. Semi-structured qualitative interviews were developed using the Consolidated Framework on Implementation Research (CFIR) and specifically focused on understanding outer and inner setting and individual domains [4]. We elicited stakeholders’: (1) context for, experiences with, and perspectives about providing care to Veterans with liver conditions and unhealthy alcohol use; and (2) feedback regarding a practice facilitation intervention. Rapid content analysis was used to extract relevant themes.

**Results**

Qualitative interviews highlighted barriers to and facilitators of providing alcohol-related care and tailoring practice facilitation. Barriers included lack of systematic alcohol screening procedures; variability in clinicians’ knowledge, comfort, and interest in providing evidence-based treatments (e.g., medications for alcohol use disorder); perceived inadequate linkage with specialty addiction treatment and/or behavioral health; and challenges related to staffing time/availability. Facilitators included system- and clinic-level leadership support, histories of successful quality improvement efforts, staff who are well-prepared to serve as clinical champions, consensus regarding the importance of addressing alcohol use, enthusiasm for several planned practice facilitation elements, and cohesive teams. Consistent with the CFIR, findings from 23 liver clinic staff suggest that a practice facilitation intervention can capitalize and build on existing setting and individual characteristics.

**Conclusions**

Specifically, the intervention should build on existing leadership support, enthusiasm, team cohesiveness, and successful past quality improvement efforts and include: (1) assistance integrating standardized alcohol screening into clinic flow; (2) training and ongoing support regarding evidence-based care for unhealthy alcohol use; and (3) linkages with or internal capacity building for behavioral health or specialty addictions treatment.

**References**

1. Fuster D, Samet JH. Alcohol use in patients with chronic liver disease. N Engl J Med. 2018; 379(13):1251-1261.

2. Owens MD, Ioannou GN, Tsui JI, Williams EC. Receipt of alcohol-related care among patients with HCV and unhealthy alcohol use. Drug Alcohol Depend .2018; 188:79-85.

3. Baskerville NB, Liddy C, Hogg W. Systematic review and meta-analysis of practice facilitation within primary care settings. Ann Fam Med. 2012;10(1):63-74.

4. Damschroder LJ, Aron DC. Keith RE, Kirsh SR, Alexander JA, Lowery JC. Fostering implementation of health services research findings into practice: a consolidated framework for advancing implementation science. Implement Sci. 2009;4:50.

## A178 Implementation science and entrepreneurship: harnessing synergy for discovery uptake

### Enola Proctor^1^, Emre Toker^2^, Rachel Tabak^1^, Cole Hooley^3^, Virginia McKay^1^

#### ^1^Brown School, Washington University in St. Louis, St. Louis, MO, USA; ^2^Arizona State University, Tempe, AZ, USA; ^3^Brigham Young University, Provo, UT, USA

##### **Correspondence:** Enola Proctor (ekp@wulst.edu)

**Background**

Implementation science and social entrepreneurship share the goal of accelerating the uptake of medical discoveries for widespread use in clinical and community healthcare. This paper reports infrastructure and activities within a CTSA program to advance synergy between these two fields. This work is based on the assumptions that implementation science can benefit from entrepreneurship’s emphasis on market demand, while entrepreneurship can benefit from implementation science’s emphasis on data, models, and context—particularly the policy, social, and organizational context of healthcare.

**Materials and Methods**

Our CTSA conducted activities to identify challenges and areas of complementarity between implementation researchers and entrepreneurs. First, we held a series of meetings between implementation researchers and entrepreneurs, the purpose of which was to identify and map shared and distinctive approaches regarding criteria for roll-out readiness, metrics for assessing return on investment, roll-out processes, risk tolerance, and priority products. Second, we convened an IdeaBounce@ experience, an event in which implementation researchers “pitched” their innovations to an audience of entrepreneurs and received feedback. A qualitative researcher observed both activities, taking notes and synthesizing observations.

**Results**

This work revealed that implementation researchers and entrepreneurs had different perspectives on: criteria for rollout readiness, return on investment, risk tolerance, and product goals. Implementation researchers focus on empirical evidence of innovation benefit, minimizing risk and unanticipated consequences, and carefully sequenced steps in implementation. Entrepreneurs focused on market demand, innovation cost, numbers expected to benefit from the innovation, and infrastructure and payment required for sustainment.

**Conclusions**

Harnessing the synergy between these disciplines can advance full realization of the benefits of biomedical research health care and population health. Both fields face the reality that the number of discoveries needing translational support greatly exceeds available funding and absorptive capacity. Innovative approaches, infrastructure development, and training are required to leverage the yet-untapped synergy between these fields. We identify a number of mechanisms for advancing synergy between these two fields--an exemplar of team science.

## A179 Implementing across an integrated health care system and a state criminal justice system – lessons from a peer support intervention for veterans leaving incarceration

### D. Keith McInnes^1^, Justeen Hyde^1,2^, Thomas Byrne^3^, Beth Ann Petrakis^1^, Vera Yakovchenko^1^

#### ^1^Department of Veterans Affairs, Boston, MA, USA; ^2^Center for Healthcare Organization and Implementation Research, Boston, MA, USA; ^3^Boston University, Boston, MA, USA

##### **Correspondence:** D. Keith McInnes (keith.mcinnes@va.gov)

**Background**

Veterans just released from incarceration (“reentry veterans”) experience barriers to housing and health services, which heightens risk for homelessness, recidivism, morbidity and mortality [1]. The Veterans Health Administration (VHA) employs peer support specialists, but does not have dedicated peers supporting the multi-faceted needs of reentry veterans. We developed and implemented the VHA’s first peer-support [2] initiative for reentry veterans, the Post-Incarceration Engagement (PIE) program.

**Materials and Methods**

We used a Facilitation strategy to implement PIE in Massachusetts. External facilitation included developing an intervention manual, worksheets, training curriculum, and hiring veteran peers. Peers met weekly with reentry veterans to address life priorities, and support community reintegration in 3 areas: linkage to services, skill building, and social support. External facilitation also involved stakeholder engagement with Massachusetts’ Department of Correction (DOC), Mental Health, and Veterans Services; network development with community-based service providers; and marketing presentations to VHA regional homelessness programs and to veteran inmates in DOC facilities. Internal facilitation, led by a social worker champion, involved educating service line chiefs at Massachusetts’ VHA medical centers about the benefits of the PIE program.

**Results**

High levels of collaboration were achieved between with the Department of Veterans affairs central office justice programs and a coalition of state agencies and community organizations. PIE served 30 reentry veterans released from 6 DOC prisons and 3 county jails. Peers had over 200 encounters with these veterans. PIE reentry veterans had a higher likelihood than comparison veterans of linkage to substance use treatment (80% versus 19%, respectively, P<0.001) and mental health care (87% versus 64%, respectively, borderline significant). They were more likely than comparison veterans to access VHA homelessness services, such as the domiciliary inpatient program (53.3% versus 2.7%, respectively, P<0.001) and short-term emergency beds (26.7% versus 5.4%, respectively, P=0.05). Though not statistically significant, trends suggested reentry veterans had greater access, than comparison veterans, to transitional grant and per diem housing (26.7% versus 25.4%, respectively) and to federal housing vouchers (6.7% versus 3.7%, respectively).

**Conclusions**

In summary, a facilitation strategy contributed to the implementation of a reentry veteran peer support program which shows promise in improving access to housing and health services.

**References**

1. Visher CA, Travis J. Transitions from prison to community: understanding individual pathways. Annu Rev Sociol. 2003;29:89–113.

2. Chinman M, George P, Dougherty RH, Daniels AS, Ghose SS, Swift A, Delphin-Rittmon ME. Peer support services for individuals with serious mental illnesses: assessing the evidence. Psychiatr Serv. 2014; 65(4):429–41.

## A180 Implementation of evidence-based mental health interventions in rural settings: a scoping literature review

### Christopher Weatherly, Meagan Pilar

#### Brown School, Washington University in St. Louis, St. Louis, MO, USA

##### **Correspondence:** Christopher Weatherly (weatherly@wustl.edu)

**Background**

Bridging the gap between rural and urban mental health services requires developing creative solutions to complex challenges that are unique to rural areas. Previous research has documented numerous mental health disparities in rural settings, including higher rates of depression and suicide [1-2], and limited resources and barriers to care restrict access to mental health services for rural residents [3-4]. Despite the demonstrated need for intervention, there are significant challenges associated with implementing evidence-based interventions in rural community settings [5]. However, little is known about the current state of mental health implementation in rural settings. This scoping review thus aims to address this gap by providing a systematic overview of how evidence-based mental health interventions are being implemented within rural community settings.

**Materials and Methods**

The scoping review is structured according to Peters et al’s framework for conducting scoping studies [6]. We searched the following databases: PubMed, CINAHL, PsychINFO, EMBASE, SCOPUS, Web of Science, ClinicalTrials.gov, and the Cochrane Library. The three search strings used for this review included variations of “mental health,” “implementation,” and “rural.” Inclusion criteria: 1) empirical study involving the implementation of a mental health intervention in a rural setting; 2) English language; and 3) peer-reviewed journal publication. No restrictions were placed on year of publication, sample size, or research design. Screening and review of articles will be carried out by two reviewers. A third reviewer will be involved as needed for consensus. We will assess and review findings through both tabular and thematic analyses.

**Results**

We are currently in the process of screening abstracts. In April-May 2019, we will review and extract full texts. We will present the yields, characteristics of included articles, and a description of evidence-based mental health interventions being implemented and tailored to the unique rural context. We will also describe the implementation strategies, adaptation methods, and outcomes associated with these studies.

**Conclusions**

This project is intended to provide an overview of the current state of mental health implementation research in rural settings to identify gaps in previous research and identify areas for future work.

**References**

1. Eberhardt MS, Pamuk ER: The importance of place of residence: examining health in rural and nonrural areas. Am J Public Health. 2004;94:1682–1686.

2. Simmons LA, Braun B, Charnigo R, Havens JR, Wright DW. Depression and poverty among rural women: a relationship of social causation or social selection? J Rural Health. 2008;24:292–298.

3. Orloff TM, Tynmann B. Rural health: an evolving system of accessible services. Washington, DC: National Governors’ Association. 1995.

4. Pathman DE, Steiner BD, Jones BD, Konrad TR. Preparing and retaining rural physicians through medical education. Acad Med. 1999;74:810–820.

5. Smith TA, Adimu TF, Martinez AP, Minyard K. Selecting, adapting, and implementing evidence-based interventions in rural settings: an analysis of 70 community examples. J Health Care Poor Underserved. 2016;27(4A):181-193.

6. Peters MDJ, Godfrey C, McInerney P, Baldini Soares C, Khalil H, Parker D. Chapter 11: Scoping Reviews. In: Aromataris E, Munn Z, eds. Joanna Briggs Institute Reviewer’s Manual. The Joanna Briggs Institute; 2017. https://reviewersmanual.joannabriggs.org. Accessed 1 Feb 2019

## A181 Identifying determinants of implementation of the Cornerstone Intervention to develop a user-centered implementation manual

### Danielle Adams^1^, Andrea Cole^2^, Michelle Munson^3^, Curtis McMillen^1^, Victoria Stanhope^3^

#### ^1^University of Chicago, Chicago, IL, USA; ^2^New York State Psychiatric Institute, New York, NY, USA; ^3^New York University, New York, USA

##### **Correspondence:** Danielle Adams (daniadams@uchicago.edu)

**Background**

Transition-age youth have elevated rates of mental disorders, and often do not receive services. Few mental health interventions have been developed for older youth in transition, and even fewer have been found to be effective over the transition to adulthood. Cornerstone, a theoretically-guided intervention has shown promise for addressing the mental health needs of this group as they emerge into adulthood [1]. Cornerstone provides case management, trauma-focused cognitive behavioral therapy, mentoring/peer support, and community-based in-vivo practice to address stigma and mental health symptoms, and practical skill development to improve the transition to independence among TAY with mental health conditions [2]. Using the Consolidated Framework for Implementation Research (CFIR) [3], this study examined determinants of implementation of Cornerstone with the goal of creating an implementation manual to guide real-world effectiveness trials and scalability efforts.

**Materials and Methods**

Within a Hybrid Type 2 trial, investigators developed a semi-structured interview protocol using implementation strategy domains as a framework [4]. Face-to-face interviews were conducted with clinic staff (n = 8) and state-level leadership (n = 3), and research staff (n = 1) on determinants of implementation for Cornerstone, such as planning, training, and supervision. Using grounded theory with sensitizing concepts, multiple coders analyzed the data using constant comparison. Iterative discussion(s) occurred over six months until saturation was met.

**Results**

Using the CFIR [3], we created a comprehensive review of implementation determinants of the Cornerstone intervention, as well as a review of contextual information (e.g., state policy reforms) from state-level stakeholders which may impact future scalability and sustainability of the intervention. Outer setting themes converged around the external policy context and incentives, with respondents discussing value-based payment and the importance of tracking non-billable tasks of mentors. Process themes pointed to important areas of planning: integration of mentors within the clinic, regular team check-ins, and the increased use of technology by mentors. Participants qualitatively reported high acceptability and feasibility for the cornerstone intervention and its components.

**Conclusions**

Results will be combined with user-centered design approaches, such as the simplification principle [5], to develop a Cornerstone Implementation Manual that will assist us in moving toward testing effectiveness of a much-needed intervention for TAY.

**Trial Registration** ClinicalTrials.gov NCT02696109

**References**

1. Cole AR, Munson MR, Ben David S, Sapiro B, Railey J, Stanhope V. Feasibility, acceptability, and preliminary impact of the Cornerstone mentoring program. Paper presented at the 10th Annual Conference on the Science of Dissemination and Implementation, Arlington, VA; 2017.

2. Munson MR, Cole A, Stanhope V, Marcus SC, McKay M, Jaccard J, Ben-David S. Cornerstone program for transition-age youth with serious mental illness: study protocol for a randomized controlled trial. Trials. 2016;17(1):537-550.

3. Damschroder LJ, Aron DC, Keith RE, Kirsh SR, Alexander JA, Lowery JC. Fostering implementation of health services research findings into practice: a consolidated framework for advancing implementation science. Implement Sci. 2009; 4(1):50.

4. Powell BJ, McMillen JC, Proctor EK, Carpenter CR, Griffey RT, Bunger AC, Glass JE, York JL. A compilation of strategies for implementing clinical innovations in health and mental health. Med Care Res Rev. 2012;69(2):123-157.

5. Lyon AR, Koerner K. User‐centered design for psychosocial intervention development and implementation. Clin Psychol. 2016;23(2):180-200.

## A182 Understanding the critical elements of an Integrated Scaling up Approach (ISA)

### Marianne Farkas, Sigal Vax, Vasudha Gidugu, Kim Mueser, Chitra Khare, Philippe Bloch

#### Boston University, Boston, MA, USA

##### **Correspondence:** Marianne Farkas (mfarkas@bu.edu)

**Background**

To reduce the gap between research and practice in community mental health services, there is a critical need to develop new methods for scaling up evidence-based practices [1]. To increase widespread access to effective practices, we are in the process of developing an Integrated Scaling Approach (ISA) for the efficient largescale implementation of empirically based interventions for people with psychiatric disabilities.

**Materials and Methods**

We conducted a scoping review of the literature across various fields, including mental health, our own field of practice, as well as public health, business, and education, fields in which large scale implementation efforts are common. We then focused on examples of the implementation of employment initiatives, which have become an important focal point for change in practice and policies in mental health agencies and systems interested in the recovery of people with psychiatric disabilities [2-3]. While interventions have proven effective in supporting employment for this population, the number of people benefiting from them remains limited [4-6]. The scoping review included interviews of a range of stakeholders, with experiences in implementing such employment initiatives in their state or region (whether successful or unsuccessful) and expertise in program leadership, employment services, mental health and vocational rehabilitation services administration and policy.

**Results**

Both literature and interviews were analyzed to identify recurring themes and critical components of large-scale implementation in community mental health services. Analyses currently in process will identify characteristics of these critical components drawn from the cross-field scoping review. A pilot test of the ISA and a final evaluation will be conducted in two states in 2020-2021.The analyses of the scoping review and the results of the pilot and evaluation studies will be used to create a handbook for intervention researchers, system and program administrators and knowledge translation specialists to use, when scaling up new interventions in mental health.

**Conclusions**

In this presentation, we will share the data collection, analysis process, and preliminary characteristics of the ISA as identified through the comprehensive scoping review.

**References**

1. Wiltsey-Stirman S, Gutner CA, Langdon K, Graham JR. Bridging the gap between research and practice in mental health service settings: An overview of developments in implementation theory and research. Behav Ther. 2016;47:920-936. doi:10.1016/j.beth.2015.12.001.

2. Langi FLFG, Balcazar FE. Risk factors for failure to enter vocational rehabilitation services among individuals with disabilities. Disabil Rehabil. 2017;39(26):2640-2647. doi:10.1080/09638288. 2016.1236410.

3. Bergmark M, Bejerholm U, Markström U. Critical components in implementing evidence-based practice: a multiple case study of Individual Placement and Support for people with psychiatric disabilities. Soc Policy Adm. 2018;52(3):790-808. doi:10.1111/spol.12243.

4. Bazelon Center for Mental Health Law. Advances in Employment Policy for Individuals with Serious Mental Illness. Washington DC; 2018. http://www.bazelon.org/wp-content/uploads/2018/10/Supported-Employment-Report_Oct-2018.pdf. Accessed 9 March 2019.

5. Interdepartmental Serious Mental Illness Coordinating Committee. The way forward: federal action for a system that works for all people living with smi and sed and their families and caregivers. Washington DC; 2017. https://store.samhsa.gov/system/files/pep17-ismicc-rtc.pdf. Accessed 9 March 2019.

6. Johnson-Kwochka A, Bond G, Becker, Deborah R, Drake RE, Greene MA. Prevalence and quality of Individual Placement and Support (IPS) supported employment in the United States. Adm Policy Ment Heal Ment Heal Serv Res. 2017;44:311-319. doi:10.1007/s10488-016-0787-5.

## A183 Applying implementation science for real world impact: operationalized core practice components, feasibility testing, and next steps

### William Aldridge, Rebecca Roppolo, Julie Austen, Robin Jenkins

#### University of North Carolina at Chapel Hill, Chapel Hill, NC, USA

##### **Correspondence:** William Aldridge (will.aldridge@unc.edu)

**Background**

Implementation science is at risk to suffer from the same challenge it was designed to address: a lack of translation into real world application. Complicating this challenge is that robust application of implementation science requires supporting behavior change at each of individual, organizational, and system levels. “Technical assistance,” “facilitation,” and “implementation support” are terms often used to describe the concept of “implementation practice.” Regardless of these labels, what drives the effective application of implementation science within real world environments?

**Materials and Methods**

Drawing from a review of relevant literature and our experience facilitating the real-world application of implementation science, members of The Impact Center at FPG organized ten theoretically- and empirically-informed core practice components to strengthen implementation support processes. These ten proposed core practice components underwent initial feasibility testing within two projects involving implementation support for communities scaling an evidence-based system of parenting interventions. For more than two years, implementation specialists have tracked their utilization of the practice components across interactions with community sites. Implementation specialists working to build the capacity of intermediary partners also tracked their use of practice components. All community and intermediary sites received monthly surveys to report process and short-term outcomes. Intended long-term outcomes of implementation support (capacity to support implementation best practices at community and intermediary levels) were assessed every six months.

**Results**

Initial results suggest that the ten proposed practice components are an effective way to organize the work of implementation support. Supported sites reported favorable process outcomes, such as acceptability, feasibility, and appropriateness. Short-term outcomes, such as working alliance, have been useful markers for early successes or challenges. Long-term capacity outcomes have demonstrated improvement over time. Notwithstanding these strengths, Center implementation specialists voiced a need for greater clarity about operational activities related to the practice components. This recently led members of Center to re-operationalize the components to better support consistent application.

**Conclusions**

Next steps include the development of a complete practice profile, stronger training and fidelity assessment materials, and formative evaluation methods to test statistical associations between the components and intended short- and long-term outcomes.

## A184 If you want more research based practice, you need more practice based and early stage D&I trained researchers (borrowed and slightly changed from Larry Green)

### Rodger Kessler, Cady Berkel, Matthew Buman, Stephanie Brenhofern, Scott Leischow

#### Arizona State University, Tempe, AZ, USA

##### **Correspondence:** Rodger Kessler (rodger.kessler@asu.edu)

**Background**

While training programs in D&I research have emerged over the last few years, they have generally been limited to single institutions or individuals traveling to national training sites. This has limited reach of training and opportunities for multi-institutional development of broad D&I capacity. In addition, investigators must effectively engage with other partners, from clinical trials centers to community partners and policymakers. Such engagement skills need to be embedded in the core D&I training. This includes assisting earlier stage translational scientists’ participation in next stage activities, with designing for dissemination at the forefront.

**Materials and Methods**

We designed a yearlong D&I training program for Arizona State University faculty and other researchers across the state, borrowing from the TIDIRH curriculum. Implementation began in February 2019. Participant expectations include: on-site attendance at presentations by local and national D&I scholars, completion of readings between sessions, and regularly working with an assigned mentor to generate a project to move their D&I effort ahead.

**Results**

We started the program recruiting the capacity of 25 trainees. After our kickoff event, seven participants withdrew (n=3 perceived relevance, n=2 distance/time, n=1 moved, n=1 unknown). We invited two trainees from the waitlist and now have 20 trainees who span the translational spectrum: Basic research (n=5); Pre-clinical (n=4); Efficacy/Adaptation (n=9); Implementation in clinical and community settings (n=12); Studying health outcomes at the population level (n=7) and career stage [graduate student/postdoc (n=4); assistant professor (n=9); associate professor (n=5); clinical professor (n=1); and adjunct professor (n=1)]. Total n is greater than 20 because we allowed trainees to select multiple areas. Each trainee was paired with one of nine mentors.

**Conclusions**

We generated a multi institutional D&I training program. Recruitment was easily accomplished. Loss of participants due to absence of distance learning needs attention and lack of fit to earlier stage translational scientists suggests we need to refine the material presented at the first event to better include those individuals. Little D&I training has been developed for the basic end of the translational spectrum; we are attempting to fill that gap. We will report further on evaluation data and the projects that trainees generate.

